# Infrastructure, policy and regulatory interventions to increase physical activity to prevent cardiovascular diseases and diabetes: a systematic review

**DOI:** 10.1186/s12889-022-14841-y

**Published:** 2023-01-16

**Authors:** Solange Durão, Jacob Burns, Bey-Marrié Schmidt, David Tumusiime, Ameer Hohlfeld, Lisa Pfadenhauer, Clémence Ongolo-Zogo, Eva Rehfuess, Tamara Kredo

**Affiliations:** 1 Cochrane South Africa, South African Medical Research Council, Cape Town, South Africa; 2 Institute for Medical Information Processing, Biometry and Epidemiology, Pettenkofer School of Public Health, LMU Munich, Munich, Germany; 3 Pettenkofer School of Public Health, Munich, Germany; 4 School of Public Health, University of the Western Cape, Cape Town, South Africa; 5 College of Medicine and Health Sciences, University of Rwanda, Kigali, Rwanda; 6 Temerty Faculty of Medicine, University of Toronto, Toronto, Canada

**Keywords:** Physical activity, Noncommunicable diseases, Cardiovascular disease, Diabetes, Infrastructure, Policy, Regulation

## Abstract

**Background:**

Noncommunicable diseases are major contributors to morbidity and mortality worldwide. Modifying the risk factors for these conditions, such as physical inactivity, is thus essential. Addressing the context or circumstances in which physical activity occurs may promote physical activity at a population level. We assessed the effects of infrastructure, policy or regulatory interventions for increasing physical activity.

**Methods:**

We searched PubMed, Embase and clinicaltrials.gov to identify randomised controlled trials (RCTs), controlled before-after (CBAs) studies, and interrupted time series (ITS) studies assessing population-level infrastructure or policy and regulatory interventions to increase physical activity. We were interested in the effects of these interventions on physical activity, body weight and related measures, blood pressure, and CVD and type 2 diabetes morbidity and mortality, and on other secondary outcomes. Screening and data extraction was done in duplicate, with risk of bias was using an adapted Cochrane risk of bias tool. Due to high levels of heterogeneity, we synthesised the evidence based on effect direction.

**Results:**

We included 33 studies, mostly conducted in high-income countries. Of these, 13 assessed infrastructure changes to green or other spaces to promote physical activity and 18 infrastructure changes to promote active transport. The effects of identified interventions on physical activity, body weight and blood pressure varied across studies (very low certainty evidence); thus, we remain very uncertain about the effects of these interventions. Two studies assessed the effects of policy and regulatory interventions; one provided free access to physical activity facilities and showed that it may have beneficial effects on physical activity (low certainty evidence). The other provided free bus travel for youth, with intervention effects varying across studies (very low certainty evidence).

**Conclusions:**

Evidence from 33 studies assessing infrastructure, policy and regulatory interventions for increasing physical activity showed varying results. The certainty of the evidence was mostly very low, due to study designs included and inconsistent findings between studies. Despite this drawback, the evidence indicates that providing access to physical activity facilities may be beneficial; however this finding is based on only one study. Implementation of these interventions requires full consideration of contextual factors, especially in low resource settings.

**Trial registration:**

PROSPERO 2018 CRD42018093429.

**Supplementary Information:**

The online version contains supplementary material available at 10.1186/s12889-022-14841-y.

## Background

Non-communicable diseases (NCDs), such as cardiovascular diseases (CVDs) and type 2 diabetes, are a major contributor to morbidity and mortality worldwide [1]. CVDs are the leading cause of death globally and account for 17.9 million deaths annually. Similarly, the number of premature deaths from type 2 diabetes, a risk factor for CVD, has increased to 1.5 million deaths in 2019, while 422 million adults continue to live with type 2 diabetes [2]. Of all premature deaths due to NCDs, more than 77% occur in LMICs [3] and more than 80% of people living with type 2 diabetes reside in LMICs [4, 5]. The World Health Assembly, through its 2013 global monitoring and evaluation framework for the prevention and control of NCDs, called for a 25% reduction in NCD deaths, including from CVDs and type 2 diabetes, in individuals aged 30–70 years by 2025 [6].

To achieve this, we need to address the modifiable risk factors for CVD and type 2 diabetes, which include, among others, overweight and obesity, and physical inactivity [7]. Indeed, action to address physical inactivity has been emphasised through the Global Action Plan on Physical Activity 2018–2030 (GAPPA): more active people for a healthier world [8], with concrete guidance on necessary levels of physical activity offered through WHO guidelines on physical activity and sedentary behaviour in 2020 [9]. Despite there being a large body of evidence on the health benefits of physical activity, implementing solutions for reducing physical inactivity remains a common public health challenge globally [10, 11].

### Population-level physical activity interventions

Population-level health interventions are policies or programmes that aim to mitigate the distribution of health risk by addressing the underlying socioeconomic, environmental, behavioral or cultural conditions in which people live and work [12]. They target the whole population or population groups regardless of variations in individual risk status, thus addressing the underlying causes of diseases and minimising exposure of the population to the risk factors for those diseases [13, 14].

A wide-range of population-level health interventions have been considered in efforts to increase physical activity or address barriers to physical activity [15]. These types of interventions require a political and social approach, and they vary from superficial to radical approaches [14]. Superficial approaches depend more on individual agency for behavior change and include, for example, mass campaigns to promote physical activity. Radical approaches aim to change the context or circumstances, in which behavior occurs, by implementing structural changes to social institutions and norms that shape the behavior of individuals. Examples of radical approaches include interventions addressing infrastructure (e.g. cycling lanes and outdoor gyms) and policies or regulations (e.g. compulsory school or workplace physical activity policies, and guidelines for urban design and planning).

Physical activity interventions may directly improve physical and mental health but they may also indirectly affect health through influencing diet choices and smoking behavior [16], which are additional factors influencing CVD and type 2 diabetes outcomes.

Existing reviews on population-level interventions addressing NCD risk factors focus on dietary risk factors at the population-level [12, 17–21]. Existing or ongoing reviews on physical activity interventions focus on individual’s clinical conditions, treatment and rehabilitation [22–24], or on community, school or workplace settings [15, 23, 25]. One review includes population-level interventions but focuses on interventions that promote walking only [26]. Existing guidelines on PA focus on individual-level recommendations for time spent in PA across age groups rather than on recommendations regarding population-level interventions [27].

This review thus aims to assess the effects of infrastructure, policy or regulatory interventions for increasing physical activity with the primary or secondary aim to prevent cardiovascular diseases and type 2 diabetes. Given the high burden of NCDs in LMICs and the fact that most of these types of interventions are implemented in high-income countries, we also aim to consider the implications for low- and middle-income countries (LMICs).

## Methods

This protocol was registered with the PROSPERO International prospective register of systematic reviews (PROSPERO 2018 CRD42018093429) and was conducted according to the Preferred Reporting Items for Systematic Reviews and Meta-Analyses [28].

### Eligibility criteria

#### Types of studies

Due to the ecological nature of research on population-level interventions, we expected that much of the evidence exists as non-randomized studies (NRS). We thus included the following randomized and selected non-randomized study designs: Randomized controlled trials (RCTs), cluster RCTs, controlled before-after (CBA) studies, and interrupted time-series (ITS) studies (see definitions in the protocol). We included studies in any language and regardless of their publication status.

#### Types of participants and setting

We included studies conducted in healthy populations of any age or gender and not diagnosed with CVDs or diabetes; these populations could have presented with risk factors for CVDs or diabetes. Studies that only included participants with a particular disease or condition were excluded.

#### Types of interventions

**Fig. 1:**
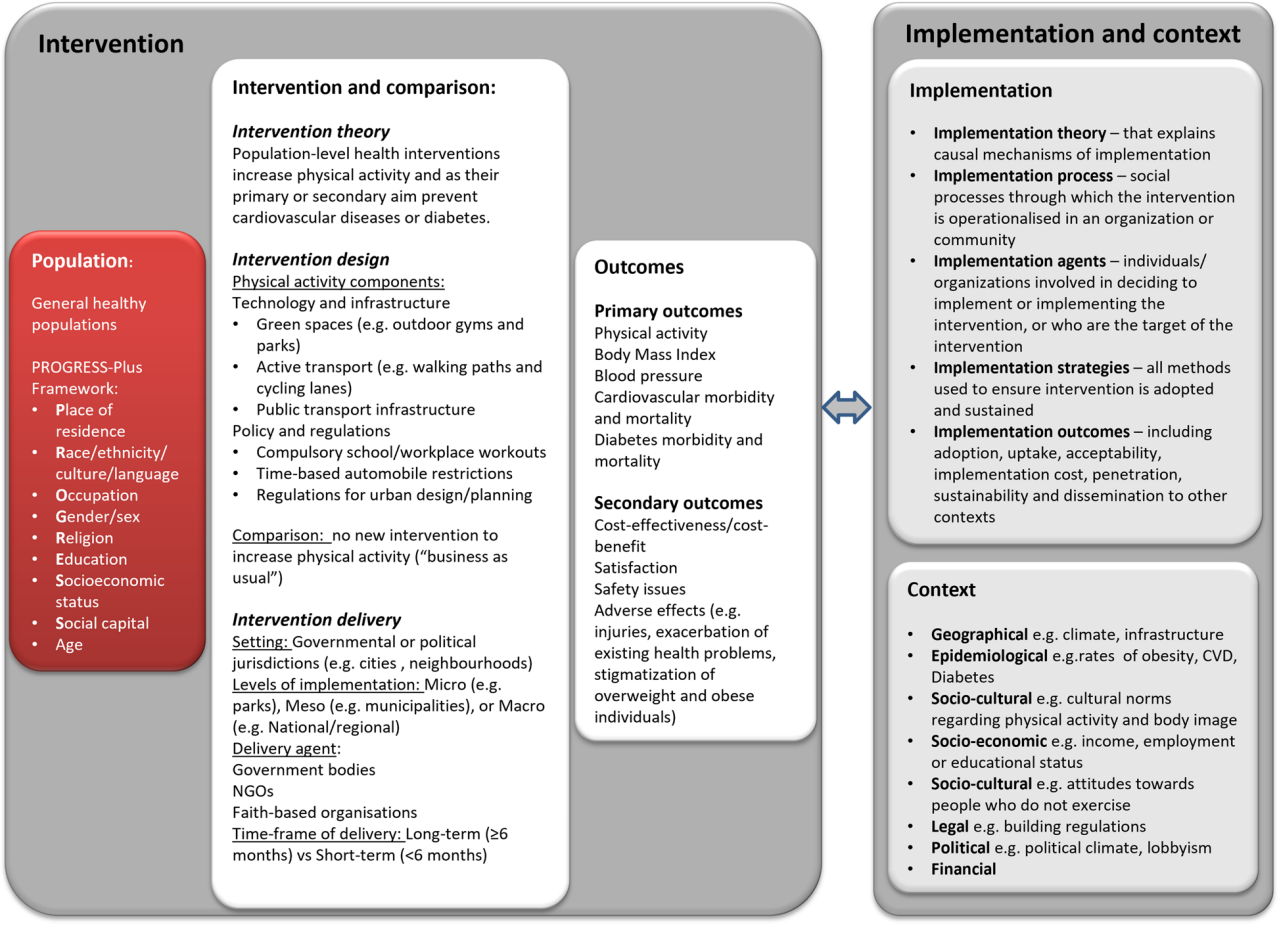
Logic model detailing the intervention components as well as the implementation and context factors that could affect the ability of the intervention to achieve the desired outcomes

The logic model (Fig. 1) details the types of interventions eligible for this review: 1) Infrastructure interventions that create physical spaces where people can engage in physical activity through exercise where they live, learn, work and play [29, 30] (e.g. green space interventions such as outdoor gyms and parks, active transport infrastructure such as walking and cycling lanes public transport infrastructure); and 2) Policy and regulatory interventions which can help plan, promote, and coordinate efforts to increase physical activity to be implemented as routine practice [22] (e.g. policies on compulsory school or workplace physical activity programmes such as national regulations for exercise in schools). Interventions had to have been implemented at the population level, i.e. at a governmental or political jurisdiction level, which refers to certain population or geographic areas with a defined legal authority such as cities, provinces, or countries. Interventions delivered one-on-one, in a small group format, or solely delivered in specific settings such as individual schools or workplaces (rather than at the level of a governmental or political jurisdiction) were excluded.

We included studies that compared the intervention of interest with no new intervention to enable or increase physical activity or with existing interventions to promote physical activity (i.e. “business as usual”).

Studies with complementary interventions (co-interventions) were included if these were delivered in both groups.

#### Types of outcome measures

We included studies that assessed at least one of our primary or secondary outcomes of interest, outlined below.

##### Primary outcomes

- 1- Physical activity: measures of population-level physical activity, e.g. duration, frequency, and proportion of people active or meeting specific physical activity recommendations. Physical activity measures could be related to walking, cycling, as well as with leisure time physical activity.
- 2- Body weight and related measures (e.g. BMI)
- 3- Blood pressure
- 4- CVD morbidity (e.g. incidence, prevalence, hospitalisation)
- 5- Diabetes morbidity (e.g. incidence, prevalence, hospitalisation)
- 6- CVD mortality
- 7- Diabetes mortality

##### Secondary outcomes

- 8- Costs and cost-effectiveness (as reported by study authors or by cost-related sub-studies of included studies)
- 9- Satisfaction or dissatisfaction with the intervention or control as reported by the population targeted by the intervention
- 10- Any report that the intervention impacts on equity issues (e.g. accessibility; safety for specific population groups; considering the PROGRESS-PLUS factors: Place of Residence, Race/Ethnicity, Occupation, Gender, Religion, Education, Socioeconomic Status, and Social Capital, and Plus represents additional categories such as Age, Disability, and Sexual Orientation) [31]
- 11- Any report of safety issues (e.g. accessibility of parks at night; street lights)
- 12- Any reports of adverse effects (e.g. injuries, exacerbation of existing health problems, stigmatization of obese or overweight individuals, exacerbation of body image issues)

### Search strategy

To identify relevant records, we searched three databases from their inception to February 2018 (PubMed, Embase and Web of Science). We updated the search in February 2020 in one key database (PubMed) which had retrieved most of the relevant records in the previous search. No restrictions on language or publication status were applied. We searched ClinicalTrials.gov in November 2021 for the recent initiation of relevant studies. The detailed search strategies are available in Additional file 1. We also screened the reference lists of included studies and of systematic reviews identified through the search.

### Data collection

#### Study selection and data extraction

All titles and abstracts were screened in Rayyan (https://rayyan.qcri.org/) by one reviewer to determine eligibility against the review inclusion criteria. For every novice reviewer taking part in screening, an initial 100 studies were screened independently and in duplicate by an experienced reviewer. If any relevant studies were excluded by the novice, these were discussed, and an additional 100 studies were screened in duplicate. Duplicate screening continued until the novice reviewers were proficient. Full-texts of potentially eligible records were screened independently and in duplicate using the Covidence platform [32], except for the trial registry results which were screened by one reviewer only. Disagreements regarding eligibility were resolved through discussion and involvement of a third reviewer, if necessary.

We used EndNote software [33] to manage retrieved records and to remove duplicate reports of the same study. All records related to the same study were grouped together so that the unit of study of the review was the unique study.

We extracted data independently and in duplicate in Covidence [32], and discrepancies were resolved through discussion or arbitration by a third author, if necessary.

#### Risk of bias assessment

We assessed the risk of bias in included studies independently and in duplicate using the Cochrane ‘Risk of bias’ tool, as modified by Cochrane EPOC, with separate criteria for controlled studies (RCTs, c-RCTs, CBAs and c-ITS) and for u-ITS (Cochrane Effective Practice and Organisation of Care (EPOC) [34]. For each criterion, each study was rated at high, low, or unclear risk of bias. Any disagreements were solved through discussion and reaching consensus or through checking with a third reviewer, if necessary.

#### Measures of treatment effect

For dichotomous outcomes we had planned to report the risk ratios (RR) of outcomes in the intervention group compared to the control group alongside the 95% confidence interval (CI). For continuous outcomes we had planned to report the mean difference (MD) between the change in the intervention and control groups if studies measured the outcomes in the same way and the standardized mean difference (SMD) if they did not measure them in the same way. However, due to substantial differences in analytical methods and reporting across included studies, we report the effect estimate reported by each included study.

#### Unit of analysis issues

For cluster RCTs that reported analyses at the individual level, we ascertained whether they reported the method used to account for clustering. For non-randomised studies, and RCTs with baseline imbalances, we reported estimates adjusted for baseline imbalances and other confounders, if this data were reported. If outcome data were available for multiple timepoints we reported the latest timepoint in the synthesis. In the supplementary material, which describe results of individual studies, we also grouped the outcomes according to the different periods of follow-up: short term (< 3 months), medium-term (3–6 months) and long-term (> 6 months).

#### Dealing with missing data

We did not contact the authors of included studies for clarification regarding study methods or results. We recorded all missing outcome data in the data extraction form and in the risk of bias table.

#### Assessment of heterogeneity

We assessed heterogeneity in relation to the PICO elements as well as context and implementation and documented this in tables summarising the included studies. As we did not conduct any meta-analyses, we were not able to assess heterogeneity by visually inspecting the confidence interval overlap in forest plots, or by using the Chi^2^ and I^2^ statistics.

#### Assessment of reporting biases

There were not enough studies reporting the same outcome (< 10), therefore no funnel plots were used to investigate the risk of publication bias.

#### Data synthesis

Due to substantial heterogeneity, we could not pool any results in meta-analyses. We thus synthesised the results based on effect direction, represented graphically using harvest plots [35]. Harvest plots are a clear and transparent way to portray evidence from a heterogeneous evidence base, especially where primary studies are not well-suited to statistical pooling [36, 37]. We created separate harvest plots for each intervention type, depicting effects on the primary outcomes of interest. The effect direction categories used for analysis included:

i) Clear effect favouring the intervention (when the effect measure favoured the intervention and the 95% CI did not cross the null),
ii) Unclear effect potentially favouring the intervention (when the effect measure favoured the intervention and the 95% CI crossed the null),
iii) No difference in effect (if the effects were identical in both groups or if the study only reported that no difference was observed between the groups, without reporting actual outcome values),
iv) Unclear effect potentially favouring the control (when the effect measure favoured the control and the 95% CI crossed the null), or
v) Clear effect favouring the control (when the effect measure favoured the control, and the 95% CI did not cross the null).

In cases where multiple measures and timepoints of the same outcome were reported in the same study, we selected those measures that most closely reflect the outcome of interest and the one measured at the longest timepoints. For example, one study reported both the observed number of people visiting the park as well as the proportion of people engaged in moderate vigorous physical activity (MVPA) at the park; the latter measure was selected for analysis.

#### Subgroup and sensitivity analyses

We intended to compare the effects of interventions across specific subgroups such as Global Burden of Disease (GBD) region, level of income, time of implementation, PROGRESS indicators, and according to the presence or absence of accompanying behavioural interventions. However, it was not possible to carry out these subgroup analyses.

We were not able to conduct sensitivity analyses as no meta-analysis were done due to the heterogeneity of the data.

### Assessment of certainty of evidence

Two reviewers assessed the overall certainty of the evidence using the Grading of Recommendations Assessment, Development and Evaluation (GRADE) approach. For RCTs the certainty of the evidence started at high and for NRSs at low. Five factors were then considered for downgrading the certainty (risk of bias, inconsistency, indirectness, imprecision, publication bias) and three factors were considered for upgrading the certainty (large effect size, all plausible confounding would reduce the demonstrated effect, dose response gradient). We did not upgrade the certainty of evidence for NRSs if there were existing reasons for downgrading [38]. For each factor, we provided a judgement with a rationale included as a footnote in the Summary of Findings (SoF) table. We prepared SoF tables for each comparison and all primary outcomes: 1) Measures of population-level physical activity, 2) CVD mortality, 3) Diabetes mortality, 4) CVD morbidity (e.g. incidence, prevalence, hospitalisation); 5) Diabetes morbidity (e.g. incidence, prevalence, hospitalisation), 6) Body weight and related measures and 7) Blood pressure.

## Results

### Results of the search

**Fig. 2:**
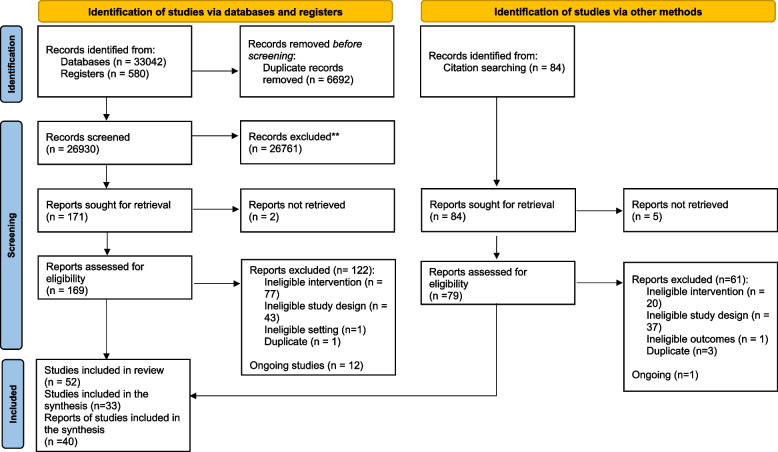
PRISMA flowchart of study selection

After screening 26,930 titles and abstracts and 248 full texts we identified 52 records for inclusion, 13 records as ongoing studies, and we excluded 185 records (Fig. 2). Of the excluded records, 97 assessed ineligible interventions, 80 did not have an eligible study design, one assessed ineligible outcomes, one took place in an ineligible setting, and four were duplicate records (Additional file 2).

Of the 52 records included, 40 records relating to 33 studies were included in the synthesis. The remaining 12 records relating to eight studies assessed broad multicomponent interventions that sometimes included a small environmental change component to promote physical activity. Although potentially relevant, these studies do not answer our review question, as we are not able to distinguish the effects of the specific environmental component and were thus not included in the graphical and narrative synthesis (they are described in Additional file 3).

### Description of included studies

#### Studies included in the synthesis (*n*=33)

Of the 33 studies, 28 were CBA studies, four were ITS studies [39–42] and one was a cluster RCT [43]. Table 1 provides an overview of included studies, which are described in more detail in S4 File. One study [41] presented an additional CBA analysis.

**Table 1:** Overview of studies included in the synthesis (*n* = 33)

Intervention category	Intervention type	Study design and ID
**1. Infrastructure** **(*n* = 31)**	**1.1 Green or other spaces** (e.g. upgrade or construction of parks, play/open streets) (*n* = 13)	1 cRCT: Veitch 2018 [43] 11 CBA studies: Goldsby 2016 [44], Cortinez O'Ryan 2017 [45], D’Haese 2015 [46], Quigg 2012 [47], Kubota 2019 [48], Ward Thompson 2019 [49], Richardson 2020 [50], Bohn-Goldbaum 2013 [51], Tester 2009 [52], Cohen 2009 [53], Slater 2016 [54] 1 ITS study: Branas 2011 [39]
	**1.2 Active transport** (new/upgraded cycling/walking infrastructure) (*n* = 18)	16 CBA studies: Østergaard 2015 [55], Goodman 2013 [56], Fitzhugh 2010 [57], Rissel 2015 [58], Jung 2017 [59], Brown 2016 [60], Benjamin Neelon 2015 [61]; McDonald 2013 [62], Prins 2017 [63], Frank 2019 [64], Dill 2014 [65] , Hong 2016 [66], Hirsch 2017 [67], West 2011 [68] [68], Pazin 2016 [69], Chapman 2014 [70] 2 ITS studies: Skov-Petersen 2017 [42]; Grunseit 2019 [40]
**2. Policy and regulations (*n* = 2)**	**2.1 Access to PA facilities**	1 ITS study: Higgerson 2018 [41] (includes CBA analysis)
	**2.2 Free bus travel**	1 CBA study: Green 2014 [71]

The sample size differed substantially across studies; studies including a fixed sample of individuals ranged from 73 [66] to 35,375 individuals [71]. Other studies did not assess a fixed sample of individuals, but instead observed individuals in a fixed setting. For example, some studies observed all users at specific parks or vacant lots [39, 50, 51, 53, 54], all residents of specific neighbourhoods or areas [56, 57, 59, 67], or all attendees of a specific facility [41]. Other studies used automated counters to record the number of cyclists passing a specific point [40, 42]. Seven studies assessed children specifically [45–47, 55, 61, 62, 65]. Five studies evaluated adults [48, 49, 58, 63, 69]. Several studies did not report the age of participants, often referring only to ‘residents’ [60, 66, 68].

Follow-up also varied widely across studies. Four studies had a follow-up of less than one year, ranging from 1 week [46] to 9 months [51]. Eleven studies had a follow-up of one year, while 18 studies had a follow-up of longer than a year, with the longest being 10 years [39, 41, 56, 67].

All but one of the included studies were conducted in high-income countries (HICs) with one study conducted in Brazil [69]. Most studies were from the USA (*n* = 14) [39, 44, 50, 52–54, 57, 60–62, 65–68], the UK (*n* = 5) [41, 49, 63, 71, 72], and Australia (*n* = 4). Two studies each were from Denmark [42, 55] and New Zealand [47, 70], and one study each were from Belgium [46], Chile [45], Korea [59], and Japan [48].

Of the 33 included studies, 31 assessed infrastructure interventions and two assessed policy and regulatory interventions to increase physical activity (Table 2). Of those studies assessing infrastructure interventions, 13 assessed interventions where green or other spaces were created or improved to enable and promote physical activity (e.g., upgrading or building parks, temporary closing of streets to encourage outside play and activities or installing cycle tracks), and 18 assessed active transport interventions, which consisted of improvements to walking or cycling infrastructure or extension of motorways away from residential areas. The two studies assessing policy and regulatory interventions evaluated a government scheme to increase access to physical activity facilities and a policy for free bus travel for youth.

**Table 2:** Characteristics of studies included in green or other spaces comparison

Study ID	Study design	Country	Description of Intervention and comparison	Participants/ Setting	Outcomes reported
Branas 2011 [39]	CBA study	USA	Greening of abandoned vacant lots (involved removing trash and debris, grading the land, planting grass and trees to create a park-like setting, and installing low wooden post-and-rail fences around each lot’s perimeter **vs** No greening of vacant lots	Vacant lots in urban Pennsylvania	Physical activity Blood pressure
Bohn-Goldbaum 2013 [51]	CBA study	Australia	Upgrade of playgrounds in a park **vs** Parks not renovated/with similar pre-renovation playgrounds as intervention park	Visitors to parks in lower SE urban neighbourhoods	Physical activity
Cortinez O'Ryan 2017 [45]	CBA study	Chile	Neighbourhood with street closed for play **vs** control neighbourhood	Children living in selected neighbourhoods	Physical activity
Cohen 2009 [53]	CBA study	USA	Park improvements ( e.g. new or refurbished gymnasiums, field improvements in watering and landscaping; improvements to picnic areas, upgrades to a walking path, and enhancements to a play-ground area..” **vs** No intervention	Visitors to study parks	Physical activity Park safety
D’Haese 2015 [46]	CBA study	Belgium	Play streets **vs** no intervention	Children living in streets part of study	Physical activity
Goldsby 2016 [44]	CBA study	USA	Living in close proximity (near) to new inner-city park (within 1.5 miles) **vs** living farther away from the park (further than 5 miles)	Children under 19 years old living in the intervention area	Body weight and related measures
Kubota 2019 [48]	CBA study	Japan	Construction of a new multipurpose exercise facility including indoor facilities (25 m pool, 170 m walking trail, multi-purpose gym, and group exercise rooms) and outdoor facilities (multi-purpose athletic field, 875 m walking trail, and park), accessible to all residents for a small fee + PA promotion **vs** No new exercise facility or PA promotion but with routine health promotion program	Adults 30–74 years old living in communities near the new infrastructure	Physical activity
Quigg 2012 [47]	CBA study	New Zealand	Playground upgrade **vs** no intervention	Children 5–10 years old attending schools in selected communities	Physical activity
Richardson 2020 [50]	CBA study	USA	Public housing development and greenspace landscaping, including changing the streetscape surrounding the developments, providing improved aesthetics (e.g. trees, grass) and walkability (e.g. sidewalks, street crossings) targeted for specific neighborhoods. Renovation of current greenspace, including multiple parks, six outdoor stairwells, and three trails connecting parks **vs** Fewer investments, exclusively related to housing	Visitors to study parks in low-income urban neighbourhoods	Physical activity Body weight and related measures Neighborhood satisfaction Neighborhood safety
Slater 2016 [54]	CBA study	USA	Park renovation (including replacing old playground equipment and ground surfacing and community engagement) **vs** no renovations and no community engagement	Adults visiting the selected parks	Physical activity Neighborhood safety
Tester 2009 [52]	CBA study	USA	Park renovations (artificial turf, new fencing, landscaping, lighting, and picnic benches were added. In Park A, permanent soccer goals were installed, and in Park B, a walkway around the field was restored) **vs** no intervention	Visitors to study parks	Physical activity
Veitch 2018 [43]	Cluster RCT	Australia	Park refurbishment **vs** no infrastructure changes	Visitor to study parks	Physical activity
Ward Thompson 2019 [49]	CBA study	Scotland	Physical changes to the woodland environment to facilitate access to and use of the woods **vs** no intervention	Adults living in communities at specific distances classified as in the lowest 30% of deprivation	Physical activity

All but one [44] of the included studies reported multiple measures of physical activity, including the proportion of participants meeting physical activity guidelines, time spent engaged in moderate to vigorous physical activity (MVPA) and leisure-time spent walking or cycling, among others. As there is no gold standard for measuring physical activity, we have reported all measures in this review. Four studies reported on body weight and related measures, specifically BMI and the proportion overweight or obese [44, 50, 55, 61]. One study assessed blood pressure [39]. None of the other primary outcomes were reported. Regarding secondary outcomes, two studies reported on satisfaction [50, 59], four on safety [50, 53, 54, 71], and one on adverse events [71].

#### Studies not included in the synthesis

Seven studies assessed the effect of multicomponent interventions on physical activity and health; three were cluster RCTs [73–75], three were CBA studies [76–78] and one was an ITS study [79] (Additional File 4).

Four studies were conducted in HICs [74, 76, 77, 79] and three in middle-income countries [73, 75, 78]. One study included children between 7 and 11 years of age in primary schools [75], one included adolescents between 11 and 14 years of age [77], and five studies included adults, one of them targeting adults > 65 years of age [76].

All studies assessed the effects of infrastructure interventions, including improvements to available green space, urban pedestrian circuits, footpaths, cycle tracks, playgrounds, sport facilities, or creating green space. One of the studies also assessed a policy and regulatory intervention – directives on allowing time to exercise at the workplace [73]. All studies included co-interventions, mostly of an educational nature such as through campaigns and community engagement programmes.

Three studies reported on measures of population-level physical activity, such as physical activity scores, frequency of physical activity, use of active school transport. One study reported on body weight and related measures (i.e., BMI) [75]. Four studies reported on secondary outcomes of interest, including changes in quality of life, and perceived health.

#### Studies ongoing, and awaiting classification

Eight studies were classified as ongoing, which are described in Additional file 5.

Seven studies were marked as awaiting assessment as they were conference papers or their full-texts could not be accessed. They are described in Additional file 6.

### Risk of bias in included studies

**Fig. 3:**
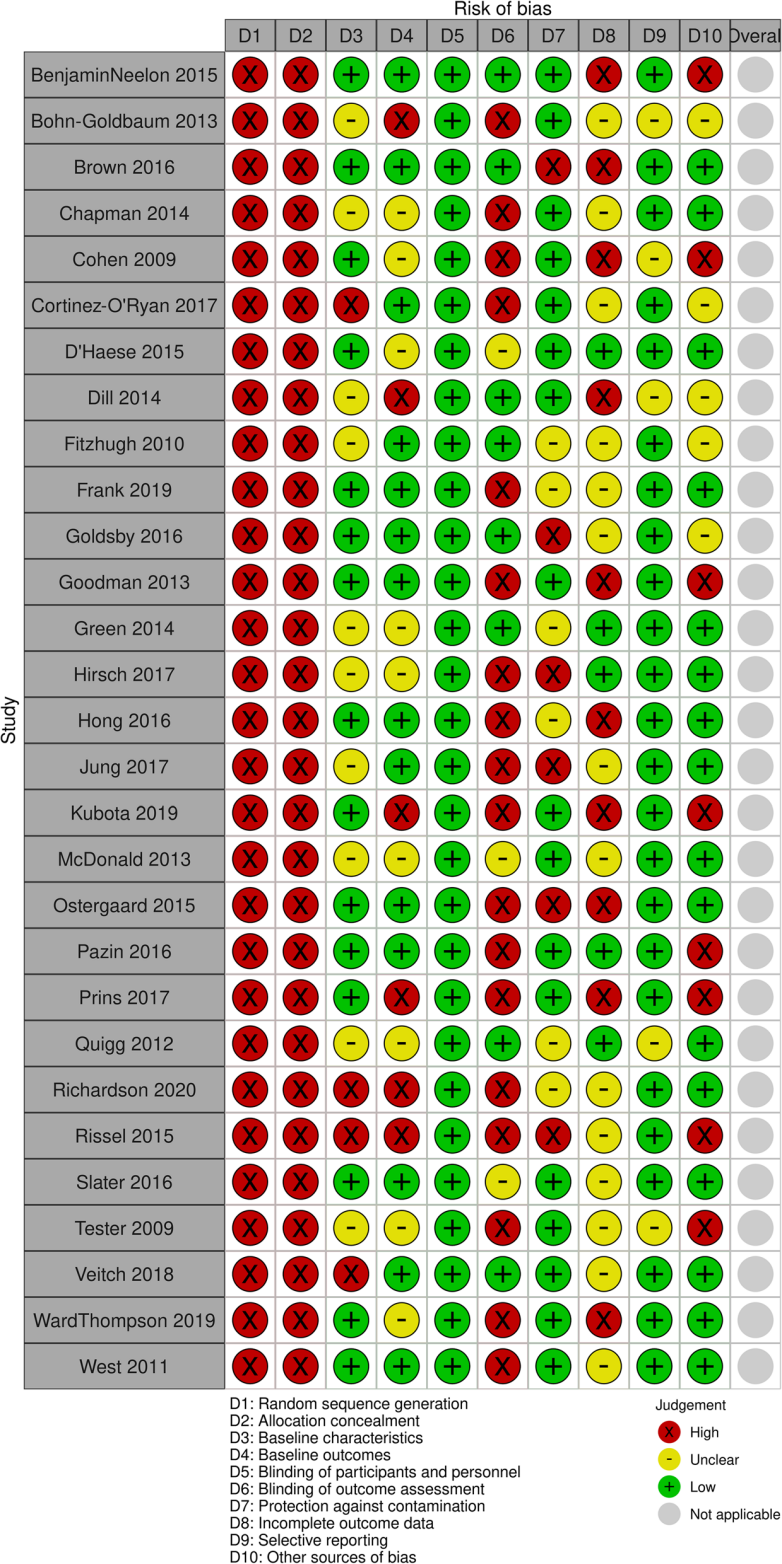
Summary of risk of bias assessments of trials and CBA studies

Most studies with a comparison group (*n* = 29) were at high risk of selection bias due to lack of randomization (Fig. 3). Regarding similarity in baseline characteristics and outcomes, most studies were at low risk of selection bias (*n* = 15 and 14, respectively) as there was no baseline imbalance, as any baseline differences were adjusted for in the analysis, as they were at unclear risk of selection bias (*n* = 10 and 9, respectively), or as they did not report sufficient information. All studies were at low risk of performance bias; although blinding of participants and personnel in these studies is generally not possible, due to the ecological nature of the interventions, performance bias is unlikely to meaningfully influence effects. Most studies (*n* = 18) were at high risk of detection bias as blinding was not possible or not reported and the outcomes were self-reported and thus more prone to influence from lack of blinding. Regarding protection against contamination, most studies (*n* = 19) were at low risk of bias as sites were different geographic areas and thus contamination was unlikely. Most studies were either at unclear (*n* = 14) or high (*n* = 10) risk of attrition bias; the latter due to reporting high levels of attrition (> 10%) or very low response rates, which differed between study groups. Most studies (*n* = 24) were at low risk of reporting bias and of other potential sources of bias (*n* = 16).

**Fig. 4:**
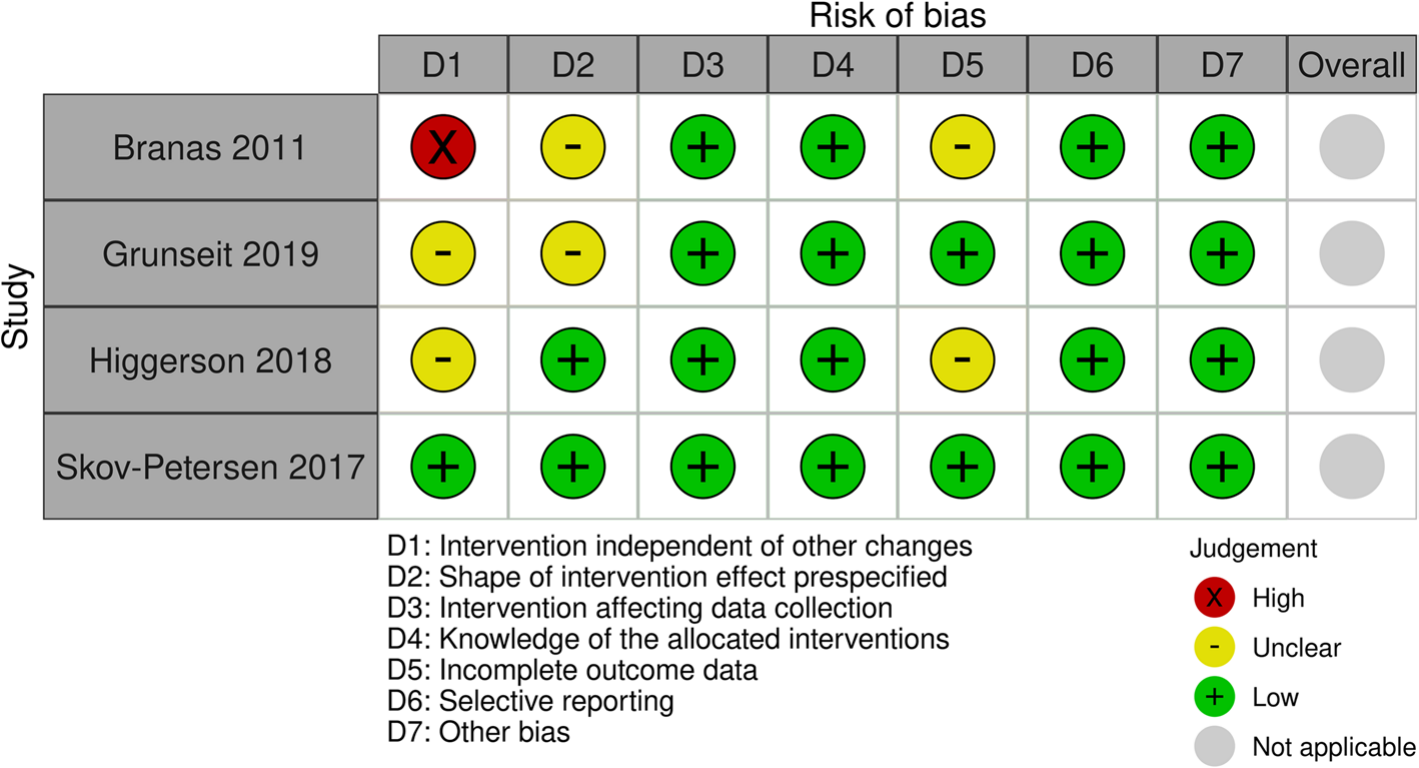
Summary of risk of bias assessments for ITS studies

Regarding the ITS studies (*n* = 4), one was at high risk of bias due to confounding with a high likelihood of factors outside of the intervention influencing the outcome [39] (Fig. 4), one [42] was at low risk of bias and two [40, 41] were at unclear risk of bias. Two studies [41, 42] were at low risk of bias in the classification of the intervention as the point of analysis was the point of the intervention, and two studies [39, 40] were at unclear risk of bias. All four studies were at low risk of bias in the measurement of the outcome; data collection was not influenced by the intervention and was collected in the same way before and after the intervention. All four studies were at low risk of detection bias; the outcomes assessed were objective and collected using routinely collected data or automatic counters. Two studies [40, 42] were at low risk of attrition bias; the data was collected using automatic counters and thus missing data was unlikely or no missing data was reported. The other two studies [39, 41] were at unclear risk of attrition bias. All studies were judged at low risk of bias from selective reporting and from other bias; all relevant methods and outcomes were reported and no other bias was identified.

A more detailed description of the risk of bias assessment is available in Additional file 7.

## Effects of interventions

The results of all individual studies are presented in Additional file 7, with highlighted rows indicating the outcomes selected for the synthesis.

### Interventions addressing infrastructure

#### Green or other spaces compared to no intervention

Thirteen studies—one cluster RCT, and 12 CBA studies—assessed the effects of introducing or upgrading green or other public spaces (Table 2). The interventions comprised closing streets for a specified period to create an environment for children to play [45, 46], creating new or upgrading existing parks or playgrounds [43, 44, 47, 51–54], physical environment changes to woodlands [49], neighbourhood development including infrastructure changes [50], treating or greening vacant lots [39], and building of a new exercise facility [48].

##### Primary outcome: Physical activity

**Fig. 5:**
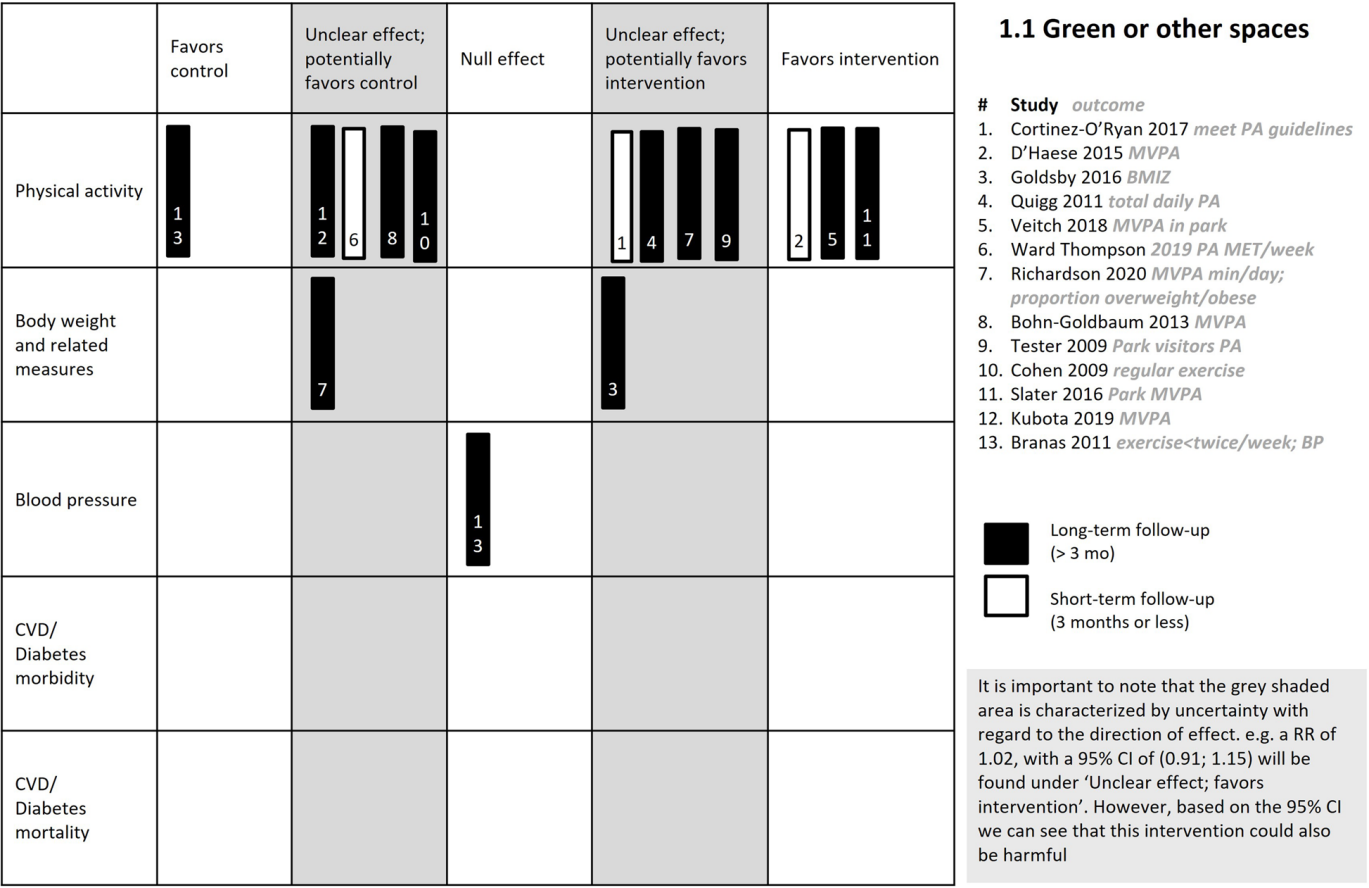
Harvest plot for comparison 1.1: Green or other spaces

We are very uncertain about the effects of interventions to green or other spaces on physical activity (12 studies, *very low certainty evidence*, Table 3). As these were observational studies the certainty of the evidence started at low, and it was further downgraded due to inconsistency and imprecision. The effects varied across the 12 studies (Table 4, Fig. 5).

**Table 3:** Summary of findings table for comparison 1.1—Changes in green or other spaces

**Population**: Children and adults living in study communities **Setting**: Communities and neighbourhoods in high-income countries **Intervention**: Changes in green or other spaces such as renovating or building playgrounds or parks, implementing playstreets, greening vacant lots or building multipurpose exercise facilities, to increase physical activity **Comparison**: control (no intervention or distance from intervention site)			
**Outcomes**	**№ of participants** **(studies)** **Follow-up**	**Certainty of the evidence (GRADE)**	**Impact**
Physical activity assessed with: MVPA, meeting PA guidelines, TDPA, time walking, cycling or taking part in sports follow-up: range 1 weeks to 3.5 years	(12 observational studies)	⨁◯◯◯ Very low^a,b^	A range of effects reported across 12 studies: clear effect favouring the control in one study, unclear effect potentially favouring the control in four studies, unclear effect potentially favouring the intervention in four studies, and a clear effect favouring the intervention in three studies
CVD mortality—not reported			
Diabetes mortality—not reported			
CVD morbidity—not reported			
Diabetes morbidity—not reported			
Body weight assessed with: BMI z-scores follow-up: 16 months	(2 observational studies)	⨁◯◯◯ Very low^b^	One study reported an unclear effect potentially favouring the intervention in children (Goldsby 2016 [44]) and the other an unclear effect potentially favouring the control in all ages (Richardson 2020 [50])
Blood pressure assessed with: self-report	(1 observational study)	⨁◯◯◯ Very low^c^	One CBA study (Branas 2011 [39]) indicates no effect of an intervention where vacant lots are greened to create a park-like setting) regression coefficient 0.63, 95% CI 0.32 to 0.94)

Explanations

^a^Downgraded by 1 due to inconsistency: effect direction varied across included studies

^b^Downgraded by 1 due to imprecision: most studies' results fall into an unclear effect category because of wide confidence intervals which include both beneficial and harmful effects

^c^Downgraded by 1 due to risk of bias: outcome was self-reported

**Table 4:** Results of studies included in comparison 1: Green or other spaces

Study ID Study design (Country)	Comparison	Participants at baseline (n)*	Outcome measure reported	Intervention		Control		Effect measure reported	Effe ct direction	Time of outcome measure
				Baseline value	Follow-up	Baseline value	Follow-up			
**Primary outcome: Physical activity**										
**Short term effects (< 3 months)**										
1. D’Haese 2015 [46] CBA study (Belgium)	Play streets vs no intervention	167 children (Playstreet: 71; control: 96)	MVPA (minutes/day)	54.92 (24.94)	67.05 (38.00)	57.41 (33.68)	" 52.87 (27.98)	Regression coefficient, 0.854, 95% CI: 0.204 to 1.504, SE = 0.332, p-value = 0.01	▲	1 week
2. Ward Thompson 2019 [49] CBA study (Scotland)	Physical changes to the woodland environment to facilitate access to and use of the woods vs no intervention	Cross-sectional sample of 5460 participants (wave 1, *n* = 2117; wave 2, *n* = 1672)	Overall PA (MET-minutes per week)	NR	NR	NR	NR	b = –282.4 95% CI –732.1 to 167.3	▽	2 months
**Medium-term effects (3–6 months)**										
3. Cortinez O'Ryan 2017 [45] (CBA) (Chile)	Neighbourhood with street closed for play vs control neighbourhood	100 children (intervention neighbourhood: 51, control neighbourhoods: 49)	Meeting pedometer-derived physical activity guidelines	27.5%	52.8%;	49%	53%	Change in IG: 25.3%; Change in CG: 4.0%; Between group comparison: p > 0.05 *Significant increase in intervention sites and non-significant increase in control sites*	△	3 months
**Long-term effects (> 6 months)**										
4. Bohn-Goldbaum 2013 [51] CBA study (Australia)	Upgrade of playgrounds in a park **vs** Parks not renovated/with similar pre-renovation playgrounds as intervention park	NR	MVPA (mean number of children engaged in MVPA per 2-h observation period)	mean (SD?) 1.17 (2.21)	mean (SD?) 0.67 (1.18)	mean (SD?) 2.86 (3.95)	mean (SD?) 1.98 (3.03)	*“After the park upgrade, there was no detectable difference between parks in the number of children engaged in MVPA (interaction between park and time: P* = *0.73); the proportion of physically active children had decreased by 41% at the intervention playground and by 32% at the comparison playground”*	▽	9 months
5. Slater 2016 [54] CBA study (USA)	Park renovation (which involved replacing old playground equipment and ground surfacing and community engagement) **vs** no renovations and no community engagement	Intervention – 39 parks; Control – 39 parks	Park-based MVPA (mean number of people observed per day)	Mean (SD) 17.07 (21.87)	Mean (SD) 24.95 (23.93)"	Mean (SD) 12.33 (19.59)"	Mean (SD) 15.33 (20.44)"	beta = 0.199 SE = 0.089 *p* < 0.10 95% CI [calculated] 0.02456 to 0.37344	▲	1 year
6. Quigg 2012 [47] CBA study (New Zealand)	Playground upgrade vs no intervention	184 children (intervention: 96; control: 88)	Total daily PA (total daily accelerometer counts/child day)	NR	NR	NR	NR	Ratio of geometric means = 1.11; 95% CI 0.85 to 1.44, p-value = 0.456	△	12 months
7. Tester 2009 [52] CBA study (USA)	Park renovations (artificial turf, new fencing, landscaping, lighting, and picnic benches were added. In Park A, permanent soccer goals were installed, and in Park B, a walkway around the field was restored) **vs** no intervention	523 people observed in intervention parks; 483 people observed in control park (children, teens, adult males/females, seniors)	● Mean number of park visitors engaging in sedentary PA per observation period ● Mean number of park visitors engaging in moderate PA ● mean number of park visitors engaging in vigorous PA Note: Data only presented for each park and per gender separately	NR	NR	NR	NR	"*There were statistically significant increases among males and females who were observed at each respective PA level in the intervention parks. Sedentary visitors increased 5* + *fold, moderately active visitors increased 3* + *fold, and vigorously active visitors increased 2* + *fold* ( Table 3)*. On the control playfield, only moderately active males increased*"	△	1 year
8. Cohen 2009 [53] CBA study (USA)	Park improvements ( e.g. new or refurbished gymnasiums, field improvements in watering and landscaping; improvements to picnic areas, upgrades to a walking path, and enhancements to a play-ground area..” **vs** No intervention	10 parks: 5 intervention and 5 comparison parks Two cross-sectional samples of park users interviewed: 768 at baseline and 712 at follow-up	Proportion exercising regularly [reporting exercising at least three times per Week]	0.616	0.419	0.667	0.482	Ratio of OR: 0.99 *p* = 0.812	▽	Approximately 1 year
9. Veitch 2018 [43] (cRCT) (Australia)	Park refurbishment vs no infrastructure changes	Total visitor counts: 4756 ( intervention park: 2374; control park: 2382	Proportion engaging in MVPA at the park	n (%) 789 (33.2)	n (%) 907 (28.7)	n (%) 1028 (43.2)	n (%) 583 (35.2)	IRR 2.28 (95% CI 1.19 to 4.38, *p* = 0.013)	▲	2 years
10. Kubota 2019 [48] CBA study (Japan)	Construction of a new multipurpose exercise facility including indoor facilities (25 m pool, 170 m walking trail, multi-purpose gym, and group exercise rooms) and outdoor facilities (multi-purpose athletic field, 875 m walking trail, and park), accessible to all residents for a small fee + PA promotion **vs** No new exercise facility or PA promotion but with routine health promotion program	Intervention: 1107 adults Control: 1125 adults	Percentage engaging in MVPA	N (%) 821 (42.6)	N (%)1018 (39.6)	N (%) 845 (44.5)	N (%)924 (43.3)	OR 0.96 95% CI (0.84, 1.09) *p* = 0.51	▽	2 years
11. Richardson 2020 [50] CBA study (USA)	Public housing development and greenspace landscaping, including changing the streetscape surrounding the developments, providing improved aesthetics (e.g. trees, grass) and walkability (e.g. sidewalks, street crossings). Renovation of current greenspace, including multiple parks, six outdoor stairwells, and three trails connecting parks **vs** Fewer investments, exclusively related to housing	17 parks (8 intervention, 9 control) Participants: 673 in intervention, 330 in control	MVPA (minutes /day) (Dubowitz 2019) [accelerometer data]	Mean (SE) 6.89 (0.90)	Mean 6.06	Mean (SE) 6.18 (1.22)	Mean 5.12	DID: 0.24 *p* = 0.813	△	3 years
12. Branas 2011 [39] CBA study (USA)	Greening of abandoned vacant lots (involved removing trash and debris, grading the land, planting grass and trees to create a park-like setting, and installing low wooden post-and-rail fences around each lot’s perimeter **vs** No greening of vacant lots	"Greened vacant lots (intervention)—*n* = 4,436 Control vacant lots—*n* = 13,308"	Low Exercise (proportion responding < 2 times/week)	NR	NR	NR	NR	Beta = 0.25 SE = 0.12 95% CI [calculated]: 0.0148 to 0.4852	▼	10 years
**Primary outcome: Body weight and related measures**										
1. Goldsby 2016 [44] CBA (USA)	living in close proximity (near) to new inner-city park (within 1.5 miles) vs living farther away from the park (further than 5 miles)	1443 children 2 to 17.9 years old (intervention – “near”: 45, control – “far”: 935)	BMI z-score change (for all children and subgroups: overweight/obese vs normal weight at baseline)	0.61(1.00)	0.66(1.09)	0.83(1.09)	0.87(1.11)	Regression coefficient = -0.0033, 95% CI: -0.115 to 0.109, SE = 0.0572, p-value = 0.4804	△	16 months
2. Richardson 2020 [50] CBA (USA)	Public housing development and greenspace landscaping, including changing the streetscape surrounding the developments, providing improved aesthetics (e.g. trees, grass) and walkability (e.g. sidewalks, street crossings). Renovation of current greenspace, including multiple parks, six outdoor stairwells, and three trails connecting parks **vs** Fewer investments, exclusively related to housing	17 parks (8 intervention, 9 control) Participants: 673 in intervention, 330 in control	Proportion overweight or obese (BMI > 25 kg/m^2^)	79.46%	77.11%	79.30%	75.52%	DiD = 1.43 *p* = 0.568	▽	3 years
**Primary outcome: Blood pressure**										
1. Branas 2011 [39] ITS	Greening of abandoned vacant lots (involved removing trash and debris, grading the land, planting grass and trees to create a park-like setting, and installing low wooden post-and-rail fences around each lot’s perimeter vs No greening of vacant lots	"Greened vacant lots (intervention)—*n* = 4,436 Control vacant lots—*n* = 13,308"	Proportion self-reporting high BP	NR	NR	NR	NR	Beta = 0.63 SE = 0.16 95% CI 0.32 to 0.94 [calculated]	▼	10 years
**Secondary outcome: Satisfaction**										
1. Richardson 2020 [50] CBA (USA)	Public housing development and greenspace landscaping, including changing the streetscape surrounding the developments, providing improved aesthetics (e.g. trees, grass) and walkability (e.g. sidewalks, street crossings). Renovation of current greenspace, including multiple parks, six outdoor stairwells, and three trails connecting parks **vs** Fewer investments, exclusively related to housing	17 parks (8 intervention, 9 control) Participants: 673 in intervention, 330 in control	Neighborhood satisfaction	69.49%	73.38%	42.64%	52.42%	DiD estimator: -5.89% p-value: 0.342	▽	
**Secondary Outcome: Safety issues**										
1. Slater 2016 [54] CBA (USA)	Park renovation (including replacing old playground equipment and ground surfacing, and community engagement) **vs** no renovations or community engagement	Intervention – 39 parks; Control – 39 parks	Neighborhood safety (crime count)	mean (sd) 747.89 (904.68)	Mean (sd) 622.58 (721.28)	Mean (sd) = 579.41 (385.11)	mean (sd) 498.90 (297.18)	NR Crime count reduced in both groups but slightly more in the intervention group	△	1 year
2. Cohen 2009 [53] CBA (USA)	Park improvements ( e.g. new or refurbished gymnasiums, field improvements in watering and landscaping; improvements to picnic areas, upgrades to a walking path, and enhancements to a play-ground area..” **vs** No intervention	10 parks: 5 intervention and 5 comparison parks Two cross-sectional samples of park users interviewed: 768 at baseline and 712 at follow-up	Proportion reporting Perceived park safety	0.696	0.913	0.860	0.774	Ratio of ORs = 1.35 p < 0.001	▲	3–14 months
3. Richardson 2020 [50] CBA (USA)	Public housing development and greenspace landscaping, including changing the streetscape surrounding the developments, providing improved aesthetics (e.g. trees, grass) and walkability (e.g. sidewalks, street crossings). Renovation of current greenspace, including multiple parks, six outdoor stairwells, and three trails connecting parks **vs** Fewer investments, exclusively related to housing	17 parks (8 intervention, 9 control) Participants: 673 in intervention, 330 in control	Perceived neighbourhood safety	3.03% (SE 0.03)	3.18%	2.55% (SE 0.06)	2.78%	DiD estimator = -0.08 p-value = 0.280	▽	3 years

^*^Where provided, we report the number of participants in the intervention group and control group, separately. Where this is not provided, we report the total sample. Where the number of participants is not reported in the study, we could not provide it here

##### Primary outcome: body weight and related measures

We are very uncertain about the effect of interventions on green or other spaces on body weight (2 studies, *very low certainty evidence*). The certainty of the evidence started at low as these were observational studies and was downgraded further due to imprecision. Both studies reported unclear effects, one potentially favouring the intervention, at 16 months [44] and the other, the control, at 3 years [50]. Goldsby 2016 [44] assessed children living near vs far new inner-city parks whereas Richardson 2020 [50] assessed visits to parks in low-income neighbourhoods.

##### Primary outcome: blood pressure

One ITS study on greening vacant lots to create a park-like setting [39] reported a clear effect favouring the control on blood pressure (regression coefficient 0.63, 95% CI 0.32 to 0.94), however the certainty of the evidence was *very low*. The study started at low certainty, and it was further downgraded due to risk of bias.

##### Secondary outcome: satisfaction

One study reported an unclear effect potentially favouring the control on participant satisfaction with their neighbourhood after a public housing and greenspace landscaping intervention [50]. The proportion of participants that reported being satisfied with their neighbourhood increased in both the intervention and control neighbourhoods, but it increased more in the control neighbourhoods (DiD estimator -5.89%, p-value 0.342, *n* = 1003 participants).

##### Secondary outcome: safety issues

Three CBA studies reported this outcome; one showed a clear effect favouring the intervention on the proportion of participants reporting perceived park safety [53], one an unclear effect potentially favouring the intervention on crime counts in the neighbourhood [54], and one an unclear effect potentially favouring the control on the proportion of people reporting perceived neighbourhood safety [50].

#### Active transport interventions compared to no intervention

Eighteen studies – 15 CBA studies and three ITS studies—assessed the effects of environmental changes to promote active transport and thus physical activity (Table 5). These comprised street improvements such as adding bike lanes, sidewalks, or crosswalks, and road surfacing, among others [55, 59–62, 70]; building or improving bicycle boulevards, greenways and cycleways [40, 42, 56–58, 64, 65, 67–69]; and building a light rail line or a motorway to divert traffic and free up space for pedestrians and cyclists [63, 66]. Some of these environmental changes were embedded within larger initiatives and included other intervention components; for example, Goodman 2013 [56] assessed the ‘Cycling Cities and Towns’ initiative, which comprised a range of changes to make communities more cycling-friendly.

**Table 5:** Characteristics of studies included in the Active transport comparison

Study ID	Study design (ROB)	Country	Intervention and comparison description	Participants	Outcomes reported
1. Brown 2016 [60]	CBA	USA	Street improvements including new bike lanes, wider and better lit sidewalks. Participants living near (within 800 m) the intervention street were compared with those living farther away	Adults who planned to stay in the neighbourhood for at least a year	Physical activity
2. Benjamin Neelon 2015 [61]	CBA	USA	Built environment changes including new sidewalks and crosswalks Additional components: walking/running clubs in the elementary schools and in the community and provision of portable play equipment Compared to no intervention	Children attending elementary school in study communities, and their adult parents	Physical activity; Body weight and related measures
3. Chapman 2014 [70]	CBA	New Zealand	Infrastructure upgrading e.g., footpath renewal, new tracks, new cycle paths, lighting, bike stands, shared space or pathway projects, etc Compared to no intervention	Occupants 10 years and older in households randomly selected	Physical activity
4. Dill 2014 [65]	CBA	USA	Installation of bicycle boulevards to reduce the speed and volume of motor vehicles and create a better environment for people on bicycles, compared to no intervention	Children aged 5 to 17 and adult parent or guardian physically able to ride a bicycle, have access to a working bicycle, and not be intending to move in the near future	Physical activity
5. Frank 2019 [64]	CBA	Canada	Building of the Comox greenway, to improve conditions for bicyclists. The two-kilometre route consists of a mix of cycling facilities and other streetscape improvements Proximity to infrastructure changes was compared to those farther away	Residents in study communities with no plans to move during the study period	Physical activity
6. Fitzhugh 2010 [57]	CBA	USA	Building of an urban greenway/trail to connect the pedestrian infrastructure with nearby retail establishments and schools Compared to no intervention	Individuals observed in study neighbourhoods	Physical activity
7. Goodman 2013 [56]	CBA	UK	Connect2 Initiative: one flagship engineering project and improvements to cycle routes. One in Cardiff, where a traffic-free bridge was built over Cardiff Bay; Kenilworth, where a traffic-free bridge was built over a busy trunk road; and Southampton, where an informal riverside footpath was turned into a boardwalk. Towns implemented educational and promotional activities as well	Adults 18 years and older living within 5 kms of the projects	Physical activity
8. Hong 2016 [66]	CBA	USA	Building of new light rail line Those residing < ½ mile to the new infrastructure compared to those residing farther away (> ½ mile)	Households interested to participate in study	Physical activity
9. Hirsch 2017 [67]	CBA	USA	Additions of the Hiawatha Trail (4.7 miles) and Midtown Greenway (5.5 miles); these provide 10.2 miles of off-road paved paths, including a dedicated bicycle/pedestrian bridge over a busy freeway Comparison: Before vs after changes for those near (25^th^ percentile/1.08 km) the infrastructure	Census data used	Physical activity
10. Jung 2017 [59]	CBA	Korea	Design street project (including the improvement of sidewalks, public spaces, signs, fences, and other physical elements of the streets) **vs** typical street	2016 + 15,686 responses	Satisfaction
11. McDonald 2013 [62]	CBA	USA	Safe Routes to School (SRTS) programme: includes arms with education + covered bike parking, and with education + Sidewalks/crosswalks Compared to schools with no SRTS programme	Schools (classroom and parent surveys)	Physical activity
12. Pazin 2016 [69]	CBA	Brazil	New avenue, parking lots, and an on-road walking and cycling route, built along the seashore Those living nearer (0-500 m) to the new infrastructure were compared to those farther away (501–1000)	Adults 18 years and older residing in study area	Physical activity
13. Prins 2017 [63]	CBA	Scotland	Construction of a new 5-mile, six-lane section of motorway, to relieve through traffic on an existing urban motorway, promote economic regeneration, and remove traffic from local streets to create a more pedestrian- and cycle-friendly environment	Adults ≥ 16 years residing in study areas	Physical activity; Mental health
14. Rissel 2015 [58]	CBA	Australia	New cycling infrastructure of 2.4 km length built by the City of Sydney as part of its expanding bicycle network Compared to no intervention	Individuals 18–55 years living in geographic proximity to study areas	Physical activity
15. Østergaard 2015 [55]	CBA	Denmark	Physical environment changes in schools to increase cycling, including road surfacing, signposting and traffic regulation such as one-way streets and regulation of car drop off zones, plus 'soft' interventions (motivation and safety encouragement) Compared to no intervention	Public school children in 4^th^ and 5^th^ grade	Physical activity, Body weight and related measures; Adverse events (injuries)
16. West 2011 [68]	CBA	USA	Building of a new greenway for recreational use Those living near the greenway (within .5 miles) were compared to those living far (within .51–1.0 miles)	Property owners living within 1 mile of the greenway	Physical activity
17. Skov-Petersen 2017 [42]	ITS	Denmark	Improvements to two routes: cycle greenway (Vestvolden) and a cycle highway (Albertslund Route), including new surface and light conditions along a substantial part of the routes. Additional components: roller skate tracks, and a range of information activities including the establishment of an information centre, the installation of signs, and the publication of leaflets, audio guides, etc	Counts of participants using the routes	Physical activity
18. Grunseit 2019 [40]	ITS	Australia	Construction of multi-use recreational walking and cycling loop trail	Data from two infrared pyroelectric counters on the trails	Physical activity

##### Primary outcome: physical activity

**Fig. 6:**
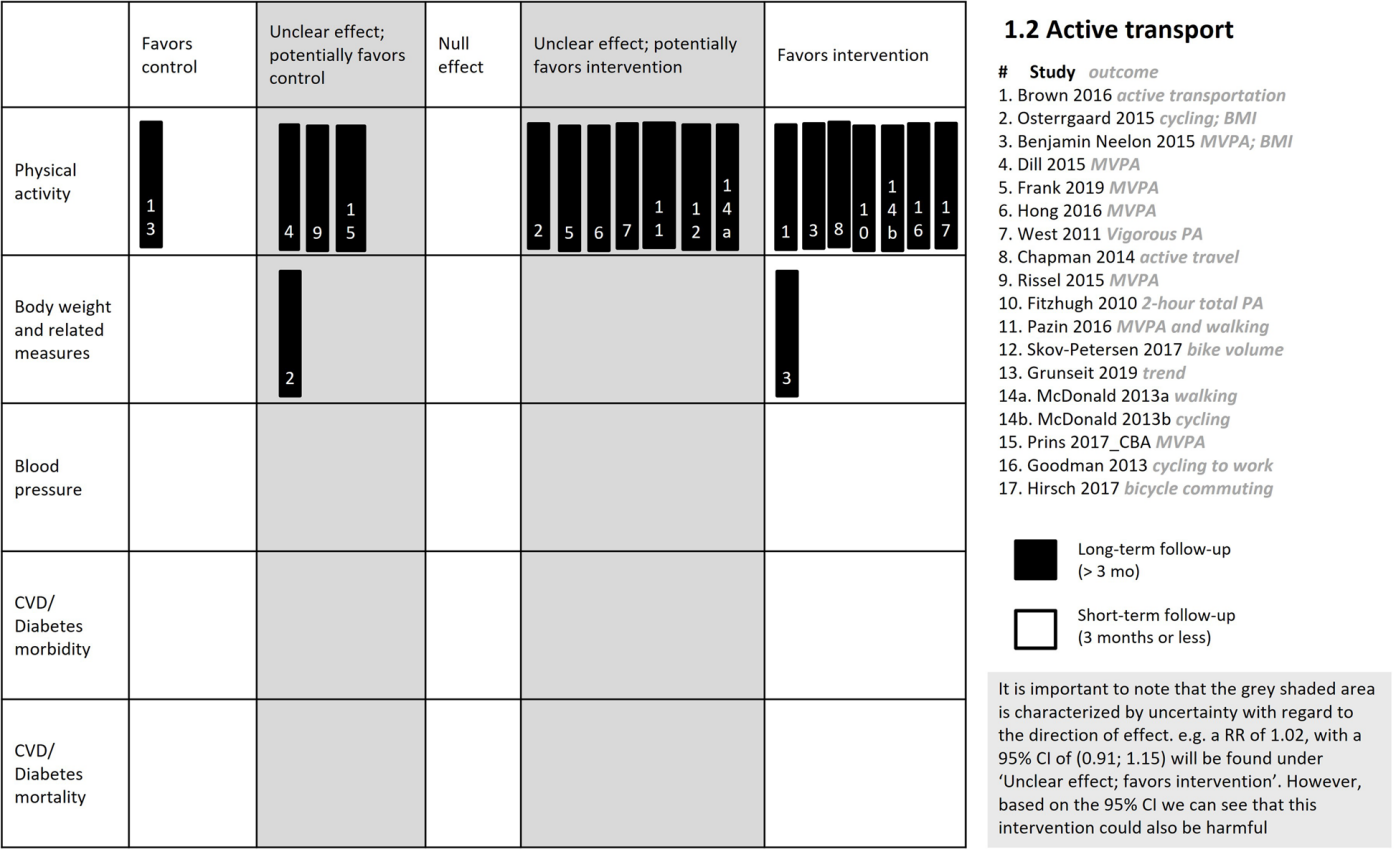
Harvest plot for comparison 1.2 Active transport interventions

The effects of active transport interventions on physical activity are very uncertain (17 studies, *very low certainty evidence*, Table 6). The certainty of the evidence started at low and was downgraded further due to imprecision and risk of bias. Of the 17 studies, seven studies reported a clear effect favouring the intervention, six reported an unclear effect potentially favouring the intervention, three reported an unclear effect potentially favouring the control, and one reported a clear effect favouring the control (Table 7, Fig. 6). Most of the studies that showed a clear effect included additional intervention components such as education and promotion to use the newly built infrastructure, whereas the studies with unclear and clear effects favouring the control did not include these.

**Table 6:** Summary of findings table for comparison 1.2: Active transport interventions

**Patient or population:** Children and adults, both living in the community as well as those travelling to and from school and work, respectively **Setting:** Communities and neighbourhoods in HICs **Intervention:** Creating or upgrading sidewalks, crosswalks, walking, cycling and running paths, light rail routes (e.g. street cars, trams), improvement of the near-school cycling and walking environment, or a motorway **Comparison:** no new intervention			
**Outcomes**	**№ of studies**	**Certainty of the evidence** **(GRADE)**	**Impact**
Physical activity assessed with: proportion/time cycling, biking, walking, MVPA, transit related active trips follow-up: 1 year to 10 years	16 CBA studies, 1 ITS	⊕ ⊝ ⊝ ⊝ VERY LOW ^a, b^	Seven studies reported a clear effect favouring the intervention, six studies reported an unclear effect potentially favouring the intervention, three studies reported an unclear effect potentially favouring the control, and one study reported a clear effect favouring the control
Body weight assessed with: BMI follow up: 12 months	2 CBA studies	⊕ ⊝ ⊝ ⊝ VERY LOW^a^	One study observed a clear effect favouring the intervention and one study observed an unclear effect potentially favouring the control
Blood pressure	0 studies	-	Not reported
CVD morbidity	0 studies	-	Not reported
Diabetes morbidity	0 studies	-	Not reported
CVD mortality	0 studies	-	Not reported
Diabetes mortality	0 studies	-	Not reported

*CI* Confidence interval, *HICs* high-income countries, *RR* Risk ratio, *OR* Odds ratio, *MVPA* moderate to vigorous physical activity, *CBA* controlled before-after

Explanation

^a^Downgraded by 1 for risk of bias: high risk across several domains in multiple studies; there is high potential for direction of effect to change

^b^Downgraded by 1 for imprecision: very wide confidence intervals in most studies

**Table 7:** Results of studies included in comparison 1.2: Active transport interventions

Study ID Study design (Country)	Comparison	Participants at baseline (n)	Outcome measure reported	Intervention		Control		Effect measure reported	Effect direction	Time of outcome measure
				**Baseline value**	**Follow-up**	**Baseline value**	**Follow-up**			
**Primary outcome: Physical activity**										
1. Brown 2016 [60] CBA (USA)	Participants living near (within 800 m) of the intervention street**.** those living far from intervention street Street improvements included new bike lanes, wider and better lit sidewalks	910 residents	Transit-related active transportation trips	proportion 0.21, SD = 0.41	pro*p* = 0.39	pro*p* = 0.15, SD = 0.35	0.25	OR = 1.48; 95% CI: 1.14 to 1.68, *p* = 0.01	▲	1 year
2. Østergaard 2015 [55] CBA (Denmark)	Physical environment changes plus 'soft' interventions (motivation and safety encouragement) **vs**. no intervention Interventions to increase cycling: structural changes near the school in e.g. road surface, signposting and traffic regulation such as one-way streets and regulation of car drop off zones"		PA from cycling (number of trips to and from school in previous week)	mean (SD) 5.8 (4.4)	NR	mean (SD) 6.4 (4.3)	NR	Change beta coefficient: 0.15; 95% CI: -0.25 to 0.54; p-value = 0.463	△	1 year
3. Benjamin Neelon 2015 [61] CBA (USA)	Built environment changes including new sidewalks and crosswalks **vs.** no intervention	Intervention – 64 children; Control – 40 children	MVPA (min/hr)	Mean (SD 4.0 (1.7)	Mean (SD) 4.2, (1.9)	Mean (SD) 3.8 (2.0)	Mean (SD) 3.4 (1.5)	Regression coefficient: 1.3; 95% CI: 0.2 to 2.3; p-value = 0.03	▲	1 year
4. Dill 2014 [65] CBA (USA)	Installation of bicycle boulevards **vs.** no installation	255 parents living at 8 intervention and 11 control street segments	Minutes of MVPA per day	Mean (SD) 39.5 (21.9)	Mean (SD) 35.6 (19.0)	Mean (SD) 35.4 (20.8)	Mean (SD) 34.8 (19.4)	beta-coefficient: − 3.44 *p*-value = 0.33	▽	1 year
5. Frank 2019 [64] CBA (Canada)	Proximity to infrastructure changes (greenway development); close **vs.** further away	Intervention – 219 residents; Control – 265 residents	MVPA (proportion engaging in > 20 min/day)	67.6% (95% CI 61.3, 73.8)	69.4% (95% CI = 63.3, 75.6	68.7% (95% CI 63.1, 74.3)	60.8% (95% CI = 54.8, 66.7)	OR 2.00 95% CI: 1.00 to 3.98	△	1 year?
6. Hong 2016 [66] CBA (USA)	New light rail line; treatment group (residing < ½ mile) **vs.** control group (> ½ mile)	Intervention – 32 residents; Control – 41 residents of an urban area	Average minutes of daily MVPA	Mean (SD) 23.09 (17.49)	Mean (SD) 21.52 (16.24)	Mean (SD) 19.81 (18.01)	Mean (SD) 18.56 (17.02)	Coefficient = -0.34 *p* = 0.063*	△	1 year
7. West 2011 [68] CBA (USA)	Living near (within .5 miles) **vs.** far (within .51–1.0 miles) to new greenway construction	Intervention – 93 residents; Control – 73 residents	moderate PA (number of days/week)	Mean (SD) 1.76 (1.99)	Mean (SD) 2.39 (1.93) 0.63	mea*n* = 1.63; SD = 1.81	mea*n* = 2.11; SD = 1.91	"Wilks’s Lambda = .997, F(1, 165) = . 509", *P* < 0.476	△	1 year
8. Chapman 2014 [70] CBA New Zealand	"the introduction of cycle and walkway infrastructure, along with measures to encourage active travel" **vs.** no intervention	4861 trips	Proportion engaged in active travel	19.7% (*n* = 111) (unadjusted)	17.8% (*n* = 151) (unadjusted)	19.4% (*n* = 131) (unadjusted)	15.0% (*n* = 132) (unadjusted)	OR 1.37 (1.08 to 1.73)	▲	1 year
9. Rissel 2015 [58] CBA (Australia)	building cycling infrastructure **vs.** no intervention	Intervention – 398 adult residents; Control – 448 adult residents	MVPA (min/week)	Mean (SD) 239.5 (274.5); *n* = 398	Mean (SD) 204.0 (252.9); *n* = 189)"	Mean (SD) 211.1 (229.6); *n* = 448"	Mean (SD) 180.5 (197.6); *n* = 229"	DID = -4.9 [calculated]	▽	16 months
10. Fitzhugh 2010 [57] CBA (USA)	Building greenway/trail **vs.** no intervention	Intervention – 1 neighbourhood; Control – 2 neighbourhoods	2-h counts of total PA	Median: 4.5 (IQR: 3.0–6.0)	Median: 13.0 (IQR: 11.0–15.0)	Median: 3.0 (IQR: 0.0–8.0)	Median: 1.0 (IQR: 0.0—6.0)	NR, *p* = 0.001 “… the experimental neighborhoods’ change in physical activity was found to be significantly different from the control neighborhoods’ for pedestrian (*p* = 0.001); cycling (*p* = 0.038); and total physical activity (*p* = 0.001)”	**▲**	2 years
11. Pazin 2016 [69] CBA (Brazil)	living nearer (0-500 m) **vs.** farther away (501–1000) from new walking and cycling route	Intervention – 192 adults; Control – 137 adults from 6 urban neighborhoods	MVPA + walking in previous week (min/week)	Mean (95% CI): 107 (90 to 124)	Mean (95% CI): 158 (130 to 187)	Mean (95% CI): 149 (105 to 193)	Mean (95% CI): 128 (99 to 156)	NR Naïve DID = 72	△	3 years
12. Skov-Petersen 2017 [42] ITS (Denmark)	cycle highways (Albertslund) upgrade **vs.** no upgrade	50,954 counts	Bike volume (cyclists/hr)	NR	NR	NR	NR	Beta: 0.95 error: 0.89 *p* = 0.2858	△	Over 35 months
	cycle greenway upgrade (Vestvolden) **vs.** no upgrade			NR	NR	NR	NR	Beta: 3.15, error: 1.11, *p* = 0,0046	▲	
13. Grunseit 2019 [40] ITS (Australia)	Trends before and after the construction of multi-use recreational walking and cycling loop trail	All cyclists riding on two trails	Trail use (immediate effect): Counts of bike passes (at Jamieson park)	NR	NR	NR	NR	adj beta 189,995% CI 1672, 2126	▲	120 time points "19 weeks February 25th to July 14th (weeks 9 to 28) for each year 2013, 2014 and 2015"
			Trend in trail use: for bikes (Jamieson park) effect over time	NR	NR	NR	NR	adj beta -6295% CI -80 to -44	▼	
14. McDonald 2013 [62] CBA USA	Schools with SRTS* programme (education + covered bike parking) **vs.** schools with no SRTS programs	Intervention Schools—9; control schools—5	proportion biking	NR	NR	NR	NR	marginal effect: 0.106; 95% CI: 0.018 to 0.195	▲	5 years
	Schools with SRTS programme (education + Sidewalks/crosswalks) **vs.** schools with no SRTS programs		proportion walking	NR	NR	NR	NR	Marginal effect: 0.064; 95% CI: -0.002 to 0.130	△	
15. Prins 2017 [63]_CBA analysis	new motorway **vs.** no motorway	1412 adults from two urban areas	Proportion participation in MVPA	65.5% (*n* = 220)	71.9% (*n* = 231)	62% (*n* = 234)	68.5% (*n* = 254)	OR: 0.95 (95% CI: 0.53 to 1.72)	▽	8 years
16. Goodman 2013 [56] CBA (UK)	Town-level cycling initiative (infrastructure and health promotion) **vs**. Matched comparison	Intervention – 37 urban census areas; Control – 27 urban census areas	Proportion of commuters cycling to work	5.81%; (5.77; 5.86)	6.78%; (6.74; 6.83)	4.03% (3.99; 4.08)	4.32%; (4.28; 4.36)"	coefficient: 0.69; 95% CI: 0.60; 0.77	▲	10 years
17. Hirsch 2017 [67] CBA study (USA)	Before **vs**. after infrastructure changes for those near (25^th^ percentile/1.08 km) the infrastructure changes (construction of an off-road trail system)	116 census tracts (population differed at different timepoints)	Proportion commuting to work by bicycle	mean (sd)1.76% (1.96%)	mean (sd)4.04% (3.48%)	NR	NR	coefficient 2.03; 95% CI (0.13; 3.93)	▲	10 years
**Primary outcome: Body weight and related measures**										
1. Østergaard 2015 [55] CBA (Denmark)	Physical environment change plus 'soft' interventions (motivation and safety encouragement) **vs.** no intervention	1390 children	Change in BMI	Mean (SD) 18.24 (2.93)	NR	Mean (SD) 18.23 (2.84)	NR	beta coefficient: 0.01; 95% CI: (-0.13; 0.15); p-value: 0.887	▽	1 year
2. Benjamin Neelon 2015 [61] CBA (USA)	Active transport **vs.** no intervention	104 children; Intervention –64; Control – 40	BMI Z-score	Mean (SD) 0.6 (1.2)	NR	Mean (SD) 1.2 (1.2)	NR	Regression Coefficient: -0.5; 95% CI = -0.9 to -0.02; p-value = 0.045	▲	1 year
**Secondary outcome: Satisfaction**										
1. Jung 2017 [59] CBA	Design street project (including the improvement of sidewalks, public spaces, signs, fences, and other physical elements of the streets) **vs.** typical street	Intervention – 2016 pedestrians; Control – 15,686 pedestrians	Pedestrian satisfaction score	3.213	3.355	3.256	3.092	coefficient = 0.291; (SE = 1.31), p < 0.05	▲	3 years
**Secondary outcome: Adverse event—injuries**										
1. Østergaard 2015 [55] CBA	Physical environment change plus 'soft' interventions (motivation and safety encouragement) **vs.** no intervention	1684 children; Intervention –897; Control – 641)	Cycling injuries frequency	193	184	147	137	NR Naïve DID = 1	▽	1 year
**Secondary outcome: Adverse events—Mental health**										
1. Prins 2017 [63]_CBA analysis	Construction of a new motorway (also hypothesized to remove traffic from local streets and create a more pedestrian- and cycle-friendly environment) **vs.**no motorway	1778 adults from two urban areas	Mental well-being (MCS-8 score)	NR	NR	NR	NR	Regression coefficient: -0.8; 95% CI: –3.1 to 1.5	▽	8 years

^*^Where provided, we report the number of participants in the intervention group and control group, separately. Where this is not provided, we report the total sample. Where the number of participants is not reported in the study, we could not provide it here

##### Primary outcome: body weight and related measures

We are very uncertain about the effect of active transport interventions on BMI (two studies, *n* = 1494 participants, *very low certainty evidence, Table *6); the certainty of the evidence was downgraded due to risk of bias. The studies reported different effects; one a clear effect favouring the intervention [61] and the other an unclear effect potentially favouring the control [55] (Fig. 6).

##### Secondary outcome: satisfaction

One study assessed a pedestrian satisfaction score (ranging from 1 to 5) in relation to the ‘Design Street’ initiative, which involved the improvement of sidewalks, public spaces, and other environmental aspects of the pedestrian environment [59]. It reported a small yet clear increase of 0.291 points in satisfaction at intervention sites in comparison to control sites (DiD estimate 0.291, p < 0.05, Table 4).

##### Secondary outcome: adverse events – injuries

One study assessed whether the near-school cycling and walking environment influenced the number of injuries in school children [55]. It reported a small decrease in injuries at both the intervention and control sites, although this effect was slightly larger at control sites (193 to 184 children vs 147 to 137 children, Table 7).

##### Secondary outcome: adverse events—mental health

One CBA study assessed whether the introduction of a new motorway influenced mental health and well-being of area residents [63]. Mental well-being was assessed using the MCS-8 score (mental component summary of the Short Form 8 Health Survey, and higher scores represented higher well-being). It reported little or no difference in the mental health of residents at intervention sites compared to control sites after 8 years (coefficient -0.8 MCS-8 points, 95% CI -1.6 to 3, *n* = 1778 participants, Table 4).

### Policy and regulatory interventions

#### Access to PA facilities compared to no intervention

One ITS study in England assessed the effects of a policy that provided all individuals living in the intervention community with free access to government leisure facilities at most times of the day [41]. Some of the facilities included swimming pools and gyms.

##### Primary outcome: Physical activity

Free access to government leisure facilities may increase gym or swimming-related physical activity (1 ITS study, RR 1.64, 95% CI: 1.43 to 1.89, low certainty evidence, Table 8). The study was an ITS study, therefore the certainty of the evidence started at low; it was not downgraded.

**Table 8:** Results of studies included in comparison 2: Policy and regulatory interventions

Study ID Study design Country	Participants at baseline (n)*	Outcome measure reported	Intervention		Control		Effect measure reported	Effect direction	Time of outcome measure
			**Baseline value**	**Follow-up**	**Baseline value**	**Follow-up**			
Comparison 2.1 Free access to local government leisure facilities vs no access									
** Primary outcome: Physical activity**									
1. Higgerson 2018 [41]_ITS UK	Gym and swim attendees of a leisure center	Increase in activity (gym or swimming) (based on logged attendances)	NR	NR	NR	NR	RR = 1.64, 95% CI 1.43 to 1.89, *p* < 0.001	**▲**	7 years
Comparison 2.2 Free bus travel for 12–17-year-olds vs no free bus travel for 25–59 year olds									
** Primary outcome: Physical activity**									
Green 2014 [71] CBA London	Intervention group—4206; Control group—31,169	Number of walking trips	Proportion: 0.99	Proportion: 0.83	Proportion: 0.83	Proportion: 0.91	Ratio of ratios: 0.76 (95% CI 0.70 to 0.85)	**▼**	3 years
		Number of cycling trips	Proportion: 0.06	Proportion: 0.04	Proportion: 0.05	Proportion: 0.07	Ratio of ratios: 0.53 (95% CI 0.35 to 0.87)	**▼**	3 years
** Secondary outcome: Adverse events—Injuries**									
Green 2014 [71] CBA London	NR	Incidence of Road Traffic Injuries for all transport modes	5.46 per 1000 person years	3.23 per 1000 person years	5.81 per 1000 person years	4.08 per 1000 person years	Ratio of ratios 0.84 95% CI 0.82 to 0.87	**▲**	3 years
** Secondary outcome: Safety issues**									
Green 2014 [71] CBA London	NR	Rate of hospitalisation for injuries inflicted by assaults	1.13 admissions per 1000 person-years	1.61 admissions per 1000 person-years	0.77 admissions per 1000 person-years	0.91 admissions per 1000 person-years	Relative effect 19% 95% CI 16% to 22%	**▼**	3 years

^*^Where provided, we report the number of participants in the intervention group and control group, separately. Where this is not provided, we report the total sample. Where the number of participants is not reported in the study, we could not provide it here

#### Free bus travel compared to no intervention

One CBA study (Green 2014) in London assessed the effects of a policy providing free bus travel for individuals 12–17 years old compared to a population of adults 25–59 years old that did not have free bus travel on different physical activity measures.

##### Primary outcome: Physical activity

The evidence on the effects of the free bus travel policy for youth, which aimed to reduce car use and increase active travel, on physical activity was very uncertain (1 CBA study, very low certainty evidence). The included study (Green et al., 2014) [71] reported a clear effect favouring the control, i.e. a reduction in the proportion of walking (Ratio of ratios 0.76, 95% CI 0.70 to 0.85) and cycling trips (Ratio of ratios 0.53, 95% CI 0.35 to 0.87) among those in the intervention group compared to the control group (Table 8). The certainty of the evidence started at low and was downgraded due to indirectness as the main objective of the intervention was to reduce car use and the population was from an urban setting in the UK which may not be applicable to an LMIC population where the public transport system is very different.

##### Secondary outcome: safety

The free bus travel policy for youth was associated with an increase in rates of hospitalization due to injuries inflicted by assaults (Relative effect 19%; 95% CI 16% to 22%).

##### Secondary outcome: adverse events—injuries

The included study (Green et al., 2014) [71] reported a clear reduction in the incidence of road traffic injuries across all transport modes among those in the intervention arm at 3 years of follow-up (Ratio of ratios 0.84, 95% CI 0.82 to 0.87).

## Discussion

### Summary of main results and certainty of the evidence

This review included 33 studies assessing population-level interventions focused on infrastructure, policies and regulations to increase physical activity. Thirteen studies (1 cluster RCT and 12 CBA studies) assessed infrastructure changes to green or other spaces to promote physical activity. Evidence regarding these interventions is variable, and we remain very uncertain about the effects of the interventions on important health outcomes including physical activity (12 studies), body weight (2 studies) or blood pressure (one study).

Eighteen studies (15 CBA studies and three ITS studies) assessed infrastructure changes to promote active transport, such as building of cycle lanes, sidewalks, rail lines or motorways. Evidence regarding these interventions is very uncertain about their effects on physical activity (17 studies) and body weight (2 studies). The other two studies assessed the effects of policy and regulatory interventions. One assessed a policy that provided free access to physical activity facilities, reporting low certainty evidence that this approach may increase gym or swimming-related physical activity. The other assessed the effects of a policy providing free bus travel for youth aged 12–17 years; it showed that the effects were very uncertain.

The certainty of the evidence across interventions ranged from *low* to *very low certainty*. Almost all studies had an observational design, which started at low certainty. The reasons for downgrading the evidence further primarily included inconsistency, imprecision, and risk of bias. Risk of bias issues were mainly due to risk of selection, detection, and attrition biases. For all studies, except for the policy intervention providing free bus travel, the certainty of the evidence was not downgraded for indirectness. Our rationale for not downgrading for indirectness more frequently was threefold: i) LMIC settings were not part of our eligibility criteria, ii) some of the studies were conducted in low income settings within HICs, and iii) we had already downgraded the evidence to very low certainty.

### Overall completeness and applicability of evidence

All included studies were conducted in high-income countries, except for one conducted in Brazil – an upper-middle-income country (UMIC). Thus, the implementation of these types of interventions in LMIC settings, may require different considerations. Among the studies that were included in the review but not included in the synthesis, one was conducted in a lower-middle-income country (Iran) and two were conducted in an UMIC (China). All studies were also conducted in urban settings, with some of these being low-income urban communities or neighbourhoods.

Most of the included interventions focused on infrastructure interventions, with only two assessing policy and regulatory interventions. This may reflect the difficulty of conducting these types of studies using the study designs considered eligible for this review; many of the studies screened out had a relevant intervention but were lower-quality observational studies such as before-after studies without a control group or cross-sectional studies.

Major gaps in terms of outcomes reported are the lack of studies reporting on some of the primary outcomes of interest: CVD and diabetes morbidity and mortality. This may reflect the distance between the intervention and these types of outcomes along the effect pathway. Most of the studies also had short-term follow-up and thus it would be difficult to observe these longer-term outcomes.

The multi-pronged database searches for this review were last updated comprehensively in February 2020, as described in the Methods section, and thus the studies most recently published are not included in the review. We updated the Pubmed search in May 2022, which retrieved 2012 deduplicated records. A quick screening of these based on title keyword searches in Endnote (e.g. “green”, “infrastructure”, “cycling”, etc.) identified 10 records related to seven potentially eligible unique studies. We screened the full texts of these seven studies; three assessed an ineligible intervention, one had an ineligible study design, and three would be eligible for inclusion in this review, though two of these are still ongoing. These studies will be included in the update of this review and based on an informal assessment of the one completed study, we do not believe that the conclusions of this review would be altered by the inclusion of these results.

### Agreements and disagreements with other studies or reviews

Reviews assessing similar questions showed comparable results to this review including variability of effects, poor study quality and variability of measures used to assess physical activity and other outcomes. One review assessed the effectiveness of interventions in urban green space to encourage physical activity, which included those with physical changes to urban green spaces [80]. Of the nine included studies assessing these interventions, four showed benefits for increasing physical activity. The authors noted the need for more robust evaluations and that a combination of physical activity interventions plus physical environment modifications were probably the most effective approaches.

One recent systematic review assessed the association between access to public transport and childhood obesity [81]. It included 25 cross-sectional studies and two longitudinal studies conducted in 10 countries, mostly HICs except for one in Iran and one in China. Although they report that these studies showed inconsistent findings, they also found that most of the studies reported null associations between access to public transport and physical activity and/or body weight. Another review assessed the association between active transport to work or school and cardiovascular health and weight [82]. This review included 19 studies which showed that active transport was associated with improved cardiovascular health and lower body weight. However, the strength of the evidence varied for different outcomes and authors reported weak study designs and poor comparability between studies. Patterson and colleagues found a positive association between public transportation and lower BMI, as reported in 10 longitudinal studies included in the review [83]. Valdés-Badilla and colleagues assessed the effects of physical activity governmental programs on the health of independent older adults [84]. Five studies were included, which showed benefits of these programs for physical activity as well as for health outcomes such as blood pressure, blood glucose and blood lipids. However, included studies primarily assessed individual-level programs, comprising muscle-strengthening exercises, stretching, and walking, rather than population-level interventions. A systematic review of empirical and simulation studies evaluating the effects transportation interventions on health suggested that bike lanes and bus rapid transit systems can promote physical activity and active travel; however, this review did not assess the certainty of the evidence [85]. It also highlighted the fact that few longitudinal studies of these interventions that assess health outcomes exist and LMICs are understudied in the literature, similar to what we found.

The International Society for Physical Activity and Health (ISPAH) has outlined eight investments that they suggest work for physical activity; including active transport – designing cities to support walking, cycling and public transportation, active urban design – built environment elements that promote physical activity such as parks and urban green spaces, and community-wide programs including systems-based approaches such as policies to promote physical activity (International Society for Physical Activity and Health (ISPAH), 2020). This review did not find concrete evidence of the effectiveness of active transport or active urban design interventions due to the uncertain nature of the evidence base. However, one of the policy interventions included, which provided free access to physical activity facilities, showed potential to improve physical activity levels.

### Strengths and limitations

We searched multiple databases for ongoing and published studies and employed robust systematic review methodology. The update of the search was only carried out in one database – Medline – as this was the database, where most of the studies included in the first search had been identified.

Although the effects reported in included studies were often of very small magnitude, we considered that any effect different from the null might be relevant at a population level. The synthesis approach used, based on effect direction, did not allow us to provide an average effect measure for the interventions assessed; estimation of such an effect, however, would not have been possible for the identified evidence base.

## Authors’ conclusions

We identified, appraised, and synthesised 33 studies evaluating the effect of various infrastructure and policy and regulatory interventions for increasing physical activity, with varying results and often with very low certainty evidence. This was mostly due to issues with observational study designs and inconsistent or imprecise findings. Unarguably, public health interventions are challenging to measure with robust designs; however, efforts should be strengthened and investments made to use comparative study designs with adequate follow up periods to measure effects on short- and longer-term health outcomes. Similarly, further research in LMICs would be important to understand the different implementation issues in low-resource settings.

Despite this drawback, the evidence base provides indications thatpopulation-level interventions, such as providing access to physical activity facilities, may work. Furthermore, this review has provided details regarding relevant studies that could be considered for different settings in LMICs with due consideration of local contextual factors, barriers and enablers. When introducing new policies and interventions, these should ideally be monitored and evaluated robustly to inform enhancements and when to scale up or discontinue.

## Supplementary Information

**Additional file 1.** Search strategies.

**Additional file 2.** Characteristics of excluded studies.

**Additional file 3.** Characteristics of included studies.

**Additional file 4.** Table of studies not included in the synthesis.

**Additional file 5.** Characteristics of ongoing studies.

**Additional file 6.** Characteristics of studies awaiting classification.

**Additional file 7.** Detailed Risk of bias assessment.

**Additional file 8.** Individual study results table.

## Data Availability

All data generated or analysed during this study are included in this published article (and its supplementary information files).
