# Supplementary material for: Infrastructure, policy and regulatory interventions to increase physical activity to prevent cardiovascular diseases and diabetes: a systematic review
Source: BMC Public Health. 2023 Jan 16;23:112. doi: 10.1186/s12889-022-14841-y (PMC9841711; doi:10.1186/s12889-022-14841-y)
Supplement: Supplementary file 1 — Additional file 1. Search strategies. [file 12889_2022_14841_MOESM1_ESM.docx]

S1 file. Search strategies

|  | Ovid MEDLINE Epub Ahead of Print, In-Process & Other Non-Indexed Citations, Ovid MEDLINE Daily and Ovid MEDLINE (Run on 05 February, 2018) |  |
| --- | --- | --- |
| **#** | **Search Terms** | **Results** |
| 1 | exp exercise / or exp physical fitness/ or exp physical activity/ or (exercis* or sport or sports or physical activity or physical activities or physical education or physical training or fitness or leisure activity or leisure activities or physical conditioning or high-intensity interval training or sprint interval training or resistance training or running or jogging or swimming or walking or climbing or bicycling or biking or cycling or circuit training or run or jog or swim or walk or climb or aerobics or physical endurance or outdoor activity or outdoor activities or indoor activity or indoor activities).ti,ab. | 684931 |
| 2 | exp social planning/ or exp parks, recreational/ or exp public facilities/ or (environment design* or healthy place* or built environment* or environmental plan* or urban plan* or urban design* or urban environment or green space* or community park or urban park or recreational park or parks or public open space* or public space* or recreational cent* or recreational space* or playground* or social plan* or city plan* or town plan* or bicycle lane* or cycling lane* or bike lane* or sidewalk* or recreation cent* or recreational venue* or recreational facilit* or physical environment* or transport plan* or transport infrastructure* or transportation infrastructure* or public transport* or physical infrastructure or commuting or urban health or gym or public facilit* or community facilit* or leisure facilit* or exercise facilit* or fitness equipment or fitness facilit* or fitness cent*).ti,ab. | 32742 |
| 3 | exp environmental medicine/ or exp community health planning/ or exp health promotion/ or exp environmental policy/ or (wellness program* or health campaign*).ti,ab. or ((public or environmental or community or population or social) adj5 (strategy or strategies or program or programs or programme or programmes or policy or policies or intervention or interventions or regulation or regulations or legislation or legislations or legislative)).ti,ab. | 192477 |
| 4 | (randomized controlled trial or controlled clinical trial).pt. | 539956 |
| 5 | (randomized or placebo or (random* and trial* and group*)).mp. | 798391 |
| 6 | comparative study.pt. | 1788372 |
| 7 | control groups/ or control group*.mp. or follow-up studies/ or follow-up stud*.mp. or follow-up assessment.mp. or prospective studies/ or prospectiv*.mp. or non-random*.mp. or nonrandom*.mp. | 1604896 |
| 8 | (before after stud* or (time and series) or retrospective* or longitud* or (controlled and cohort* and stud*)).mp. | 1226273 |
| 9 | "before and after".mp. | 235690 |
| 10 | (controlled before or pre test or pretest or posttest or post test or pre intervention or post intervention).mp. | 35035 |
| 11 | 1 and 2 | 7297 |
| 12 | 1 and 3 | 21668 |
| 13 | 11 or 12 | 27378 |
| 14 | or/4-10 | 4579198 |
| 15 | 13 and 14 | 8912 |
| 16 | exp animals/ not humans/ | 4421271 |
| 17 | 15 not 16 | 8853 |

|  | Embase 1974 to 2018 February 2 (Run on 05 February, 2018) |  |
| --- | --- | --- |
| **#** | **Search Terms** | **Results** |
| 1 | exp exercise / or exp physical fitness/ or exp physical activity/ or (exercis* or sport or sports or physical activity or physical activities or physical education or physical training or fitness or leisure activity or leisure activities or physical conditioning or high-intensity interval training or sprint interval training or resistance training or running or jogging or swimming or walking or climbing or bicycling or biking or cycling or circuit training or run or jog or swim or walk or climb or aerobics or physical endurance or outdoor activity or outdoor activities or indoor activity or indoor activities).ti,ab. | 1046572 |
| 2 | exp social planning/ or exp parks, recreational/ or exp public facilities/ or (environment design* or healthy place* or built environment* or environmental plan* or urban plan* or urban design* or urban environment or green space* or community park or urban park or recreational park or parks or public open space* or public space* or recreational cent* or recreational space* or playground* or social plan* or city plan* or town plan* or bicycle lane* or cycling lane* or bike lane* or sidewalk* or recreation cent* or recreational venue* or recreational facilit* or physical environment* or transport plan* or transport infrastructure* or transportation infrastructure* or public transport* or physical infrastructure or commuting or urban health or gym or public facilit* or community facilit* or leisure facilit* or exercise facilit* or fitness equipment or fitness facilit* or fitness cent*).ti,ab. | 489406 |
| 3 | exp environmental medicine/ or exp community health planning/ or exp health promotion/ or exp environmental policy/ or (wellness program* or health campaign*).ti,ab. or ((public or environmental or community or population or social) adj5 (strategy or strategies or program or programs or programme or programmes or policy or policies or intervention or interventions or regulation or regulations or legislation or legislations or legislative)).ti,ab. | 349777 |
| 4 | randomized controlled trial/ or randomized/ or controlled study/ or comparative study/ or clinical study/ or quasi experimental study/ or experimental study/ or control group/ or follow up/ or prospective study/ or retrospective study/ | 7995568 |
| 5 | (randomized or placebo or (random* and trial* and group*)).mp. | 1148533 |
| 6 | (control group* or follow-up stud* or follow-up assessment or prospectiv* or non-random*or nonrandom*).mp. | 1516586 |
| 7 | (before after stud* or "before and after" or (time and series) or retrospective* or longitud* or (controlled and cohort* and stud*)).mp. | 1967065 |
| 8 | (controlled before or pre test or pretest or posttest or post test or pre intervention or post intervention).mp. | 51096 |
| 9 | 1 and 2 | 23207 |
| 10 | 1 and 3 | 32003 |
| 11 | 9 or 10 | 52472 |
| 12 | or/4-8 | 9415056 |
| 13 | 11 and 12 | 18298 |
| 14 | exp animals/ not humans/ | 10220141 |
| 15 | 13 not 14 | 11043 |

**Database: Web of Science**

**Date of search: 6 February 2018**

| **Set** | **Results** |  |
| --- | --- | --- |
| # 15 | [11,246](http://ezproxy.samrc.ac.za:2099/summary.do?product=WOS&doc=1&qid=15&SID=F42MkE6nKY2GUZpfX99&search_mode=CombineSearches&update_back2search_link_param=yes) | #14 AND #13  Indexes=SCI-EXPANDED, SSCI, A&HCI, ESCI Timespan=All years |
| # 14 | [48,506](http://ezproxy.samrc.ac.za:2099/summary.do?product=WOS&doc=1&qid=14&SID=F42MkE6nKY2GUZpfX99&search_mode=AdvancedSearch&update_back2search_link_param=yes) | #3 OR #7  Indexes=SCI-EXPANDED, SSCI, A&HCI, ESCI Timespan=All years |
| # 13 | [3,711,701](http://ezproxy.samrc.ac.za:2099/summary.do?product=WOS&doc=1&qid=13&SID=F42MkE6nKY2GUZpfX99&search_mode=CombineSearches&update_back2search_link_param=yes) | #12 OR #11 OR #10 OR #9 OR #8  Indexes=SCI-EXPANDED, SSCI, A&HCI, ESCI Timespan=All years |
| # 12 | [936,482](http://ezproxy.samrc.ac.za:2099/summary.do?product=WOS&doc=1&qid=12&SID=F42MkE6nKY2GUZpfX99&search_mode=AdvancedSearch&update_back2search_link_param=yes) | TS=(“control group*” OR “follow-up stud*” OR “follow-up assessment” OR prospectiv* OR “non-random*” OR nonrandom*)  Indexes=SCI-EXPANDED, SSCI, A&HCI, ESCI Timespan=All years |
| # 11 | [110,033](http://ezproxy.samrc.ac.za:2099/summary.do?product=WOS&doc=1&qid=11&SID=F42MkE6nKY2GUZpfX99&search_mode=AdvancedSearch&update_back2search_link_param=yes) | TS=“comparative study”  Indexes=SCI-EXPANDED, SSCI, A&HCI, ESCI Timespan=All years |
| # 10 | [41,652](http://ezproxy.samrc.ac.za:2099/summary.do?product=WOS&doc=1&qid=10&SID=F42MkE6nKY2GUZpfX99&search_mode=AdvancedSearch&update_back2search_link_param=yes) | TS=(“controlled before” OR “pre test” OR pretest OR “post test” OR posttest OR “pre intervention” OR “post intervention”)  Indexes=SCI-EXPANDED, SSCI, A&HCI, ESCI Timespan=All years |
| # 9 | [1,393,626](http://ezproxy.samrc.ac.za:2099/summary.do?product=WOS&doc=1&qid=9&SID=F42MkE6nKY2GUZpfX99&search_mode=AdvancedSearch&update_back2search_link_param=yes) | TS=(“before-after stud*” OR (time AND series) OR retrospective* OR longitud* OR “before and after” OR (controlled AND cohort AND stud*))  Indexes=SCI-EXPANDED, SSCI, A&HCI, ESCI Timespan=All years |
| # 8 | [1,812,135](http://ezproxy.samrc.ac.za:2099/summary.do?product=WOS&doc=1&qid=8&SID=F42MkE6nKY2GUZpfX99&search_mode=AdvancedSearch&update_back2search_link_param=yes) | TS=(“randomized controlled trial” OR “controlled clinical trial” OR randomi?ed OR placebo OR “clinical trials” OR randomly OR trial)  Indexes=SCI-EXPANDED, SSCI, A&HCI, ESCI Timespan=All years |
| # 7 | [30,444](http://ezproxy.samrc.ac.za:2099/summary.do?product=WOS&doc=1&qid=7&SID=F42MkE6nKY2GUZpfX99&search_mode=CombineSearches&update_back2search_link_param=yes) | #6 AND #1  Indexes=SCI-EXPANDED, SSCI, A&HCI, ESCI Timespan=All years |
| # 6 | [278,422](http://ezproxy.samrc.ac.za:2099/summary.do?product=WOS&doc=1&qid=6&SID=F42MkE6nKY2GUZpfX99&search_mode=CombineSearches&update_back2search_link_param=yes) | #5 OR #4  Indexes=SCI-EXPANDED, SSCI, A&HCI, ESCI Timespan=All years |
| # 5 | [252,613](http://ezproxy.samrc.ac.za:2099/summary.do?product=WOS&doc=1&qid=5&SID=F42MkE6nKY2GUZpfX99&search_mode=AdvancedSearch&update_back2search_link_param=yes) | TS=((public OR environmental OR community OR social OR population) NEAR/5 (strategy OR strategies OR program OR programs OR programme OR programmes OR policy OR policies OR intervention OR interventions OR regulation OR regulations OR legislation OR legislations OR legislative))  Indexes=SCI-EXPANDED, SSCI, A&HCI, ESCI Timespan=All years |
| # 4 | [31,149](http://ezproxy.samrc.ac.za:2099/summary.do?product=WOS&doc=1&qid=4&SID=F42MkE6nKY2GUZpfX99&search_mode=AdvancedSearch&update_back2search_link_param=yes) | TS=(“environmental medicine” OR “community health planning” OR “health promotion” OR “wellness program*” OR “health campaign*”)  Indexes=SCI-EXPANDED, SSCI, A&HCI, ESCI Timespan=All years |
| # 3 | [20,162](http://ezproxy.samrc.ac.za:2099/summary.do?product=WOS&doc=1&qid=3&SID=F42MkE6nKY2GUZpfX99&search_mode=CombineSearches&update_back2search_link_param=yes) | #2 AND #1  Indexes=SCI-EXPANDED, SSCI, A&HCI, ESCI Timespan=All years |
| # 2 | [157,317](http://ezproxy.samrc.ac.za:2099/summary.do?product=WOS&doc=1&qid=2&SID=F42MkE6nKY2GUZpfX99&search_mode=AdvancedSearch&update_back2search_link_param=yes) | TS=(“social planning” OR “environment design*” OR “healthy place*” OR “built environment*” OR “environmental plan*” OR “urban plan*” OR “urban design*” OR “urban environment” OR “green space*” OR “community park” OR “urban park” OR “recreational park” OR parks OR “public open space*” OR “public space*” OR playground* OR “social plan*” OR “city plan*” OR “town plan*” OR “bicycle lane*” OR “cycling lane*” OR “bike lane*” OR sidewalk* OR “recreation cent*” OR “recreational cent*” OR “recreational venue*” OR “recreational facilit*” OR “recreational space*” OR “physical environment*” OR “transport plan*” OR “transport infrastructure*” OR “transportation infrastructure*” OR “public transport*” OR “physical infrastructure” OR commuting OR “urban health” OR gym OR “public facilit*” OR “community facilit*” OR “leisure facilit*” OR “exercise facilit*” OR “fitness equipment” OR “fitness facilit*” OR “fitness cent*”)  Indexes=SCI-EXPANDED, SSCI, A&HCI, ESCI Timespan=All years |
| # 1 | [2,026,227](http://ezproxy.samrc.ac.za:2099/summary.do?product=WOS&doc=1&qid=1&SID=F42MkE6nKY2GUZpfX99&search_mode=AdvancedSearch&update_back2search_link_param=yes) | TS=(Exercis* OR sport OR sports OR “physical activity” OR “physical activities” OR “physical education” OR “physical training” OR fitness OR “leisure activity” OR “leisure activities” OR “physical conditioning” OR “high-intensity interval training” OR “sprint interval training” OR “resistance training” OR running OR jogging OR swimming OR walking OR climbing OR bicycling OR biking OR cycling OR “circuit training” OR run OR jog OR swim OR walk OR climb OR aerobics OR “physical fitness” OR “physical endurance” OR “outdoor activity” OR “outdoor activities” OR “indoor activity” OR “indoor activities”)  Indexes=SCI-EXPANDED, SSCI, A&HCI, ESCI Timespan=All years |
