# Supplementary material for: Infrastructure, policy and regulatory interventions to increase physical activity to prevent cardiovascular diseases and diabetes: a systematic review
Source: BMC Public Health. 2023 Jan 16;23:112. doi: 10.1186/s12889-022-14841-y (PMC9841711; doi:10.1186/s12889-022-14841-y)
Supplement: Supplementary file 2 — Additional file 2. Characteristics of excluded studies. [file 12889_2022_14841_MOESM2_ESM.docx]

Characteristics of excluded studies

| **Study** | **Reason for exclusion** |
| --- | --- |
| Aittasalo 2017(1) | Wrong study design |
| Aittasalo 2019(2) | Wrong study design |
| Amin 2010(3) | Wrong study design |
| Curl 2018(4) | Wrong intervention |
| Atalla 2019(5) | Wrong study design |
| Avila-Palencia 2018(6) | Wrong study design |
| Baquero 2018(7) | Wrong study design |
| Baquet 2018(8) | Wrong intervention |
| Barbosa Filho 2019(9) | Wrong intervention |
| Barrett-Williams 2017(10) | Wrong intervention |
| Beenackers 2012(11) | Wrong study design |
| Bentley 2018(12) | Wrong study design |
| Benton 2018(13) | Wrong intervention |
| Bird 2014(14) | Wrong study design |
| Blond 2016(15) | Wrong intervention |
| Bowen 2018(16) | Wrong intervention |
| Boyle-Holmes 2010(17) | Wrong intervention |
| Brand 2014(18) | Wrong outcomes |
| Braun 2016(19) | Wrong study design |
| Briancon 2020(20) | Wrong intervention |
| Brown 2007(21) | Wrong study design |
| Brown 2009(22) | Wrong study design |
| Brown 2015(23) | Wrong study design |
| Brown 2016(24) | Wrong study design |
| Brownson 2004(25) | Wrong intervention |
| Brownson 2005(26) | Wrong intervention |
| Buckley 2013(27) | Wrong study design |
| Buliung 2011(28) | Wrong intervention |
| Bungum 2014(29) | Wrong intervention |
| Burbidge 2009(30) | Wrong study design |
| Canaway 2019(31) | Wrong study design |
| Cardon 2009(32) | Wrong intervention |
| Carroll 2020(33) | Wrong study design |
| Celis-Morales 2017(34) | Wrong study design |
| Centeio 2018(35) | Wrong intervention |
| Chandrabose 2019(36) | Wrong study design |
| Chuang 2018(37) | Wrong intervention |
| Clark 2014(38) | Wrong intervention |
| Clark 2019(39) | Wrong intervention |
| Cohen 2013(40) | Wrong intervention |
| Coombes 2016(41) | Wrong intervention |
| Cordeiro 2018(42) | Wrong intervention |
| Cranney 2016(43) | Wrong study design |
| Crawford 2013(44) | Wrong study design |
| D’Agostino 2018(45) | Wrong study design |
| DeCocker 2007(46) | Wrong intervention |
| delCampoVega 2017(47) | Wrong study design |
| DeMeester 2014(48) | Wrong study design |
| Boles 2011(49) | Wrong intervention |
| DiMaggio 2013(50) | Wrong study design |
| Dinu 2019(51) | Wrong study design |
| Droomers 2015(52) | Duplicate |
| Ducheyne 2014(53) | Wrong intervention |
| Dudley 2018(54) | Wrong intervention |
| Economos 2007(55) | Wrong intervention |
| Economos 2013(56) | Wrong intervention |
| Edwards 2006(57) | Wrong study design |
| Evenson 2005(58) | Wrong study design |
| Faerstein (59)2018 | Wrong study design |
| Foster 2018(60) | Wrong study design |
| Giles-Corti 2013(61) | Wrong study design |
| Goerke 2019 (62) | Wrong study design |
| Goodman 2013 (63) | Duplicate |
| Goodman 2016 (64) | Wrong intervention |
| Green 2014(65) | Duplicate |
| Gutierrez 2014(66) | Wrong intervention |
| Heesch 2016(67) | Wrong study design |
| Heinen 2015(68) | Wrong study design |
| Henderson 2013(69) | Wrong study design |
| Hinckson 2011(70) | Wrong study design |
| Hoelscher 2016(71) | Wrong intervention |
| Hooper 2019(72) | Wrong study design |
| Hunter 2019(73) | Wrong study design |
| Jia 2019(74) | Wrong study design |
| Jia 2019(75) | Wrong study design |
| Karmeniemi 2018(76) | Wrong study design |
| King 2015(77) | Wrong study design |
| Knott 2019(78) | Wrong study design |
| Droomers 2016(52) | Duplicate |
| Larouche 2018(79) | Wrong study design |
| Laverty 2018(80) | Wrong intervention |
| Laxer 2014(81) | Wrong intervention |
| Levinger 2019(82) | Uncontrolled pre-post |
| Lewis 2019(83) | Wrong intervention |
| Lupton 2003(84) | Wrong intervention |
| MacMillan 2018(85) | Wrong study design |
| Mammen 2014(86) | Wrong study design |
| Mammen 2014(87) | Wrong study design |
| Matsudo 2002(88) | Wrong study design |
| McCormack 2016(89) | Wrong intervention |
| McGavock 2019 (90) | Wrong intervention |
| McMinn 2012 (91) | Wrong intervention |
| Mendoza 2011(92) | Wrong intervention |
| Merom 2003(93) | Wrong study design |
| Michael 2014(94) | Wrong study design |
| Miller 2015(95) | Wrong study design |
| Miller 2018(96) | Wrong study design |
| Milner 2018(97) | Wrong intervention |
| Molenberg 2019(98) | Wrong study design |
| Morris 1997(99) | Wrong study design |
| Mutrie 2000 (100) | Wrong intervention |
| Nathan 2018(101) | Wrong study design |
| Nathan 2019(102) | Wrong intervention |
| Nguyen 2015 (103) | Wrong study design |
| Ogilvie 2010(104) | Wrong study design |
| Panter 2015(105) | Wrong study design |
| Panter 2016(106) | Wrong study design |
| Patterson 2019(107) | Wrong study design |
| Pedroso 2016(108) | Wrong study design |
| Peters 2016(109) | Wrong intervention |
| Reger-Nash 2005(110) | Wrong intervention |
| Robinson 2019(111) | Wrong intervention |
| Rodriguez 2019(112) | Wrong intervention |
| Roemmich 2014(113) | Wrong study design |
| Rowland 2003(114) | Wrong intervention |
| Sahlqvist 2013(115) | Wrong study design |
| Saito 2018(116) | Wrong intervention |
| Salinas 2018 (117) | Wrong intervention |
| Salopuro 2010(118) | Wrong intervention |
| Sayers 2012(119) | Wrong intervention |
| Slater 2019(120) | Wrong study design |
| Smith 2019(121) | Wrong study design |
| Solomon 2014(122) | Wrong intervention |
| Song 2017 | Exclusion reason: Wrong study design; |
| Stewart 2014 | Exclusion reason: Wrong study design; |
| Taddei 2015 | Exclusion reason: Wrong study design; |
| Tudor-Smith 1998 | Exclusion reason: Wrong intervention; |
| Vanwolleghem 2014 | Exclusion reason: Wrong intervention; |
| Villa-Gonzalez 2018 | Exclusion reason: Wrong study design; |
| Weinstock 2020 | Exclusion reason: Wrong setting; |
| Wells 2008 | Exclusion reason: Wrong study design; |
| Wyatt 2018 | Exclusion reason: Wrong intervention; |
| Smith nd | wrong intervention |
| Ogilvie 2017 | wrong study design |
| Boles 2011 | wrong study design |

**REFERENCES**

1. Aittasalo M, Tiilikainen J, Tokola K, Seimela T, Sarjala SM, Metsapuro P, et al. Socio-ecological intervention to promote active commuting to work: Protocol and baseline findings of a cluster randomized controlled trial in Finland. International Journal of Environmental Research and Public Health. 2017;14(10):1257.

2. Aittasalo M, Tiilikainen J, Tokola K, Suni J, Sievanen H, Vaha-Ypya H, et al. Socio-Ecological Natural Experiment with Randomized Controlled Trial to Promote Active Commuting to Work: Process Evaluation, Behavioral Impacts, and Changes in the Use and Quality of Walking and Cycling Paths. International journal of environmental research and public health. 2019;16(9).

3. Amin H, Vasquez KR, Thilagaratnam S, Choo L, Tseng P. Lose to win: A national workplace weight management programme. Obesity Reviews. 2010;11(SUPPL. 1):60.

4. Curl A, Kearns A, Macdonald L, Mason P, Ellaway A. Can walking habits be encouraged through area-based regeneration and relocation? A longitudinal study of deprived communities in Glasgow, UK. Journal of Transport & Health. 2018;10:44-55.

5. Atalla M, Pinto AJ, Mielke GI, Benatti FB, Gualano B. Impact of a Real-World Lifestyle Intervention in an Entire Latin American City with More Than 50,000 People. Obesity. 2019;27(12):1967-74.

6. Avila-Palencia I, Int Panis L, Dons E, Gaupp-Berghausen M, Raser E, Gotschi T, et al. The effects of transport mode use on self-perceived health, mental health, and social contact measures: A cross-sectional and longitudinal study. Environment international. 2018;120:199-206.

7. Baquero B, Kava CM, Ashida S, Daniel-Ulloa J, Laroche HH, Haines H, et al. Active Ottumwa: Adapting Evidence-Based Recommendations to Promote Physical Activity in a Micropolitan New Destination Community. International journal of environmental research and public health. 2018;15(5).

8. Baquet G, Aucouturier J, Gamelin FX, Berthoin S. Longitudinal Follow-Up of Physical Activity During School Recess: Impact of Playground Markings. Frontiers in public health. 2018;6:283.

9. Barbosa Filho VC, Bandeira AdS, Minatto G, Linard JG, Silva JAd, Costa RMd, et al. Effect of a Multicomponent Intervention on Lifestyle Factors among Brazilian Adolescents from Low Human Development Index Areas: A Cluster-Randomized Controlled Trial. International journal of environmental research and public health. 2019;16(2).

10. Barrett-Williams SL, Franks P, Kay C, Meyer A, Cornett K, Mosier B. Bridging Public Health and Education: Results of a School-Based Physical Activity Program to Increase Student Fitness. Public health reports (Washington, DC : 1974). 2017;132(2_suppl):81S-7S.

11. Beenackers MA, Foster S, Kamphuis CBM, Titze S, Divitini M, Knuiman M, et al. Taking up cycling after residential relocation: built environment factors. American journal of preventive medicine. 2012;42(6):610-5.

12. Bentley R, Blakely T, Kavanagh A, Aitken Z, King T, McElwee P, et al. A Longitudinal Study Examining Changes in Street Connectivity, Land Use, and Density of Dwellings and Walking for Transport in Brisbane, Australia. Environmental health perspectives. 2018;126(5):057003.

13. Benton JS, Anderson J, Cotterill S, Dennis M, Lindley SJ, French DP. Evaluating the impact of improvements in urban green space on older adults' physical activity and wellbeing: protocol for a natural experimental study. BMC public health. 2018;18(1):923.

14. Bird EL, Powell JE, Ogilvie D, Goodman A, Rutter H, editors. Health economic assessment of walking and cycling interventions in the physical environment: Interim findings from the iConnect study2014.

15. Blond K, Jensen MK, Rasmussen MG, Overvad K, Tjønneland A, Østergaard L, et al. Prospective Study of Bicycling and Risk of Coronary Heart Disease in Danish Men and Women. Circulation. 2016;134(18):1409-11.

16. Bowen DJ, Quintiliani LM, Bhosrekar SG, Goodman R, Smith E. Changing the housing environment to reduce obesity in public housing residents: a cluster randomized trial. BMC public health. 2018;18(1):883.

17. Boyle-Holmes T, Grost L, Russell L, Laris BA, Robin L, Haller E, et al. Promoting elementary physical education: results of a school-based evaluation study. Health education & behavior : the official publication of the Society for Public Health Education. 2010;37(3):377-89.

18. Brand C, Goodman A, Ogilvie D. Evaluating the impacts of new walking and cycling infrastructure on carbon dioxide emissions from motorized travel: a controlled longitudinal study. Applied energy. 2014;128:284-95.

19. Braun LM, Rodriguez DA, Song Y, Meyer KA, Lewis CE, Reis JP, et al. Changes in walking, body mass index, and cardiometabolic risk factors following residential relocation: Longitudinal results from the CARDIA study. Journal of transport & health. 2016;3(4):426-39.

20. Briancon S, Legrand K, Muller L, Langlois J, Saez L, Spitz E, et al. Effectiveness of a socially adapted intervention in reducing social inequalities in adolescence weight. The PRALIMAP-INES school-based mixed trial. International journal of obesity (2005). 2020.

21. Brown BB, Werner CM. A New Rail Stop: Tracking Moderate Physical Activity Bouts and Ridership. American Journal of Preventive Medicine. 2007;33(4):306-9.

22. Brown BB, Werner CM. Before and After a New Light Rail Stop: Resident Attitudes, Travel Behavior, and Obesity. Journal of the American Planning Association. 2008;75(1):5-12.

23. Brown BB, Werner CM, Tribby CP, Miller HJ, Smith KR. Transit Use, Physical Activity, and Body Mass Index Changes: Objective Measures Associated With Complete Street Light-Rail Construction. American Journal of Public Health. 2015;105(7):1468-74.

24. Brown BB, Tharp D, Tribby CP, Smith KR, Miller HJ, Werner CM. Changes in bicycling over time associated with a new bike lane: relations with kilocalories energy expenditure and body mass index. Journal of transport & health. 2016;3(3):357-65.

25. Brownson RC, Baker EA, Boyd RL, Caito NM, Duggan K, Housemann RA, et al. A community-based approach to promoting walking in rural areas. American journal of preventive medicine. 2004;27(1):28-34.

26. Brownson RC, Hagood L, Lovegreen SL, Britton B, Caito NM, Elliott MB, et al. A multilevel ecological approach to promoting walking in rural communities. Preventive Medicine. 2005;41(5-6):837-42.

27. Buckley A, Lowry MB, Brown H, Barton B. Evaluating safe routes to school events that designate days for walking and bicycling. Transport Policy. 2013;30:294-300.

28. Buliung R, Faulkner G, Beesley T, Kennedy J. School Travel Planning: Mobilizing School and Community Resources to Encourage Active School Transportation. Journal of School Health. 2011;81(11):704-12.

29. Bungum TJ, Clark S, Aguilar B. The Effect of an Active Transport to School Intervention at a Suburban Elementary School. American Journal of Health Education. 2014;45(4):205-9.

30. Burbidge SK, Goulias KG. Evaluating the Impact of Neighborhood Trail Development on Active Travel Behavior and Overall Physical Activity of Suburban Residents. Transportation Research Record. 2009(2135):78-86.

31. Canaway A, Frew E, Lancashire E, Pallan M, Hemming K, Adab P, et al. Economic evaluation of a childhood obesity prevention programme for children: Results from the WAVES cluster randomised controlled trial conducted in schools. PloS one. 2019;14(7):e0219500.

32. Cardon G, Labarque V, Smits D, De Bourdeaudhuij I. Promoting physical activity at the pre-school playground: the effects of providing markings and play equipment. Preventive medicine. 2009;48(4):335-40.

33. Carroll SJ, Dale MJ, Taylor AW, Daniel M. Contributions of multiple built environment features to 10-year change in body mass index and waist circumference in a south australian middle-aged cohort. International Journal of Environmental Research and Public Health. 2020;17(3):870.

34. Celis-Morales CA, Lyall DM, Welsh P, Anderson J, Steell L, Guo Y, et al. Association between active commuting and incident cardiovascular disease, cancer, and mortality: prospective cohort study. BMJ (Clinical research ed). 2017;357:j1456.

35. Centeio EE, McCaughtry N, Moore EWG, Kulik N, Garn A, Martin J, et al. Building healthy communities: A comprehensive school health program to prevent obesity in elementary schools. Preventive medicine. 2018;111:210-5.

36. Chandrabose M, Rachele JN, Gunn L, Kavanagh A, Owen N, Turrell G, et al. Built environment and cardio-metabolic health: systematic review and meta-analysis of longitudinal studies. Obesity reviews : an official journal of the International Association for the Study of Obesity. 2019;20(1):41-54.

37. Chuang R-J, Sharma SV, Perry C, Diamond P. Does the CATCH Early Childhood Program Increase Physical Activity Among Low-Income Preschoolers?-Results From a Pilot Study. American journal of health promotion : AJHP. 2018;32(2):344-8.

38. Clark S, Bungum T, Shan GG, Meacham M, Coker L. The effect of a trail use intervention on urban trail use in Southern Nevada. Preventive Medicine. 2014;67:S17-S20.

39. Clark AF, Campbell J, Tucker P, Wilk P, Gilliland JA. If You Make it Free, Will They Come? Using a Physical Activity Accessibility Model to Understand the Use of a Free Children's Recreation Pass. Journal of physical activity & health. 2019;16(7):493-503.

40. Cohen DA, Han B, Derose KP, Williamson S, Marsh T, McKenzie TL. Physical activity in parks: A randomized controlled trial using community engagement. American journal of preventive medicine. 2013;45(5):590-7.

41. Coombes E, Jones A. Gamification of active travel to school: A pilot evaluation of the Beat the Street physical activity intervention. Health & Place. 2016;39:62-9.

42. Cordeiro R, Monteiro W, Cunha F, Pescatello LS, Farinatti P. Influence of Acute Concurrent Exercise Performed in Public Fitness Facilities on Ambulatory Blood Pressure Among Older Adults in Rio de Janeiro City. Journal of strength and conditioning research. 2018;32(10):2962-70.

43. Cranney L, Phongsavan P, Kariuki M, Stride V, Scott A, Hua M, et al. Impact of an outdoor gym on park users' physical activity: A natural experiment. Health & place. 2016;37:26-34.

44. Crawford S, Garrard J. A Combined Impact-Process Evaluation of a Program Promoting Active Transport to School: Understanding the Factors That Shaped Program Effectiveness. Journal of Environmental and Public Health. 2013;2013:816961.

45. D'Agostino EM, Day SE, Konty KJ, Larkin M, Saha S, Wyka K. The association of fitness and school absenteeism across gender and poverty: a prospective multilevel analysis in New York City middle schools. Annals of epidemiology. 2018;28(3):189-96.

46. De Cocker KA, De Bourdeaudhuij IM, Brown WJ, Cardon GM. Effects of "10,000 steps Ghent" - A whole-community intervention. American Journal of Preventive Medicine. 2007;33(6):455-63.

47. Del Campo Vega C, Tutte V, Bermudez G, Parra DC. Impact on Area-Level Physical Activity Following the Implementation of a Fitness Zone in Montevideo, Uruguay. Journal of physical activity & health. 2017;14(11):883-7.

48. De Meester F, Van Dyck D, De Bourdeaudhuij I, Deforche B, Cardon G. Changes in physical activity during the transition from primary to secondary school in Belgian children: what is the role of the school environment? BMC public health. 2014;14:261.

49. Boles M, Dilley JA, Dent C, Elman MR, Duncan SC, Johnson DB. Changes in local school policies and practices in Washington State after an unfunded physical activity and nutrition mandate. Preventing chronic disease. 2011;8(6):A129.

50. DiMaggio C, Li G. Effectiveness of a Safe Routes to School Program in Preventing School-Aged Pedestrian Injury. Pediatrics. 2013;131(2):290-6.

51. Dinu M, Pagliai G, Macchi C, Sofi F. Active Commuting and Multiple Health Outcomes: A Systematic Review and Meta-Analysis. Sports medicine (Auckland, NZ). 2019;49(3):437-52.

52. Droomers M, Jongeneel-Grimen B, Kramer D, de Vries S, Kremers S, Bruggink J-W, et al. The impact of intervening in green space in Dutch deprived neighbourhoods on physical activity and general health: results from the quasi-experimental URBAN40 study. Journal of epidemiology and community health. 2016;70(2):147-54.

53. Ducheyne F, De Bourdeaudhuij I, Lenoir M, Spittaels H, Cardon G. Children's cycling skills: Development of a test and determination of individual and environmental correlates. Accident; analysis and prevention. 2013;50:688-97.

54. Dudley D, Cotton W, Peralta L, Winslade M. A stepped-wedge implementation and evaluation of the healthy active peaceful playgrounds for youth (HAPPY) intervention. BMC public health. 2018;18(1):532.

55. Economos CD, Hyatt RR, Goldberg JP, Must A, Naumova EN, Collins JJ, et al. A community intervention reduces BMI z-score in children: Shape Up Somerville first year results. Obesity (Silver Spring, Md). 2007;15(5):1325-36.

56. Economos CD, Hyatt RR, Must A, Goldberg JP, Kuder J, Naumova EN, et al. Shape Up Somerville two-year results: a community-based environmental change intervention sustains weight reduction in children. Preventive medicine. 2013;57(4):322-7.

57. Edwards P, Tsouros A. Promoting physical activity and active living in urban environments: The role of local governments. Denmark: WHO Regional Office for Europe; 2006.

58. Evenson KR, Herring AH, Huston SL. Evaluating change in physical activity with the building of a multi-use trail. American journal of preventive medicine. 2005;28(2 Suppl 2):177-85.

59. Faerstein E, da Silveira IH, Boclin KdLS, Curioni CC, Castro IRRd, Junger WL. Associations of neighborhood socioeconomic, natural and built environmental characteristics with a 13-year trajectory of non-work physical activity among civil servants in Rio de Janeiro, Brazil: The Pro-Saude Study. Health & place. 2018;53:110-6.

60. Foster C, Kelly P, Reid HAB, Roberts N, Murtagh EM, Humphreys DK, et al. What works to promote walking at the population level? A systematic review. British Journal of Sports Medicine. 2018;52(12):807-12.

61. Giles-Corti B, Macaulay G, Middleton N, Boruff B, Bull F, Butterworth I, et al. Developing a research and practice tool to measure walkability: a demonstration project. Health Promotion Journal of Australia. 2014;25(3):160-6.

62. Goerke D, Zolfaghari E, Marek AP, Endorf FW, Nygaard RM. Incidence and Profile of Severe Cycling Injuries After Bikeway Infrastructure Changes. Journal of community health. 2019.

63. Goodman A, Sahlqvist S, Ogilvie D, iConnect c. Who uses new walking and cycling infrastructure and how? Longitudinal results from the UK iConnect study. Preventive medicine. 2013;57(5):518-24.

64. Goodman A, van Sluijs EMF, Ogilvie D. Impact of offering cycle training in schools upon cycling behaviour: a natural experimental study. International Journal of Behavioral Nutrition and Physical Activity. 2016;13(1):34.

65. Green J, Steinbach R, Jones A, Edwards P, Kelly C, Nellthorp J, et al. On the buses: a mixed-method evaluation of the impact of free bus travel for young people on the public health. Public Health Res. 2014;2(1).

66. Gutierrez CM, Slagle D, Figueras K, Anon A, Huggins AC, Hotz G. Crossing guard presence: Impact on active transportation and injury prevention. Journal of Transport & Health. 2014;1(2):116-23.

67. Heesch KC, James B, Washington TL, Zuniga K, Burke M. Evaluation of the Veloway 1: A natural experiment of new bicycle infrastructure in Brisbane, Australia. Journal of Transport & Health. 2016;3(3):366-76.

68. Heinen E, Panter J, Mackett R, Ogilvie D. Changes in mode of travel to work: A natural experimental study of new transport infrastructure. International Journal of Behavioral Nutrition and Physical Activity. 2015.

69. Henderson S, Tanner R, Klanderman N, Mattera A, Webb LM, Steward J. Safe Routes to School: A Public Health Practice Success Story—Atlanta, 2008−2010. Journal of Physical Activity and Health. 2013;10(2):141-2.

70. Hinckson EA, Badland HM. School Travel Plans: Preliminary Evidence for Changing School-Related Travel Patterns in Elementary School Children. American Journal of Health Promotion. 2011;25(6):368-71.

71. Hoelscher D, Ory M, Dowdy D, Miao J, Atteberry H, Nichols D, et al. Effects of Funding Allocation for Safe Routes to School Programs on Active Commuting to School and Related Behavioral, Knowledge, and Psychosocial Outcomes: Results From the Texas Childhood Obesity Prevention Policy Evaluation (T-COPPE) Study. Environment and Behavior. 2016;48(1):210-29.

72. Hooper P, Foster S, Giles-Corti B. A Case Study of a Natural Experiment Bridging the 'Research into Policy' and 'Evidence-Based Policy' Gap for Active-Living Science. International journal of environmental research and public health. 2019;16(14).

73. Hunter RF, Cleland C, Cleary A, Droomers M, Wheeler BW, Sinnett D, et al. Environmental, health, wellbeing, social and equity effects of urban green space interventions: A meta-narrative evidence synthesis. Environment International. 2019;130:104923.

74. Jia Y, Ding D, Gebel K, Chen L, Zhang S, Ma Z, et al. Effects of new dock-less bicycle-sharing programs on cycling: a retrospective study in Shanghai. BMJ open. 2019;9(2):e024280.

75. Jia P, Xue H, Cheng X, Wang Y, Wang Y. Association of neighborhood built environments with childhood obesity: Evidence from a 9-year longitudinal, nationally representative survey in the US. Environment international. 2019;128:158-64.

76. Karmeniemi M, Lankila T, Ikaheimo T, Koivumaa-Honkanen H, Korpelainen R. The Built Environment as a Determinant of Physical Activity: A Systematic Review of Longitudinal Studies and Natural Experiments. Annals of behavioral medicine : a publication of the Society of Behavioral Medicine. 2018;52(3):239-51.

77. King DK, Litt J, Hale J, Burniece KM, Ross C. 'The park a tree built': Evaluating how a park development project impacted where people play. Urban Forestry & Urban Greening. 2015;14(2):293-9.

78. Knott CS, Sharp SJ, Mytton OT, Ogilvie D, Panter J. Changes in workplace car parking and commute mode: a natural experimental study. Journal of epidemiology and community health. 2019;73(1):42-9.

79. Larouche R, Mammen G, Rowe DA, Faulkner G. Effectiveness of active school transport interventions: a systematic review and update. BMC public health. 2018;18(1):206.

80. Laverty AA, Webb E, Vamos EP, Millett C. Associations of increases in public transport use with physical activity and adiposity in older adults. The international journal of behavioral nutrition and physical activity. 2018;15(1):31.

81. Leatherdale ST, Brown KS, Carson V, Childs RA, Dubin JA, Elliott SJ, et al. The COMPASS study: a longitudinal hierarchical research platform for evaluating natural experiments related to changes in school-level programs, policies and built environment resources. Bmc Public Health. 2014;14:7.

82. Levinger P, Panisset M, Dunn J, Haines T, Dow B, Batchelor F, et al. Exercise interveNtion outdoor proJect in the cOmmunitY for older people - the ENJOY Senior Exercise Park project translation research protocol. BMC public health. 2019;19(1):933.

83. Lewis S, Bambra C, Barnes A, Collins M, Egan M, Halliday E, et al. Reframing "participation" and "inclusion" in public health policy and practice to address health inequalities: Evidence from a major resident-led neighbourhood improvement initiative. Health & social care in the community. 2019;27(1):199-206.

84. Lupton BS, Fonnebo V, Sogaard AJ. The Finnmark Intervention Study: Is it possible to change CVD risk factors by community-based intervention in an Arctic village in crisis? Scandinavian Journal of Public Health. 2003;31(3):178-86.

85. MacMillan F, George ES, Feng X, Merom D, Bennie A, Cook A, et al. Do Natural Experiments of Changes in Neighborhood Built Environment Impact Physical Activity and Diet? A Systematic Review. International journal of environmental research and public health. 2018;15(2).

86. Mammen G, Stone MR, Faulkner G, Ramanathan S, Buliung R, O'Brien C, et al. Active school travel: an evaluation of the Canadian school travel planning intervention. Preventive medicine. 2014;60:55-9.

87. Mammen G, Stone MR, Buliung R, Faulkner G. School travel planning in Canada: Identifying child, family, and school-level characteristics associated with travel mode shift from driving to active school travel. Journal of Transport & Health. 2014;1(4):288-94.

88. Matsudo V, Matsudo S, Andrade D, Araujo T, Andrade E, De Oliveira LC, et al. Promotion of physical activity in a developing country: The Agita Sao Paulo experience. Public Health Nutrition. 2002;5(1 A):253-61.

89. McCormack GR, Graham TM, Swanson K, Massolo A, Rock MJ. Changes in visitor profiles and activity patterns following dog supportive modifications to parks: A natural experiment on the health impact of an urban policy. SSM - Population Health. 2016;2:237-43.

90. McGavock J, Brunton N, Klaprat N, Swanson A, Pancoe D, Manley E, et al. Walking on Water-A Natural Experiment of a Population Health Intervention to Promote Physical Activity after the Winter Holidays. International journal of environmental research and public health. 2019;16(19).

91. McMinn D, Rowe DA, Murtagh S, Nelson NM. The effect of a school-based active commuting intervention on children's commuting physical activity and daily physical activity. Preventive medicine. 2012;54(5):316-8.

92. Mendoza JA, Watson K, Nguyen N, Cerin E, Baranowski T, Nicklas TA. Active commuting to school and association with physical activity and adiposity among US youth. Journal of physical activity & health. 2011;8(4):488-95.

93. Merom D, Bauman A, Vita P, Close G. An environmental intervention to promote walking and cycling - The impact of a newly constructed Rail Trail in Western Sydney. Preventive Medicine. 2003;36(2):235-42.

94. Michael YL, Nagel CL, Gold R, Hillier TA. Does change in the neighborhood environment prevent obesity in older women? Social Science & Medicine. 2014;102:129-37.

95. Miller HJ, Tribby CP, Brown BB, Smith KR, Werner CM, Wolf J, et al. Public transit generates new physical activity: Evidence from individual GPS and accelerometer data before and after light rail construction in a neighborhood of Salt Lake City, Utah, USA. Health & place. 2015;36:8-17.

96. Miller GF, Sliwa S, Michael S, Lee S, Burgeson C, Krautheim AM, et al. Evaluation of Let's Move! active schools activation grants. Preventive medicine. 2018;108:36-40.

97. Milner S, Sherker S, Clinton-Mcharg T, Dray J, Zukowski N, Gonzalez S, et al. Cluster randomised controlled trial of a multicomponent intervention to support the implementation of policies and practices that promote healthier environments at junior sports clubs: Study protocol. BMJ Open. 2018;8(1):e018906.

98. Molenberg FJM, Panter J, Burdorf A, Van Lenthe FJ. A systematic review of the effect of infrastructural interventions to promote cycling: Strengthening causal inference from observational data. International Journal of Behavioral Nutrition and Physical Activity. 2019;16(1):93.

99. Morris JN, Hardman AE. Walking to health. Sports Medicine. 1997;23(5):306-32.

100. Mutrie N, Carney C, Blamey A, Whitelaw A, Crawford F, Aitchison T. Can active commuting increase quality of life? Three-month results from a randomized control trial. Journal of Sports Sciences. 2000;18(1):18-9.

101. Nathan N, Elton B, Babic M, McCarthy N, Sutherland R, Presseau J, et al. Barriers and facilitators to the implementation of physical activity policies in schools: A systematic review. Preventive medicine. 2018;107:45-53.

102. Nathan N, Wiggers J, Bauman AE, Rissel C, Searles A, Reeves P, et al. A cluster randomised controlled trial of an intervention to increase the implementation of school physical activity policies and guidelines: study protocol for the physically active children in education (PACE) study. BMC public health. 2019;19(1):170.

103. Nguyen PN, Koh PP, Wong YD. Impacts of bicycle infrastructure: a case study in Singapore. Proceedings of the Institution of Civil Engineers - Municipal Engineer. 2015;168(3):186-98.

104. Ogilvie D, Griffin S, Jones A, Mackett R, Guell C, Panter J, et al. Commuting and health in Cambridge: a study of a 'natural experiment' in the provision of new transport infrastructure. BMC public health. 2010;10:703.

105. Panter J, Ogilvie D, iConnect c. Theorising and testing environmental pathways to behaviour change: natural experimental study of the perception and use of new infrastructure to promote walking and cycling in local communities. BMJ open. 2015;5(9):e007593.

106. Panter J, Heinen E, Mackett R, Ogilvie D. Impact of New Transport Infrastructure on Walking, Cycling, and Physical Activity. American Journal of Preventive Medicine. 2016;50(2):e45-e53.

107. Albery WB. Acceleration in Other Axes Affects +Gz Tolerance: Dynamic Centrifuge Simulation of Agile Flight. Aviation Space and Environmental Medicine. 2004;75(1):1-6.

108. Pedroso FE, Angriman F, Bellows AL, Taylor K. Bicycle Use and Cyclist Safety Following Boston’s Bicycle Infrastructure Expansion, 2009–2012. American Journal of Public Health. 2016;106(12):2171-7.

109. Peters P, Gold A, Abbott A, Contreras D, Keim A, Oscarson R, et al. A quasi-experimental study to mobilize rural low-income communities to assess and improve the ecological environment to prevent childhood obesity. BMC public health. 2016;16:376.

110. Reger-Nash B, Bauman A, Booth-Butterfield S, Cooper L, Smith H, Chey T, et al. Wheeling walks - Evaluation of a media-based community intervention. Family & Community Health. 2005;28(1):64-78.

111. Robinson JC, Temple ML, Duck A, Klamm M. Feasibility and effectiveness of two built environmental interventions on physical activity among 3-5-year-old preschoolers. Journal for specialists in pediatric nursing : JSPN. 2019;24(3):e12262.

112. Rodriguez NM, Arce A, Kawaguchi A, Hua J, Broderick B, Winter SJ, et al. Enhancing safe routes to school programs through community-engaged citizen science: two pilot investigations in lower density areas of Santa Clara County, California, USA. BMC public health. 2019;19(1):256.

113. Roemmich JN, Beeler JE, Johnson L. A microenvironment approach to reducing sedentary time and increasing physical activity of children and adults at a playground. Preventive medicine. 2014;62:108-12.

114. Rowland D, DiGuiseppi C, Gross M, Afolabi E, Roberts I. Randomised controlled trial of site specific advice on school travel patterns. Archives of disease in childhood. 2003;88(1):8-11.

115. Sahlqvist S, Goodman A, Cooper AR, Ogilvie D, iConnect c. Change in active travel and changes in recreational and total physical activity in adults: longitudinal findings from the iConnect study. The international journal of behavioral nutrition and physical activity. 2013;10:28.

116. Saito Y, Oguma Y, Tanaka A, Kamada M, Inoue S, Inaji J, et al. Community-wide physical activity intervention based on the Japanese physical activity guidelines for adults: A non-randomized controlled trial. Preventive medicine. 2018;107:61-8.

117. Salinas JJ, McDaniel M, Parra-Medina D. The Role of Social Support and the Neighborhood Environment on Physical Activity in Low-income, Mexican-American Women in South Texas. Journal of preventive medicine and public health = Yebang Uihakhoe chi. 2018;51(5):234-41.

118. Salopuro T, Saaristo T, Oksa H, Puolijoki H, Saltevo J, Ebeling T, et al. Population-level effects of a national diabetes prevention programme on the prevalence of obesity: FIN-D2D. Obesity Reviews. 2010;11(SUPPL. 1):410-1.

119. Sayers SP, LeMaster JW, Thomas IM, Petroski GF, Ge B. A Walking School Bus Program: Impact on Physical Activity in Elementary School Children in Columbia, Missouri. American Journal of Preventive Medicine. 2012;43(5):S384-S9.

120. Slater SJ, Tarlov E, Jones K, Matthews SA, Wing C, Zenk SN. Would increasing access to recreational places promote healthier weights and a healthier nation? Health & place. 2019;56:127-34.

121. Smith BJ, MacKenzie-Stewart R, Newton FJ, Haregu TN, Bauman A, Donovan RJ, et al. A longitudinal study examining uptake of new recreation infrastructure by inactive adults. The international journal of behavioral nutrition and physical activity. 2019;16(1):59.

122. Solomon E, Rees T, Ukoumunne OC, Metcalf B, Hillsdon M. The Devon Active Villages Evaluation (DAVE) trial of a community-level physical activity intervention in rural south-west England: a stepped wedge cluster randomised controlled trial. The international journal of behavioral nutrition and physical activity. 2014;11:94.
