# Supplementary material for: Infrastructure, policy and regulatory interventions to increase physical activity to prevent cardiovascular diseases and diabetes: a systematic review
Source: BMC Public Health. 2023 Jan 16;23:112. doi: 10.1186/s12889-022-14841-y (PMC9841711; doi:10.1186/s12889-022-14841-y)
Supplement: Supplementary file 3 — Additional file 3. Characteristics of included studies. [file 12889_2022_14841_MOESM3_ESM.docx]

**Characteristics of included studies**

# Benjamin Neelon 2015

| **Methods** | **Study design:** CBA  **Study grouping:**  **Study aim or objective:** To evaluate the effect of a community-based intervention, initiated by residents, to promote PA and decrease obesity.  **Unit of allocation or exposure:** Town-level  **Number of intervention sites:** 1  **Number of control sites:** 1  **Methods for selecting intervention and control sites:** The intervention took place in a small town in central North Carolina - Mebane. Intervention was designed and implemented by community members, who then approached the researchers to evaluate impact. Researchers then recruited a second town with similar geographical and sociodemographic characteristics to serve as the comparison community. The comparison community was located 96 miles from the intervention town, which was close enough to allow for travel for data collection, but provided enough distance to avoid contamination.  **How were participants recruited and sampled within the study sites?:** "We sent invitation letters to nearly 2000 parents of elementary school children at three schools in each community. We invited parents who completed the survey to enroll their children in a sub study that included anthropometric and more objective PA assessment, with the goal of enrolling a small subset of 100 children ages 5–11 years per community."  **Pre-intervention time period (before intervention start):**  **Post-intervention period (start and end date or year it was conducted):** Summer 2011 - Spring 2012  **Sample size justification and outcome used:** Sample size was dictated by available funding to conduct the in-depth, child-level assessments measuring PA and weight status.  **How was missing data handled?:** 52 children (30 from intervention and 22 from control groups) who did not meet accelerometer compliance at 1 year follow-up were excluded from the analyses.  **Time points of data collection:** 2 - spring 2011, spring 2012 |
| --- | --- |
| **Participants** | **Baseline Characteristics**  Intervention   - *Age*: Mean (SD): 7.8 (1.8) - *Place of residence*: NR - *Sex*: n (%) female: 35 (54.7) - *Ethnicity and language*: n (%) white: 49 (76.6), African American: 12 (18.8), other: 3 (4.7), Hispanic/Latino: 4 (6.3) - *Occupation*: NR - *Education*: [of parents] n (%) ≤ High school: 2 (3.1), Vocational or some college: 14 (21.9), ≥ College graduate: 48 (75.0) - *Socioeconomic status*: Yearly household income - n (%): ≤$30,000: 11 (17.2), $30,001–$90,000: 27 (42.2), ≥$90,001: 26 (40.6) - *Social capital*: NR   Control   - *Age*: Mean (SD): 8.3 (1.9) - *Place of residence*: NR - *Sex*: n (%) female: 15 (37.5) - *Ethnicity and language*: n (%) white: 34 (85.0) , African American: 5 (12.5) , other: 1 (2.5), Hispanic/Latino: 0 (0.0) - *Occupation*: NR - *Education*: [of parent] n (%) ≤ High school: 5 (12.5) , Vocational or some college: 14 (35.0), ≥ College graduate: 21 (52.5) - *Socioeconomic status*: Yearly household income - n (%): ≤$30,000: 14 (35.0), $30,001–$90,000: 20 (50.0), ≥$90,001: 6 (15.0) - *Social capital*: NR   Overall   - *Age*: NR - *Place of residence*: NR - *Sex*: NR - *Ethnicity and language*: NR - *Occupation*: NR - *Education*: NR - *Socioeconomic status*: NR - *Social capital*: NR   **Included criteria:** "To participate, children needed to attend an elementary school in one of our two communities, and parents needed to be 18 years or older and speak and read English."  **Excluded criteria:** NR  **Pretreatment:** "Communities did not differ in child race or age at baseline, but children in the intervention community had lower BMIz-scores at baseline (0.6 vs. 1.2; p = 0.03). Parents in the intervention community reported higher family incomes (40.6% intervention vs.15.0% comparison reported ‡ $90,001 per year; p = 0.01) and parental education (75.0% intervention vs. 52.5% comparison graduated college; p = 0.03; Table 1)."  **How were baseline differences assessed?:** Wilcoxon’s rank-sum tests for continuous variables and Pearson’s exact chi-square tests for categorical variables.  **Total number in each relevant group pre-intervention:** Intervention: 64 Comparison: 40  **Total number randomised per relevant group:** NA  **Total number completed and analysed per relevant group:** Intervention: 34 Comparison: 18  **Attrition per relevant group:** Overall attrition: 50%. n=30 of 64 children excluded from analysis in intervention group (46%) and n=22 of 40 excluded from analysis in control group (55%) |
| **Interventions** | **Intervention Characteristics**  Intervention   - *Type of intervention (e.g. infrastructure, policy or both)*: Infrastructure - *Description of main intervention*: "...sidewalks, crosswalks, and walking trails installed throughout town.... The built environment changes included adding new sidewalks and crosswalks in the downtown area, creating a walking path throughout the town with 60 colorful and engraved stone pavers marking distance, and encouraging enhancements and clean-up efforts for all local parks and existing trails. The new sidewalks and crosswalks were also linked to the walking path to guide walkers and runners through town" - *Description of additional intervention components*: "(1) walking and running clubs in the elementary schools for children and in the community for families, (2) portable play equipment provided to low-income families through home delivery food assistance programs. Walking and running clubs were established in the elementary schools in the district and were open to all children who attended those schools. Additionally, free community exercise programs were offered to children and adults at the local recreation center. The Mebane on the Move intervention also included an annual 5-km run that takes place within the downtown area and is open to all residents for a nominal fee. Funds raised through this run help sustain intervention activities." - *Duration of intervention*: The infrastructure-related interventions should be seen as permanent after the point of implementation (spring 2011). - *How was the intervention implemented?*: The intervention was designed by community members. A group of concerned residents raised funds through local, private donations to launch the Mebane on the Move intervention in 2011. Residents engaged business leaders, faith communities, schools, government officials, and local health professionals to promote PA throughout the community. Funds raised through this run help sustain intervention activities. Not all of the planned components of the Mebane on the Move intervention took place as intended. However, some crosswalks and bike lanes were not installed owing to funding limitations and problems obtaining approval from the state department of transportation, and improvements to park equipment and greenery were not implemented due to funding constraints. - *Co-interventions*: NR - *Where was the intervention implemented?*: In the town, elementary schools, recreation centres, with all residents - *Resource requirements to replicate the intervention*: NR - *Economic indicators*: "They did not receive any funding outside of the town or community members, and all activities were driven exclusively by the needs of the community."   Control   - *Type of intervention (e.g. infrastructure, policy or both)*: No intervention - *Description of main intervention*: No intervention - *Description of additional intervention components*: No intervention - *Duration of intervention*: No intervention - *How was the intervention implemented?*: No intervention - *Co-interventions*: The schools within the comparison community had strong obesity-prevention initiatives that launched near the start of the study. Two of the schools in the comparison community started promoting healthier eating and active play to the parents of children enrolled. - *Where was the intervention implemented?*: No intervention - *Resource requirements to replicate the intervention*: No intervention - *Economic indicators*: No intervention |
| **Identification** | **Sponsorship source:** "This study was supported, in part, by a grant from Active Living Research, a national program of the Robert Wood Johnson Foundation (RWJF; ID no. 68593)."  **Country:** USA  **Setting:** Small towns in central North Carolina  **Author’s name:** Sara E. Benjamin Neelon  **Email:** sara.benjamin@duke.edu  **Declaration of interests stated?:** Yes. "No competing financial interests exist."  **Study or programme name or acronym:** Mebane on the move  **Type of record:** Journal article  **Specify any documents relevant to this study:** NA |
| **Notes** | **n/a** |

## Risk-of-bias table

| **Bias** | **Authors' judgement** | **Support for judgement** |
| --- | --- | --- |
| Random sequence generation (selection bias) | High risk | Study is a CBA, the control site was chosen because it had "similar geographical and sociodemographic characteristics" to the intervention site. |
| Allocation concealment (selection bias) | High risk | Study is a CBA; those conducting the research chose the control site. |
| Baseline characteristics similar | Low risk | There were imbalances in baseline characteristics between the 2 groups but analysis was adjusted for baseline values and co-variates. |
| Baseline outcome measurements similar | Low risk | The "intervention community had lower BMI z-scores at baseline (0.6 vs. 1.2; p = 0.03)" compared to the control community. Children in both groups engaged in similar average number of minutes of moderate-to-vigorous physical activity (MVPA) at baseline. Analysis was adjusted for baseline values. |
| Blinding of participants and personnel (performance bias) | Low risk | It was not possible to blind participants and personnel (who was the community) in this type of intervention. However, this is unlikely to affect participant or community members’ behaviors. |
| Blinding of outcome assessment (detection bias) | Low risk | Data collectors were blinded to study aims but it is not clear whether they were blinded to intervention assignment. This is unlikely due to the nature of the intervention. However, outcomes were objective and therefore unlikely to have been affected by lack of blinding. |
| Protection against contamination | Low risk | "The comparison community was located 96 miles from the intervention town, which was close enough to allow for travel for data collection, but provided enough distance to avoid contamination." |
| Incomplete outcome data (attrition bias) | High risk | There were substantial missing data due to attrition in both groups (intervention group: 53.1%; control group 45%), and it appears that these were simply excluded from the analysis. Authors do address this in the text (see excerpt), but this still may have introduced bias. "When we compared baseline values of children who completed a follow-up assessment to those who withdrew from the study, we did not observe differences in MVPA levels (3.9 vs. 4.2 minutes per hour; p = 0.42), BMI z-score (0.6 vs. 1.0 units; p = 0.12) or any other covariates included in the analyses." |
| Selective reporting (reporting bias) | Low risk | No evidence of selective outcome reporting. |
| Other bias | High risk | Events occurring at the control site were potentially also positively influencing PA and obesity around the same time as the intervention. This would, however, have introduced bias towards the null: "the schools within the comparison community had strong obesity-prevention initiatives that launched near the start of the study. Two of the schools in the comparison community started promoting healthier eating and active play to the parents of children enrolled." Misclassification bias - Researchers identified the control community. Residents of the community where the intervention was taking place could have been sampled but not actively taking part in the study. Measurement bias - unlikely: anthropometric data was collected using appropriate methods. PA data was based on one week of accelerometer data, measured using a validated tool. Incorrect analysis - n/a. |

# Bohn Goldbaum 2013

| **Methods** | **Study design:** CBA  **Study grouping:** Parallel group  **How was missing data handled?:** Missing data NR.  **How were participants recruited and sampled within the study sites?:** For direct observation: "Each park was divided into target areas for observation. The children’s playground was one of four pre-upgrade observation areas in the intervention park." "Staff scanned target areas from left to right every thirty minutes during the observation periods and recorded park users’ gender, age, activity type, and activity intensity." For the park user interviews: "Post-upgrade intercept surveys were conducted with consenting park users aged 16 years or older who were accompanied by children under 13 years of age." "Interviews were conducted by one of the authors (EB) between 10:30 and 17:00 hours on the same days during which direct observation was performed. Interviews were conducted throughout the park space, one interview per target area, rotating through all target areas for each data collection period. In the event of a refusal, another park user within the same area was approached; if no users participated in a given target area, data collection directly continued in the next target area."  **Methods for selecting intervention and control sites:** Intervention park was purposefully selected as it was targeted for un upgrade as part of "Sydney’s recreational strategy aims to “increase participation by residents in physical activity thereby enhancing their health and well-being”. The comparison park (Park B) was chosen for its similarities to Park A and is located in a nearby urban neighborhood within the city of Sydney.  **Number of control sites:** 1  **Number of intervention sites:** 1  **Post-intervention period (start and end date or year it was conducted):** 9 months (from end of upgrade to endline evaluation)  **Pre-intervention time period (before intervention start):** May 2007 (baseline) to approx September 2008 (Park renovation/upgrade completed)  **Sample size justification and outcome used:** NR  **Study aim or objective:** "Using a quasi-experimental design, the aims of the current study were to (1) determine if an urban park renovation that included playground alterations affects usage and PA in children within playgrounds; (2) determine whether playground alterations affects parents’ self-report of playground visitation post intervention, in association with proxy reports of children’s PA levels; and (3) assess parental impressions of environmental features (e.g., equipment safety) of the renovated playground post intervention."  **Time points of data collection:** Two: Baseline (May 2007) and endline (May 2009)  **Unit of allocation or exposure:** Park |
| --- | --- |
| **Participants** | **Baseline Characteristics**  Intervention   - *Age*: NR - *Place of residence*: "lower socioeconomic urban neighborhood within the city of Sydney" - *Sex*: NR - *Ethnicity and language*: NR - *Occupation*: NR - *Education*: NR - *Socioeconomic status*: parks located in "lower socioeconomic status" areas - *Social capital*: NR   Control   - *Age*: - *Place of residence*: "lower socioeconomic urban neighborhood within the city of Sydney" - *Sex*: NR - *Ethnicity and language*: NR - *Occupation*: NR - *Education*: NR - *Socioeconomic status*: parks located in "lower socioeconomic status" areas - *Social capital*: NR   Overall   - *Age*: NR - *Place of residence*: "lower socioeconomic urban neighborhood within the city of Sydney" - *Sex*: NR - *Ethnicity and language*: NR - *Occupation*: NR - *Education*: NR - *Socioeconomic status*: parks located in "lower socioeconomic status" areas - *Social capital*: NR   **Included criteria:** NR. "Systematic observations of playground visitors aged 2–12 years." and "Post-upgrade intercept surveys were conducted with consenting park users aged 16 years or older who were accompanied by children under 13 years of age."  **Excluded criteria:** NR  **Pretreatment:** "children’s playground usage at baseline was lower in the intervention Park A compared to comparison Park B (likelihood ratio test: p = 0.03). This difference could be due to a childcare facility being located next to the comparison park." "At baseline, fewer children performed MVPA in Park A than in Park B (p = 0.02)."  **Attrition per relevant group:** Two cross-sectional samples used. No missing data reported.  **How were baseline differences assessed?:** "likelihood ratio test"  **Total number completed and analysed per relevant group:** NR  **Total number in each relevant group pre-intervention:** NR  **Total number randomised per relevant group:** NR |
| **Interventions** | **Intervention Characteristics**  Intervention   - *Type of intervention (e.g. infrastructure, policy or both)*: Infrastructure - *Description of main intervention*: Upgrade of playgrounds in a park. "Sydney’s recreational strategy aims to “increase participation by residents in physical activity thereby enhancing their health and well-being” [37]and its objectives for this park renovation included provision for recreation and children’s play by “amenities to facilitate use and enjoyment . . .including but not limited to children’s play equipment” [38]. Specific changes in the park renovation included upgrading paths and adding new greenery, lighting, and facilities (e.g., park furniture). More green space was created by opening the adjacent sports field to public use, thus increasing the accessible park size from 2.2 to 4.6 ha." "The new children’s playgrounds are three unfenced areas dispersed throughout the park. The playgrounds incorporate the city of Sydney’s design mandate to include public art, an aboriginal theme, and, following community consultation, a water play feature. One area includes two swings and a climbing structure designed for use primarily by young children, with wood chips as flooring.For children up to 7 years old, play sculptures with a soft-fall surface and an interactive water feature on cement were installed in a second area, as shown in Figure 2. A third play area, for older children, is comprised of three climbing poles with wood chips as flooring. As part of the upgrade, a large cement area for basketball and skating was installed adjacent to the poles." - *Description of additional intervention components*: NR - *Duration of intervention*: 9 months (From final upgrade to endline evaluation) - *How was the intervention implemented?*: NR - *Co-interventions*: There were also broader changes across the intervention park besides the playground upgrade "Secondly, changes in playground layout resulted in observation scan areas at follow-up that include both play equipment and other park amenities. This complicated the comparison of playground usage and PA levels." - *Where was the intervention implemented?*: Urban parks in Sidney, Australia - *Resource requirements to replicate the intervention*: NR - *Economic indicators*: NR   Control   - *Type of intervention (e.g. infrastructure, policy or both)*: No intervention - *Description of main intervention*: No upgrade. "The comparison park was chosen for its similar size(4.2 ha) and type of park. Its playground is similar to the pre-renovation playground in Park A: a fenced area with soft-fall flooring and containing multifunction apparatuses, swings, slides, and other equipment as shown in Figure 3. The comparison playground had a shade net and equipment pieces were grouped into toddler and school-age sections." - *Description of additional intervention components*: n/a - *Duration of intervention*: n/a - *How was the intervention implemented?*: n/a - *Co-interventions*: None reported - *Where was the intervention implemented?*: Urban parks in Sidney, Australia - *Resource requirements to replicate the intervention*: NR - *Economic indicators*: NR |
| **Identification** | **Sponsorship source:** "This study was funded by the Cluster for Physical Activity and Health, Prevention Research Collaboration, School of Public Health, the University of Sydney."  **Country:** Australia  **Setting:** Parks in lower socioeconomic urban neighborhoods  **Comments:**  **Author’s name:** Erika E. Bohn-Goldbaum  **Institution:**  **Email:** erika.goldbaum@sydney.edu.au  **Address:**  **Declaration of interests stated?:** "The authors declare that they have no conflict of interests."  **Specify any documents relevant to this study:** n/a  **Study or programme name or acronym:** n/a  **Type of record:** Journal article |
| **Notes** |  |

## Risk-of-bias table

| **Bias** | **Authors' judgement** | **Support for judgement** |
| --- | --- | --- |
| Random sequence generation (selection bias) | High risk | This was a CBA study; no randomisation carried out |
| Allocation concealment (selection bias) | High risk | This was a CBA study |
| Baseline characteristics similar | Unclear risk | No baseline characteristics reported, although the authors report that the comparison park was selected for its similarity with the intervention park. |
| Baseline outcome measurements similar | High risk | There was lower observed playground use and MVPA in the intervention park compared to the control park at baseline. "This difference could be due to a childcare facility being located next to the comparison park." |
| Blinding of participants and personnel (performance bias) | Low risk | There was no blinding but this would not influence the delivery of this type of intervention. |
| Blinding of outcome assessment (detection bias) | High risk | Blinding was not possible. Outcomes were assessed by observation by research staff who may have been influenced by knowledge of which park was renovated. |
| Protection against contamination | Low risk | Allocation by park and contamination is unlikely. |
| Incomplete outcome data (attrition bias) | Unclear risk | Authors do not report any missing data or exclusions of data in analyses for any reason. Numbers of participants not reported for the baseline and endline cross-sectional samples. |
| Selective reporting (reporting bias) | Unclear risk | Study does not mention a protocol. |
| Other bias | Unclear risk | *Misclassification bias*: low risk; park visitors observed by trained staff.  *Measurement bias*: Unclear risk; "While the observation periods occurred at predetermined times throughout the day totaling six hours per day for 14 days, this period may neither be representative of total playground use nor capture secular variations in usage and PA. Our inter-rater agreement for PA levels (sedentary/light versus MVPA) was lower than that reported in a previous study which used a similar tool [40], but also higher at follow-up. This could be because more people were doing light/sedentary activities at follow-up; there were relatively few PA observations classified at MVPA level. Alternatively, there may be a greater error in our baseline survey in relation to MVPA assessment, and so our findings for decline may be biased" .  *Incorrect analysis*: n/a |

# Branas 2011

| **Methods** | **Study design:** Interrupted time series study  **Study grouping:**  **How was missing data handled?:** NR  **How were participants recruited and sampled within the study sites?:** Routinely collected data was used to assess outcomes in the areas affected.  **Methods for selecting intervention and control sites:** Intervention vacant lots were those that were greened by the Pennsylvania Horticultural Society (PHS), identified through a master database of vacant lots. Control vacant lots were those lots that could have been chosen (eligible) for greening but were never greened; these were selected randomly and matched to intervention lots 3:1. There were 2 different pools of matched controls.  **Number of control sites:** 13,308 vacant lots. 49,690 vacant lots not greened but because none of the study’s final conclusions markedly differed by control group type and because control lots with open violations were a better statistical match to greened lots (in terms of area, age, and unemployment), only findings obtained using this control group are reported.  **Number of intervention sites:** 4,436 greened vacant lots  **Post-intervention period (start and end date or year it was conducted):** Varies depending on the lot  **Pre-intervention time period (before intervention start):** "The pre-greening period for each treated lot was defined as the years prior to the year that the lot was greened/treated (from 1999 to 2008). This same pre-period was assigned to the 3 randomly selected, matched control lots."  **Sample size justification and outcome used:** NR  **Study aim or objective:** "The authors conducted a decade-long difference-indifferences analysis of the impact of a vacant lot greening program in Philadelphia, Pennsylvania, on health and safety outcomes."  **Time points of data collection:** T1=1998, T2 =2000, T3= 2002, T4= 2004, T5= 2006, and T6 = 2008 waves  **Unit of allocation or exposure:** vacant lots |
| --- | --- |
| **Participants** | **Baseline Characteristics**  Intervention = treated or greened vacant lots   - *Age*: Median Age of Residents per Square Mile in years - 36.42 - *Place of residence*: Philadelphia (West, North West, North and South) - *Sex*: NR - *Ethnicity and language*: Median No. of Hispanic Residents per Square Mile -11.53. Median number of Black residents per square mile: 491.29 - *Occupation*: Median No. of Unemployed Residents per Square Mile -20.14 - *Education*: Median No. of College-Educated Residents per Square Mile -71.96 - *Socioeconomic status*: Median No. of Residents in Poverty per Square Mile = 301.94 - *Social capital*: NR   Control = non-treated/ non-greened control vacant lots   - *Age*: Median Age of Residents per Square Mile in years - 36.98 - *Place of residence*: Philadelphia (West, North West, North and South) - *Sex*: NR - *Ethnicity and language*: Median No. of Hispanic Residents per Square Mile -12.74. Median no. of Black residents per Square Mile: 467.85 - *Occupation*: Median No. of Unemployed Residents per Square Mile -20.39 - *Education*: Median No. of College-Educated Residents per Square Mile -87.57 - *Socioeconomic status*: Median No. of Residents in Poverty per Square Mile = 313.63 - *Social capital*: NR   Overall   - *Age*: NR - *Place of residence*: NR - *Sex*: NR - *Ethnicity and language*: NR - *Occupation*: NR - *Education*: NR - *Socioeconomic status*: NR - *Social capital*: NR   **Included criteria:** "Vacant lots were defined as abandoned parcels of open land with no buildings on them."  **Excluded criteria:** Vacant properties that contained buildings and other structures.  **Pretreatment:** At baseline: Median annual household income, median no of black residents, median no of Hispanic residents.  **Attrition per relevant group:** NA. Data is from routinely collected data. "A total of 68 treated vacant lots became inactive before the study period ended (housing or other structures were developed on them or they became inaccessible or unmaintainable), no longer functioned in the same way as actively greened lots, and were recoded as untreated for the years they were inactive."  **How were baseline differences assessed?:** NR  **Total number completed and analysed per relevant group:** NR  **Total number in each relevant group pre-intervention:** Greened vacant lots (intervention) - n=4,436 Control vacant lots - n=13,308  **Total number randomised per relevant group:** |
| **Interventions** | **Intervention Characteristics**  Intervention = treated or greened vacant lots   - *Type of intervention (e.g. infrastructure, policy or both)*: Infrastructure - *Description of main intervention*: Abandoned vacant lots in Philadelphia were greened, using a consistent treatment protocol that involved removing trash and debris, grading the land, planting grass and trees to create a park-like setting, and installing low wooden post-and-rail fences around each lot’s perimeter to show that the lot was cared for and deter illegal dumping. Multiple times each year, PHS returned to each treated lot to perform basic maintenance activities, such as mowing the grass, tending trees, or repairing fences. - *Description of additional intervention components*: No additional components - *Duration of intervention*: 10 years; Study evaluated the program between 1999 and 2008 - *How was the intervention implemented?*: The Pennsylvania Horticultural Society (PHS), along with other community and municipal partners, directs a program to clean, green, and maintain abandoned vacant lots in Philadelphia, Pennsylvania." "Multiple times each year, PHS returned to each treated lot to perform basic maintenance activities,such as mowing the grass, tending trees, or repairing fences." - *Co-interventions*: None reported - *Where was the intervention implemented?*: The study was conducted within 4 of the 5 sections of Philadelphia. The Northeast section was excluded because a trivial number of vacant lots (<0.2%) were greened there. - *Resource requirements to replicate the intervention*: NR - *Economic indicators*: NR   Control = non-treated/ non-greened control vacant lots   - *Type of intervention (e.g. infrastructure, policy or both)*: No intervention - *Description of main intervention*: No treating of greenspaces/lots - *Description of additional intervention components*: No intervention - *Duration of intervention*: No intervention - *How was the intervention implemented?*: No intervention - *Co-interventions*: No intervention - *Where was the intervention implemented?*: No intervention - *Resource requirements to replicate the intervention*: No intervention - *Economic indicators*: No intervention |
| **Identification** | **Sponsorship source:** "This work was completed in part with funding from the Centers for Disease Control and Prevention (grant U49CE001093) and the National Institutes of Health (grant R01AA016187)."  **Country:** USA  **Setting:** Urban area  **Comments:**  **Author’s name:** Charles C. Branas  **Institution:**  **Email:** cbranas@upenn.edu  **Address:**  **Declaration of interests stated?:** Yes. "Conflict of interest: none declared."  **Specify any documents relevant to this study:**  **Study or programme name or acronym:**  **Type of record:** Journal article |
| **Notes** | *Solange Durao* on 21/05/2020 23:24 **Select** screened in previous round was excluded: ineligible intervention (vacant plots and violence) |

## Risk-of-bias table

| **Bias** | **Authors' judgement** | **Support for judgement** |
| --- | --- | --- |
| Random sequence generation (selection bias) | High risk | CBA study |
| Allocation concealment (selection bias) | High risk | CBA study |
| Baseline characteristics similar | Low risk | Some characteristics differed between the groups but these were adjusted for in the DID analyses. |
| Baseline outcome measurements similar | Unclear risk | Baseline outcome measures not reported. |
| Blinding of participants and personnel (performance bias) | Low risk | Outcomes are based on routinely collected data and participants unaware of study. |
| Blinding of outcome assessment (detection bias) | Low risk | Outcomes are based on routinely collected data. |
| Protection against contamination | Unclear risk | Unclear whether contamination could have occurred. 68 plots initially identified as greened were recoded as inactive during the intervention. |
| Incomplete outcome data (attrition bias) | Unclear risk | The authors did not specify the number of cites/lots analysed compared to baseline. |
| Selective reporting (reporting bias) | Low risk | All relevant outcomes in the methods section are reported in the results section (crime and health factors). |

# Brown 2016

| **Methods** | **Study design:** CBA  **Study grouping:**  **How was missing data handled?:** "Because over 5% of residents (n=75) did not report income, missing income values were imputed by multiple regression, with random residuals chosen from complete cases."  **How were participants recruited and sampled within the study sites?:** "Initial mail invitation was followed by door-to-door recruitment visits at varied times of day and week" of "residents living Near (=800 m) and Far (801–2000 m) from the complete street renovation in Salt Lake City, Utah." Those that met inclusion criteria and agreed to participate were included. How sampling was done is NR.  **Intervention period (start and end date or year it was conducted):** April 2013. No intervention end date because intervention was permanent infrastructure changes.  **Methods for selecting intervention and control sites:** Intervention - residents living NEAR infrastructure changes, Control - residents living FAR from infrastructure changes. "Based on past research and planning practice, we defined residents as living Near to new light rail lines when they lived within 800 m. We also conducted sensitivity tests using shorter (600 m) and longer (1000 m) distances to acknowledge that exposure to the intervention might be optimal at distances other than 800 m."  **Number of control sites:** 1  **Number of intervention sites:** 1  **Pre-intervention time period (before intervention start):** March-December of 2012  **Sample size justification and outcome used:** "Sample size met power calculation requirements (i.e. testing the conservative assumption that use increases from 5% to 10%, which required N=210 for 80% power for an alpha of .05)”. Outcome: "proportion of nearby residents who make use of the street for walking, using transit, or biking."  **Study aim or objective:** "The current study evaluates whether a complete street intervention in Salt Lake City, Utah that involves a new light rail extension, better and more complete bike paths, and wider more aesthetically pleasing sidewalks increases the proportion of nearby residents who make use of the street for walking, using transit, or biking."  **Time points of data collection:** 2: Baseline: March-December 2012, Follow-up: May–November 2013  **Unit of allocation or exposure:** Individuals |
| --- | --- |
| **Participants** | **Baseline Characteristics**  Intervention   - *Age*: NR - *Place of residence*: Urban area, Salt Lake City - *Sex*: Proportion female, proportion (SD): 0.51 (0.50) - *Ethnicity and language*: White race, proportion (SD): 0.64 (0.48) - *Occupation*: Employed, proportion (SD): 0.63 (0.48) - *Education*: NR - *Socioeconomic status*: Have a car, proportion (SD): 0.83 (0.37). Household income in thousands of dollars, mean (SD): 36.6 (27.88) - *Social capital*: NR   Control   - *Age*: NR - *Place of residence*: Urban area, Salt Lake City - *Sex*: Proportion female, proportion (SD): 0.51 (0.50) - *Ethnicity and language*: White race, proportion (SD): 0.74 (0.44) - *Occupation*: Employed, proportion (SD): 0.74 (0.44) - *Education*: NR - *Socioeconomic status*: Have a car, proportion (SD): 0.92 (0.27). Household income in thousands of dollars, mean (SD): 47.59 (35.15) - *Social capital*: NR   Overall   - *Age*: Age in years, mean, SD: 41.72 (14.77). Proportion of households with children (SD): 0.41 (0.49) - *Place of residence*: Urban area, Salt Lake City - *Sex*: Proportion Female (SD): 0.51 (0.50) - *Ethnicity and language*: Hispanic ethnicity, proportion (SD): 0.24 (0.43). - *Occupation*: NR - *Education*: College graduation status, proportion (SD): 0.37 (0.48) - *Socioeconomic status*: Household size, mean (SD): 2.99 (1.79). Renter status, proportion (SD): 0.49 (0.50) - *Social capital*: NR   **Included criteria:** "...adults ≥ 18 who were not pregnant, spoke English or Spanish, could walk for a few blocks, could complete the informed consent process from the first author’s Institutional Review Board, wear the equipment, and who planned to stay in the neighborhood at least a year". Additionally, in Brown 2014: "... able to walk a few blocks and complete surveys and wear the devices. To be retained, participants needed to complete surveys and register ≥3 days of ≥10-hour accelerometer wear"  **Excluded criteria:** NR  **Pretreatment:** "Residents from Near and Far areas were not significantly different for most of the demographic variables". "Near residents were significantly less likely to report being white, married, or employed. They also were less likely to have cars and they reported lower household incomes."  **Attrition per relevant group:** Overall n=374 (n=340 participants moved away or become ineligible and n=34 refused to participate at follow-up). Attrition per group not reported.  **How were baseline differences assessed?:** "Tests included chi-square tests of dummy variables representing categories and independent t-tests for continuous variables. Km means kilometer."  **Total number completed and analysed per relevant group:** Overall: n=536. Number per group not reported.  **Total number enrolled per relevant group:** Overall: n=910. Number per group not reported.  **Total number randomised per relevant group:** N/A |
| **Interventions** | **Intervention Characteristics**  Intervention   - *Type of intervention (e.g. infrastructure, policy or both)*: Infrastructure - *Description of main intervention*: "The street improvements completed a sporadic bike lane and widened sections of it so that it is now designated a “high comfort” bike lane on the city bike map. A light rail line (6.7 miles 10.7 km) was extended from downtown westward to the airport." The study is focused along the eastern section, which included 5 new rail stops spread across 3.5 km within walking distances of residents. "Complete street improvements also included narrowed automotive lanes and wider and better lit sidewalks." - *Description of main intervention components*: NR - *Duration of intervention*: From April 2013; interventions were permanent - *How was the intervention implemented?*: NR - *Co-interventions*: None reported - *Where was the intervention implemented?*: Salt Lake City, Utah: light rail line from downtown westward to the airport; 5 new rail lines on easter section. "The complete street corridor is fronted by multiple commercial and office areas; some multifamily rental, condominium, and hotel lodging; the state fair park; some industrial sites; and a variety of services." - *Resource requirements to replicate the intervention*: NR - *Economic indicators*: NR   Control   - *Type of intervention (e.g. infrastructure, policy or both)*: No intervention - *Description of main intervention*: No intervention - *Description of main intervention components*: No intervention - *Duration of intervention*: No intervention - *How was the intervention implemented?*: No intervention - *Co-interventions*: No intervention - *Where was the intervention implemented?*: No intervention - *Resource requirements to replicate the intervention*: No intervention - *Economic indicators*: No intervention |
| **Identification** | **Sponsorship source:** "The project described was supported (in part) by grant number CA157509 from the National Cancer Institute at the National Institutes of Health and the Robert Wood Johnson Foundation."  **Country:** USA  **Setting:** City in Salt Lake City, Utah.  **Comments:**  **Author’s name:** Barbara B Brown  **Institution:**  **Email:** Barbara.brown@fcs.utah.edu.  **Address:**  **Declaration of interests stated?:** NR  **Specify any documents relevant to this study:** Miller 2015 - related evaluation, Brown 2014 - baseline characteristics  **Study or programme name or acronym:** Moving Across Places Study (MAPS)  **Type of record:** Journal article |
| **Notes** |  |

## Risk-of-bias table

| **Bias** | **Authors' judgement** | **Support for judgement** |
| --- | --- | --- |
| Random sequence generation (selection bias) | High risk | This is a CBA; randomisation was not done. |
| Allocation concealment (selection bias) | High risk | There was no allocation concealment. Allocation to near/intervention or far/control group depended on distance of resident to street with infrastructure changes, and distance was determined a priori. |
| Baseline characteristics similar | Low risk | There were baseline imbalances, but these were adjusted for in the analysis. |
| Baseline outcome measurements similar | Low risk | Trip modes at baseline similar between the groups. |
| Blinding of participants and personnel (performance bias) | Low risk | Blinding was not possible, but it is unlikely that lack of blinding had an effect on the participant's or researcher behavior. |
| Blinding of outcome assessment (detection bias) | Low risk | Blinding was not possible, but it is unlikely that it would have affected outcomes measured. Outcomes were based on GPS and accelerometer data. |
| Protection against contamination | High risk | Exposure to the intervention was defined by distance. Participants in "far" group are potential users of the new infrastructure, thus being "exposed" to the intervention |
| Incomplete outcome data (attrition bias) | High risk | Many participants lost to follow up (n=374/910; 41%) due to moving away or by becoming ineligible. It is not clear what made participants ineligible. Authors do not report attrition per group. If these values were available, they could influence effect observed. |
| Selective reporting (reporting bias) | Low risk | Authors report on outcomes outlined in method’s section. |
| Other bias | Low risk | Misclassification bias: unlikely. Measurement bias - unlikely, all measurements followed appropriate methods. |

# Chapman 2014

| **Methods** | **Study design:** Controlled before-after study  **Study grouping:**  **How was missing data handled?:** "There were 13 individuals in the ACTIVE survey for whom age was unavailable and 1 with missing information for sex. These were not used in the analysis."  **How were participants recruited and sampled within the study sites?:** "Using sampling frames that were lists of addresses supplied to us by the four district councils, we randomly sampled households from the urbanised parts of the four districts...Although the original intention was to survey the same households in all 3 years, the response rate was lower than anticipated in the ﬁrst year. Some randomly sampled households were therefore added in 2012, with a higher number added in the intervention cities."  **Methods for selecting intervention and control sites:** Intervention sites: "Two local government areas, New Plymouth District Council and Hastings District Council, received programme initiatives from the New Zealand Transport Agency"; Control sites: "The control cities have similar demographics, economic proﬁles and climates to the intervention cities. These two cities were also suitable to act as controls because both were interested in encouraging active travel, but had not received additional funding for this purpose."  **Number of control sites:** 2  **Number of intervention sites:** 2  **Post-intervention period (start and end date or year it was conducted):** 2012-2013 (results from 2013)  **Pre-intervention time period (before intervention start):** 2011  **Sample size justification and outcome used:** NR  **Study aim or objective:** "Our ACTIVE study (Activating Communities to Improve Vitality and Equality) aimed to determine whether a combined central and local government initiative to construct cycling and walking infrastructure shifted travel from motorised (mainly driving or being a passenger in a motor vehicle) to active modes (walking and cycling), and more generally, increased levels of physical activity."  **Time points of data collection:** 3 (only two analysed)  **Unit of allocation or exposure:** City |
| --- | --- |
| **Participants** | **Baseline Characteristics**  Intervention   - *Age*: <20 y.o.: 15%; > 60 y.o.: 28% - *Place of residence*: NR - *Sex*: Female: 61% - *Ethnicity and language*: Maori: 18% - *Occupation*: NR - *Education*: NR - *Socioeconomic status*: Income < $10k: 18%; $10k-20k: 21%; > $40k: 33% - *Social capital*: NR   Control   - *Age*: <20 y.o.: 14%; > 60 y.o.: 33% - *Place of residence*: NR - *Sex*: Female: 62% - *Ethnicity and language*: Maori: 19% - *Occupation*: NR - *Education*: NR - *Socioeconomic status*: Income < $10k: 17%; $10k-20k: 26%; > $40k: 29% - *Social capital*: NR   Overall: NR  **Included criteria:** "All occupants of sampled households who were aged 10 years and over were invited to participate."  **Excluded criteria:** NR  **Pretreatment:** Based on Table 3 from Chapman: some slight differences spread across characteristics, including age and personal income.  **Attrition per relevant group:** "The response rate for the baseline ACTIVE household survey carried out over winter 2011 was 38% (the number of households with completed questionnaires, 400, divided by the number of eligible households, 1054). There was a 48.9% response rate in 2012 (from households who responded in 2011 plus those newly recruited in 2012) and a 55.3% response rate in the ﬁnal year."  **How were baseline differences assessed?:** Described in Table 3 (Chapman 2014)  **Total number completed and analysed per relevant group:** NR for individuals; for trips: Intervention: 1304; Control: 1151; for trip legs: Intervention: 2051; Control: 471  **Total number in each relevant group pre-intervention:** NR for individuals; for trips: Intervention: 2214; Control: 2202; for trip legs: Intervention: 2231; Control: 623  **Total number randomised per relevant group:** NA |
| **Interventions** | **Intervention Characteristics**  Intervention   - *Type of intervention (e.g. infrastructure, policy or both)*: Infrastructure - *Description of main intervention*: "The MCP aimed to deliver safe, urban environments that would encourage ‘novice users’ to walk or cycle to school or to work in fully integrated walking and cycling transport networks...The cities developed their programmes in line with local aims and were branded Let’s Go in New Plymouth and iWay in Hastings... Infrastructure upgrading and new investment, e.g. footpath renewal, new tracks, new cycle paths, lighting, bike stands, shared space or pathway projects, etc." - *Description of additional intervention components*: "Publicity and awareness campaigns for attitudes towards walking and cycling...Travel plan support through mapping and internet-based schemes." - *Duration of intervention*: Permanent after completion 2012/13 - *How was the intervention implemented?*: "In early 2010 the New Zealand Transport Agency sought tenders from local councils to establish the MCP, with the aim of encouraging the uptake of walking and cycling through structural changes and educational efforts. The MCP aimed to deliver safe, urban environments that would encourage ‘novice users’ to walk or cycle to school or to work in fully integrated walking and cycling transport networks [54]. Two North Island local governments, New Plymouth District Council and Hastings District Council were selected based on the central government’s criteria, and were funded $4.9million and $4.3 m. respectively from July 2010 to July2012. " - *Co-interventions*: NR - *Where was the intervention implemented?*: Plymouth and Hastings, two small cities - *Resource requirements to replicate the intervention*: "...funded $4.9 million and $4.3 m. respectively from July 2010 to July 2012." - *Economic indicators*: NR   Control   - *Type of intervention (e.g. infrastructure, policy or both)*: None ('...interest in encouraging active travel, but had not received additional funding for this purpose). - *Description of main intervention*: None - *Description of additional intervention components*: None - *Duration of intervention*: None - *How was the intervention implemented?*: None - *Co-interventions*: NR - *Where was the intervention implemented?*: None - *Resource requirements to replicate the intervention*: None - *Economic indicators*: None |
| **Identification** | **Sponsorship source:** "Financial and practical support from Hastings and New Plymouth District Councils, and the practical support of NZTA, are acknowledged with thanks. Financial support from the universities of Otago and Auckland, and the New Zealand Ministry of Business, Innovation and Employment ‘Resilient Urban Futures’ grant, which includes the ACTIVE (Activating Communities to Improve Vitality and Equality study), is also acknowledged."  **Country:** New Zealand  **Setting:** Plymouth and Hastings, two small cities  **Comments:**  **Author’s name:** Michael Keall  **Institution:**  **Email:** michael.keall@otago.ac.nz  **Address:**  **Declaration of interests stated?:** "The authors declare that they have no competing interests"  **Specify any documents relevant to this study:** Supplemental material available online  **Study or programme name or acronym:** Activating Communities to Improve Vitality and Equality (ACTIVE)  **Type of record:** Journal article |
| **Notes** | Linked study: Keall 2015 |

## Risk-of-bias table

| **Bias** | **Authors' judgement** | **Support for judgement** |
| --- | --- | --- |
| Random sequence generation (selection bias) | High risk | CBA study. |
| Allocation concealment (selection bias) | High risk | CBA study - control sites chosen by evaluators. |
| Baseline characteristics similar | Unclear risk | Table 3 (Chapman 2014) shows some small differences in age and personal income that could nevertheless potentially be meaningful. |
| Baseline outcome measurements similar | Unclear risk | Authors write that baseline levels of active travel are 'generally similar', looking at Table 1, overall, this appears to be the case, however there are some differences across age groups that could be meaningful. |
| Blinding of participants and personnel (performance bias) | Low risk | No blinding, however this was unlikely to influence the results apart from the effect of the intervention itself. |
| Blinding of outcome assessment (detection bias) | High risk | Use of self-reported outcomes could be biased due to knowledge of the intervention. |
| Protection against contamination | Low risk | Two different cities |
| Incomplete outcome data (attrition bias) | Unclear risk | Participation in general was low, and attrition quite high over time; it appears as though this may be somewhat balanced across groups, however, at this level of attrition there still may be bias, but it is not possible to judge this well. |
| Selective reporting (reporting bias) | Low risk | Some slight differences in outcomes outlined in the initial publication, however, overall it appears that the overall frame and parameters of the analysis plan remained the same. |
| Other bias | Low risk | No further sources of bias identified. |

# Cohen 2009

| **Methods** | **Study design:** CBA  **Study grouping:**  **How was missing data handled?:** Missing data excluded. "...analysis included only eight of the ten study parks. The first pair was eliminated from this analysis because a few key questions had not been included in the initial survey given to residents living near those parks."  **How were participants recruited and sampled within the study sites?:** Park users were observed during specific times: "Observations were conducted in all activity areas 7:30–8:30 AM, 12:30–1:30 PM, 3:30–4:30 PM, and 6:30–7:30 PM during each of the 7 days of the week." They also "...surveyed park users and recruited them systematically from the most- and least-busy areas, by gender, and by activity level (i.e., sedentary, physically active)." "In addition, residents living within a 2-mile radius of the park were surveyed. More specifically, households were classified into four strata (within ¼ mile, from ¼ to ½ mile, from ½ to 1 mile, and from 1 to 2 miles from each park) and sampled, approximately equal numbers of households were sampled from each stratum."  **Methods for selecting intervention and control sites:** "Five intervention parks had been scheduled for major improvements with budgets in excess of $1,000,000 after December 2003, and each intervention park was matched with a similar park (i.e., comparison park) that was not slated to receive upgrades by the city. The selected comparison park was similar in size, features, and amenities, and it served a population with similar sociodemographic characteristics as its intervention counterpart."  **Number of control sites:** 5 parks  **Number of intervention sites:** 5 parks  **Post-intervention period (start and end date or year it was conducted):** NR. It is assumed that construction started after Nov 2004 (end of baseline data collection)  **Pre-intervention time period (before intervention start):** Dec 2003-Nov 2004, period of data collection when renovations hadn't started. Construction dates NR  **Sample size justification and outcome used:** NR  **Study aim or objective:** "An opportunity for a natural experiment to improve local parks using community participation in the design served as the basis of the current study, which was aimed at determining the impact of the improvements on park use and the physical activity of park users."  **Time points of data collection:** Two: Baseline data were collected between December 2003 and November 2004; follow-up data were collected between April 2006 and March 2008" "...follow-up measures were initiated at least 3 months after construction; thus, the onset of observations varied from 3 months to 14 months post-construction"  **Unit of allocation or exposure:** Park |
| --- | --- |
| **Participants** | **Baseline Characteristics**  Intervention   - *Age*: NR - *Place of residence*: NR - *Sex*: NR - *Ethnicity and language*: NR - *Occupation*: NR - *Education*: NR - *Socioeconomic status*: NR - *Social capital*: NR   Control   - *Age*: NR - *Place of residence*: NR - *Sex*: NR - *Ethnicity and language*: NR - *Occupation*: NR - *Education*: NR - *Socioeconomic status*: NR - *Social capital*: NR   Overall   - *Age*: Of park users interviewed; median age in years: 36.5. Of park users interviewed; median age in years: 38.5 - *Place of residence*: Low-income neighborhoods in southern California - *Sex*: (Males %) Park users: 46.2%, Residents : 37.8% - *Ethnicity and language*: Of park users interviewed; Race/ethnicity (%): Latino 79.2; White: 3.3; Black 16.9; Asian 0.6. Of park users interviewed; Race/ethnicity (%): Latino 86.5; White: 0.7; Black 12.3; Asian 0.2 - *Occupation*: NR - *Education*: NR - *Socioeconomic status*: NR - *Social capital*: NR   **Included criteria:** NR. Parks had been selected for renovations. People visiting the parks were observed and interviewed. Residents within a 2-mile radius of the park were sampled and interviewed.  **Excluded criteria:** NR  **Pretreatment:** Differences according to intervention/control parks NR, only for each park.  **Attrition per relevant group:** Two cross-sectional samples of park users interviewed: 768 at baseline and 712 at follow-up. Same sample of residents followed up from baseline to follow-up; attrition = 147 residents.  **How were baseline differences assessed?:** "Propensity score weighting is an effective way of eliminating the differences in the observed characteristics (such as age, gender, and race) between survey respondents sampled at an intervention park at follow-up and respondents sampled at a control park at follow-up."  **Total number completed and analysed per relevant group:** 8/10 parks analysed: 4 intervention and 4 comparison parks. Two cross-sectional samples of park users interviewed: 768 at baseline and 712 at follow-up. Total number of residents analysed: 620  **Total number in each relevant group pre-intervention:** 10 parks: 5 intervention and 5 comparison parks. Two cross-sectional samples of park users interviewed: 768 at baseline and 712 at follow-up. Total number of park users observed at baseline: 768.  **Total number randomised per relevant group:** NA |
| **Interventions** | **Intervention Characteristics**  Intervention   - *Type of intervention (e.g. infrastructure, policy or both)*: Infrastructure - *Description of main intervention*: Infrastructure upgrade: "Three parks constructed completely new gymnasiums. Two of the three parks had old gymnasiums: One retained the old gym, so they ended up with two gyms, whereas the other razed and replaced the one they had. The fourth park had its old gymnasium refurbished and underwent some field improvements in watering and landscaping. The fifth had improvements to picnic areas,upgrades to a walking path, and enhancements to a play-ground area so that it had rubberized surfacing around the climbing apparatus and stationary horses." - *Description of additional intervention components*: "Three parks constructed completely new gymnasiums. Two of the three parks had old gymnasiums: One retained the old gym, so they ended with two gyms, whereas the other razed and replaced the one they had. The fourth park had its old gymnasium refurbished and underwent some field improvements in watering and landscaping. The fifth had improvements to picnic areas,upgrades to a walking path, and enhancements to a playground area so that it had rubberized surfacing around the climbing apparatus and stationary horses." - *Duration of intervention*: indefinite but "follow-up measures were initiated at least 3 months after construction; thus, the onset of observations varied from 3 months to 14 months post-construction." - *How was the intervention implemented?*: "Each intervention park scheduled open public meetings to discuss improvements and a voluntary oversight committee was formed with members appointed by local elected officials to ensure community participation. " However "..was limited to initial plans and did not continue once they were approved", "other changes that occurred concurrently with facility improvements. During the study period, the Department of Recreation and Parks suffered budget cuts that led to reduced programming. This resulted in reduced hours for several gymnasiums, and one gymnasium reduced hours as a consequence of gang intimidation. Another park shortened its baseball season, which earlier attracted both players and hundreds of spectators. Subsequently, with reduced hours of operation and fewer organized programs and events, lower attendance would be expected regardless of new park facilities." - *Co-interventions*: NR - *Where was the intervention implemented?*: Five parks in low-income neighborhoods in southern California - *Resource requirements to replicate the intervention*: NR - *Economic indicators*: "Five intervention parks had been scheduled for major improvements with budgets in excess of$1,000,000 after December 2003."   Control   - *Type of intervention (e.g. infrastructure, policy or both)*: No infrastructure - *Description of main intervention*: "... (i.e., comparison park) that was not slated to receive upgrades by the city" - *Description of additional intervention components*: No intervention - *Duration of intervention*: NA - *How was the intervention implemented?*: NA - *Co-interventions*: NR - *Where was the intervention implemented?*: Five parks in low-income neighborhoods in southern California - *Resource requirements to replicate the intervention*: NA - *Economic indicators*: NA |
| **Identification** | **Sponsorship source:** "This study was supported by NIEHS Grant P50ES012383-05"  **Country:** United States  **Setting:** Urban parks and surrounding communities in Southern California  **Comments:**  **Author’s name:** Deborah A. Cohen  **Institution:** RAND Corporation, Santa Monica  **Email:** dcohen@rand.org  **Address:**  **Declaration of interests stated?:** Yes. "No financial disclosures were reported by the authors of this paper."  **Specify any documents relevant to this study:** NR  **Study or programme name or acronym:** NA  **Type of record:** Journal article |
| **Notes** |  |

## Risk-of-bias table

| **Bias** | **Authors' judgement** | **Support for judgement** |
| --- | --- | --- |
| Random sequence generation (selection bias) | High risk | CBA study. |
| Allocation concealment (selection bias) | High risk | CBA study - control sites chosen by evaluators. |
| Baseline characteristics similar | Low risk | Propensity score weighting is an effective way of eliminating the differences in the observed characteristics (such as age, gender, and race) between survey respondents sampled at an intervention park at follow-up and respondents sampled at a control park at follow-up. |
| Baseline outcome measurements similar | Unclear risk | There seems to be some difference in outcomes between intervention and comparison parks but this was not formally assessed. |
| Blinding of participants and personnel (performance bias) | Low risk | No blinding done but unlikely to influence behavior of participants/personnel |
| Blinding of outcome assessment (detection bias) | High risk | No blinding done; assessors could potentially be influenced in their observation and interviews if they knew which park was renovated or not. |
| Protection against contamination | Low risk | “*Five intervention parks had been scheduled for major improvements with budgets in excess of $1,000,000 after December 2003, and each intervention park was matched with a similar park (i.e., comparison park) that was not slated to receive upgrades by the city.”*  Allocation was done by the city |
| Incomplete outcome data (attrition bias) | High risk | Two parks excluded from analysis (8/10 = 20%). For the longitudinal sample, 142/712 (19.9%) excluded. |
| Selective reporting (reporting bias) | Unclear risk | Protocol not available. |
| Other bias | High risk | Measurement bias: High risk. Long time span between baseline and follow-up (only two time points 2-4 years apart) meant other factors could have influenced the outcomes. |

# Cortinez O’Ryan 2017

| **Methods** | **Study design:** CBA  **Study grouping:**  **How was missing data handled?:** "Missing data for pedometer-derived PA were imputed in some children (baseline n = 18; final n = 36) who did not have enough valid days (i.e. forgot to use it in the morning or left it at home) assuming that missing data were missing at random. Missing values in participants were assumed to be similar to those participants with similar age, gender, and body mass index."  **How were participants recruited and sampled within the study sites?:** Not reported in detail - Figure 1 shows that 66 children from the intervention neighborhood and 76 children from the control neighborhood were invited to participate; "After identifying the intervention and control neighborhoods (with residences consisting of both houses and apartments) children/parents were invited to participate.  **Methods for selecting intervention and control sites:** Only two neighborhoods met inclusion criteria - it is unclear how it was determined to which of the two the intervention would be allocated. "Six neighborhoods (1191 households) were surveyed to assess neighborhood eligibility according to inclusion criteria. Although we initially planned to randomly select two neighborhoods (as Fig 1 shows) only two out of the six neighborhoods assessed for eligibility met inclusion criteria and therefore became our study groups." Neighborhood inclusion criteria: "Inclusion criteria for both the intervention and control group were: matching socio-economic and environmental characteristics such as proximity to and size of green space; presence of both apartments and houses, and level of crime...The presence of at least 80 children between the ages of four and 12, the absence of sport centre alternatives, and separation from each other of at least 1.5 km—to prevent intervention contamination—were also required."  **Number of control sites:** 1  **Number of intervention sites:** 1  **Post-intervention period (start and end date or year it was conducted):** September - December 2014 (12 weeks)  **Pre-intervention time period (before intervention start):** Baseline - two weeks before intervention begin  **Sample size justification and outcome used:** Sample size calculation not provided. Authors reported that "The small sample size of this study may also have limited the power to detect significant differences between groups..."  **Study aim or objective:** "The aim of this study was to investigate the feasibility and effect of a street play initiative on children’s outdoor play and PA."  **Time points of data collection:** Baseline: 2 weeks pre-intervention begin; Follow-up: last two weeks of the intervention period  **Unit of allocation or exposure:** Neighborhood |
| --- | --- |
| **Participants** | **Baseline Characteristics**  Intervention   - *Age*: Median (IQR): 9(5) - *Place of residence*: Apartment n(%): 20(39.2) - *Sex*: Female n(%): 24(47) - *Ethnicity and language*: - *Occupation*: - *Education*: - *Socioeconomic status*: Socio-economic position Low n(%): 39 (76) - *Social capital*:   Control   - *Age*: Median (IQR): 7(5) - *Place of residence*: Apartment n(%): 19(18) - *Sex*: Female n(%): 27(55) - *Ethnicity and language*: - *Occupation*: - *Education*: - *Socioeconomic status*: Socio-economic position Low n(%): 36(73) - *Social capital*:   Overall   - *Age*: - *Place of residence*: - *Sex*: - *Ethnicity and language*: - *Occupation*: - *Education*: - *Socioeconomic status*: - *Social capital*:   **Included criteria:** NR  **Excluded criteria:** NR  **Pretreatment:** See table 1: age was significantly different between groups (9 vs. 7). "No differences were observed between the intervention neighborhood and control neighborhood for any demographic characteristics except for age, in which participants from the control neighborhood were younger than intervention neighborhood participants."  **Attrition per relevant group:** Intervention neighborhood Pedometer outcomes: 21/51 = 41% attrition Questionnaire outcomes: 5/51 = 10% attrition Control neighborhood Pedometer outcomes: 15/49 = 31% attrition Questionnaire outcome: 2/49 = 4% attrition; however analyses were based on imputed data for participants with missing data.  **How were baseline differences assessed?:** "Mann Withney U test for continuous data and Chi-square test for categorical data."  **Total number completed and analysed per relevant group:** Intervention neighborhood Pedometer outcomes: 30 Questionnaire outcomes: 46 Control neighborhood Pedometer outcomes: 34 Questionnaire outcomes: 47. However, the analyses are based on 51 (intervention) and 49 (control) children, as imputed data were used.  **Total number in each relevant group pre-intervention:** Intervention neighborhood: 51 Control neighborhood: 49  **Total number randomised per relevant group:** NA |
| **Interventions** | **Intervention Characteristics**  Intervention   - *Type of intervention (e.g. infrastructure, policy or both)*: Infrastructure - *Description of main intervention*: JETB: Play in the neighborhood which entailed closing a street in a low-to middle income neighborhood in order to increase children’s outside play and physical activity. So four continuous blocks were closed to motorized traffic from 17:30 to 20:30 each Wednesday and Friday to create supportive physical and social environments for children and promote active free-play. - *Description of additional intervention components*: Additional components were designed for different levels: Individual, interpersonal, community, policy: Informational materials to create awareness of JETB fitness: Individual " informational leaflet and a magnetic calendar with programmed JETB sessions...". : Interpersonal: Local monitors led group games and incentivized children to meet each other during the first four sessions. Some neighbors took on this role while others provided music and organized contests in some sessions..: Community: The social and physical environment was modified through street closures organized by experienced stewards from CicloRecreoVia, who were located at each intersection. Wearing identifiable uniforms and using special traffic signs they rerouted traffic, kept the street free from parked cars, and alerted other adults if any problems arose. Their vigilance also increased the sense of perceived safety, also termed ‘eyes in the street’ [39]. They placed physical barriers at each end of the neighborhood’s main street and cones at every intersection. Policy: The temporary road closure had local authority permission, which was granted after they met with the research team, and reflects their support towards the intervention and its purpose. - *Duration of intervention*: "12 weeks twice per week (September-December 2014)...17:30 to 20:30 each Wednesday and Friday" - *How was the intervention implemented?*: "A meeting with neighbors and stakeholders was held in the intervention neighborhood to obtain input concerning feasibility, acceptability, and design of JETB. The project was well received and the strategies proposed by neighbors such as street cleaning (stones, pieces of glass, street dogs), posting advertising posters, and providing activation aids such as music, facilitators, and playing materials, were included in the intervention. The schedule for the intervention was decided by neighbor consensus...The websitewww.juegaentubarrio.cl, a logo, and a Facebook page were developed....The temporary road closure had local authority permission, which was granted after they met with the research team, and reflects their support towards the intervention and its purpose." - *Co-interventions*: NR - *Where was the intervention implemented?*: Along four continuous blocks in a low SES mixed land-use neighborhood - *Resource requirements to replicate the intervention*: Trainers and all essential resources e.g. uniforms, stewards and coordinators - *Economic indicators*: "The overall intervention cost (resources, uniforms, stewards and coordinator fees) for the 26 sessions was US$2275."   Control: No intervention |
| **Identification** | **Sponsorship source:** "This research was funded by the National Fund for research and development in health, FONIS, http://www.conicyt.cl/fonis/, Grant number: EVS-13I0052 (AC)."  **Country:** Chile  **Setting:** Underserved neighborhoods in Santiago, Chile  **Comments:**  **Author’s name:** Andrea Corinez-O'Ryan  **Institution:**  **Email:** andrea.cortinez@ufrontera.cl  **Address:**  **Declaration of interests stated?:** "The authors have declared that no competing interests exist."  **Specify any documents relevant to this study:** Supplementary files: S1 Table. Comparison of intervention and control neighborhoods’ environmental and social conditions.S1 File. Semi-structured interview guide.  **Study or programme name or acronym:** Juega en tu Barrio (Play in your Neighborhood)  **Type of record:** Journal article |
| **Notes** |  |

## Risk-of-bias table

| **Bias** | **Authors' judgement** | **Support for judgement** |
| --- | --- | --- |
| Random sequence generation (selection bias) | High risk | Quote: "eligibility according to inclusion criteria. <b>Although we initially planned to randomly select two neighborhoods (as Fig 1 shows) only two out of the six neighborhoods assessed for eligibility met inclusion criteria and therefore became our study groups.</b> Setting JETB was conducted in."  CBA study, likely that bias due to differences in sites present. |
| Allocation concealment (selection bias) | High risk | CBA study, unclear how the intervention and control allocation was determined between the two study sites. |
| Baseline characteristics similar | High risk | Quote: "PA decreases with age [61], increased PA levels in the control neighborhood could be attributed to age differences, as the average child in the control neighborhood was significantly younger than those included in the intervention neighborhood. The small sample size of"  As described in the discussion, the between-group difference in age may have biased the results on physical activity. |
| Baseline outcome measurements similar | Low risk | Judgement Comment: Across the many outcomes, only 'meeting pedometer-derived physical activity guidelines' was significantly different between the two groups at baseline. |
| Blinding of participants and personnel (performance bias) | Low risk | Lack of blinding of parental-reported outcomes could have led to bias in the study; potentially the pedometer-based outcomes could also be biased, but this seems less likely. |
| Blinding of outcome assessment (detection bias) | High risk | The lack of blinding of parent-reported outcomes (given that they were trained interviewer-assisted), could have led to bias; objective pedometer outcomes would not have been susceptible to bias. |
| Protection against contamination | Low risk | Intervention and control neighborhood were separated by 4.91 km, unlikely that contamination led to bias. |
| Incomplete outcome data (attrition bias) | Unclear risk | Quote: "per day for females [46]. <b>Missing data for pedometer-derived PA were imputed in some children (baseline n = 18; final n = 36) who did not have enough valid days (i.e. forgot to use it in the morning or left it at home) assuming that missing data were missing at random [47]. Missing values in participants were assumed to be similar to those participants with similar age, gender, and body mass index. No differences were observed between those participants that provided valid pedometer data and those who did not in regards to their demographic characteristics.</b> Participants received a small item."  Missing outcome data were imputed; given that no sensitivity analyses were conducted to explore the impact of data imputation, it is difficult to determine whether this may have introduced bias. |
| Selective reporting (reporting bias) | Low risk | The outcomes reported are consistent in both methods and results section. Selective outcome reporting is not apparent. |
| Other bias | Unclear risk | The accuracy of subjective parent-reported data is questionable. |

# D’Haese 2015

| **Methods** | **Study design:** CBA  **Study grouping:**  **How was missing data handled?:** NR  **How were participants recruited and sampled within the study sites?:** "A list with all play streets in Ghent during the summer vacation was obtained....In play streets and in adjacent streets that were directly connected to the Play Street, and in comparable control neighborhoods, door-to-door visits were conducted" to recruit eligible children.  **Methods for selecting intervention and control sites:** "A list with all Play Streets in Ghent (Flanders) during summer vacation (2 months: July and August) 2013 (n = 79) was obtained from the city council… Nineteen Play Street projects that lasted at least 7 consecutive days were selected... For each Play Street, a control neighborhood (i.e. statistical sector) with comparable walkability characteristics and annual household income (National Institute of Statistics – Belgium, 2008) in Ghent was selected. The distance between the Play Streets and the control neighborhoods was on average 4.9 ± 3.4 km."  **Number of control sites:** N = 19  **Number of intervention sites:** N = 19  **Post-intervention period (start and end date or year it was conducted):** after August 2013  **Pre-intervention time period (before intervention start):** prior to July 2013  **Sample size justification and outcome used:** NR  **Study aim or objective:** "The aim of this pilot study was to test the effectiveness of Play Streets to increase urban children’s MVPA and to decrease their sedentary time."  **Time points of data collection:** Group A : Questionnaire 1 before measurement under normal condition, then measurement under intervention condition and Questionnaire 2 after intervention. Group B: Questionnaire 1 before measurement under intervention condition then measurement under normal condition and questionnaire 2 after normal condition. ("Data was collected in Ghent during July and August 2013.")  **Unit of allocation or exposure:** Individuals |
| --- | --- |
| **Participants** | **Baseline Characteristics**  Intervention   - *Age*: Mean age was 8.7 ± 2.2 years - *Place of residence*: Ghent - *Sex*: Male (%): 59.3 - *Ethnicity and language*: NR - *Occupation*: School-going children - *Education*: Primary school level - *Socioeconomic status*: Low as measured by parent's education : 38.9% - *Social capital*: NR   Control   - *Age*: Mean age was 9.3 ± 2.0 years - *Place of residence*: Ghent - *Sex*: Male (%): 51.4 - *Ethnicity and language*: NR - *Occupation*: School-going children - *Education*: Primary school level - *Socioeconomic status*: Low as measured by parent's education: 36.1% - *Social capital*: NR   Overall   - *Age*: Mean age was 9.0 ± 2.1 years - *Place of residence*: Ghent - *Sex*: Male (%): 54.8 - *Ethnicity and language*: NR - *Occupation*: School-going children - *Education*: Primary school level - *Socioeconomic status*: Low as measured by parent's education: 37.3% - *Social capital*: NR   **Included criteria:** ..."2 inclusion criteria: 1) being in primary school or starting primary school after summer school vacation or finished primary school in June (age 6-12) and 2) residing at home during the one week measurement period (not going on holidays, not staying with friends or grandparents…)..."  **Excluded criteria:** NR  **Pretreatment:** - There were more boys in the intervention group (59.3%) compared to the control group (51.4%) although the total number of children in the control group was higher (72 compared to 54). - On average the children in the control group were older than those in the intervention group.  **Attrition per relevant group:** NR  **How were baseline differences assessed?:** "Children’s age and sex were derived from the questionnaire 1. Parents were asked to report their own and their partner’s level of education. Educational attainment was used as a proxy for family socio-economic status (=SES). Families were classified as high SES-families if at least one parent had a college or university education; otherwise they were classified as low SES families."  **Total number completed and analysed per relevant group:** N = 126 (n = 72 for control and n = 54 for Playstreet)  **Total number in each relevant group pre-intervention:** N = 167 (n=71 for Playstreet, n=96 for Control)  **Total number randomised per relevant group:** NR |
| **Interventions** | **Intervention Characteristics**  Intervention   - *Type of intervention (e.g. infrastructure, policy or both)*: Infrastructure (could be a policy intervention?) - *Description of main intervention*: "Play Streets offer children a safe play space to be active in their own neighborhood. By organizing Play Streets,the neighborhood environment (e.g. creating a car-free play place) and the social environment (e.g. increased social interaction between children playing in the street) are targeted... a street in Ghent is eligible to become a PlayStreet if the street complies with the following conditions: the street is a residential street, with a speed limit of maximum 50 km/h, there is no significant passing traffic (e.g. public transport, no firehouse in the street), and the surrounding streets remain accessible after the introduction of the Play Street." - *Description of additional intervention components*: NR - *Duration of intervention*: "In Ghent, a street can become a Play Street for maximally 14 days in July and August (e.g. every Sunday, every weekend in July, one week in July and 1 week in August, 14 consecutive days)." - *How was the intervention implemented?*: "If the street meets all these requirements, every street inhabitant can fill out an application for a Play Street at the city council... When the city council approves the application,the majority of the households in the street have to agree with the approved application. Besides, at least 3 volunteers living in the street have to sign an agreement with the city council to hold responsibility for the organization of the Play Streets. They are the contact persons between the city council and the other street inhabitants. The task of the volunteers is to inform the street inhabitants about the rules and timing of the Play Streets. Each day the PlayStreet is organized, the volunteers enclose the Play Street with fences and a traffic sign, indicating that car traffic is forbidden in the streets. The fences and traffic signs are delivered by the city council. When the Play Street is not used on a specific day (e.g. due to heavy rain), the Play Street can be cancelled by the volunteers on that day." - *Co-interventions*: NR - *Where was the intervention implemented?*: Playstreets in Ghent, Belgium - *Resource requirements to replicate the intervention*: Volunteers, Play Materials, Adequate Setting (streets in residential areas with low speed traffic). - *Economic indicators*: NR   Control: No intervention |
| **Identification** | **Sponsorship source:** "SDH and DVD were supported by Research Foundation Flanders (FWO)."  **Country:** Belgium  **Setting:** Cities and villages in Ghent  **Comments:**  **Author’s name:** Greet Cardon  **Institution:**  **Email:** greet.cardon@ugent.be  **Address:**  **Declaration of interests stated?:** "The authors declare that they have no competing interests."  **Specify any documents relevant to this study:** N/A  **Study or programme name or acronym:** N/A  **Type of record:** Journal article |
| **Notes** |  |

## Risk-of-bias table

| **Bias** | **Authors' judgement** | **Support for judgement** |
| --- | --- | --- |
| Random sequence generation (selection bias) | High risk | CBA |
| Allocation concealment (selection bias) | High risk | CBA |
| Baseline characteristics similar | Low risk | Imbalance in sex and age adjusted in the analysis. |
| Baseline outcome measurements similar | Unclear risk | It is unclear whether outcomes were assessed for the 2 groups at baseline. |
| Blinding of participants and personnel (performance bias) | Low risk | Blinding was not done; unlikely that it would lead to bias. |
| Blinding of outcome assessment (detection bias) | Unclear risk | The authors do not report on whether outcome assessors were blinded. |
| Protection against contamination | Low risk | At least 4.9 ± 3.4 km separation between control and intervention groups. |
| Incomplete outcome data (attrition bias) | Low risk | Authors mentioned how they dealt with missing/insufficient data (they defined cutoff points). |
| Selective reporting (reporting bias) | Low risk | All relevant outcomes in the methods section are reported in the results section. |
| Other bias | Low risk | No misclassification or measurement bias. |

# Dill 2014

| **Methods** | **Study design:** CBA study  **Study grouping:** Parallel group  **How was missing data handled?:** All models in the analysis were limited to participants with at least three valid days of activity data and complete surveys in both the pre and post phases.  **How were participants recruited and sampled within the study sites?:** "Households within 1000 ft of the selected streets were recruited to participate through a flyer left at the front door of every accessible housing unit and mailed invitations for inaccessible units (n= 54,381)." Then participants were screened for eligibility.  **Methods for selecting intervention and control sites:** "The study areas include 8 street segments scheduled by the city for bicycle boulevard installation (0.9 to 4.2 miles long) and 11 control street segments (1.0 to 5.7 miles long). The control streets were selected to be similar in urban form and demographic characteristics, particularly with respect to access to bicycle infrastructure, and were often parallel streets several blocks away." The reason why the city selected those particular street segments is not reported.  **Number of control sites:** 11 street segments (1.0 to 5.7 miles long)  **Number of intervention sites:** 8 street segments (0.9 to 4.2 miles long)  **Post-intervention period (start and end date or year it was conducted):** "The amount of time between installation and post data collection varied between two and twelve months." Data collection in August 1, 2012 – November 4, 2012 for group 1 and April 27, 2013 – August 6, 2013 for group 2 (different groups depending on dates of construction/completion).  **Pre-intervention time period (before intervention start):** July 17, 2010 to November 8, 2010 for group 1 and April 27, 2011 to September 4, 2011 for group 2.  **Sample size justification and outcome used:** NR. All residents in study sites were invited to participate.  **Study aim or objective:** "The aim of this study is to evaluate changes in physical activity and active transportation associated with installation of new bicycle boulevards using a longitudinal, panel design with a control group."  **Time points of data collection:** Group 1 - pre-installation -July 17, 2010, to November 8, 2010, and post-installation - August 1, 2012 – November 4, 2012 Group 2 - pre-installation April 27, 2011 to September 4, 2011, and post-installation - April 27, 2013 – August 6, 2013.  **Unit of allocation or exposure:** Street segments |
| --- | --- |
| **Participants** | **Baseline Characteristics**  Intervention - street segments scheduled by the city for bicycle boulevard installation.   - *Age*: Mean age (at start of study): 43.1 - *Place of residence*: NR - *Sex*: % female: 61 - *Ethnicity and language*: NR - *Occupation*: NR - *Education*: % with 4-year college degree: 63 - *Socioeconomic status*: % Employed full-time: 56 - *Social capital*: % married: 60   Control - streets similar in urban form and demographic characteristics to intervention streets with respect to access to bicycle infrastructure, and often parallel streets several blocks away.   - *Age*: Mean age (at start of study): 40.8 - *Place of residence*: NR - *Sex*: % female: 64 - *Ethnicity and language*: NR - *Occupation*: NR - *Education*: % with 4-year college degree: 55 - *Socioeconomic status*: % Employed full-time: 48 - *Social capital*: % married: 56   Overall: NR  **Included criteria:** "Potential participants were screened for eligibility. At least one child aged 5 to 17 and one adult parent or guardian had to agree to participate for the length of the study; both had to be physically able to ride a bicycle, have access to a working bicycle, and not be intending to move in the near future."  **Excluded criteria:** NR  **Pretreatment:** "For participants with both pre and post data, the adults in the treatment group were slightly more likely to be employed full-time, be married, and have a four-year college degree." "The treatment households have better access to sidewalks and are somewhat closer to downtown."  **Attrition per relevant group:** Missing participants across activity data; GPS data; survey data (participants without valid pre- and post data): Treatment 23% - 28%; Control; 32% - 35%.  **How were baseline differences assessed?:** NR. proportions of demographic characteristics and objective environment measures reported for each group. For latter p-values are reported.  **Total number completed and analysed per relevant group:** Depends on outcome. For activity data: T 154, C 139 adults. For GPS data: T 177, C 164 adults. For survey data: T 183, C 170 adults.  **Total number in each relevant group pre-intervention:** Varies according to data. For activity data: T 215 , C 214 adults. For GPS data: T 231 , C 240 adults. For survey data: T 237 , C 253 adults.  **Total number randomised per relevant group:** n/a |
| **Interventions** | **Intervention Characteristics**  Intervention -street segments scheduled by the city for bicycle boulevard installation   - *Type of intervention (e.g. infrastructure, policy or both)*: Infrastructure - *Description of main intervention*: Installation of "bicycle boulevards. Bicycle boulevards are low-volume streets, often residential, that use traffic calming, diversion, signage, and intersection treatments to reduce the speed and volume of motor vehicles and create a better environment for people on bicycles." - *Description of additional intervention components*: NR - *Duration of intervention*: Once-off installation. "The amount of time between installation and post data collection varied between two and twelve months." - *How was the intervention implemented?*: "Installation of the boulevards often took more than a year, with speed humps and pavement markings going in early and crossing improvements taking longer to install. The amount of time between installation and post data collection varied between two and twelve months. Moreover, some elements of two of the nine projects were not completed within the study timeframe" "the design of each bicycle boulevard treatment differed, particularly regarding crossing treatments.Some projects included more substantial investments, such as a pocket park, flashing beacons, curb extensions, and extensive landscaping.Others consisted primarily of more subtle changes, including speed humps, sharrow markings, changed stop signs, and signage." - *Co-interventions*: None reported - *Where was the intervention implemented?*: Urban areas in Portland, Oregon - *Resource requirements to replicate the intervention*: NR - *Economic indicators*: NR   Control -streets similar in urban form and demographic characteristics to intervention streets with respect to access to bicycle infrastructure, and often parallel streets several blocks away   - *Type of intervention (e.g. infrastructure, policy or both)*: No intervention - *Description of main intervention*: No intervention - *Description of additional intervention components*: n/a - *Duration of intervention*: n/a - *How was the intervention implemented?*: n/a - *Co-interventions*: None reported - *Where was the intervention implemented?*: Urban areas in Portland, Oregon - *Resource requirements to replicate the intervention*: NR - *Economic indicators*: NR |
| **Identification** | **Sponsorship source:** "This research was funded by the Robert Wood Johnson Foundation Active Living Research program (#67127) and the Oregon Transportation Research and Education Consortium (OTREC) (Grant 446), a university transportation center funded by the U.S. Department of Transportation."  **Country:** USA  **Setting:** Street segments in urban areas in Portland, Oregon  **Comments:**  **Author’s name:** Jennifer Dill  **Institution:**  **Email:** jdill@pdx.edu  **Address:**  **Declaration of interests stated?:** Yes. "The authors do not have conflicts of interest to declare."  **Specify any documents relevant to this study:**  **Study or programme name or acronym:** Data analysed from the Family Active Study (FAS)  **Type of record:** Journal article |
| **Notes** |  |

## Risk-of-bias table

| **Bias** | **Authors' judgement** | **Support for judgement** |
| --- | --- | --- |
| Random sequence generation (selection bias) | High risk | CBA study |
| Allocation concealment (selection bias) | High risk | CBA study |
| Baseline characteristics similar | Unclear risk | "For participants with both pre and post data, the adults in the treatment group were slightly more likely to be employed full-time, be married, and have a four-year college degree." Authors report adjusting for demographic/ geographic/ attitudinal covariates but do not specify which. |
| Baseline outcome measurements similar | High risk | More participants in intervention group biked or walked and were more in favour of walking or biking compared to the control group. |
| Blinding of participants and personnel (performance bias) | Low risk | Authors state that "Participants were not told that the study was related to installation of bicycle boulevards or any other infrastructure." However, they do not state what they told them it was about. |
| Blinding of outcome assessment (detection bias) | Low risk | Data was collected through surveys, accelerometers and person-based GPS. It is unlikely that detection bias was introduced. |
| Protection against contamination | Low risk | Different street segments allocated to each intervention group. Changes to control street segments not reported. |
| Incomplete outcome data (attrition bias) | High risk | Authors state that "Retention in the study was higher among the treatment group." Attrition ranged from 23-28% in treatment group and 33-35% in control group (depending on type of outcome/data)." The city may have chosen to install bicycle boulevards in locations where residents were supportive of new bicycle infrastructure. This could correlate with stronger interest in the study, though study participants were not told that the study purpose was to evaluate the effect of the new facilities." |
| Selective reporting (reporting bias) | Unclear risk | Protocol not available |
| Other bias | Unclear risk | Misclassification bias of exposure: low risk. Measurement bias: Unclear risk; "The data collection methods (GPS and accelerometers) may change behavior, particularly during the pre-installation phase when the novelty of the devices and study is fresh; since our treatment participants had slightly more positive attitudes towards active transportation, this bias could result indifferences between the treatment and control. Finally, travel modes (bicycling and walking) were imputed using a regression model, which introduces error." |

# Fitzhugh 2010

| **Methods** | **Study design:** CBA  **Study grouping:**  **How was missing data handled?:** NR  **How were participants recruited and sampled within the study sites?:** Data was based on direct observation of participants in their normal daily activities. "Neighborhood-level direct observation, based on the pedestrian count survey methodology,11 was conducted for 2 hours each in the morning (7:00–9:00AM); midday (11:00AM–1:00PM); and evening (4:00–6:00PM) on 2 days (Wednesday and Saturday)". "School-level direct observation, which followed the protocol developed by Suminski and colleagues,12 was conducted for 2 days (Tuesday and Thursday) during the hours of 7:00–9:00AM and 2:30–4:00PM in order to best capture active transport to school (ATS)"  **Methods for selecting intervention and control sites:** 1. The Knoxville–Knox County Metropolitan Planning Commission first identified the intervention neighborhood in 1995 as a “special planning opportunity area” for retrofitting existing physical assets of the built environment to achieve a “true urban village form.” 2. Investigators identified five candidate neighborhoods that matched the intervention neighborhood along socioeconomic dimensions, and two control neighborhoods were selected.  **Number of control sites:** Two neighborhoods. Schools: two elementary and one high school  **Number of intervention sites:** One neighborhood. Schools: two elementary and one middle school  **Post-intervention period (start and end date or year it was conducted):** Dec 2005 to March 2007 "The construction of the greenway/trail ended in December 2005, allowing a 14-month exposure before the post-intervention assessment."  **Pre-intervention time period (before intervention start):** March 2005 to Dec 2005 "Pre-intervention assessment of directly observed physical activity occurred in March 2005..." and "The construction of the greenway/trail ended in December 2005..."  **Sample size justification and outcome used:** NR  **Study aim or objective:** "Direct observation of physical activity was used to examine whether improvements in the built environment causes increases in physical activity, in both neighborhood and school settings, in the intervention and control neighborhoods."  **Time points of data collection:** Pre-intervention assessment: 4th week of March 2005 and post-intervention assessment: 4th week of March 2007.  **Unit of allocation or exposure:** Neighborhoods |
| --- | --- |
| **Participants** | **Baseline Characteristics**  Intervention   - *Age*: median age (years): 30.0. percentage of elderly (65 years and over): 10.9. - *Place of residence*: Urban neighborhood in Knoxville - *Sex*: percentage female: 50.2 - *Ethnicity and language*: percentage black: 6.9. percentage minority ethnicity: 17.7. - *Occupation*: percentage unemployed: 5.6 - *Education*: proportion with less than high school education: 9.3 - *Socioeconomic status*: Median year house built: 1952. Median household income: $36563. percentage living in poverty: 32.2. - *Social capital*: NR   Control   - *Age*: median age (years): 39.5 - *Place of residence*: Knoxville-Knox county, Tennessee - *Sex*: percentage female: 53 - *Ethnicity and language*: percentage black: 5.4 - *Occupation*: percentage unemployed: 4.4 - *Education*: proportion with less than high school education: 9.7 - *Socioeconomic status*: Median year house built: 1967. Median household income: $50612 - *Social capital*: NR   Overall   - *Age*: NR - *Place of residence*: NR - *Sex*: NR - *Ethnicity and language*: NR - *Occupation*: NR - *Education*: NR - *Socioeconomic status*: NR - *Social capital*: NR   **Included criteria:** NR  **Excluded criteria:** NR  **Pretreatment:** NR. "Although the methods used in the current study included appropriate techniques for identifying control neighborhoods, the possibility remains that the experimental and control areas differ along unobservable dimensions."  **Attrition per relevant group:** N/A  **How were baseline differences assessed?:** NR  **Total number completed and analysed per relevant group:** NR  **Total number in each relevant group pre-intervention:** NR. in intervention neighborhood the population density=2590 people per square mile; 2207 occupied housing units Control neighborhoods: NR  **Total number randomised per relevant group:** N/A |
| **Interventions** | **Intervention Characteristics**  **Intervention**   - *Type of intervention (e.g. infrastructure, policy or both)*: Infrastructure - *Description of main intervention*: "The specifıc intervention involved retrofıtting a neighborhood with an urban greenway/trail to connect the pedestrian infrastructure with nearby retail establishments and schools." - *Description of additional intervention components*: None reported - *Duration of intervention*: Dec 2005-present - *How was the intervention implemented?*: Greenway/trail built. No other details reported. - *Co-interventions*: None reported. "In order to maintain the integrity of the current research design, the study neighborhoods were not exposed to any social marketing or awareness campaigns during the course of the study." - *Where was the intervention implemented?*: Urban neighborhood of Knoxville - *Resource requirements to replicate the intervention*: NR - *Economic indicators*: "Construction of the 8-foot-wide and 2.9-mile-long asphalt greenway cost $2.1 million"   **Control**: No intervention |
| **Identification** | **Sponsorship source:** "This study was supported by grants from the Office of Research and the Southeastern Transportation Center at the University of Tennessee, Knoxville."  **Country:** USA  **Setting:** Urban neighbourhoods in Knoxville, Tennessee  **Comments:**  **Author’s name:** Eugene C. Fitzhugh  **Institution:**  **Email:** fıtzhugh@utk.edu  **Address:**  **Declaration of interests stated?:** Yes. "No financial disclosures were reported by the authors of this paper."  **Specify any documents relevant to this study:** n/a  **Study or programme name or acronym:** n/a  **Type of record:** Journal article |
| **Notes** |  |

## Risk-of-bias table

| **Bias** | **Authors' judgement** | **Support for judgement** |
| --- | --- | --- |
| Random sequence generation (selection bias) | High risk | This was a CBA therefore no randomisation was done. |
| Allocation concealment (selection bias) | High risk | This was a CBA, therefore there was no randomisation. |
| Baseline characteristics similar | Unclear risk | The intervention and control neighborhoods appear to differ in key characteristics (e.g. high school education, household income, median age, percent unemployed), which could influence the levels of PA observed in these neighborhoods. |
| Baseline outcome measurements similar | Low risk | "At baseline (2005), there was no signifıcant relationship between the 2-hour total physical activity counts in the experimental and control neighborhoods.” (p0.370) |
| Blinding of participants and personnel (performance bias) | Low risk | It was not possible to blind users of the greenway or the research staff but this is unlikely to have influenced their behavior or outcomes recorded. |
| Blinding of outcome assessment (detection bias) | Low risk | It was not possible to blind users of the greenway or the research staff but this is unlikely to have influenced their behavior or outcomes recorded. |
| Protection against contamination | Unclear risk | It is unclear which were the control neighborhoods and how far they were from the intervention neighborhood. This would have influenced the possibility of contamination. "It is possible, however, that some of the greenway/trail users in the present study, especially those observed cycling, originated from outside the neighborhood, perhaps from neighborhoods close to the experimental neighborhood.”[10] |
| Incomplete outcome data (attrition bias) | Unclear risk | It is unclear whether there was missing data or if any data was excluded from analysis. |
| Selective reporting (reporting bias) | Low risk | Authors reported on outcomes for physical activity (pedestrians, cyclists, and individuals performing other forms of physical activity) at neighborhood level and active transport at school-level specified in the methods section. |
| Other bias | Unclear risk | Misclassification bias: unclear risk. Pedestrians may not be from the intervention neighborhood, and this would not have been picked up by research staff. Measurement bias: unclear risk. Measuring PA through direct observation of physical activity in a 1-week period at baseline and 14 months may be insufficient to validly assess PA. Incorrect analysis - n/a. |

# Frank 2019

| **Methods** | **Study design:** CBA study  **Study grouping:** Parallel  **How was missing data handled?:** Participants missing the outcome measures (n= 40 for MVPA; n= 38 for sedentary behavior) were not included in the models.  **How were participants recruited and sampled within the study sites?:** "In brief, residents living approximately within 1 km of the greenway were included in the study population. The study area is located in Vancouver's West End, a high-density residential and commercial neighborhood. A random sample of household addresses were identiﬁed by Mustel Group, a third-party market research company, using the Canada Post address data ﬁle as the sampling frame. Invitation letters were sent out by mail from the City of Vancouver to potential participants. Residents who provided informed consent were recruited into the study if they had no plans to move outside the study area during the time of the study. No reference to the proposed greenway was provided to minimize participation bias."  **Methods for selecting intervention and control sites:** Various distance thresholds to define likely 'exposure' to greenway: 100, 200, 300, 400 and 500 meters. 300m was considered the main threshold:" To represent the base experimental condition, a threshold of 300m was selected based on two criteria. First, 300m is equal to two-and-a-half street blocks using the existing street grid before reaching a major commercial street. Beyond this distance, bicyclists have the option of choosing a more attractive existing oﬀ-road shared pedestrian and bicyclist pathway along the waterfront. Second, 300m provides a roughly equal sample size for the experimental and control groups."  **Number of control sites:** 1  **Number of intervention sites:** 1  **Post-intervention period (start and end date or year it was conducted):** October 2014–March 2015  **Pre-intervention time period (before intervention start):** October 2012–March 2013  **Sample size justification and outcome used:** " A sample size calculation was conducted showing that given a neighborhood population of 44,543 (City of Vancouver, n.d.), 95% conﬁdence level, and a 5% margin of error, a minimum of 381 subjects needed to be analyzed."  **Study aim or objective:** The first of the following two objectives is relevant for this review." This study has two objectives. First, it assesses the eﬀect of the greenway retroﬁt on moderate-to-vigorous physical activity (MVPA) and sedentary behavior for residents living close to and further away from the greenway. Second, it assesses the eﬀect of residential proximity (distance away from the greenway) on the MVPA and sedentary behavior outcomes."  **Time points of data collection:** Baseline: October 2012–March 2013;Follow up: October 2014–March 2015  **Unit of allocation or exposure:** Geographical area |
| --- | --- |
| **Participants** | **Baseline Characteristics**  Intervention   - *Age*: mean (95% CI) 46.2 (44.3,48.1) - *Place of residence*: NR - *Sex*: % Female (95%CI): 0.55 (0.49,0.62) - *Ethnicity and language*: % White (95% CI):0.86(0.82,0.91) - *Occupation*: % employed (95% CI): 0.73 (0.67, 0.78) - *Education*: % with post secondary education (95% CI): 0.72 (0.67, 0.78) - *Socioeconomic status*: NR - *Social capital*: NR   Control   - *Age*: mean (95% CI) 44.7 (43.0, 46.4) - *Place of residence*: NR - *Sex*: % Female (95%CI): 0.59 (0.53, 0.65) - *Ethnicity and language*: % White (95% CI): 0.77 (0.72, 0.82) - *Occupation*: % employed (95% CI): 0.77 (0.72, 0.82) - *Education*: % with post secondary education (95% CI): 0.78 (0.73, 0.83) - *Socioeconomic status*: NR - *Social capital*: NR   Overall   - *Age*: NR - *Place of residence*: NR - *Sex*: NR - *Ethnicity and language*: NR - *Occupation*: NR - *Education*: NR - *Socioeconomic status*: NR - *Social capital*: NR   **Included criteria:** "Residents who provided informed consent were recruited into the study if they had no plans to move outside the study area during the time of the study."  **Excluded criteria:** "Participants were excluded from the study if they did not participate in the follow-up period (n = 556), did not complete the survey for the two survey days (n = 25), or were otherwise ineligible (n = 8)."  **Pretreatment:** "There was a signiﬁcant diﬀerence in ethnicity between the experimental (86.2% white) and the control group (76.8% white, t = −2.7)."  **Attrition per relevant group:** Total study: 47.1% NR per group  **How were baseline differences assessed?:** "Baseline diﬀerences in demographic covariates were compared across the experimental and control groups using two-tailed t-tests."  **Total number completed and analysed per relevant group:** Intervention: 239 Control: 285  **Total number in each relevant group pre-intervention:** At the baseline, a total of 1113 participants were recruited  **Total number randomised per relevant group:** NA |
| **Interventions** | **Intervention Characteristics**  Intervention   - *Type of intervention (e.g. infrastructure, policy or both)*: Infrastructure - *Description of main intervention*: "The Comox Greenway is a major active transportation corridor extending east-to-west through downtown Vancouver, with a particular aim of improving conditions for bicyclists of all ages and abilities (Fig. 1). The two-kilometer route consists of a mix of cycling facilities and other streetscape improvements: one-way shared on-street with counterﬂow lanes (22%); one-way protected (29%); and two-way shared on-street (49%)." - *Description of additional intervention components*: None - *Duration of intervention*: Permanent after implementation (June 2013) - *How was the intervention implemented?*: NR - *Co-interventions*: NR - *Where was the intervention implemented?*: "The study area is located in Vancouver's West End, a high-density residential and commercial neighborhood." - *Resource requirements to replicate the intervention*: NR - *Economic indicators*: NR   Control   - *Type of intervention (e.g. infrastructure, policy or both)*: No intervention - *Description of main intervention*: No intervention - *Description of additional intervention components*: No intervention - *Duration of intervention*: No intervention - *How was the intervention implemented?*: No intervention - *Co-interventions*: No intervention - *Where was the intervention implemented?*: No intervention - *Resource requirements to replicate the intervention*: No intervention - *Economic indicators*: No intervention |
| **Identification** | **Sponsorship source:** "It was funded by the City of Vancouver (Contract #: PS20120467) with further support from the UBC Health & Community Design Lab through the Bombardier Foundation. The City of Vancouver reviewed and approved the study design of the larger study, and provided input into the selection of incentives for participant recruitment, particularly with respect to the use of gift certiﬁcates and prize draw for civic facilities. They were not involved in the collection, analysis, and interpretation of the data in the larger or the present study, or the decision to submit this paper for publication."  **Country:** Canada  **Setting:** The study area is located in Vancouver's West End, a high-density residential and commercial neighborhood.  **Comments:**  **Author’s name:** Andy Hong (contact author)  **Institution:** University of British Columbia, School of Population and Public Health, School of Community and Regional Planning  **Email:** andyhong@gmail.com  **Address:**  **Declaration of interests stated?:** NR  **Specify any documents relevant to this study:** Supplementary data to this article can be found online at https://doi.org/10.1016/j.ypmed.2019.01.011.  **Study or programme name or acronym:** Travel, Health, Safety and Social impact of the Comox-Helmcken Greenway Corridor  **Type of record:** Journal article |
| **Notes** |  |

## Risk-of-bias table

| **Bias** | **Authors' judgement** | **Support for judgement** |
| --- | --- | --- |
| Random sequence generation (selection bias) | High risk | CBA study - exposure threshold selected by researchers. |
| Allocation concealment (selection bias) | High risk | CBA study - exposure threshold selected by researchers. |
| Baseline characteristics similar | Low risk | Differences were present, however, these are unlikely to have introduced substantial bias due to the inclusion of covariates into DiD models. |
| Baseline outcome measurements similar | Low risk | Differences were present, however, these are unlikely to have introduced substantial bias due to the DiD analysis. |
| Blinding of participants and personnel (performance bias) | Low risk | Both main outcomes, MVPA and physical activity were subjective outcomes reported through use of a survey question. It is possible that this led to biased reporting of effects differentially between groups. |
| Blinding of outcome assessment (detection bias) | High risk | No blinding and subjective outcome assessed. |
| Protection against contamination | Unclear risk | By nature of the study design, contamination was likely an issue here; however, it would have biased the effect estimate towards 0. Additionally, the addition of additional analyses varying the distance threshold allowed authors to explore this. |
| Incomplete outcome data (attrition bias) | Unclear risk | Attrition was extremely high; additionally, for some who completed the study, outcome data were missing. Authors claim that missing individuals did not differ with regard to demographic characteristics from those at baseline. "A participant drop-out analysis was conducted and no statistically signiﬁcant diﬀerences were found across socio-demographic characteristics from the baseline sample to the ﬁnal follow-up sample." |
| Selective reporting (reporting bias) | Low risk | No indication of selective outcome reporting. |
| Other bias | Low risk | No other risks of bias identified. |

# Goldsby 2016

| **Methods** | **Study design:** CBA  **Study grouping:**  **How was missing data handled?:** Missing data was excluded from the study. "Thus, nine children who were not seen in both the pre-park and post-park time periods were excluded from this study."  **How were participants recruited and sampled within the study sites?:** "convenience sample of 2151 children under the age of 19 at baseline who were seen in a Jefferson County Department of Health (JCDH) clinic for primary care visits during the study period...". The authors used electronic health records (EHR) data to recruit participants.  **Methods for selecting intervention and control sites:** Railroad Park is a 19-acre green space that contains numerous areas for PA for both children and adults - it was chosen as intervention site: "We consider our exposure group as those children living in closest proximity (i.e., walkable distance) to the newly-established park" and "Our control group is identified as similar children who live farther away from the park and, thus, presumably cannot walk to the park."  **Number of control sites:** 1 based on distance from park (>5 miles)  **Number of intervention sites:** 3 exposure groups based on nearness to park: within 1.5 miles, 1.5-3 miles, 3-5 miles).  **Post-intervention period (start and end date or year it was conducted):** 1 March 2011 through 28 February 2012 (time during which data was collected). Park completion Sep 2010.  **Pre-intervention time period (before intervention start):** 1 February 2009 through 1 September 2010  **Sample size justification and outcome used:** NR  **Study aim or objective:** "The purpose of this study was to determine how close to the park is close enough to experience health benefits. We also examine changes in Body Mass Index (BMI) z-scores at various distances from the park." "The current study examines how exposure to Railroad Park as measured by the Euclidian distance from one’s home to the park relates to changes in BMI z-scores over time among children."  **Time points of data collection:** Two. Pre-park time frame: 1 February 2009 through 1 September 2010. Post-park timeframe: 1 March 2011 through 28 February 2012.  **Unit of allocation or exposure:** individual |
| --- | --- |
| **Participants** | **Baseline Characteristics**  Railroad Park   - *Age*: mean age (range) - near: 9 (2, 16.9); intermediate 1: 10.5 (2, 17.7); intermediate 2: 10.8 (2, 17.6). - *Race*: race, n(%) - near: black 35(78); white: 10(22), other 0(0). Intermediate 1.5-3 miles: black 140(85), white 21(13), other 3(2). Intermediate 3-5 miles: black 270(90), white 27(9), other 2(1). - *Sex*: n(%) female. near: 26(25), intermediate 1.5-3 miles: 91(55), intermediate 3-5 miles: 160(54). - *Ethnicity and language*: - *Overweight or Obese*: overweight (85%-95%), n(%) - near: 7(16), intermediate 1.5-3 miles: 34(21), intermediate 3-5 miles: 53(18). Obese, n(%) - near: 8(18), intermediate 1.5-3 miles: 44(27), intermediate 3-5 miles: 81(27). - *Payer Type*: DELETE - *Socioeconomic status*: Payer type, n(%) - Self-pay: near: 23 (51), intermediate 1.5-3 miles: 69 (42), intermediate 3-5 miles: 100 (33). Other, n(%) - near: 22 (49), intermediate 1.5-3 miles: 95 (58), intermediate 3-5 miles: 199 (67). - *Social capital*: DELETE   Control   - *Age*: Median (range): 10.3 (2, 17.9) - *Race*: Black 672 (72%) White 257 (27%) Other 6 (1%) - *Sex*: Female n=532 (57%); Male n=403 (43%) - *Ethnicity and language*: Hispanics 137 (15%) Non-hispanics 798 (85%) - *Overweight or Obese*: Overweight (85%–95%), n(%): 182 (19%); Obese, n(%): 232 (25%) - *Payer Type*: DELETE - *Socioeconomic status*: Payer type, n(%) - Self-pay, n(%): 378 (40). Other, n(%): 557 (60) - *Social capital*: NR   Overall   - *Age*: Median Age (range): 10.3 (2, 17.9) - *Race*: Black 1117 (77%) White 315 (22%) Other 11 (1%) - *Sex*: Gender, n(%) - Female: 809 (56); Male: 634 (44%) - *Ethnicity and language*: n(%): Hispanic: 196 (14); Non-Hispanic: 1247 (86) - *Overweight or Obese*: n(%) - Overweight (85%–95%): 276 (19); Obese: 365 (25) - *Payer Type*: DELETE - *Socioeconomic status*: Payer type, n(%) - Self-pay: 570 (40); Other: 873 (60) - *Social capital*: NR   **Included criteria:** "...children under the age of 19 at baseline who were seen in a Jefferson County Department of Health (JCDH) clinic for primary care visits during the study period." "To be included in this study, individuals need to be seen at one of the six clinics both pre- and post-park in order to examine the change in BMI z-scores over time."  **Excluded criteria:** If BMI measurements appear implausible: "to account for implausibly large changes in BMI (i.e., data recording errors) 671 children whose BMI z-score changed by more than 0.67 between consecutive visits, or by more than 0.33 for visits less than 90 days apart, were excluded from the study. "AND" if a family relocated during the study period, the child’s data were excluded from the study."  **Pretreatment:** Group differences were assessed at baseline (Table 1); significance test for group differences is not provided. There seem to be some significant differences for some of the variables e.g. % obese.  **Attrition per relevant group:** N/A. Dataset obtained from EHR data.  **How were baseline differences assessed?:** Descriptively by comparing means and proportions (Table 1). No statistical test performed.  **Total number completed and analysed per relevant group:** Control Group: 935 children (living five miles or further from the park). Intervention: 508 children (45 children living in near group - within 1.5 miles from the park; 164 children living 1.5 to 3 miles from the park; 299 children living 3 to 5 miles from the park).  **Total number in each relevant group pre-intervention:** "A total of 1443 children were included in this study." Intervention: 508 children. Control: 935 children  **Total number randomised per relevant group:** n/a |
| **Interventions** | **Intervention Characteristics**  Railroad Park construction   - *Type of intervention (e.g. infrastructure, policy or both)*: Infrastructure - *Description of main intervention*: Construction of a park; "Railroad Park is a 19-acre green space that contains numerous areas for PA for both children and adults. The park includes two age-appropriate play areas with modern playground equipment (e.g., climbing dome, slides, ropes, obstacle courses, and a skateboard park) appropriate for young children and adolescents. Additionally, Railroad Park includes outdoor gym equipment similar to Muscle Beach for outdoor fitness among young adults. Finally, the park includes a large amount of green space and trails for walking and running. The park is open daily from 7 am to 11 pm, and monitored around the clock by a security system and by rangers on patrol." - *Description of additional intervention components*: n/a - *Duration of intervention*: September 2010 (date of completing park construction) - ongoing (permanent intervention) - *How was the intervention implemented?*: Construction of Railroad park; a 19-acre green space. - *Co-interventions*: N/A - *Where was the intervention implemented?*: "...inner-city of B Birmingham, Alabama" an area primarily serving low-income, African American Children - *Resource requirements to replicate the intervention*: NR - *Economic indicators*: NR   Control: No intervention |
| **Identification** | **Sponsorship source:** Funded in part by National Institutes of Health (NIH) grants P30DK056336, T32DK062710-01A1, R25HL124208, R25DK099080, and R25GM116167  **Country:** United States  **Setting:** "...inner-city of Birmingham, Alabama" an area primarily serving low-income, African American Children  **Comments:**  **Author’s name:** TaShauna U. Goldsby  **Institution:**  **Email:** tgoldsby@uab.edu  **Address:**  **Declaration of interests stated?:** Yes. "We have no relevant financial disclosures or conflicts of interest with respect to this manuscript."  **Specify any documents relevant to this study:**  **Study or programme name or acronym:** n/a  **Type of record:** Journal Article |
| **Notes** |  |

## Risk-of-bias table

| **Bias** | **Authors' judgement** | **Support for judgement** |
| --- | --- | --- |
| Random sequence generation (selection bias) | High risk | This was a CBA. |
| Allocation concealment (selection bias) | High risk | This was a CBA. The participants and assessors could foresee assignment into intervention or control depending on proximity to the park. However, it is not clear if such would have introduced selection bias given that only EHR data was used for recruitment. |
| Baseline characteristics similar | Low risk | The baseline characteristics among participants in intervention groups and the control group are reported; no statistical test for their differences is provided. However, covariates (Age, gender, race (African American or not), Ethnicity (Hispanic or not), and payer type) are adjusted! |
| Baseline outcome measurements similar | Low risk | The baseline outcome measurements are similar in intervention and control areas; and data analysis adjusted for any inherent differences. |
| Blinding of participants and personnel (performance bias) | Low risk | Blinding was not possible due to the nature of the intervention. This is unlikely to have influenced participants' or personnel's behavior. |
| Blinding of outcome assessment (detection bias) | Low risk | Outcome data by nurses at clinics as part of regular health visits and captured on the electronic health records. Therefore, blinding doesn't apply in this study. |
| Protection against contamination | High risk | People in the control group could have gone to the park. Distance from park in intervention group is within 5 miles (8km) of the park, and more than 5 miles for the control group. |
| Incomplete outcome data (attrition bias) | Unclear risk | Not reported |
| Selective reporting (reporting bias) | Low risk | The outcomes reported are consistent in both methods and results section. |
| Other bias | Unclear risk | Misclassification of exposure: low risk. Measurement bias: unclear risk. Routinely collected data may not be highly accurate. Incorrect analysis: n/a. |

# Goodman 2013

| **Methods** | **Study design:** CBA  **Study grouping:** Parallel group  **How was missing data handled?:** NR  **How were participants recruited and sampled within the study sites?:** Census data were used, i.e. all individuals targeted for survey  **Methods for selecting intervention and control sites:** 1. "These 17 [intervention] towns and one city (henceforth ‘towns’) were chosen through a competitive process whereby local authorities submitted detailed plans to the Department for Transport... They were then selected on the basis of strong leadership; a deliverable strategy that seemed likely substantially to increase cycling; and evidence of local matched funding" 2. "CDT/CCT status was not assigned at random, but rather was awarded to towns which (1) applied for funding and (2) best met the criteria of leadership, strategy and evidence for matched funding." 3. "...but instead sought to make our evaluation more robust to confounding by defining multiple comparison groups with complementary strengths. These were: a matched comparison group (selected a priori as the primary comparator); an unfunded comparison group; and a non-London national comparison group."  **Number of control sites:** Three separate comparisons analyzed: Matched comparison: 18 sites; Unfunded comparison: 67 sites; National comparison: 282 sites (see Tables 1 and 2)  **Number of intervention sites:** 18 intervention sites  **Post-intervention period (start and end date or year it was conducted):** CDTs: October 2005 and March 2011; CCTs: April 2008 and March 2011. Post-intervention data from 2011.  **Pre-intervention time period (before intervention start):** 2001 (NOTE: interventions were implemented from 2005-2011 and 2008-2011, yet data collection only occurred one time pre-intervention).  **Sample size justification and outcome used:** No sample size justification; all available data were assessed.  **Study aim or objective:** "We therefore evaluated all 18 town-wide cycling initiatives with a large and representative dataset derived from the English census. Our primary aim was to examine whether the prevalence of cycling to work increased in intervention towns relative to matched comparison towns. Our secondary aims were to examine: (1) changes in the prevalence of walking, driving or using public transport to travel to work; (2) whether effects differed by levels of small-area deprivation; and (3) whether intervention effects differed between towns."  **Time points of data collection:** Baseline: 2001. Follow-up: 2011.  **Unit of allocation or exposure:** See table 2; 'units' represented the analyzed towns and were defined as "contiguous urban areas forming units of analysis." |
| --- | --- |
| **Participants** | **Baseline Characteristics**  Intervention   - *Age*: 16-74 years old - *Place of residence*: Urban areas outside London - *Sex*: NR - *Ethnicity and language*: NR - *Occupation*: NR, but currently employed - *Education*: NR - *Socioeconomic status*: Affluence (IQR, 2010): 46.1 (26.1, 71.2) - *Social capital*: NR   Control   - *Age*: 16-74 years old - *Place of residence*: Urban areas outside London - *Sex*: NR - *Ethnicity and language*: NR - *Occupation*: NR, but currently employed - *Education*: NR - *Socioeconomic status*: Matched: Median affluence rank, 2010 (IQR): 44.0 (23.4, 68.9); Unfunded: Median affluence rank, 2010 (IQR): 35.9 (35.9 (14.8, 59.9); National: Median affluence rank, 2010 (IQR): 43.4 (20.6, 69.4) - *Social capital*: NR   Overall   - *Age*: 16-74 years old - *Place of residence*: Urban areas outside London - *Sex*: NR - *Ethnicity and language*: NR - *Occupation*: NR, but currently employed - *Education*: NR - *Socioeconomic status*: NR - *Social capital*: NR   **Included criteria:** None described; survey data were used, the question from which the outcome was calculated was relevant for individuals 16-74 who work somewhere other than their private address. It can likely be assumed that all individuals for which outcome data were available were included in the analysis.  **Excluded criteria:** NR  **Pretreatment:** See table 2. With respect to the primary outcome, % cycling to work, pre-intervention levels were higher at intervention sites than at all three types of comparison sites: Intervention sites: 5.81 (5.77, 5.86) Matched control sites: 4.03 (3.99, 4.08) Unfunded control sites: 3.47 (3.45, 3.49) National control site: 3.38 (3.37, 3.39) Population density and affluence were not assessed pre-intervention, but in 2011 and 2010, respectively. There were slight, likely not meaningful differences between groups with regard to population density, yet potentially meaningful differences in affluence (intervention sites more affluent than all three types of control sites).  **Attrition per relevant group:** NR  **How were baseline differences assessed?:** Pre-intervention outcomes are listed in Table 2; these are not further analyzed. However, the difference-in-differences analysis applied means that these slight differences are likely not relevant.  **Total number completed and analysed per relevant group:** Intervention: 1,266,337 Matched control: 969,605 Unfunded control: 4,195,540 National control: 10,356,452  **Total number in each relevant group pre-intervention:** NR  **Total number randomised per relevant group:** No randomization done. "CDT/CCT status was not assigned at random, but rather was awarded to towns which (1) applied for funding and (2) best met the criteria of leadership, strategy and evidence for matched funding." |
| **Interventions** | **Intervention Characteristics**  Intervention   - *Type of intervention (e.g. infrastructure, policy or both)*: Infrastructure - *Description of main intervention*: [See Box 1 for full description] The 18 intervention towns were located across England (see Supplementary File S1) and varied considerably in their size, cycling infrastructure and cycling cultures. Initiatives were therefore tailored to each setting, but all towns spent a mixture of capital investment (e.g. building cycle lanes, creating cycle parking) and revenue investment (e.g. promotional activities, cycle training), with an average capital:revenue ratio of 3:1. Investment themes included: 1) Cycling to workplaces; 2) Schools and colleges; 3) General infrastructure improvements; 4) Cycling to stations; 5) Targeting specific neighborhoods or groups - *Description of additional intervention components*: Towns implemented educational and promotional activities as part of the interventions, e.g. "workplaces to become more cycle friendly by helping them develop travel plans, services offered directly to employees, such as free personalised travel planning, cycling ‘taster’ sessions, on-site cycle repairs and training in cycle maintenance. A third category of components aimed to create a supportive social and organisational culture for cycling, for example by organising Bicycle User Groups, ‘bike breakfasts’ and annual workplace cycling challenges. All towns invested in ‘Bikeability’ cycle training in schools, with over 32,000 pupils receiving basic, off-road training and 46,000 receiving more advanced, on-road training between 2008 and 2011." - *Duration of intervention*: There were two different sets of intervention towns; the six Cycling Demonstration Towns (CDTs) received the intervention October 2005 - March 2011, while the 12 Cycling Cities and Towns received the intervention from April 2008 - March 2011. - *How was the intervention implemented?*: The 18 towns "were chosen through a competitive process whereby local authorities submitted detailed plans to the Department for Transport. They were then selected on the basis of strong leadership; a deliverable strategy that seemed likely substantially to increase cycling; and evidence of local matched funding. In each selected town, dedicated specialist cycling teams designed and delivered a tailored programme of interventions that aimed to deliver a marked and visible improvement in the facilities for cycling and in the profile of cycling." - *Co-interventions*: NR - *Where was the intervention implemented?*: In 18 towns chosen through competitive process, they were located across England. - *Resource requirements to replicate the intervention*: NR - *Economic indicators*: The first set of intervention towns increased their spending on cycling to £17 per person per year, through a combination of central government andmatched local funding. The second set of intervention towns increased their funding to £14 per person per year.   Control: No intervention |
| **Identification** | **Sponsorship source:** "DO and SS are supported by the Medical Research Council (Unit Programme number MC_UP_1001/1) and DO is also supported by the Centre for Diet and Activity Research (CEDAR), a UKCRC Public Health Research Centre of Excellence. Funding for CEDAR from the British Heart Foundation, Economic and Social Research Council, Medical Research Council, NIHR and Wellcome Trust, under the auspices of the UK Clinical Research Collaboration, is gratefully acknowledged. AG and JP are funded by National Institute of Health Research (NIHR) post-doctoral fellowships."  **Country:** UK  **Setting:** "...urban areas of England outside London"  **Comments:**  **Author’s name:** Anna Goodman  **Institution:**  **Email:** anna.goodman@lshtm.ac.uk  **Address:**  **Declaration of interests stated?:** NR  **Specify any documents relevant to this study:**  **Study or programme name or acronym:** N/A  **Type of record:** Journal article |
| **Notes** | Linked studies: Goodman 2014(14) |

## Risk-of-bias table

| **Bias** | **Authors' judgement** | **Support for judgement** |
| --- | --- | --- |
| Random sequence generation (selection bias) | High risk | CBA study; intervention sites applied and were selected to receive the intervention, while three various types of control site were chosen for the analysis. |
| Allocation concealment (selection bias) | High risk | CBA study; the study authors had control of the control site selection process. |
| Baseline characteristics similar | Low risk | DiD analysis was conducted, these differences did not likely lead to bias. |
| Baseline outcome measurements similar | Low risk | Quote: "Proximity to Connect2 was likewise not associated with preintervention activity levels but was strongly and progressively associated with greater use of Connect2"  Judgement Comment: There were differences in the outcome (see Table 2), however, given that a DiD analysis was conducted, these differences did not likely lead to bias. |
| Blinding of participants and personnel (performance bias) | Low risk | Participants and personnel were not blinded; due to the nature of the intervention, however, it is unlikely that this led to meaningful bias. |
| Blinding of outcome assessment (detection bias) | High risk | Outcome assessors were not blinded; as outcomes were defined based on already collected census data, it is unlikely that this led to meaningful bias in the study. other Data was collected through self-reported surveys and respondents answers may have been influenced by knowledge of the intervention |
| Protection against contamination | Low risk | It is conceivable that some contamination occurred, e.g. through individuals travelling between towns, however, given the large numbers analyzed, it is unlikely that this meaningfully biased results. |
| Incomplete outcome data (attrition bias) | High risk | Other Data was collected through self-reported surveys and respondents answers may have been influenced by knowledge of the intervention |
| Selective reporting (reporting bias) | Low risk | No evidence of selective reporting; all analyses described in the methods are reported in the results. |
| Other bias | High risk | Study authors do not report what other changes (co-interventions) happened in the study period. Given that only two data points were analyzed, over 10 years apart, other changes in those 10 years could have meaningfully biased results. |

# Green 2014

| **Methods** | **Study design:** CBA study  **Study grouping:** Parallel group  **How was missing data handled?:** "Missing journey times and missing distances were estimated using the median times and distances for each age group and travel mode. Where reported times and distances were deemed implausible, these were treated as missing and replacement values were imputed."  **How were participants recruited and sampled within the study sites?:** Data is from routinely conducted surveys or routinely collected data with residents across London. "The LATS and LTDS include randomly sampled London households and are comparable as they use similar sampling designs and daily travel diaries. The sampling design is multistage, using postcode geography as primary sampling units and households selected at random at the second stage of sampling. LATS surveyed 30,000 London households in 2001 and LTDS has surveyed 8000 London households each year since 2006."  **Methods for selecting intervention and control sites:** There are no intervention sites. The intervention group is the age group benefiting from the free bus travel policy (12-17 years) and the control group is the age group that did not benefit from the policy (25-59 years).  **Number of control sites:** One group: adults (25-59 years)  **Number of intervention sites:** One group: young people (12-17 years) (a year later it was extended to those younger than 18 years old).  **Post-intervention period (start and end date or year it was conducted):** Policy introduced in 2005 (data collected until 2008)  **Pre-intervention time period (before intervention start):** Before 2005 (data collected in 2001)  **Sample size justification and outcome used:** "The LATS and LTDS samples include data on around 3000 young people before and after the intervention (Table 1), giving over 80% power to detect a 10% relative reduction in average distances walked daily by young people [i.e. from 0.9 (standard deviation; SD 1.3) km to 0.8 (SD 1.3) km per day] at a 5% significance level. Similarly, the study will have over 90% power to detect a 10% increase in the average distance of bus travel [i.e. from 4.3 (SD 4.1) km to 4.7 (SD 4.1) km per day]. For transport-related injury, the study would have 80% power to detect a 10% change, or 90% power to detect a 12% change significant at the 5% level. Statistical power is inevitably more limited for subgroup analyses, but there will be 90% power to detect a 15% change in average distance travelled by bus by young people within the most deprived quartile, for example."  **Study aim or objective:** "This study aimed to evaluate the impact of free bus travel on public health, using a mixed-method design, and to assess the economic costs and benefits of the scheme. Our specific aims were to: l provide empirical evidence for the impact of this intervention on key health behaviours and outcomes (e.g. injuries, active travel) for young people l explore the effects on the determinants of health (e.g. access to education and training) l identify the effects of increased young people’s access to bus travel on older citizens l develop and apply methods for economic assessment, and l contribute to the development of methods to strengthen causal inference in non-randomised designs."  **Time points of data collection:** Pre-intervention: London Area Transport Survey (LATS) (2001). Post-intervention: London Travel Demand Survey (LTDS) (2005–8).  **Unit of allocation or exposure:** Age groups |
| --- | --- |
| **Participants** | **Baseline Characteristics**  Intervention   - *Age*: 12-17 years old - *Place of residence*: London, UK - *Sex*: NR - *Ethnicity and language*: NR - *Occupation*: NR - *Education*: NR - *Socioeconomic status*: NR - *Social capital*: NR   Control   - *Age*: 25-59 years old - *Place of residence*: London, UK - *Sex*: NR - *Ethnicity and language*: NR - *Occupation*: NR - *Education*: NR - *Socioeconomic status*: NR - *Social capital*: NR   Overall: NR  **Included criteria:** NR. Any person older than 5 years old in selected households completed the 1-day travel diary.  **Excluded criteria:** NR  **Pretreatment:** Characteristics NR. According to graphs presented, the number of bus trips and bus distance travelled per day was higher in younger (target) group compared to older (control) group; the percentage of short trips by bus appear similar.  **Attrition per relevant group:** NR. Data based on routinely collected data (annual surveys). Any missing data was imputed.  **How were baseline differences assessed?:** NR  **Total number completed and analysed per relevant group:** Control group: 14085 Intervention group: 2024. LATS/LTDS - approx 3000 people before and after the intervention (for transport related outcomes). unclear for other outcomes and data sets.  **Total number in each relevant group pre-intervention:** Control group: 31169 Intervention group:4206 (???)  **Total number randomised per relevant group:** N/A |
| **Interventions** | **Intervention Characteristics**  Intervention   - *Type of intervention (e.g. infrastructure, policy or both)*: Policy - *Description of main intervention*: "In 2005, the Greater London Authority (GLA) granted secondary school-aged children unlimited travel on buses and trams displaying the London Buses symbol (both within and just outside London,12 replacing a reduced, 40p flat fare for each journey on the London bus network. This fare exemption was extended a year later to include 17-year-olds in full-time education (p. 7)13 and now also includes all 18-year-olds (and some 19-year-olds) in full-time education or on a work-based learning scheme." - *Description of additional intervention components*: NR - *Duration of intervention*: 3 years (2005-2008) (still ongoing) - *How was the intervention implemented?*: "To access free travel, young people apply for an electronic photo card called a ‘zip’ card, which is tapped on a reader on entering the bus. As well as granting the cardholder unlimited free travel on all buses, the zip card also acts as a conventional ‘Oyster’ card, used by most residents and visitors in London. This can be loaded with pre-pay or travelcards for the cardholders to use on other parts of the Transport for London (TfL)network [tube, Docklands Light Railway (DLR), London overground and most National Rail services operating in the capital] at a discounted rate (pp. 6–11)." - *Co-interventions*: "Car ownership is lower in London than in other areas of the UK, and a number of policies in addition to the expansion of public transport aim to reduce car use." "The free bus travel scheme introduced for young people in London in 2005 was introduced not in isolation, but within a context of other explicit policies and secular changes that shape the ways that people in London travel and which potentially impact on health." "London has also experienced the introduction of other transport policies over the study period (e.g. London congestion charge was introduced in 2003), which may have altered choices of travel mode within the population." - *Where was the intervention implemented?*: London - *Resource requirements to replicate the intervention*: NR - *Economic indicators*: Administrative costs (i.e. issuing of cards: £3,608,000). additional Bus operating costs: £1,723,700 per annum   Control   - *Type of intervention (e.g. infrastructure, policy or both)*: No intervention - *Description of main intervention*: Older participants were not beneficiaries of the new policy - *Description of additional intervention components*: N/A - *Duration of intervention*: N/A - *How was the intervention implemented?*: N/A - *Co-interventions*: "Car ownership is lower in London than in other areas of the UK, and a number of policies in addition to the expansion of public transport aim to reduce car use." "The free bus travel scheme introduced for young people in London in 2005 was introduced not in isolation, but within a context of other explicit policies and secular changes that shape the ways that people in London travel and which potentially impact on health." "London has also experienced the introduction of other transport policies over the study period (e.g. London congestion charge was introduced in 2003), which may have altered choices of travel mode within the population." - *Where was the intervention implemented?*: N/A - *Resource requirements to replicate the intervention*: N/A - *Economic indicators*: N/A |
| **Identification** | **Sponsorship source:** Funding for this study was provided by the Public Health Research programme of the National Institute for Health Research.  **Country:** UK  **Setting:** London  **Comments:**  **Author’s name:** Judith Green  **Institution:** Department of Health Services Research and Policy, London School of Hygiene and Tropical Medicine, London, UK  **Email:** NR  **Address:**  **Declaration of interests stated?:** "Declared competing interests of authors: Helen Roberts is a member of the NIHR Journals Library editorial board."  **Specify any documents relevant to this study:** n/a  **Study or programme name or acronym:** n/a  **Type of record:** Report |
| **Notes** |  |

## Risk-of-bias table

| **Bias** | **Authors' judgement** | **Support for judgement** |
| --- | --- | --- |
| Random sequence generation (selection bias) | High risk | CBA study |
| Allocation concealment (selection bias) | High risk | CBA study |
| Baseline characteristics similar | Unclear risk | NR |
| Baseline outcome measurements similar | Unclear risk | Some outcomes appear different at baseline but formal analyses not conducted |
| Blinding of participants and personnel (performance bias) | Low risk | Data based on routinely collected data (i.e. national surveys). Participants are aware of the interventions. Unlikely to have introduced performance bias. |
| Blinding of outcome assessment (detection bias) | Low risk | Data based on routinely collected data beyond the purpose of the current study. |
| Protection against contamination | Unclear risk | Exposure to intervention was according to age group. it is possible that some in the intervention group did not participate in the intervention. |
| Incomplete outcome data (attrition bias) | Low risk | Missing or implausible data were imputed. |
| Selective reporting (reporting bias) | Low risk | Protocol outlines same outcomes and analysis (check). |
| Other bias | Low risk | Misclassification bias of exposure: low risk; exposure determined by age group. |

# Grunseit 2019

| **Methods** | **Study design:** Interrupted time-series study  **Study grouping:**  **How was missing data handled?:** NR  **How were participants recruited and sampled within the study sites?:** No participants assessed (relevant for this review); relevant data stem from two infrared pyroelectric counters.  **Methods for selecting intervention and control sites:** Two intervention sites selectively chosen because of differences in nature: one near entrance at busy road; one near entrance from low-traffic residential area.  **Number of control sites:** NA  **Number of intervention sites:** 2  **Post-intervention period (start and end date or year it was conducted):** 25 February - 14 July 2015  **Pre-intervention time period (before intervention start):** 3 November 2012 - 24 February 2015  **Sample size justification and outcome used:** NR  **Study aim or objective:** "This paper focuses on one such evaluation of the impact of the completion of a recreational trail located in a suburb of Sydney, Australia on PA and trail usage, including the use by different subpopulations."  **Time points of data collection:** Approximately 120 time points (one data point per week throughout study period.)  **Unit of allocation or exposure:** Intervention site |
| --- | --- |
| **Participants** | **Baseline Characteristics**  Intervention   - *Age*: See Table 2 for proxy - *Place of residence*: See Table 2 for proxy - *Sex*: See Table 2 for proxy - *Ethnicity and language*: See Table 2 for proxy - *Occupation*: See Table 2 for proxy - *Education*: See Table 2 for proxy - *Socioeconomic status*: See Table 2 for proxy - *Social capital*: See Table 2 for proxy   Control: NA  Overall: NA  **Included criteria:** NA (all count data used)  **Excluded criteria:** NA (all count data used)  **Pretreatment:** NA  **Attrition per relevant group:** NA (all count data used)  **How were baseline differences assessed?:** NA  **Total number completed and analysed per relevant group:** NR  **Total number in each relevant group pre-intervention:** NR  **Total number randomised per relevant group:** NA |
| **Interventions** | **Intervention Characteristics**  Intervention   - *Infrastructure*: Completion of the Narrabeen Lagoon Trail, a recreational loop trail for cyclists and pedestrians - *Description of main intervention*: "Multi-use recreational walking and cycling loop trail (a trail which connects back with itself when travelling continuously in one or the other direction) located in a densely populated area of Northern Sydney, Australia...The local council implemented the project which involved building new bridges, 2 km of new boardwalk, reserve and car park upgrades, a boat ramp, toilet facility upgrades, park furniture, rest stops, vantage outlook points, heritage restoration, environmental protection and substantial planting of local vegetation." - *Description of additional intervention components*: NA - *Duration of intervention*: Permanent after construction (beginning 2010; completion 2015) - *How was the intervention implemented?*: "The trail has been undergoing development since 2010 with the final stage of the trail opening on 25th February 2015. Completion of the final section means that the 8.5km trail fully circumnavigates Narrabeen Lagoon linking...The local council implemented the new project..." - *Co-interventions*: NR - *Where was the intervention implemented?*: "...a densely populated area of Northern Sydney, Australia. It runs through bushland, parks and passes by amenities such as parking areas, other recreational activities (ie., watercraft hire) and cafes/restaurants." - *Resource requirements to replicate the intervention*: " $AUD 11.4 million [31]. The trail was 60% funded by the then Warringah (now Northern Beaches) Council, and supported by State and Federal grants programs." - *Economic indicators*: NR   Control: NA |
| **Identification** | **Sponsorship source:** "This work was in part supported by Warringah Council for the visual count and survey data collection."  **Country:** Australia  **Setting:** Densely populated suburban Sydney  **Comments:**  **Author’s name:** Dafna Merom  **Institution:**  **Email:** d.merom@uws.edu.au  **Address:**  **Declaration of interests stated?:** "The authors declare that they have no competing interests."  **Specify any documents relevant to this study:** NR  **Study or programme name or acronym:** NA  **Type of record:** Journal article |
| **Notes** |  |

## Risk-of-bias table

| **Bias** | **Authors' judgement** | **Support for judgement** |
| --- | --- | --- |
| ITS - Were incomplete outcome data addressed? | Low risk | Not discussed, however given the nature of the outcome collection (routine collection using automated counters), it is unlikely that missing data was a substantial problem in the study. |
| ITS - Was knowledge of the allocated interventions adequately prevented during the study | Low risk | Allocation was not concealed, however given the nature of the intervention and outcome assessed, this should not have substantially influenced results. |
| ITS - Was the study free from other bias? | Low risk | Authors describe a small risk of undercounting with automated counters, however this is only in the magnitude of a few percent, and unlikely to alter the results meaningfully. Otherwise no further sources of bias identified. |
| ITS - Was the shape of intervention effect prespecified? | Unclear risk | “Not discussed, however given the nature of the outcome collection (routine collection using automated counters), it is unlikely that missing data was a substantial problem in the study.” |
| ITS - Was the study free from selective reporting | Low risk | Reporting of outcome data is clear and comprehensive, no apparent issues with selective reporting. |
| ITS - Was the intervention unlikely to affect data collection? | Low risk | Automated count data would not have been affected by the implementation of the intervention. |
| ITS - Was the intervention independent of other changes? | Unclear risk | There is no information explaining the intervention independence |

# Higgerson 2018

| **Methods** | **Study design:** ITS study  **Study grouping:**  **How was missing data handled?:** Missing data was excluded from analysis (both ITS and CBA analyses).  **How were participants recruited and sampled within the study sites?:** ITS study - Convenience sample, based who chose to take up the free access offer. Data was extracted from the leisure management IT system. Intervention was implemented in an England local authority, and free access to leisure facilities was available to people living, registered or registered with a general practitioner in this Local Authority. Over time different age groups had access to the free access; with more age groups being able to access them at later stages. To raise awareness/recruit people to participate in this programme there were extensive marketing and promotional activities and Healthy Community Partnerships with volunteers running community events and acting as buddies for those attending their first activity sessions. CBA study - "random sample from each LA in England selected using Random Digit Dialing, drawing data from a large national annual survey of sports participation (the Active People Survey). One person aged ≥16 is randomly selected from eligible household members."  **Methods for selecting intervention and control sites:** ITS study - no control site. Nine leisure facilities in Blackburn with Darwen. There was data for 14 quarters before the intervention and 26 quarters after the intervention. CBA study based on a random sample of repeated cross-sectional national surveys, where intervention was Blackburn with Darwen and the control was the rest of England.  **Number of control sites:** ITS study - 9 leisure facilities (these were intervention and control). CBA study - rest of England  **Number of intervention sites:** ITS study - 9 leisure facilities (these were intervention and control). CBA study - 1: Blackburn with Darwen  **Post-intervention period (start and end date or year it was conducted):** July 2008 to 2014, but programme still ongoing. In 2016 intervention change to charge flat fee of 1 pound.  **Pre-intervention time period (before intervention start):** 2 years: 2005 to 2007  **Sample size justification and outcome used:** NR  **Study aim or objective:** "We therefore investigated the impact of the re:fresh scheme, introduced in Blackburn with Darwen, a deprived LA in the northwest of England in 2008, that provided free access to activities in leisure centres (swimming pools and gyms) at most times of the day along with community outreach activities. We use quasi-experimental methods to investigate whether the scheme led to an increase in swimming and gym activities and overall levels of physical activity and whether these effects differed by socioeconomic group."  **Time points of data collection:** ITS: "...data extracted from the Leisure Management System, to conduct an interrupted time series (ITS) analysis investigating the relative change in attendances associated with the introduction of re:fresh." Data related to the number of attendances for the 14 quarters (ie, 3-month periods) before the intervention, from 2005 to 2007, and 26 quarters after the intervention, from 2008 to 2014. CBA: National annual survey (Active People's Survey - APS). "The interviews for each survey are evenly spread across 12 months, running from October of one year to October of the next year. We used data for all surveys from APS1 (2005–2006) to APS9 (2014–2015)". "There was a gap from October 2006 to October 2007 when no survey was completed."  **Unit of allocation or exposure:** Individuals |
| --- | --- |
| **Participants** | **Baseline Characteristics**  Intervention   - *Age*: NR - *Place of residence*: NR - *Sex*: NR - *Ethnicity and language*: 31% of the population in Blackburn with Darwen were from a black or minority ethnic group - *Occupation*: NR - *Education*: NR - *Socioeconomic status*: NR - *Social capital*: NR   Control   - *Age*: NR - *Place of residence*: ITS: Blackburn with Darwen LA. CBA: Rest of England - *Sex*: NR - *Ethnicity and language*: NR - *Occupation*: NR - *Education*: NR - *Socioeconomic status*: NR - *Social capital*: NR   Overall   - *Age*: NR - *Place of residence*: Deprived Local Authority in England - *Sex*: NR - *Ethnicity and language*: "31% of the population were from a black or minority ethnic group." - *Occupation*: NR - *Education*: NR - *Socioeconomic status*: NR - *Social capital*: NR   **Included criteria:** "people living, working or registered with a general practitioner in Blackburn with Darwen." "Initially in July 2008 the free offer was only available to people >50 years old, being extended to people aged 16–24 years in September 2008 and finally to people aged 25–49 years in April 2009."  **Excluded criteria:** None reported.  **Pretreatment:** ITS analysis without a control group. CBA - NR  **Attrition per relevant group:** ITS analysis - Missing data not reported. CBA - "Average response rates are low, ranging from 27.1% to 27.8% during the study period". "Data in the sample were missing on age for 2.2%, ethnicity for 1.8% and socioeconomic status for 2.2%. A further 7.4% of the sample was excluded as their socioeconomic status was unclassifiable based on their reported occupation."  **How were baseline differences assessed?:** ITS - not relevant CBA - NR  **Total number completed and analysed per relevant group:** ITS - NRCBA - n=1,556,563 (n= 6,160 Blackburn with Darwen, n= 1,550,403 from the other LA areas of England)  **Total number in each relevant group pre-intervention:** ITS - NRCBA - total sample n= 1,763,780  **Total number randomised per relevant group:** n/a |
| **Interventions** | **Intervention Characteristics**  Intervention   - *Type of intervention (e.g. infrastructure, policy or both)*: Infrastructure - *Description of main intervention*: "The re:fresh scheme began in July 2008, with the provision of free access to local government leisure facilities at most times of the day for people living, working or registered with a general practitioner in Blackburn with Darwen. At the time, there were nine leisure facilities in Blackburn with Darwen. Three of these facilities included swimming pools and gyms, one facility just had a swimming pool and five sites had gym facilities only... Several of the leisure facilities were located close to deprived neighborhoods...". Overall during the scheme, free leisure was available for 90% of the opening hours of the nine facilities. - *Description of additional intervention components*: The free offer was supported by outreach work delivered by Health Trainers and a Healthy Communities Partnership. Five full time equivalent (FTE) Health Trainers were employed during the project, offering 1 to 1 and group sessions, to around 700 inactive people per year supporting behaviour change through goal setting and motivational interviewing. Two FTE community workers delivered the Healthy Communities Partnership which supported a network of volunteers who ran community events to engage people in taster sessions and increase the awareness of re:fresh, and act as buddies to accompany people to their first activity sessions. The programme was also supported by considerable marketing and promotional activity to raise awareness of the offer and to promote participation. The scheme is ongoing, although in 2016a flat fee of £1 was introduced for previously free activities in response to cuts in local government funding." - *Duration of intervention*: July 2008 - still ongoing (data for analysis from 2014) - *How was the intervention implemented?*: Local authority leisure facilities ensured free access for eligible population. Marketing and community outreach helped promote awareness and participation in the scheme. - *Co-interventions*: None reported - *Where was the intervention implemented?*: Leisure facilities of local authority in England (Blackburn with Darwen) - *Resource requirements to replicate the intervention*: "Five full time equivalent (FTE) Health Trainers were employed during the project, offering 1 to 1 and group sessions, to around 700 inactive people per year supporting behaviour change through goal setting and motivational interviewing. Two FTE community workers delivered the Healthy Communities Partnership which supported a network of volunteers who ran community events to engage people in taster sessions and increase the awareness of re:fresh, and act as buddies to accompany people to their first activity sessions." - *Economic indicators*: "Between 2008 and 2014, the NHS contributed a total of £6 million on top of the core funding for leisure facilities provided by the council over this period (£22 million). The outreach activities cost approximately £2 million over this time."   Control: No intervention |
| **Identification** | **Sponsorship source:** "This work (project reference: SPHR-LIL-PES-LAL) was supported and funded by the NIHR School for Public Health Research (SPHR) Public Health Practice Evaluation Scheme (PHPES). BB was supported by the National Institute for Health Research (NIHR) Collaboration for Leadership in Health Research and Care (CLAHRC NWC)."  **Country:** England  **Setting:** Deprived local authority in the Northwest of England: Blackburn with Darwen  **Comments:**  **Author’s name:** Ben Barr  **Institution:**  **Email:** benbarr@ liverpool.ac.uk  **Address:**  **Declaration of interests stated?:** Yes. "RB is employed by Blackburn with Darwen Council in their Leisure and Environment department, involved in providing information about the nature of the intervention, facilitating access to the data, and providing contextual information upon request when interpreting the results."  **Specify any documents relevant to this study:** Supplementary appendices 1 to 6  **Study or programme name or acronym:** re:fresh scheme  **Type of record:** Journal article |
| **Notes** |  |

## Risk-of-bias table

| **Bias** | **Authors' judgement** | **Support for judgement** |
| --- | --- | --- |
| ITS - Were incomplete outcome data addressed? | Unclear risk | The authors do not report on missing data. |
| ITS - Was knowledge of the allocated interventions adequately prevented during the study | Low risk | Outcomes are objective, e.g. number of swim and gym attendances, % of people reporting at least one moderate gym or swim session in the previous four weeks. |
| ITS - Was the study free from other bias? | Low risk | Unlikely that there was seasonal bias because data was collected from 2005 to 2014 across yearly quarters. |
| ITS - Was the shape of intervention effect prespecified? | Low risk | Intervention began in July 2018 and the intervention point in the model is the 3rd quarter of 2008. |
| ITS - Was the study free from selective reporting | Low risk | All relevant outcomes in the methods section are reported in the results section. |
| ITS - Was the intervention unlikely to affect data collection? | Low risk | Data collection methods were not changed from pre to post intervention period "...we extracted data from the leisure management IT system...we used data from a large national annual survey of sports participation—the Active People Survey". |
| ITS - Was the intervention independent of other changes? | Unclear risk | No specific events/variables that could have influenced outcome were mentioned but authors state that "however, it may still be subject to bias if there were other unobserved determinants of physical activity that changed around the same time as the intervention" "It is also possible that the effectiveness of the scheme may also have been contingent on other factors in Blackburn with Darwen." |

# Hirsch 2017

| **Methods** | **Study design:** Controlled before-after study  **Study grouping:**  **How was missing data handled?:** NR  **How were participants recruited and sampled within the study sites?:** "This longitudinal repeated cross-sectional study used data from 116 tracts (based on 2010 Census boundary delineations) for Minneapolis. Among U.S. cities, Minneapolis represents an ideal city to understand the influence of bicycling infrastructure on commuting by bicycle."  **Methods for selecting intervention and control sites:** The main comparison of the study is 2000 vs. 2010; there is no explicit group comparison. Stratified analyses are conducted based on the quartile distance from the trail system; we can utilize this to compare the 25h percentile (within 1.08 km from the trail) with the 75th percentile (within 5.91 km of the trail system) to approximate a comparison of individuals likely exposed and likely not exposed to the intervention. These 'groups', however, were not directly compared with one another.  **Number of control sites:** 1 - those living at the 5.91 km from the trail system  **Number of intervention sites:** 1 - those living 1.08 km of the trail system  **Post-intervention period (start and end date or year it was conducted):** 2010  **Pre-intervention time period (before intervention start):** 2000  **Sample size justification and outcome used:** NR  **Study aim or objective:** "Data from Minneapolis, Minnesota was used to test whether increases in bicycle commuting between 2000 and 2010 are associated with implementation of an off-road trail system specifically designed to promote bicycle commuting."  **Time points of data collection:** 2 (one pre-, one post-intervention)  **Unit of allocation or exposure:** Area-level (likely exposed vs. likely unexposed, based on difference from trail system). |
| --- | --- |
| **Participants** | **Baseline Characteristics**  Off-road trail system   - *Age*: - *Place of residence*: - *Sex*: - *Ethnicity and language*: - *Occupation*: - *Education*: - *Socioeconomic status*: - *Social capital*:   No new intervention   - *Age*: - *Place of residence*: - *Sex*: - *Ethnicity and language*: - *Occupation*: - *Education*: - *Socioeconomic status*: - *Social capital*:   Overall   - *Age*: % workers 18-34 years old: 33.2 (12.6) - *Place of residence*: NR - *Sex*: NR - *Ethnicity and language*: % non-Hispanic white: 60.9 (26.6) - *Occupation*: % professional employment: 38.1 (14.7) - *Education*: % college graduate: 34.6 (19.7) - *Socioeconomic status*: Median household income (x$10000): 5.52 (2.2) - *Social capital*: NR   **Included criteria:** Census data for the city used for the analyses; data for the entire city included.  **Excluded criteria:** Census data for the city used for the analyses; no specific data were excluded.  **Pretreatment:** The main comparison of the study is 2000 vs. 2010; there is no explicit group comparison. Stratified analyses are conducted based on the quartile distance from the trail system; we can utilize this to compare the 25h percentile (within 1.08 km from the trail) with the 75th percentile (within 5.91 km of the trail system) to approximate a comparison of individuals likely exposed and likely not exposed to the intervention.  **Attrition per relevant group:** NA - census data at two points assessed; all data assessed.  **How were baseline differences assessed?:** Means and standard deviations of groups 2000 and 2010 were compared using the Wilcoxon rank test.  **Total number completed and analysed per relevant group:** 116 census tracts were analyzed at both time points. The mean (SD) tract population was 3299 (1180) in 2000 and 3319 (1299) in 2010.  **Total number in each relevant group pre-intervention:** NR  **Total number randomised per relevant group:** NA |
| **Interventions** | **Intervention Characteristics**   - *Type of intervention (e.g. infrastructure, policy or both)*: Infrastructure - *Description of main intervention*: "...additions of the Hiawatha Trail (4.7 miles) and Midtown Greenway (5.5 miles) which provide 10.2 miles of off-road paved paths transecting the city north-south and east-west, respectively, including a dedicated bicycle/pedestrian bridge over a busy freeway. These trails connect residential neighborhoods to employment centers downtown and at the University of Minnesota." - *Description of additional intervention components*: NR - *Duration of intervention*: "These vital trail system components were constructed between 2000 and 2007" -7 years - *How was the intervention implemented?*: "...constructed between 2000 and 2007" - *Co-interventions*: Addition of other cycling infrastructure ("...on-road bicycle lanes") - *Where was the intervention implemented?*: See Figure 2; "transecting the city north-south and east-west" - *Resource requirements to replicate the intervention*: Residential areas, workplaces, staff, bicycle commuters, infrastructure investment to build the new off-trail systems, 116 trails - *Economic indicators*: NR   **Control**: Probable non-exposure to the intervention |
| **Identification** | **Sponsorship source:** "National Heart, Lung, and Blood Institute (NHLBI) R01-HL114091 and R01HL104580 and by the National Institute on Child Health and Development (NICHD) T32-HD007168"  **Country:** United States  **Setting:** City  **Comments:**  **Author’s name:** Jana A. Hirsch  **Institution:**  **Email:** hijana@mailbox.sc.edu; ktmeyer@emaill.unc.edu  **Address:**  **Declaration of interests stated?:** "NIH had no role in the design or conduct of the study; collection, management, analysis, or interpretation of the data; or preparation, review, or approval of the manuscript for submission." "For general support, the authors are grateful to the Carolina Population Center, University of North Carolina at Chapel Hill (P2C-HD050924 from the NICHD), the Nutrition Obesity Research Center (NORC), University of North Carolina (P30-DK56350 from the National Institute for Diabetes and Digestive and Kidney Diseases [NIDDK]), and the UNC Center for Environmental Health and Susceptibility (CEHS), University of North Carolina (P30-ES010126 from the National Institute of Environmental Health Sciences [NIEHS])."  **Specify any documents relevant to this study:** Additional file 1: Table S1.Sensitivity analysis of estimated differences in bicycle commuting (%) in 2010 vs 2000 by trail access and potential use. Table S2. Multivariable-adjusted regression estimates for the difference in the percentage of workers commuting by bicycle in 2000 and 2010 according to joint levels of the distance between the track and the trail system and proportion of commuting trips that cross the trail system. (DOCX 21 kb)"  **Study or programme name or acronym:** N/A  **Type of record:** Journal article |
| **Notes** |  |

## Risk-of-bias table

| **Bias** | **Authors' judgement** | **Support for judgement** |
| --- | --- | --- |
| Random sequence generation (selection bias) | High risk | CBA study; additionally, the 'group' comparison we are utilizing is not explicitly compared in the study. |
| Allocation concealment (selection bias) | High risk | CBA study; distance quartiles used to create areas for which exposure to the intervention is more and less likely. |
| Baseline characteristics similar | Unclear risk | Cannot be assessed based on the data reported in this study. |
| Baseline outcome measurements similar | Unclear risk | Cannot be assessed based on the data reported in this study. |
| Blinding of participants and personnel (performance bias) | Low risk | Blinding of the intervention itself in this case is impossible. Change in behavior based on awareness of the intervention is the hypothesized aim, thus a lack of blinding in this regard would not represent a risk of bias. |
| Blinding of outcome assessment (detection bias) | Unclear risk | Outcome data on commuting collected as part of the census through self-reporting, completely independent from the construction of the trail system. However, sensitization to cycling because of the proximity to the trails or the construction may have influenced how people responded to questions about commuting. |
| Protection against contamination | High risk | Judgement Comment: Distance from trails used to represent more and less likely exposure to the intervention. Likely that there is some contamination using this definition. |
| Incomplete outcome data (attrition bias) | Low risk | Data before the intervention was harmonised with data after the intervention. Census data used; source is likely to be very complete. Authors do not describe any data as being missing. |
| Selective reporting (reporting bias) | Low risk | No indication of any selective reporting. |
| Other bias | Low risk | Judgement Comment: No other obvious sources of bias. |

# Hong 2016

| **Methods** | **Study design:** CBA study  **Study grouping:** Parallel  **How was missing data handled?:** Subjects with partial data were excluded from the models.  **How were participants recruited and sampled within the study sites?:** The study used two waves of longitudinal samples from the Expo Line study, which were reported elsewhere. Invitation letters were sent to all households (n = 27,275) in the study area based on addresses purchased from a commercial database provider. All households with an interest in participating were selected into the study."  **Methods for selecting intervention and control sites:** "The treatment group is defined as residents living within ½ mile of a new Expo Line station; and the control group as residents living farther away from a station."  **Number of control sites:** Two (mobile sample and survey sample)  **Number of intervention sites:** Two (mobile sample and survey sample)  **Post-intervention period (start and end date or year it was conducted):** March 2012 - January 2013  **Pre-intervention time period (before intervention start):** "The survey was conducted five to seven months before (Baseline, September 2011 – February 2012)."  **Sample size justification and outcome used:** NR  **Study aim or objective:** To investigate the before-and-after impact of a new light rail transit line on active travel behavior.  **Time points of data collection:** Time 1 (baseline or ‘‘before opening” survey); Time 2 (follow-up or ‘‘after opening” survey).  **Unit of allocation or exposure:** Households |
| --- | --- |
| **Participants** | **Baseline Characteristics**  Intervention -The treatment group was defined as residents living within 1⁄2 mile of a new Expo Line station   - *Age*: Survey sample, Mean (SD) was 49.68 (14.49), and mobile sample was 51.38 (14.39) - *Place of residence*: NR - *Sex*: Survey sample - 79% were female and 21% were male, mobile sample - 72% were female and 28% were male - *Ethnicity and language*: Survey sample in % - White=27, Black=45, Asian=13, Hispanic=9, and other=6, for mobile sample in % - White=28 Black=53, Asian=9, Hispanic=6, and other=3 - *Occupation*: %: Survey Sample: employed 63% Mobile Sample: employed59% - *Education*: Survey sample in % <12th grade=7, High school=0, Some college=27, Associate=16, Bachelor=26, Post graduate=24, for mobile sample in % <12th grade=6, High school=0 Some college=26, Associate=16, Bachelor=26, Post graduate=26 - *Socioeconomic status*: Household income in %, survey sample - <15K=17, 15–35K=26, 35–55K=20, 55–75K=12, 75–100 K=12 , >100K=12, for mobile sample - <15K=16, 15–35K=31, 35–55K=9, 55–75K=9, 75–100 K=19 , >100K=16 - *Social capital*: NR   Control - The control group was defined as residents living farther away > 1/2 mile from a station.   - *Age*: Survey sample, Mean (SD) was 49.67 (14.37), and mobile sample was 53.20 (13.44) - *Place of residence*: NR - *Sex*: Survey sample - 70% were female and 30% were male, mobile sample - 61% were female and 39% were male - *Ethnicity and language*: Survey sample in % - White=28, Black=53, Asian=11, Hispanic=5, other=3, for mobile sample in % - White=29, Black=56, Asian=2, Hispanic=7, other=5 - *Occupation*: %: Survey Sample: employed 61% Mobile Sample: employed 56% - *Education*: Survey sample in % <12th grade=4, High school=6, Some college=23, Associate=10, Bachelor=34, Post graduate=24, for mobile sample in % <12th grade=5, High school=7, Some college=29, Associate=12, Bachelor=34, Post graduate=12 - *Socioeconomic status*: Household income in %, survey sample - <15K=15, 15–35K=25, 35–55K=19, 55–75K=18, 75–100 K=11 , >100K=14,for mobile sample - <15K=15, 15–35K=29, 35–55K=10, 55–75K=15, 75–100 K=15 , >100K=17 - *Social capital*: NR   Overall   - *Age*: NR - *Place of residence*: NR - *Sex*: NR - *Ethnicity and language*: NR - *Occupation*: NR - *Education*: NR - *Socioeconomic status*: NR - *Social capital*: NR   **Included criteria:** "All households with an interest in participating were selected into the study."  **Excluded criteria:** NR  **Pretreatment:** The treatment and control groups were similar in terms of demographic characteristics.  **Attrition per relevant group:** Survey sample: 26.9% (75/279); mobile sample: 48.9% (70/143): "A total of 279 households responded indicating one household adult was willing to participate in a demographic survey and a 7-day travel survey (survey sample), and 204 of the initial sample participated again in the post-opening follow-up survey. 143 subjects indicated that they were willing to participate in a more detailed survey involving accelerometer and GPS devices (mobile sample). After going through a set of filtering processes, the total size of the mobile sample came down to 73 subjects who participated in both pre- and post-opening surveys.  **How were baseline differences assessed?:** The significance of the baseline differences between the treatment and control groups for the survey and mobile samples was determined by t-tests for continuous variables and Fisher tests for categorical variables  **Total number completed and analysed per relevant group:** Survey sample: n=204; n=101 in the treatment group and n=103 in the control group. Mobile sample: n=73; n=32 participants in the treatment group and n=41 in the control group  **Total number in each relevant group pre-intervention:** Survey sample: n=204; n=101 in the treatment group and n=103 in the control group. Mobile sample: n=73; n=32 participants in the treatment group and n=41 in the control group  **Total number randomised per relevant group:** N/A |
| **Interventions** | **Intervention Characteristics**  Intervention -The treatment group was defined as residents living within 1⁄2 mile of a new Expo Line station   - *Type of intervention (e.g. infrastructure, policy or both)*: Infrastructure - *Description of main intervention*: Living closer to a new light rail line. Only phase I construction included in this paper. - *Description of additional intervention components*: NR - *Duration of intervention*: Phase I construction of the Expo line opened in stages in April and June 2012 - *How was the intervention implemented?*: NR - *Co-interventions*: None reported - *Where was the intervention implemented?*: The study neighborhood is located along the Expo light rail line (Expo Line) in south Los Angeles. This new light rail line extends south and west from downtown Los Angeles, eventually reaching downtown Santa Monica - *Resource requirements to replicate the intervention*: NR - *Economic indicators*: NR   Control - The control group was defined as residents living farther away > 1/2 mile from a station.   - *Type of intervention (e.g. infrastructure, policy or both)*: Infrastructure - *Description of main intervention*: Living farther away from new railway line (<1/2 mile from a station) - *Description of additional intervention components*: None reported - *Duration of intervention*: Phase I construction of the Expo line opened in stages in April and June 2012 - *How was the intervention implemented?*: NR - *Co-interventions*: None reported - *Where was the intervention implemented?*: The study neighborhood is located along the Expo light rail line (Expo Line) in south Los Angeles. This new light rail line extends south and west from downtown Los Angeles, eventually reaching downtown Santa Monica - *Resource requirements to replicate the intervention*: NR - *Economic indicators*: NR |
| **Identification** | **Sponsorship source:** "The research was financially supported by the California Air Resources Board; the John Randolph and Dora Haynes Foundation; the Lincoln Institute of Land Policy; the Mineta Transportation Institute, San Jose State University; the Southern California Association of Governments; the University of California Multi-Campus Research Program on Sustainable Transportation; the University of California Transportation Center; and the University of Southern California Lusk Center for Real Estate."  **Country:** USA  **Setting:** The study was conducted in the neighbourhood located along the Expo light rail line in south Los Angeles.  **Comments:**  **Author’s name:** Andy Hong  **Institution:**  **Email:** andyhong@gmail.com ; boarnet@usc.edu ; houston@uci.edu  **Address:**  **Declaration of interests stated?:** NR  **Specify any documents relevant to this study:** n/a  **Study or programme name or acronym:** Expo Line study  **Type of record:** Journal article |
| **Notes** |  |

## Risk-of-bias table

| **Bias** | **Authors' judgement** | **Support for judgement** |
| --- | --- | --- |
| Random sequence generation (selection bias) | High risk | Controlled before and after study. |
| Allocation concealment (selection bias) | High risk | CBA study. |
| Baseline characteristics similar | Low risk | Quote: "However, none of these differences between treatment group and control group are statistically signiﬁcant, implying that the two groups are similar in terms of demographic characteristics."  baseline characteristics between the treatment and the control group were similar. |
| Baseline outcome measurements similar | Low risk | The longitudinal analysis was performed using an ANCOVA (analysis of covariance) framework, wherein the dependent variable was regressed on past behavior, the treatment condition (proximity to transit), and an interaction between the past behavior and the treatment condition. The form of the regression is shown in Eq. (1). |
| Blinding of participants and personnel (performance bias) | Low risk | Blinding was not possible but it is unlikely to influence participant behavior. |
| Blinding of outcome assessment (detection bias) | High risk | Some outcomes are self-reported through surveys and may be more susceptible to lack of blinding. |
| Protection against contamination | Unclear risk | Contamination could occur given that distance from the station is what distinguishes the intervention from the comparison group. |
| Incomplete outcome data (attrition bias) | High risk | There was a high rate of incomplete outcome data. |
| Selective reporting (reporting bias) | Low risk | no evidence that outcomes were selectively reported. |
| Other bias | Low risk | No other sources of bias observed. |

# Jung 2017

| **Methods** | **Study design:** CBA  **Study grouping:**  **How was missing data handled?:** Numbers of people surveyed before and after implementation of intervention were not reported.  **How were participants recruited and sampled within the study sites?:** Convenience sampling. Government selected the survey locations from among Seoul’s major activity centers, transportation nodes, and major land-use areas. The survey was used to collect pedestrians’ personal characteristics, their travel purpose and frequency, and levels of pedestrian satisfaction. Considering the time needed for surveyors to select respondents and for respondents to answer all the survey questions, each surveyor was asked to collect 12 responses in the morning (6 h) and 12 responses in the afternoon (6 h), yielding 24 responses per day. Each surveyor selected respondents who were passing by each location every 30 min. The surveyors gathered 72 responses at each survey location, conducting surveys with 24 pedestrians on each of the following days: Tuesday, Wednesday, and Friday. Auditors tallied pedestrian numbers at the locations for a total of five days per location, on Monday, Tuesday, Wednesday, Friday, and Saturday, between 7:30 a.m. and 8:30 p.m.  **Methods for selecting intervention and control sites:** "Among the 1000 survey locations, 28 are included in Design Street projects, and these locations constitute the treatment group. To build the control group, we selected 218 matching locations from typical streets in the administrative areas (called‘‘dongs” in Korea) that the streets of the Design Street projects run through or face."  **Number of control sites:** 218 streets  **Number of intervention sites:** 28 streets  **Post-intervention period (start and end date or year it was conducted):** "The first Design Street project was completed in October 2009, and a total of 23 projects were implemented by 2010". Interventions as infrastructure changes therefore they remain available indefinitely.  **Pre-intervention time period (before intervention start):** August to October 2009  **Sample size justification and outcome used:** NR  **Study aim or objective:** "to investigate pedestrians’ mode choice and experience by assessing the influence of physical street improvement on pedestrian volume and satisfaction" and it "examines the influence of the Design Street projects on walking behavior".  **Time points of data collection:** baseline: August to October 2009 and follow up: October to November 2012  **Unit of allocation or exposure:** Streets |
| --- | --- |
| **Participants** | **Baseline Characteristics**  Intervention   - *Age*: NR - *Place of residence*: Urban area; Seoul city - *Sex*: NR - *Ethnicity and language*: NR - *Occupation*: NR - *Education*: NR - *Socioeconomic status*: NR - *Social capital*: NR   Control   - *Age*: NR - *Place of residence*: Urban area; Seoul city - *Sex*: NR - *Ethnicity and language*: NR - *Occupation*: NR - *Education*: NR - *Socioeconomic status*: NR - *Social capital*: NR   Overall   - *Age*: NR - *Place of residence*: Urban area; Seoul city - *Sex*: NR - *Ethnicity and language*: NR - *Occupation*: Employment density, mean (SD): 0.163 (0.190) - *Education*: NR - *Socioeconomic status*: Population density, mean (SD): 0.422 (0.218). - *Social capital*: NR   **Included criteria:** "The government selected the survey locations from amongst Seoul’s major activity centers, transportation nodes, and major land-use areas." None other reported.  **Excluded criteria:** None reported.  **Pretreatment:** Analysis of baseline differences not reported but Table 1 shows differences between intervention and control streets in pedestrian count, number of lanes for vehicles, number of bus-dedicated and pedestrian-dedicated lanes, and other variables.  **Attrition per relevant group:** No survey response rates reported.  **How were baseline differences assessed?:** NR  **Total number completed and analysed per relevant group:** NR  **Total number in each relevant group pre-intervention:** Design streets: 2016 responses (28 streets x 72 responses/location) Control street: 15,696 responses (218 streets x 72 responses/location)  **Total number randomised per relevant group:** N/A |
| **Interventions** | **Intervention Characteristics**  Intervention   - *Type of intervention (e.g. infrastructure, policy or both)*: Infrastructure - *Description of main intervention*: "The Seoul Metropolitan Government [...] initiated three periods of the Design Street Project in 30 target areas from 2007 with the aim of creating pedestrian-friendly streets." "The Design Street projects include the improvement of sidewalks, public spaces, signs, fences, and other physical elements of the streets." These included cleanliness, safety, scenery, convenience. - *Description of additional intervention components*: Coordinated planning of city-wide activities involving master- planner, city development officials. - *Duration of intervention*: First design street project completed in October 2009 and still remain available. - *How was the intervention implemented?*: "In 2007, the Seoul Metropolitan Government planned the Design Street Project with the aim of encouraging walking and social activities on streets by improving physical street environments. They selected major pedestrian corridors of activity for these projects, and a master planner, cooperating with public officials, stakeholders, citizens, and professional planners, planned a comprehensive retrofit of the street-environment elements to reverse the ongoing deterioration of these public spaces. The Design Street projects include the improvement of sidewalks, public spaces, signs, fences, and other physical elements of the streets." These included cleanliness, safety, scenery, and convenience. - *Co-interventions*: None reported - *Where was the intervention implemented?*: Seoul’s major activity centers, transportation nodes, and major land-use areas - *Resource requirements to replicate the intervention*: 1. "...a master planner, cooperating with public officials, stakeholders, citizens, and professional planners, planned a comprehensive retrofit of the street-environment elements to reverse the ongoing deterioration of these public spaces". - *Economic indicators*: NR   Control: No intervention |
| **Identification** | **Sponsorship source:** "This research was supported by the Basic Science Research Program through the National Research Foundation of Korea (NRF), funded by the Ministry of Science, ICT & Future Planning (2013R1A1A1057772). This work was also supported by the 2015 Hongik University Research Fund."  **Country:** Korea  **Setting:** Urban streets  **Author’s name:** Jae Seung Lee  **Email:** jaeseung74@gmail.com  **Declaration of interests stated?:** NR  **Specify any documents relevant to this study:** N/A  **Study or programme name or acronym:** Design Street Project  **Type of record:** Journal article |
| **Notes** |  |

## Risk-of-bias table

| **Bias** | **Authors' judgement** | **Support for judgement** |
| --- | --- | --- |
| Random sequence generation (selection bias) | High risk | This was a CBA therefore there was no randomisation. |
| Allocation concealment (selection bias) | High risk | This was a CBA therefore there was no randomisation. |
| Baseline characteristics similar | Unclear risk | Characteristics of participants not reported. |
| Baseline outcome measurements similar | Low risk | Baseline pedestrian count and volume were higher in the treatment/design street arm, however, this was adjusted for in the analysis (DID). |
| Blinding of participants and personnel (performance bias) | Low risk | Blinding was not possible but this is unlikely to affect behavior of participants. |
| Blinding of outcome assessment (detection bias) | High risk | Blinding was not possible. Some outcomes were based on self reports and these may have been influenced by knowledge of which streets were not part of the intervention. |
| Protection against contamination | High risk | Intervention and control streets were close and people from control streets could access the intervention streets. |
| Incomplete outcome data (attrition bias) | Unclear risk | Number of respondents at baseline and endline is unclear, as is the number analysed. |
| Selective reporting (reporting bias) | Low risk | All relevant outcomes in the methods section are reported on in the results section; pedestrian satisfaction, pedestrian volumes and pedestrian counts. |
| Other bias | Low risk | Misclassification bias of the exposure - unlikely. Pedestrians interviewed at design/control streets. Measurement bias - unlikely. |

# Kubota 2019

| **Methods** | **Study design:** CBA  **Study grouping:** Parallel group  **How was missing data handled?:** Participants with missing data in outcomes and demographic variables were excluded.  **How were participants recruited and sampled within the study sites?:** Random sampling in each site, with a postal invitation to take part in the study, both at baseline and follow-up. "In March 2013, 3200 adult residents (aged 30–74 years) were randomly selected in each site from the registry of residential addresses. Individuals were chosen from lists of residents that were classified by age (30–39, 40–49, 50–59, and 60–74 years) and gender. This age group (30–74 years) was chosen as they are known to have a greater risk of developing chronic diseases [18]. Individuals who were selected received a postal invitation to take part in this study and those who agreed to participate received a survey questionnaire. There was no incentive to participate in this study." "In January 2015, participants were randomly recruited again."  **Methods for selecting intervention and control sites:** Choice of intervention site is not explicitly explained but it is assumed it is due to it being the site where the new exercise facility was being built. "The control site was chosen to obtain data from a locality where the population size, geography, and climate were comparable to those of the intervention site and where no major exercise facility development or PA promotion activities were expected to take place during the study period."  **Number of control sites:** 1 ("The control site was Oiso in an adjacent prefecture (Population: 32,625; percentage of population aged≥65 years: 29%, as of April 2013)").  **Number of intervention sites:** 1 ("The intervention site was Nagaizumi, Shizuoka Prefecture (Population: 41,912; percentage of population aged ≥65 years: 19%, as of April 2013)").  **Post-intervention period (start and end date or year it was conducted):** August 2013 (when new exercise facility was opened) - present. Follow up in February 2015.  **Pre-intervention time period (before intervention start):** April to August 2013  **Sample size justification and outcome used:** NR  **Study aim or objective:** This study examined the impact of a newly constructed multipurpose exercise facility on community-level PA, perceived availability of PA facilities, awareness of others being active, and willingness to engage in PA in Japan.  **Time points of data collection:** 2013 and 2015  **Unit of allocation or exposure:** Prefectures. |
| --- | --- |
| **Participants** | **Baseline Characteristics**  Intervention   - *Age : Mean (SD)*: 52.9 (12.5) - *Place of residence*: NR - *Sex :% men*: 40.9 - *Ethnicity and language*: NR - *Occupation*: Employment status, % working: 65.4 - *Education: %>12 years*: Education level, % > 12 years: 51.8 - *Socioeconomic status : % working*: NR - *Social capital*: Marital status, % single: 7.6   Control   - *Age : Mean (SD)*: Age in year, mean(SD): 53.1 (12.5) - *Place of residence*: NR - *Sex :% men*: Gender, % men: 42.0 - *Ethnicity and language*: NR - *Occupation*: Employment status, % working: 58.9 - *Education: %>12 years*: Education level, % > 12 years: 65.1 - *Socioeconomic status : % working*: NR - *Social capital*: Marital status, % single: 5.9   Overall   - *Age : Mean (SD)*: NR - *Place of residence*: NR - *Sex :% men*: NR - *Ethnicity and language*: NR - *Occupation*: NR - *Education: %>12 years*: NR - *Socioeconomic status : % working*: NR - *Social capital*: NR   **Included criteria:** "Individuals were chosen from lists of residents that were classified by age (30–39, 40–49, 50–59, and 60–74 years)". No other criteria reported.  **Excluded criteria:**  **Pretreatment:**  **Attrition per relevant group:**  **How were baseline differences assessed?:**  **Total number completed and analysed per relevant group:** Baseline (2013): 821 participants in the intervention site and 845 in the control site. Follow-up (2015): 1018 for the intervention site and 924 for the control site at follow-up.  **Total number in each relevant group pre-intervention:** control site: 1125 intervention: 1107  **Total number randomised per relevant group:** NA |
| **Interventions** | **Intervention Characteristics**  Intervention   - *Type of intervention (e.g. infrastructure, policy or both)*: - *Description of main intervention*: "...construction of a new multipurpose exercise facility, the Nagaizumi Health Promotion Center (NHPC). The NHPC, which opened in August 2013, includes indoor facilities (25 m pool, 170 m walking trail, multi-purpose gym, and group exercise rooms) and outdoor facilities (multi-purpose athletic field, 875 walking trail, and park). All residents of Nagaizumi could use facilities in the NHPC for a small fee (e.g., the use of multi-purpose gym: US$1." - *Description of additional intervention components*: - *Duration of intervention*: August 2013 to February 2015 - *How was the intervention implemented?*: All residents of the town were invited to take part in the program. Entrants self-reported whether they met various goals specified in the program, such as “did exercise/sports”, “used stairs rather than an elevator”, and “walked/cycled instead of driving”. A point (an official stamp) was given to each goal achieved at town facilities including the NHPC. The points that residents earned through this scheme could be used to pay for entrance to the NHPC or to receive discounts at local grocery stores. - *Co-interventions*: - *Where was the intervention implemented?*: In the Nagaizumi prefecture, in the new exercise facility and surrounding community - *Resource requirements to replicate the intervention*: NR - *Economic indicators*:   Control   - *Type of intervention (e.g. infrastructure, policy or both)*: - *Description of main intervention*: "No special PA promotion program was implemented in the control (Oiso) site during the study period. The NHPC is not easily accessible from Oiso. However, the health promotion division of Oiso provided routine promotion activities including health education classes and health promotion events." - *Description of additional intervention components*: n/a - *Duration of intervention*: n/a - *How was the intervention implemented?*: n/a - *Co-interventions*: n/a - *Where was the intervention implemented?*: n/a - *Resource requirements to replicate the intervention*: n/a - *Economic indicators*: n/a |
| **Identification** | **Sponsorship source:** This study was funded by the Japan Society for the Promotion of Science (Grants-in-Aid for Scientific Research, # JP24300232). The manuscript was written when the lead author was visiting Australian Catholic University, using the financial support from Tokai University. The funders had no role in study design, data collection and analysis, decision to publish, or preparation of the manuscript.  **Country:** Japan  **Setting:** Prefectures in regional city in Japan  **Comments:** this study was conducted in two different sites: intervention and control sites  **Author’s name:** Akio Kubota  **Institution:**  **Email:** ka092000@tsc.u-tokai.ac.jp  **Address:**  **Declaration of interests stated?:** The authors declare that they have no competing interests.  **Specify any documents relevant to this study:**  **Study or programme name or acronym:** NA  **Type of record:** Journal article |
| **Notes** |  |

## Risk-of-bias table

| **Bias** | **Authors' judgement** | **Support for judgement** |
| --- | --- | --- |
| Random sequence generation (selection bias) | High risk | This was a CBA and no randomisation was carried out. |
| Allocation concealment | High risk | CBA study |
| Baseline characteristics similar | Low risk | There are some baseline differences but these were adjusted for in the analyses. |
| Baseline outcome measurements similar | High risk | % of those who perceived availability of PA facilities is higher in the intervention site. All other outcomes are similar at baseline. Analyses were not adjusted for any differences in outcomes. |
| Blinding of participants and personnel (performance bias) | Low risk | Blinding is not possible in these types of interventions. However, lack of blinding is unlikely to change the intervention delivered. |
| Blinding of outcome assessment (detection bias) | High risk | Outcome was a self reported information by a participant. No information regarding whether they were or not blinded. |
| Protection against contamination | Low risk | The two sites were very distant (50Km) therefore control sites were unlikely to be affected by activities from intervention sites. |
| Incomplete outcome data (attrition bias) | High risk | Response rate to the postal surveys was very low (aprox 35% in each group). In addition, further participants were excluded from the analysis due to missing data; the percentage excluded was similar in the intervention and control groups at baseline (27% and 42% respectively) but it was higher in the control group at follow-up (9% vs 24%). |
| Selective reporting (reporting bias) | Low risk | All expected outcomes as mentioned in the method were reported. |
| Other bias | High risk | Misclassification bias of exposure: low risk; postal survey sent by researchers to participant's houses. Measurement bias: high risk; self-reported measures through postal survey at high risk of recall and desirability bias. Incorrect analysis: n/a |

# McDonald 2013

| **Methods** | **Study design:** CBA  **Study grouping:** Parallel group  **How was missing data handled?:** NR  **How were participants recruited and sampled within the study sites?:** Most data was collected through classroom surveys. Data on 'school trip travel mode' collected through surveys:- National Center for Safe Routes to School's validated student tally sheet (teachers/ volunteers complete this via hand raising in class) - this is collected on Tues, Weds, Thurs.- National Center for Safe Routes to Schools validated parent survey asking 'on most days, how does your child arrive and leave school?'- specialized survey developed by University of Oregon.  **Methods for selecting intervention and control sites:** Intervention sites had Safe Routes to School interventions and control schools did not. Information on the nature and timing of SRTS interventions at each school was collected through interviews with school personnel involved in the program and ground truthing. Details shown in Table 2  **Number of control sites:** Schools with no STRS programs n = 5  **Number of intervention sites:** Schools with STRS programs n = 9  **Post-intervention period (start and end date or year it was conducted):** "The SRTS program became fully operational in the 4J School District in the fall of 2007...". Data collection continued until fall 2011.  **Pre-intervention time period (before intervention start):** Pre-fall 2007  **Sample size justification and outcome used:** NR  **Study aim or objective:** "Our study uses a quasi-experimental research design to evaluate the impacts of the SRTS program in Eugene, Oregon on walking and biking to and from school."  **Time points of data collection:** Each fall and spring, a subset of schools was surveyed by district SRTS staff. The University of Oregon survey data were collected in spring 2008 and 2010 to supplement information collected by the school district.Each school year, data were collected on 1000–2300 students or 16–42% of students attending the study schools. All data sets were aggregated to compute the proportion of students who walked and biked by school, grade, and survey week.  **Unit of allocation or exposure:** Schools |
| --- | --- |
| **Participants** | **Baseline Characteristics**  Intervention   - *Age*: NR - *Place of residence*: NR - *Sex*: NR - *Ethnicity and language*: % American-Indian: 1.2; Asian/Pacific islander: 3.8; Black: 2.8; Hispanic: 14.3; Mutli-racial: 8.5; White: 69.4 - *Occupation*: School students - *Education*: Achievement index: 89.5 - *Socioeconomic status*: % Free or reduced price lunch: 48.2 - *Social capital*: NR   Control   - *Age*: NR - *Place of residence*: NR - *Sex*: NR - *Ethnicity and language*: % American-Indian: 1.5 Asian/Pacific islander: 2.7; Black: 3.1; Hispanic: 15.6; Multi-racial: 8.5; White: 68.5 - *Occupation*: School students - *Education*: Achievement index: 89.8 - *Socioeconomic status*: % Free or reduced price lunch: 44.8 - *Social capital*: NR   Overall   - *Age*: NR - *Place of residence*: NR - *Sex*: NR - *Ethnicity and language*: See table 2 - *Occupation*: School students - *Education*: 0.3 (p=0.999) - *Socioeconomic status*: Free or reduced price lunch: -3.4 difference (p=0.462) - *Social capital*: NR   **Included criteria:** NR  **Excluded criteria:** NR  **Pretreatment:** Reported in Table 2: No differences in demographics or built environment between control and intervention schools except for 'walkscore' which differed significantly - intervention schools had significantly higher walkscore which relies heavily on the proximity of destinations such as stores and parks and likely reflects that treatment schools are located closer to retail establishments.  **Attrition per relevant group:** NR  **How were baseline differences assessed?:** See Table 2: differences were assessed using a Mann Whitney test. They assessed: school demographics, ethnic and socioeconomic backgrounds, achievement levels and school built environment. assessed from School Demographics: National Center for Education Statistics, Oregon Department of Education; School Built Environment: ESRI, WalkScore  **Total number completed and analysed per relevant group:** 9 schools in intervention and 5 schools in control - individual level numbers not provided.  **Total number in each relevant group pre-intervention:** NR  **Total number randomised per relevant group:** N/A |
| **Interventions** | **Intervention Characteristics**  Intervention   - *Type of intervention (e.g. infrastructure, policy or both)*: Both - *Description of main intervention*: "Interventions are broadly classified into 4 categories, also known as the 4E's: Engineering interventions are usually infrastructure improvements, such as side-walk construction, crosswalks, and traffic signal improvements. Education programs aim to improve students' active commuting skills and awareness, as well as increase the safety of walking and biking activities. Similarly, encouragement interventions seek to raise the awareness of active commuting benefits among both students and their parents. “Walk and Bike to School Day” is one “example of a program designed to increase enthusiasm for active commuting”. The fourth “E” enforcement includes intervention measures such as the funding of street crossing guards, the placement of speed feedback trailers near schools, and increased police presence to enforce speed limits in school zones." - *Description of additional intervention components*: NR - *Duration of intervention*: From fall 2007 different interventions introduced over the period until 2011 when the data collection stopped for this study - *How was the intervention implemented?*: A SRTS program manager was appointed. The program funds and administers a variety of interventions aimed at increasing walking or biking to and from school by K-8 students. Different interventions at different schools. - *Co-interventions*: NR. The SRTS programme consisted of multiple interventions and were analysed accordingly. - *Where was the intervention implemented?*: At 9 schools within the 4J School District (elementary and middle schools). - *Resource requirements to replicate the intervention*: NR - *Economic indicators*: "Over 1.1. billion has been authorized for the program..."   Control   - *Type of intervention (e.g. infrastructure, policy or both)*: No intervention - *Description of main intervention*: No intervention - *Description of additional intervention components*: No intervention - *Duration of intervention*: No intervention - *How was the intervention implemented?*: No intervention - *Co-interventions*: NR - *Where was the intervention implemented?*: 5 control schools - *Resource requirements to replicate the intervention*: NR - *Economic indicators*: No intervention |
| **Identification** | **Sponsorship source:** This research was funded by a grant from the Active Living Research program of the Robert Wood Johnson Foundation.  **Country:** U.S.A  **Setting:** Eugene, Oregon, School based programme  **Comments:** NR/A  **Author’s name:** N.C.McDonald  **Institution:** Department of City and Regional Planning, University of North Carolina at Chapel Hill  **Email:** noreen@unc.edu  **Address:** Chapel Hill, North Carolina, USA  **Declaration of interests stated?:** NR  **Specify any documents relevant to this study:** N/A  **Study or programme name or acronym:** Safe Routes to School (SRTS) program  **Type of record:** Journal article |
| **Notes** |  |

## Risk-of-bias table

| **Bias** | **Authors' judgement** | **Support for judgement** |
| --- | --- | --- |
| Random sequence generation (selection bias) | High risk | CBA study - no sequence for randomisation generated. |
| Allocation concealment (selection bias) | High risk | CBA - no allocation concealment. |
| Baseline characteristics similar | Unclear risk | Most criteria were similar, except 'walkscore' which was higher in the intervention arm, which may imply that the schools receiving the intervention already had higher propensity to walking or better access to parks or retail. It is not clear whether this would result in high risk of bias. |
| Baseline outcome measurements similar | Unclear risk | Some outcome measurements such as ethnicity, school built environment and achievement index were similar at baseline (Table 2). However, main outcomes for walking and biking were not reported at baseline |
| Blinding of participants and personnel (performance bias) | Low risk | No blinding possible with this CBA study design, however, it is unlikely that the outcome was influenced by lack of blinding. |
| Blinding of outcome assessment (detection bias) | Unclear risk | Not clear if outcome assessors were blinded to the groups they were analysing (e.g. statistician). |
| Protection against contamination | Low risk |  |
| Incomplete outcome data (attrition bias) | Unclear risk | No report of attrition. Analysis done at school level and accounted for differential characteristics by statistical techniques. |
| Selective reporting (reporting bias) | Low risk | All relevant outcomes in the methods section are reported in the results section. |
| Other bias | Low risk | No misclassification or measurement bias. |

# Pazin 2016

| **Methods** | **Study design:** CBA  **Study grouping:** Parallel group  **How was missing data handled?:** missing were imputed using last observation carried forward strategy  **How were participants recruited and sampled within the study sites?:**  **Methods for selecting intervention and control sites:**  **Number of control sites:**  **Number of intervention sites:**  **Post-intervention period (start and end date or year it was conducted):** July 2009 - December 2012  **Pre-intervention time period (before intervention start):** Between March and July in 2009  **Sample size justification and outcome used:** sample size was calculated using the following parameters: 75% of adults not practicing at least 150 min/week of leisure-time PA, confidence interval of 95% and 3.3 percentage points of sampling error. The required sample size (656) was increased by 10% to account for losses and refusals, and then by 15% to allow controlling for confounding factors in multivariable analysis. Therefore, the required sample size was 820.  **Study aim or objective:** The primary aim of this study was to evaluate the effects of a new walking and cycling route on leisure time physical activity (PA) (walking and moderate-to-vigorous PA) of adults.  **Time points of data collection:** From March and July in 2009. At the baseline and March and December in 2012 at follow-up.  **Unit of allocation or exposure:** n/a |
| --- | --- |
| **Participants** | **Baseline Characteristics**  Intervention ( 0–500)   - *Age*: 18- 34 ( n = 31); 35-54 (n=79 ); 55-85 ( n=82 ) - *Place of residence*: NR - *Sex*: Male: 87, Female: 105 - *Ethnicity and language*: White ( n =154 ), Black ( n = 6), Brown (n=32 ) - *Occupation*: - *Education*: middle school ( n= 21 ); High school ( n = 68) ; College or higher ( n = 102) - *Socioeconomic status*: NR - *Social capital*: NR   Intervention (501–1000)   - *Age*: 8-34: 28, 35-54: 46, 55-85: 63 - *Place of residence*: NR - *Sex*: Male: 63,Female: 74 - *Ethnicity and language*: White (n =105 ), Black (n =7), Brown (n=25 ) - *Occupation*: NR - *Education*: middle school ( n = 20); High school (n = 49) ; College or higher (n = 68) - *Socioeconomic status*: NR - *Social capital*: NR   Intervention (1001–1500)   - *Age*: 18-34 (n=40); 35-54 (n=80 ); 55-85 (n=70) - *Place of residence*: NR - *Sex*: Male (n=67); Females (n=123 ) - *Ethnicity and language*: White (n=138), Black (n=9), Brown (n=43) - *Occupation*: NR - *Education*: middle school (n= 47); High school (n =59); College or higher (n=84) - *Socioeconomic status*: NR - *Social capital*: NR   Overall   - *Age*: 18-34 (n=99); 35-54 (n=205); 55-85 (n=215) - *Place of residence*: NR - *Sex*: Male (n=217); Females (n=302) - *Ethnicity and language*: White (n=397), Black (n=22) Brown (n=100) - *Occupation*: NR - *Education*: middle school (n=88); High school (n=176) ; College or higher (n=254) - *Socioeconomic status*: NR - *Social capital*: NR   **Included criteria:** Aged at least 18 years old, residing in the study area.  **Excluded criteria:** Participants were excluded if they met any of the following criteria: non-permanent resident in the house, planning to move from the neighborhood in a one-year period, current pregnancy, and being unable to practice PA due to health issues.  **Pretreatment:** There was a significantly greater percentage of females than males in the distance to new route group (1001m - 1500m) compared to the other two groups who had fairly evenly distributed groups of males compared to females. There was a significantly greater percent of those with middle school level of education in the distance to the new route group (1001m - 1500m) compared to the other two groups.  **Attrition per relevant group:** Attrition rate was around 30%,  **How were baseline differences assessed?:** Telephone-based interviews  **Total number completed and analysed per relevant group:** 519 reassessed at follow up.  **Total number in each relevant group pre-intervention:** 745 at Baseline  **Total number randomised per relevant group:** NA |
| **Interventions** | **Intervention Characteristics**  Intervention: 0–500m   - *Type of intervention (e.g. infrastructure, policy or both)*: Infrastructure - *Description of main intervention*: The project included a newavenue, parking lots, and an on-road walking and cycling route, all along the seashore. Fig. S1 (supplementary material) shows the area where the new facilities were built, called Beira-Mara Continental (2.3 km long). A new project exists to add 8.3 km to the Beira-Mar Continental and to connect it with the main road of the city (BR 101), but this is still being planned. Despite the main goal being to facilitate commuting, the walking and cycling route is mostly used for leisure-related activities at the moment. One reason is the current lack of connection of the route with other walking trails and cycling lanes of the city, hindering its use for commuting. - *Description of additional intervention components*: NR - *Duration of intervention*: 20–29 months after the new route was available to the public - *How was the intervention implemented?*: The new walking and cycling route brought a pleasant and safe place to practice leisure-time PA, alongside the shore. - *Co-interventions*: NR - *Where was the intervention implemented?*: The community in continental coast of Florianópolis, SC, Brazil: Six neighborhoods (Jardim Atlântico, Estreito, Capoeiras, Canto, Coloninha and Balneário) which were within 1500 m from the new route. - *Resource requirements to replicate the intervention*: NR - *Economic indicators*: NR   Intervention: 501–1000m   - *Type of intervention (e.g. infrastructure, policy or both)*: Infrastructure - *Description of main intervention*: The project included a new avenue, parking lots, and an on-road walking and cycling route, all along the seashore. Fig. S1 (supplementary material) shows the area where the new facilities were built, called Beira-Mara Continental (2.3 km long). A new project exists to add 8.3 km to the Beira-Mar Continental and to connect it with the main road of the city (BR 101), but this is still being planned. Despite the main goal being to facilitate commuting, the walking and cycling route is mostly used for leisure-related activities at the moment. One reason is the current lack of connection of the route with other walking trails and cycling lanes of the city, hindering its use for commuting. - *Description of additional intervention components*: NR - *Duration of intervention*: 20–29 months after the new route was available to the public - *How was the intervention implemented?*: The new walking and cycling route brought a pleasant and safe place to practice leisure-time PA, alongside the shore. - *Co-interventions*: NR - *Where was the intervention implemented?*: The community in continental coast of Florianópolis, SC, Brazil: Six neighborhoods (Jardim Atlântico, Estreito, Capoeiras, Canto,Coloninha and Balneário) which were within 1500 m from the new route. - *Resource requirements to replicate the intervention*: NR - *Economic indicators*: NR   Intervention: 1001–1500m   - *Type of intervention (e.g. infrastructure, policy or both)*: Infrastructure - *Description of main intervention*: The project included a new avenue, parking lots, and an on-road walking and cycling route, all along the seashore. - *Description of additional intervention components*: NR - *Duration of intervention*: 20–29 months after the new route was available to the public - *How was the intervention implemented?*: The new walking and cycling route brought a pleasant and safe place to practice leisure-time PA, alongside the shore. - *Co-interventions*: NR - *Where was the intervention implemented?*: The community in continental coast of Florianópolis, SC, Brazil: Six neighborhoods (Jardim Atlântico, Estreito, Capoeiras, Canto, Coloninha and Balneário) which were within 1500 m from the new route - *Resource requirements to replicate the intervention*: NR - *Economic indicators*: NR |
| **Identification** | **Sponsorship source:** The authors declare no funding source related with this study.  **Country:** Brazil  **Setting:** Continental coast of Florianópolis, SC, Brazil,  **Comments:**  **Author’s name:** Joris Pazin  **Institution:** State University of Santa Catarina, Center of Health and Sport Sciences, Rua Pascoal Simone, 358, ZIP code: 88080-350, Florianópolis, SC, Brazil; Federal University of Santa Catarina, Center of Sports, Campus Universitário Reitor João David Ferreira Lima  **Email:** jorispazin0306@gmail.com  **Address:** n/r  **Declaration of interests stated?:** The authors declare no conflict of interests with this study.  **Specify any documents relevant to this study:** Supplementary data associated with this article can be found in the online version athttp://dx.doi.org/10.1016/j.healthplace.2016.02.005.  **Study or programme name or acronym:** N/A  **Type of record:** Journal Article |
| **Notes** |  |

## Risk-of-bias table

| **Bias** | **Authors' judgement** | **Support for judgement** |
| --- | --- | --- |
| Random sequence generation (selection bias) | High risk | Longitudinal quasi-experiment |
| Allocation concealment (selection bias) | High risk | Systematic sampling was used to select households, based on the list of landlines of all streets within the study area at baseline (n¼7630). |
| Baseline characteristics similar | Low risk | See Table 1. Baseline characteristics similar (Score “Low risk” if baseline characteristics of the study and control group are reported and similar. |
| Baseline outcome measurements similar | Low risk | The first adult (aged Z > 18 years) of each selected household who answered the telephone call was invited to participate in the study. |
| Blinding of participants and personnel (performance bias) | Low risk | The blinding was not done but the outcome are less likely to be influenced given the type of study. |
| Blinding of outcome assessment (detection bias) | High risk | Those recording the outcomes based on the telephone call would have their details. Although we do not know the involvement of the interviewer. All we know is that they were trained. |
| Protection against contamination | Low risk | Since the intervention is the same within groups regardless of the distance differences, there is no chance for contamination. |
| Incomplete outcome data (attrition bias) | Low risk | The proportion of individuals who did not complete the follow-up survey was similar among distance groups, varying from 28% to33%. Baseline leisure-time PA, demographic and health-related characteristics among those who did not complete the follow-up survey were similar among distance groups. Attrition rate was around 30%, but similar in all groups and PA was not different between individuals who did and did not complete the follow-up survey. There was no control group unexposed to the new route, although we had three distance groups representing different exposure levels. |
| Selective reporting (reporting bias) | Low risk | They cover all the important outcomes. Although details of the secondary outcomes were not specifically listed here. Likely word count limits. Examples of outcomes not reported are - is like exercising, health problems, prefer other places to practice, dislike the infrastructure, difficult access, lack of company and feeling too tired. |
| Other bias | High risk | Since the outcome is measured based on the reported information, there is a likelihood of recall/reporting bias. |

# Prins 2017

| **Methods** | **Study design:** Controlled before-after study  **Study grouping:** Parallel group  **How was missing data handled?:** Missing data were excluded. "Participants who did not live in one of the study areas at follow-up were excluded from the analyses." "Only participants with full data on all covariates were included in the analyses." "We excluded the travel records of participants who returned a completely blank record, reported not having been at home on the day in question, returned a record so implausible that they appeared to have misunderstood the question or returned non-numeric values (such as ticks) instead of minutes values". "No missing data were imputed."  **How were participants recruited and sampled within the study sites?:** "At both time points, eligible unit postcodes (the smallest unit of postal geography in the UK, corresponding to approximately 15 addresses on average) were identified for each of the three study areas. A random sample of 3000 private residential addresses in each area – 9000 in total – was drawn using the Royal Mail Postcode Address File. A survey pack was posted to each of these households, addressed to the householder. Participants were given the option to return a consent form giving permission to be contacted again in the future. Contact with these participants was maintained via yearly mailings between 2005 and 2012. At follow-up, a further 3000 postal surveys were issued in each study area. The recipients comprised all those baseline participants who could still be contacted, including those who had moved between or out of the study areas but not out of the UK, together with a newly drawn random sample of households to bring the total up to 3000 in each area."  **Methods for selecting intervention and control sites:** "At baseline, three local study areas were defined: the ‘M74 corridor’ intervention area (South) and two control areas, one of which surrounded the existing M8 and M80 motorways (East) and one of which had no comparable major road infrastructure (North) (Figure 4). These study areas were carefully and iteratively delineated at baseline using spatially referenced Census and transport infrastructure data combined with field visits to ensure similar aggregate socioeconomic characteristics and broadly similar topographical and urban morphological characteristics apart from their proximity to urban motorway infrastructure."  **Number of control sites:** Two: one surrounding the existing M8 motorway (East) and a ..., control area with no motorway surrounding a quiet suburban railway (North)  **Number of intervention sites:** One: The ‘M74 corridor’ study area (South)  **Post-intervention period (start and end date or year it was conducted):** 2011-2013 (data collection 2013)  **Pre-intervention time period (before intervention start):** 2005-2011. Baseline assessment conducted in 2005. Motorway construction started in 2008 and ended in 2011.  **Sample size justification and outcome used:** "The study was designed to detect changes in the key primary behavioural outcomes (travel behaviour and physical activity) and the most important secondary outcome (the incidence of road traffic accidents)". ".....a cross-sectional sample of 400 participants per study area at each time point (a target that was exceeded at baseline) was expected to allow the detection with 95% confidence and 80% power of an increase of 5 minutes per day in walking for transport from baseline to follow-up within one study area, or a cross-sectional difference of 2 minutes per day in cycling for transport or 5 minutes per day in walking for transport between intervention and control areas at follow-up." "For physical activity, assuming a baseline (control) mean value for accelerometer-derived MVPA of 10 minutes per day and a SD of 7 minutes per day, 82,83 86 participants per group were required to detect a difference of 3 minutes per day in MVPA between intervention and control areas." (Ogilvie 2017)  **Study aim or objective:** "The effects of the new motorway on patterns of travel, physical activity and wellbeing in local residents were evaluated using a quasi-experimental study design."  **Time points of data collection:** Two: Baseline (October 2005), and follow-up (2013)  **Unit of allocation or exposure:** Geographical area |
| --- | --- |
| **Participants** | **Baseline Characteristics**  Intervention   - *Age*: Cohort mean (SD): 51.0 (14.1); Cross-sectional: 48.1 (17.8) - *Place of residence*: Years lived in local areas, mean (SD) - Cohort: 20.3 (18.4); Cross-sectional: 17.3 (18.4) - *Sex*: % male: Cohort: 48.8; Cross-sectional: 41.3 - *Ethnicity and language*: NR - *Occupation*: % working (in paid part/full-time employment, full time student, voluntary work) - Cohort: 59.7; Cross-sectional: 48.9 - *Education*: NR - *Socioeconomic status*: NR - *Social capital*: Home ownership %: Cohort: 61.3; Cross-sectional: 46.4; Car ownership %: Cohort: 60.8; Cross-sectional: 47.6 - *Physical activity*: % participated in MVPA: Cohort: 77.8; Cross-sectional: 65.5. MVPA time (min/day), mean (SD) - Cohort: 568.8 (508.9); Cross-sectional: 574.9 (542.8)   Control - area with no motorway or similar infrastructure   - *Age*: Cohort mean (SD): 49.0 (13.3); Cross-sectional: 49.7 (18.2) - *Place of residence*: Years lived in local areas, mean (SD) - Cohort: 16.9 (13.1); Cross-sectional: 18.9 (18.7) - *Sex*: % male: Cohort: 37.6; Cross-sectional: 36.2 - *Ethnicity and language*: NR - *Occupation*: % working (in paid part/full-time employment, full time student, voluntary work) - Cohort: 60.8; Cross-sectional: 47.2 - *Education*: NR - *Socioeconomic status*: NR - *Social capital*: Home ownership % Cohort: 60.8; Cross-sectional: 46.3; Car ownership % Cohort: 61.6; Cross-sectional: 49.4 - *Physical activity*: % participated in MVPA: Cohort: 75.3; Cross-sectional: 62.0. MVPA time (min/day), mean (SD) - Cohort: 436.5 (466.7); Cross-sectional: 573.1 (534.2)   Control - different area with other motorways   - *Age*: Cohort mean (SD): 51.3 (13.3); Cross-sectional: 48.5 (18.7) - *Place of residence*: Years lived in local areas, mean (SD) - Cohort: 17.5 (13.5); Cross-sectional: 18.2 (16.9) - *Sex*: % male: Cohort: 44.1; Cross-sectional: 34.0 - *Ethnicity and language*: NR - *Occupation*: % working (in paid part/full-time employment, full time student, voluntary work) - Cohort: 54.6; Cross-sectional: 48.9 - *Education*: NR - *Socioeconomic status*: NR - *Social capital*: % home ownership - Cohort: 61.3; Cross-sectional: 51.1. % car ownership - Cohort: 52.3; Cross-sectional: 49.4 - *Physical activity*: % participated in MVPA: Cohort: 73.8; Cross-sectional: 70.7 MVPA time (min/day), mean (SD) - Cohort: 473.9 (485.8); Cross-sectional: 565.9 (449.3)   Overall   - *Age*: Age in years, mean (SD) - Cohort: 50.4 (13.6); Cross-sectional: 48.8 (18.3) - *Place of residence*: Years lived in local areas, mean (SD) - Cohort: 18.3 (15.3); Cross-sectional: 18.2 (18.0) - *Sex*: % male - Cohort: 43.5; Cross-sectional: 37.1 - *Ethnicity and language*: NR - *Occupation*: % working (in paid part/full-time employment, full time student, voluntary work) - Cohort: 58.5; Cross-sectional: 48.3 - *Education*: NR - *Socioeconomic status*: NR - *Social capital*: % home ownership - Cohort: 61.1; Cross-sectional: 47.9. % car ownership - Cohort: 58.5; Cross-sectional: 48.8 - *Physical activity*: NR   **Included criteria:** "At baseline (2005; T1), eligible participants were adults aged ≥ 16 years residing in one of the three study areas, who responded to a postal survey delivered to their home address. If more than one householder was eligible, the individual with the most recent birthday was asked to complete the survey. At follow-up (2013; T2), eligible participants were (1) those who had responded to the postal survey at baseline, had not moved out of the UK, and responded to a subsequent postal survey at follow-up; or (2) adults aged ≥ 16 years residing in one of the three study areas, who responded to a postal survey delivered to their home address."  **Excluded criteria:** NR  **Pretreatment:** "Baseline analysis confirmed no significant differences between the achieved survey samples in these three areas on any socioeconomic or behavioural summary measures apart from a minor difference of borderline statistical significance (p = 0.053) in the distribution of housing tenure."  **Attrition per relevant group:** Because of the study sampling strategy, there is no attrition per se. Those individuals who responded at both baseline and follow-up were included in the cohort analysis. Where an individual was not available at follow-up, his/her data were included in the cross-sectional analysis, and new individuals were sampled for the follow-up time period, also to be included in the cross-sectional analysis. "The response rate was similar at both time points: 1345/(9000–676) = 16.1% at baseline and 1343/(9000–509) = 15.8% at follow-up." Ogilvie 2017 n=6979 did not respond at baseline and n=7148 did not respond at follow-up. Attrition was associated with younger age and unemployment/not studying.  **How were baseline differences assessed?:** "Differences in covariates and outcomes between the study areas at baseline and follow-up, and between the cohort and repeat cross-sectional samples, were studied using one-way analysis of variance and chi-square tests as appropriate. "  **Total number completed and analysed per relevant group:** Numbers varied per outcome analysed. See tables 16, 19-20, 31 (Ogilvie 2017)  **Total number in each relevant group pre-intervention:** "The longitudinal cohort comprised 365 participants who returned surveys at both time points. The remaining 980 (baseline) and 978 (follow-up) participants together formed the repeat cross-sectional sample."  **Total number randomised per relevant group:** NA |
| **Interventions** | **Intervention Characteristics**  Intervention: New motorway   - *Type of intervention (e.g. infrastructure, policy or both)*: Infrastructure - *Description of main intervention*: the core of the intervention comprised the construction of a new 5-mile, six-lane section of motorway, which is mostly elevated above ground and runs through a predominantly urban, deprived area of south-east Glasgow. "The new motorway was intended to relieve through traffic on an existing urban motorway, the M8. However, this infrastructural project was also intended to promote economic regeneration, and was hypothesized to remove traffic from local streets. Proponents of the scheme argued that the motorway would create a more pedestrian- and cycle-friendly environment." - *Description of additional intervention components*: "...a number of mitigation strategies would be in place to combat noise and vibration from the motorway, including low-noise road surfacing and noise barriers." - *Duration of intervention*: Introduced in 2011, permanent thereafter - *How was the intervention implemented?*: NR - *Co-interventions*: "The new motorway formed one component of a wider strategic initiative to regenerate the local area." This included demolition of commercial and industrial sites as well as loss of green space. - *Where was the intervention implemented?*: Urban deprived area in Southern Glasgow (see figure 4, Ogilvie 2017) - *Resource requirements to replicate the intervention*: NR - *Economic indicators*: NR   Control - area with no motorway or similar infrastructure  Control - different area with other motorways   - *Type of intervention (e.g. infrastructure, policy or both)*: Infrastructure (throughout the entire study period, i.e. no new implementation) - *Description of main intervention*: "...already existing motorway" - *Description of additional intervention components*: NR - *Duration of intervention*: Existed throughout the entire study period; permanent - *How was the intervention implemented?*: NR - *Co-interventions*: Unclear - *Where was the intervention implemented?*: Urban deprived area in Eastern Glasgow (see figure 4, Ogilvie 2017) - *Resource requirements to replicate the intervention*: NR - *Economic indicators*: NR |
| **Identification** | **Sponsorship source:** "DO and RP are supported by the Medical Research Council [Unit Programme number MC_UU_12015/6]; and the M74 study was conducted under the auspices of the Centre for Diet and Activity Research (CEDAR), for which funding from the British Heart Foundation, Cancer Research UK, Economic and Social Research Council, Medical Research Council, National Institute for Health Research and Wellcome Trust, under the auspices of the UK Clinical Research Collaboration, is gratefully acknowledged." "The baseline phase of the study was developed by David Ogilvie, Richard Mitchell, Nanette Mutrie, Mark Petticrew and Stephen Platt and supported by a Medical Research Council Special Training Fellowship in Health of the Public Research (award number G106/1203). The follow-up phase of the study was developed by David Ogilvie, Lyndal Bond, Fiona Crawford, Simon Griffin, Shona Hilton, David Humphreys, Andy Jones, Richard Mitchell, Nanette Mutrie, Shannon Sahlqvist and Hilary Thomson and funded by the National Institute for Health Research Public Health Research programme (NIHR PHR; project number 11/3005/07)."  **Country:** UK - Scotland  **Setting:** Deprived urban areas  **Comments:**  **Author’s name:** David Ogilvie  **Institution:** MRC Epidemiology Unit and UKCRC Centre for Diet and Activity Research, University of Cambridge  **Email:** david.ogilvie@mrc-epid.cam.ac.uk  **Address:**  **Declaration of interests stated?:** "The researchers were independent of the funders, and the latter had no role in study design, data collection and analysis, the decision to publish, or the preparation of the manuscript."  **Specify any documents relevant to this study:** Ogilvie 2006 - methodology case study; Ogilvie 2017 - main paper; reports primary outcomes; Prins 2017 - reports secondary outcomes, also includes Additional file 1, with relevant tables  **Study or programme name or acronym:** The Traffic and Health in Glasgow study  **Type of record:** Journal article |
| **Notes** |  |

## Risk-of-bias table

| **Bias** | **Authors' judgement** | **Support for judgement** |
| --- | --- | --- |
| Random sequence generation (selection bias) | High risk | CBA study. This is a natural experiment in which the control sites were selected due to specific characteristics (i.e. either presence or lack of existing roadway, as well as similarities with regard to demographic characteristics). |
| Allocation concealment (selection bias) | High risk | This is a CBA study. Allocation was not concealed, the researchers defined the study areas. |
| Baseline characteristics similar | Low risk | Baseline outcome values stratified by group are found in Additional File 1; regression models are adjusted for these aspects: age, gender, home ownership, car ownership, work status and time lived in neighbourhood. |
| Baseline outcome measurements similar | High risk | For the cohort analysis, the participants in the intervention area participated in more min/week of MVPA than either control group. In the cross-sectional analysis, the follow up sample included participants not included at baseline, so their baseline characteristics are unknown. The differences between the areas at baseline in terms of min/week of MVPA are similar. |
| Blinding of participants and personnel (performance bias) | Low risk | No blinding possible in this type of intervention, however, it is unlikely to have influenced behavior and introduced performance bias. |
| Blinding of outcome assessment (detection bias) | High risk | Outcomes were not assessed blindly. Outcomes were based on self-report on a mail survey and perceptions and preferences about the motorway could have influenced the self-report. |
| Protection against contamination | Low risk | Participants lived in different geographical study areas and it is unlikely that the control group with no motorway was exposed to the intervention (new motorway). |
| Incomplete outcome data (attrition bias) | High risk | As described in the discussion, many people from the cohort were not available at follow-up. Those remaining were different than those at follow-up, and this difference may have biased the results. "Attrition in the cohort was substantial, albeit similar to that of other studies [25]. Those remaining in the cohort were on average older and more likely to work than the rest of the baseline sample." |
| Selective reporting (reporting bias) | Low risk | Outcomes specified in the methods section are reported in the papers |
| Other bias | High risk | Misclassification bias - low risk. Participants were sent the survey to their address, which would be within a specified study area. Measurement bias - unclear risk. Self-report of physical activity may lead to overestimation. Incorrect analysis - n/a. Other bias - High risk. Very likely that other aspects changed between the two data collection points, both city-wide, but also in each area, that also influenced physical activity. These aspects could substantially bias results. |

# Quigg 2012

| **Methods** | **Study design:** CBA  **Study grouping:** n/a  **How was missing data handled?:** Missing data was excluded from the analysis: "All participants who moved away from Dunedin residences between assessments were excluded from the follow-up analyses." Some data from accelerometers was excluded, if considered not valid ("More than 5 hours of consecutive zero counts would be assumed to indicate non-wearing of the units, and the remaining data were classified as not valid").  **How were participants recruited and sampled within the study sites?:** "A two-stage sampling process was used to recruit participants. First, elementary school administrators in the control and intervention communities were asked to provide access to their pupils. Of the six schools in the intervention community, four agreed to participate. In the control community, all four schools who were asked agreed to participate. The second stage of sampling required eligible children to be recruited from the eight participating schools. Inclusion and exclusion criteria were developed and applied uniformly to ensure participants were anticipated to be available for follow-up assessment 1 year later and to maximize the comparability of participants from the two communities." "If more than one child per family had consented to participate, one child from that family was selected at random for enrollment in the study."  **Methods for selecting intervention and control sites:** "The Dunedin City Council (DCC) playground upgrade program identified the intervention community" and also broadly similar communities to serve as controls. "New Zealand (NZ) central and local government published reports were used to identify potential control communities and provide identifiable and plausible community factors." (Factors listed in the paper)  **Number of control sites:** 1 community (4 schools; 88 children). Number of playgrounds not reported.  **Number of intervention sites:** 1 community (4 schools; 96 children). "two playgrounds selected for upgrading from the six playgrounds in the intervention community  **Post-intervention period (start and end date or year it was conducted):**  Intervention remains accessible.  **Pre-intervention time period (before intervention start):** October to December 2007 (baseline measurements)  **Sample size justification and outcome used:** 63 participants in each group would provide 80% power to detect a standardized effect size difference of 0.5 standard deviations in mean physical activity (calculated over 6 days at follow-up) between communities using a two-sided test at the 0.05 level. School design effects were assumed to be negligible or at least low enough to be compensated for by any correlation between measurements at baseline and follow-up. Allowing for a 33% loss to follow-up rate, mostly expected to be from participants moving residence out of the study areas,100 participants in each group at baseline assessment stage would be sufficient.  **Study aim or objective:** "... to evaluate changes in physical activity for children when playgrounds located in public parks within their community were upgraded." "The study hypothesis is that upgrading playgrounds in a community would be associated with an increase in the accelerometer-measured, total daily physical activity (TDPA) of children attending schools located in that community compared with a matched control community."  **Time points of data collection:** Baseline: October to December 2007 Follow up: October to December 2008.  **Unit of allocation or exposure:** Community |
| --- | --- |
| **Participants** | **Baseline Characteristics**  Intervention   - *Age*: n(%) in age group - 5-6 years: 27 (28.1), 7-8 years: 31 (32.3), 9-10 years: 38 (39.6) - *Place of residence*: NR - *Sex*: Females, n(%): 46 (47.9) - *Ethnicity and language*: Ethnicity, n(%) - NZ Maori: 23 (23.9), Pacific: 6 (6.3), NZEO: 72 (75) - *Occupation*: NR - *Education*: NR - *Socioeconomic status*: NR - *Social capital*: NR - *BMI*: BMI category, n(%): Normal weight: 67 (69.8), overweight: 20 (20.8), obese: 9 (9.4)   Control   - *Age*: n(%) in age group - 5-6 years: 22 (25.0), 7-8 years: 34 (38.6), 9-10 years: 31 (35.2) - *Place of residence*: NR - *Sex*: Females, n(%): 53 (60.2) - *Ethnicity and language*: Ethnicity, n(%) - NZ Maori: 19 (21.6), Pacific: 4 (4.5), NZEO: 68 (77.3) - *Occupation*: NR - *Education*: NR - *Socioeconomic status*: NR - *Social capital*: NR - *BMI*: BMI category, n(%): Normal weight: 61 (69.3), overweight: 17 (19.3), obese: 10 (11.4)   Overall   - *Age*: n (%) age groups: 5-6 years: 49 (27); 7-8 years: 66 (36); 9-10 years: 69 (38) - *Place of residence*: Deprived urban areas - *Sex*: n (%) female: 99 (54) - *Ethnicity and language*: Ethnicity, n(%) - NZ Maori: 42 (22.8), Pacific: 10 (5.4), NZEO: 140 (76.1) - *Occupation*: NR - *Education*: NR - *Socioeconomic status*: NR - *Social capital*: NR - *BMI*: n (%) BMI category - normal: 128 (70); overweight: 37 (20); obese: 19 (10)   **Included criteria:** "The The Dunedin City Council (DCC) playground upgrade program identified the intervention community" but no criteria were provided. Criteria to select children from each consenting/participating school included:1. School Children between the ages of 5 and 10 years old (inclusive) at the time of baseline assessment 2. Classified as year 0 to 5 (inclusive) (kindergarten to grade 4)3. Residing 4 or more nights per week within the defined community.  **Excluded criteria:** None reported  **Pretreatment:** Authors do not report an assessment of baseline characteristics. According to table 1: gender differences : more females in the control group and more males in the intervention group. 22% more females than males in the control group. (n=79) 6% more males than females in the intervention group (n=77)  **Attrition per relevant group:** 19 children in intervention and 9 children in control groups LTFU.  **How were baseline differences assessed?:** NR (contact author)  **Total number completed and analysed per relevant group:** 77 children in intervention and 79 children in control groups  **Total number in each relevant group pre-intervention:** 96 children in intervention and 88 children in control groups  **Total number randomised per relevant group:** n/a |
| **Interventions** | **Intervention Characteristics**  Intervention   - *Type of intervention (e.g. infrastructure, policy or both)*: Infrastructure - *Description of main intervention*: "The intervention, the playground upgrades, was conceived and managed by the local authority with no influence from the research team. The DCC followed the community consultation processes outlined in their policy documents.20,23 The results of this process identified two playgrounds selected for upgrading from the six playgrounds in the intervention community. At one playground, ten new components,including play equipment, seating, additional safety surfacing, and waste facilities,were installed, and two existing components were removed. At the other playground,two new play equipment pieces were installed, and a small modification was made to another piece of equipment." "All playground upgrades were completed 3 months before the follow-up physical activity measurement phase." - *Description of additional intervention components*: None reported - *Duration of intervention*: Unclear (From June 2008??) - *How was the intervention implemented?*: "The intervention, the playground upgrades, was conceived and managed by the local authority with no influence from the research team. The DCC followed the community consultation processes outlined in their policy documents." - *Co-interventions*: None reported - *Where was the intervention implemented?*: Two of six playgrounds in the intervention community in Dunedin, New Zealand - *Resource requirements to replicate the intervention*: NR - *Economic indicators*: NR   Control: No intervention |
| **Identification** | **Sponsorship source:** The study was supported by the Ministry of Health District Health Board Healthy Eating Healthy Action (HEHA) Evaluation Fund, the University of Otago, the Otago Healthcare Charitable Trust, the Dunedin City Council, and Sport and Recreation New Zealand. Dr. Reeder was supported by a grant to the Cancer Society Social & Behavioural Research Unit from the Cancer Society of New Zealand Inc. and by the University of Otago.  **Country:** New Zealand  **Setting:** Communities in Dunedin City, New Zealand  **Comments:**  **Author’s name:** Robin Quigg  **Institution:**  **Email:** robin.quigg@otago.ac.nz  **Address:**  **Declaration of interests stated?:** Yes. The authors have no professional relationships with companies or manufacturers who will benefit from the results of the present study.  **Specify any documents relevant to this study:** N/A  **Study or programme name or acronym:** N/A  **Type of record:** Journal article |
| **Notes** |  |

## Risk-of-bias table

| **Bias** | **Authors' judgement** | **Support for judgement** |
| --- | --- | --- |
| Random sequence generation (selection bias) | High risk | This was a CBA and randomisation was not done. |
| Allocation concealment (selection bias) | High risk | This was a CBA. |
| Baseline characteristics similar | Unclear risk | Authors did not report a comparison of baseline characteristics between the intervention and control groups. |
| Baseline outcome measurements similar | Unclear risk | Authors do not report a comparison between intervention and control groups. |
| Blinding of participants and personnel (performance bias) | Low risk | There was no blinding. Participants in the intervention group had to wear an Actigraph belt, which could have influenced their PA-related behavior. |
| Blinding of outcome assessment (detection bias) | Low risk | Outcome was objective, measured using Actigraph belt, and thus unlikely to be influenced by lack of blinding. |
| Protection against contamination | Unclear risk | Intervention and control groups were different communities. It is not clear how distant they were from each other. |
| Incomplete outcome data (attrition bias) | Low risk | 19.8% participants LTFU from intervention groups vs 10.2% LTFU from control group, and reasons for LTFU not reported. However, authors report no differences in characteristics of children LTFU compared to those who remained in the study. |
| Selective reporting (reporting bias) | Unclear risk | TDPA was the stated primary outcome but this was not clearly reported. |
| Other bias | Low risk | Misclassification bias is unlikely as this was assigned to participants. Measurement bias - unlikely as physical activity was measured using a validated tool. Incorrect statistical analysis - N/A |

# Ostergaard 2015

| **Methods** | **Study design:** Controlled before-after study  **Study grouping:** Parallel group  **How was missing data handled?:** It is not explicitly stated in the text, but it seems that missing data were simply excluded from the analysis. Authors do write in the discussion that a post-hoc analysis not reported in the results with only complete cases, yielded similar results  **How were participants recruited and sampled within the study sites?:** 2415  **Methods for selecting intervention and control sites:** Intervention schools were those where "local plans for infrastructural changes near schools and school motivation for implementing school cycling interventions" existed. It is not explicitly stated in the paper, but likely control schools were all other schools in the area that were not "involved in any physical activity promotion projects during the study period”.  **Number of control sites:** 12  **Number of intervention sites:** 13  **Post-intervention period (start and end date or year it was conducted):** Baseline data collection was April and May 2010, and follow up data collection was April and May 2011. The exact date of intervention implementation is not specified, but it occurred some time between these two time points.  **Pre-intervention time period (before intervention start):** Baseline data collection was April and May 2010  **Sample size justification and outcome used:** Not reported  **Study aim or objective:** "The main aim of this study was to assess the effectiveness of the school cycling promotion programme entitled “Tryg og Sikker Skolecykling” (Safe and secure cycling to school) on school cycling while also assessing potential concomitant health effects. Furthermore, since cycling-related injuries are often thought to supersede the preventive health beneficial effects [18] the secondary aim of this study was to quantify the incidence, the predictors and the number of injuries related to cycling to school."  **Time points of data collection:** 2 points. Baseline data collection was April and May 2010; Follow up data collection was April and May 2011.  **Unit of allocation or exposure:** School |
| --- | --- |
| **Participants** | **Baseline Characteristics**  Cycling Infrastructure changes   - *Age*: 11.0 (0.64) - *Place of residence*: - *Sex*: 51.1% male - *Ethnicity and language*: - *Occupation*: School pupil - *Education*: - *Socioeconomic status*: - *Social capital*:   No Intervention   - *Age*: 10.9 (0.63) - *Place of residence*: - *Sex*: 48.8% male - *Ethnicity and language*: - *Occupation*: School pupil - *Education*: - *Socioeconomic status*: - *Social capital*:   Overall   - *Age*: - *Place of residence*: - *Sex*: - *Ethnicity and language*: - *Occupation*: - *Education*: - *Socioeconomic status*: - *Social capital*:   **Included criteria:** "At baseline all children (n=2415) from the 4th and 5th grade were included into the study using public school registrations."  **Excluded criteria:** Those who left their respective schools/joined the schools after the initial school recruitment were excluded (n=14).  **Pretreatment:** See table 1: Several differences exist between the intervention and control groups at baseline (age, height, cardiorespiratory fitness, long-term school cycling, cycling last week beyond school cycling, school cycling trips last week).  **Attrition per relevant group:** See Table 1 for number in each group at baseline (1296 intervention; 1105 control) and additional File 1, Table A for number included in each analysis. Attrition was approximately 29% and 40% for the intervention and control groups, respectively.  **How were baseline differences assessed?:** "Differences in continuous outcomes (including ordinal variables treated as continuous) between the control group and the intervention group were tested using t tests (Table 1) or using adjusted multiple linear regression analyses (Table 2). Chi-Square tests were used in order to test the differences in distributions in the control group compared to the intervention group (Tables 1, 3 and Additional file 1: Table A)."  **Total number completed and analysed per relevant group:** See additional File 1, Table A. There are slight differences for the various outcomes; approximately n=923 for the intervention and n=664 for the control group.  **Total number in each relevant group pre-intervention:** Intervention: 1296 Control: 1105  **Total number randomised per relevant group:** NA |
| **Interventions** | **Intervention Characteristics**  Cycling Infrastructure changes   - *Type of intervention (e.g. infrastructure, policy or both)*: Infrastructure - *Description of main intervention*: "physical environmental characteristics" as part of a package of interventions to increase cycling; ---------------------'Hard interventions' were planned ahead of the study and included "structural changes near the school in e.g. road surface, signposting and traffic regulation such as one-way streets and regulation of car drop off zones". - *Description of additional intervention components*: "...soft interventions generally focused on increasing school cycling motivation (e.g. through competitions and monitoring) as well as cycling safety (e.g. through school traffic policy, cycle training and bicycle maintenance." - *Duration of intervention*: Not explicitly stated; implementation started after baseline (April-May 2010); most of the 'hard' interventions were likely permanent. - *How was the intervention implemented?*: "The cycling incentives at the intervention schools consisted of 'hard' interventions planned ahead of this study by the local authorities and 'soft' interventions which were initiatives implemented by The Danish Cyclists Federation." - *Co-interventions*: Not reported - *Where was the intervention implemented?*: Near schools in three municipalities in Denmark: Copenhagen, Fredericia and island of Funen. - *Resource requirements to replicate the intervention*: NR - *Economic indicators*: NR   No Intervention   - *Type of intervention (e.g. infrastructure, policy or both)*: Infrastructure (to a very slight extent) - *Description of main intervention*: Some minor interventions were implemented "it was evident that we had difficulties passing on the scientific importance that control schools were not offered any cycling interventions. This might have diluted observed effects since some minor interventions were conducted at some control schools (cf. Additional file 1: Table B)." - *Description of additional intervention components*: No intervention - *Duration of intervention*: No intervention - *How was the intervention implemented?*: No intervention - *Co-interventions*: "It was required that the control schools were not involved in any physical activity promotion projects during the study period." - *Where was the intervention implemented?*: No intervention - *Resource requirements to replicate the intervention*: No intervention - *Economic indicators*: No intervention |
| **Identification** | **Sponsorship source:** "The study was financially supported by Trygfonden. The study sponsor had no role in the study design; collection, analysis, and interpretation of data; writing of the manuscript; and the decision to submit the manuscript for publication."  **Country:** Denmark  **Setting:** Municipalities of Copenhagen and Fredericia, and the island of Funen  **Comments:**  **Author’s name:** Lars Ostergaard  **Institution:**  **Email:** lostergaard@health.sdu.dk  **Address:**  **Declaration of interests stated?:** "The authors declare that they have no competing interests"  **Specify any documents relevant to this study:** Additional file: Additional file 1:Table A. Unadjusted changes in leisure time physical activity and cycling behaviour. Table B.Detailed information on the school based multifaceted interventions. Table C. Detailed information on the distribution of available information (i.e. the degree of missing) at baseline questionnaire assessed variables as well as variables assessed through physical testing by the three different regions.  **Study or programme name or acronym:** "Tryg og Sikker Skolecykling"/ Safe and secure cycling to school"  **Type of record:** Journal article |
| **Notes** |  |

## Risk-of-bias table

| **Bias** | **Authors' judgement** | **Support for judgement** |
| --- | --- | --- |
| Random sequence generation (selection bias) | High risk | CBA study "Public schools in the municipality of Copenhagen, the municipality of Fredericia and schools on the island of Funen were, based on the existence of local plans for infrastructural changes near schools and school motivation for implementing school cycling interventions, included into this quasi-experimental study as either control schools (n=12) or intervention schools (n=13) by the Danish Cyclists Federation." |
| Allocation concealment (selection bias) | High risk | CBA study; allocation not concealed. |
| Baseline characteristics similar | Low risk | As evident in table one there were differences in gender and age (statistically significant for age). However, these differences were very slight and unlikely clinically relevant. Additionally, analyses were adjusted for age and gender. |
| Baseline outcome measurements similar | Low risk | As evident in table one there were differences in some outcomes, including the cycling outcomes. Models were, however, adjusted for baseline outcome values, so this did not likely bias results meaningfully. |
| Blinding of participants and personnel (performance bias) | Low risk | Participants were not blinded. All outcomes related to physical activity were subjectively measured using questionnaires, thus it is likely that the presence of the interventions at intervention schools influenced how those students responded. |
| Blinding of outcome assessment (detection bias) | High risk | For the subjectively measured outcomes the lack of blinding of outcome assessors could have introduced bias. |
| Protection against contamination | High risk | There was substantial contamination between the two groups. In Additional file 1: Table B it can be observed that several 'interventions' were also carried out at control schools."...it was evident that we had difficulties passing on the scientific importance that control schools were not offered any cycling interventions. This might have diluted observed effects since some minor interventions were conducted at some control schools (cf. Additional file 1: Table B)." |
| Incomplete outcome data (attrition bias) | High risk | From the discussion: "We cannot rule out that the large extent of missing values have caused biased results..." - Attrition was high, and this likely introduced some bias. |
| Selective reporting (reporting bias) | Low risk | There is no evidence of selective outcome reporting. Methods and measurements seems consistent at baseline and follow-up; all analyses is reported either in main article or in supplementary file (except the post hoc analyses/sensitivity analyses). |
| Other bias | Low risk | No further sources of bias identified. |

# Richardson 2020

| **Methods** | **Study design:** CBA study  **How was missing data handled?:** Some missing data on park observations were excluded as observations on the same day/time at follow-up as at baseline was not possible (n = 10). 2 parks were excluded from the analysis as they were inaccessible at follow-up due to renovations still ongoing.  **How were participants recruited and sampled within the study sites?:** Participants visiting parks were observed; no recruitment was necessary.  **Methods for selecting intervention and control sites:** Parks in intervention and control neighbourhoods were selected. Intervention neighbourhood was selected as it was part of a new project for greenspace improvement: "In Pittsburgh, a plan (known as Greenprint) for greenspace improvement in the Hill District neighborhood, a low income, predominantly African American urban neighborhood, presented an opportunity to examine the role of neighborhood investments in greenspace and surrounds on park use." Control site: "Homewood, a socio-demographically similar Pittsburgh neighborhood, was selected to serve as a comparison neighborhood to control for secular changes that may have been occurring regardless of neighborhood investments."  **Number of control sites:** 1 neighborhood (8 parks in the intervention neighborhood)  **Number of intervention sites:** 1 neighborhood (9 parks in the control neighborhood)  **Post-intervention period (start and end date or year it was conducted):** Park improvements were finished in 2015, when follow-up data collection was carried out (August to October 2015). Given the nature of the intervention, there is no end date.  **Pre-intervention time period (before intervention start):** August to October 2012: baseline data collection, with improvements to parks and neighbourhoods taking place between then and 2015.  **Sample size justification and outcome used:** NR  **Study aim or objective:** "This study assessed the impact of greenspace, housing, and commercial investments on street characteristics (walkability, amenities, incivilities/poor esthetics) and park use by examining park use over time in two low income neighborhoods in Pittsburgh, PA (n = 17 parks), before and after neighborhood-based renovations that were primarily centered in one neighborhood."  **Time points of data collection:** Baseline: Aug - Oct 2012; Follow-up: Aug-Oct 2015  **Unit of allocation or exposure:** Neighbourhood |
| --- | --- |
| **Participants** | **Baseline Characteristics**  Intervention   - *Age*: Type of park users, mean (SD): children 5.2 (5.5); teenagers: 1.6 (0.4); adults: 3.2 (1.5) - *Place of residence*: Hill District neighbourhood (socio-economically disadvantaged) - *Sex*: Park users, mean (SD - female: 2.4 (1.1); male: 6.4 (3.8) - *Ethnicity and language*: NR - *Occupation*: NR - *Education*: NR - *Socioeconomic status*: Walkability, mean (SD): 0.4 (0.4); Amenities, mean (SD): 0.2 (0.3); Incivilities/poor esthetics, mean (SD): − 1.2 (0.2). Neighbourhood is socioeconomically disadvantaged. - *Social capital*: NR - *Level of physical activity*: Park users by type of activity, mean (SD) - sedentary: 5.4 (4.2); moderate to vigorous: 3.3 (1.0)   Control   - *Age*: Type of park users, mean (SD): children 3.7 (0.9); teenagers: 1.7 (0.9); adults: 3.6 (2.2) - *Place of residence*: Homewood neighbourhood (socio-economically disadvantaged) - *Sex*: Park users, mean (SD - female: 2.3 (0.8); male: 5.5 (2.2) - *Ethnicity and language*: NR - *Occupation*: NR - *Education*: NR - *Socioeconomic status*: Walkability, mean (SD): − 0.4 (0.3); Amenities, mean (SD): 0.1 (0.4); Incivilities/poor esthetics, mean (SD): 0.7 (0.1). Neighborhood is socioeconomically disadvantaged. - *Social capital*: NR - *Level of physical activity*: park users, mean (SD) - sedentary: 4.9 (2.0); moderate to vigorous: 3.5 (0.9)   Overall: NR  **Included criteria:** NR  **Excluded criteria:** NR  **Pretreatment:** At baseline, the parks in the intervention neighbourhood were larger than in the control neighbourhood (0.2 to 23.3 acres vs 0.2 to 10.8, respectively) and the intervention neighborhood appeared to have greater walkability and fewer incivilities/better esthetics than the control neighbourhood.  **Attrition per relevant group:** 2 parks were excluded from the analysis as the renovations were still ongoing at follow-up. it is unclear whether these are from the same or different neighbourhoods.  **How were baseline differences assessed?:** NR. Differences are just mentioned in the text of the Results section.  **Total number completed and analysed per relevant group:** Total: 17 parks Intervention group: 8 parks Control group: 9 parks  **Total number in each relevant group pre-intervention:** Total: 19 parks  **Total number randomised per relevant group:** n/a |
| **Interventions** | **Intervention Characteristics**  Intervention   - *Type of intervention (e.g. infrastructure, policy or both)*: Infrastructure - *Description of main intervention*: Green space interventions to increase walkability, aesthetics in neighbourhoods surrounding parks. - *Description of additional intervention components*: "creation and renovation of current greenspace, including multiple parks, six outdoor stairwells, and three trails connecting parks" . It is unclear but it seems that most of the interventions were not carried out in the parks but in surrounding areas. - *Duration of intervention*: 2015 - onwards - *How was the intervention implemented?*: "The intervention neighborhood faced delays in expansive greenspace renovations that were scheduled to occur during the study period (2012–2015), including the opening of multiple new parks (which ultimately happened following the study period). The Cliffside/August Wilson park was fully renovated and opened to the public in August 2016." "this study was challenged by the fact that the originally planned changes did not happen according to schedule or of the predicted magnitude that was originally anticipated." - *Co-interventions*: "Nonetheless, there were other major investments that directly changed the landscape of Hill District, including major public housing development construction and renovations that changed the surrounding neighborhood characteristics." - *Where was the intervention implemented?*: In a selected neighbourhood, in areas surrounding the parks - *Resource requirements to replicate the intervention*: NR - *Economic indicators*: NR   Control   - *Type of intervention (e.g. infrastructure, policy or both)*: No intervention - *Description of main intervention*: "While developments were also planned for Homewood, the scale of the investments was much lower than it was in Hill District." - *Description of additional intervention components*: No intervention - *Duration of intervention*: No intervention - *How was the intervention implemented?*: N/A - *Co-interventions*: N/A - *Where was the intervention implemented?*: In Homewood district, Pittsburgh - *Resource requirements to replicate the intervention*: NR - *Economic indicators*: NR |
| **Identification** | **Sponsorship source:** "Funding was provided by the National Cancer Institute (Grant No. R01CA164137 “Impact of Greenspace Improvement on Physical Activity in a Low Income Community” and National Heart Lung Blood Institute (Grant No. R01 HL122460 Neighborhood Change: Impact on Sleep and Obesity-Related Health Disparities”)."  **Country:** USA  **Setting:** Low income, urban neighborhoods  **Comments:**  **Author’s name:** Andrea S. Richardson  **Institution:**  **Email:** arichard@rand.org  **Address:**  **Declaration of interests stated?:** NR  **Specify any documents relevant to this study:** N/A  **Study or programme name or acronym:** Pittsburgh Hill/Homewood Research on Neighborhoods, Exercise and Health (also known as PHRESH Plus)  **Type of record:** Journal article |
| **Notes** | Linked studies: Dubowitz 2019(28) |

## Risk-of-bias table

| **Bias** | **Authors' judgement** | **Support for judgement** |
| --- | --- | --- |
| Random sequence generation (selection bias) | High risk | This is a CBA study and no randomisation was carried out. |
| Allocation concealment (selection bias) | High risk | This is a CBA study and no randomisation was carried out. |
| Baseline characteristics similar | High risk | At baseline, in the intervention neighbourhood Parks were bigger, it had greater walkability and fewer incivilities/better esthetics than the comparison, the number of amenities was similar across neighborhoods. Only the size of the park was adjusted for in the DID analyses. |
| Baseline outcome measurements similar | High risk | No formal methods were used to assess baseline differences. From the data presented it appears that in the intervention neighbourhood park use was higher at baseline. |
| Blinding of participants and personnel (performance bias) | Low risk | Blinding in these types of interventions is not possible. However, it is unlikely that this will influence the delivery of the intervention and experience of participants in ways other than intended. |
| Blinding of outcome assessment (detection bias) | High risk | No blinding was possible. Outcome assessors were trained fieldworkers who used validated tools to assess park users and their PA, as well as neighbourhood qualities. It is unclear whether knowledge of which was the intervention and control neighbourhood would have influenced their observations. |
| Protection against contamination | Unclear risk | The control group also received some investments but on a smaller scale; these are not clearly described. Therefore, there might have been similar improvements carried out in the control neighbourhood, however, the analysis of neighbourhood characteristics indicates that the control neighbourhood did not improve in walkability or amenities, but it performed better in terms of poor esthetics/incivilities. |
| Incomplete outcome data (attrition bias) | Unclear risk | Two parks (of 19) were excluded from analysis as they were still under renovation at follow-up. It is unclear whether these parks were from the intervention or control group. |
| Selective reporting (reporting bias) | Low risk | The protocol could not be accessed but all outcomes specified in the Methods section are reported in the Results section. |
| Selective reporting (reporting bias) | Low risk | The protocol could not be accessed but all outcomes specified in the Methods section are reported in the Results section. |
| Other bias | Low risk | Misclassification of exposure: low risk; fieldworkers surveyed designated neighbourhoods and parks. Measurement bias: low risk; validated instruments used by trained fieldworkers. Incorrect analysis: n/a |

# Rissel 2015

| **Methods** | **Study design:** CBA  **Study grouping:**  **How was missing data handled?:** Missing data were not analyzed  **How were participants recruited and sampled within the study sites?:** "Participants were recruited through various methods (online consumer panel, cold calling, social media, electronic circulation lists, mailbox drops and intercept events focused around cycling) into the panel with agreeable participants then sent a URL to begin the survey."  **Methods for selecting intervention and control sites:** Intervention area: the area surrounding the newly constructed cycling path: "defined in the intervention area by postcodes in close proximity to the new bicycle path and living not more than 2.5 km away". Comparison area: "included neighbourhoods a similar distance from the central business district and with a similar demographic profile, and where the local council had no plans to modify infrastructure during the study period".  **Number of control sites:** 1  **Number of intervention sites:** 1  **Post-intervention period (start and end date or year it was conducted):** Two waves of follow-up after baseline: "Follow-up data was collected in September- October 2014 (4 months after the cycleway opened), and one year later (September-November 2015)."  **Pre-intervention time period (before intervention start):** September–October 2013 - before construction of the bicycle path  **Sample size justification and outcome used:** The sample size is only mentioned briefly in the discussion, which implies that the study was powered for a longer-term follow-up than that reported in the present study: "...the sample was determined by expected change in the primary outcomes of cycling behavior at the longer term followup."  **Study aim or objective:** "This paper assesses the short term impact of the new cycling infrastructure on awareness and use of the new infrastructure and addresses the research question of what changes in cycling behavior and perceptions of the neighbourhood were observed."  **Time points of data collection:** "Baseline data (before construction of the bicycle path) were collected on-line in September–October 2013."; "The 12-month data were collected in September–October 2014."  **Unit of allocation or exposure:** Geographical area |
| --- | --- |
| **Participants** | **Baseline Characteristics**  Cycling infrastructure arm: NR  No cycling infrastructure arm: NR  Overall:   - *Age*: 18-24: 17.6%; 25-34: 25.3%; 35-44: 25.7%; 45-55: 31.4% - *Place of residence*: - *Sex*: Male: 41.9% - *Ethnicity and language*: - *Occupation*: - *Education*: Less than tertiary: 30.4%; Tertiary or higher: 69.6% - *Socioeconomic status*: Income less than $80k: 37.3%; More than $80k: 62.7% - *Social capital*:   **Included criteria:** Apart from the geographical proximity requirement for the intervention and comparison areas, the following criteria are listed: "...if they were aged 18–55 years, had ridden a bicycle in their life and had no current disability preventing them from riding, and had sufficient English to complete the survey."  **Excluded criteria:** No further exclusion criteria reported  **Pretreatment:** "Diﬀerences between the intervention and control sample characteristics in Wave 3 included age, with both groups losing younger participants, though more so in the control sample. More households in the intervention area had children, owned a car and bicycle, and had a less skewed distribution of wealth (all p < 0.05). "  **Attrition per relevant group:** Wave 2 Int: 39.7% attrition Con: 39.3% attrition Wave 3 Int: 52.5% attrition Con: 48.9% attrition  **How were baseline differences assessed?:** "Descriptive and summary statistics were used to compare diﬀerences across the intervention and control group characteristics: Pearson's χ2 test for categorical data, or Spearman's r for ordinal outcomes, and two-sample t-tests for continuous outcomes."  **Total number completed and analysed per relevant group:** Wave 2 Int: 240 Con: 272 Wave 3 Int: 189 Con: 229  **Total number in each relevant group pre-intervention:** Int: 398 Con: 448  **Total number randomised per relevant group:** NA |
| **Interventions** | **Intervention Characteristics**  Cycling infrastructure   - *Type of intervention (e.g. infrastructure, policy or both)*: Infrastructure - *Description of main intervention*: Cycling infrastructure (2.4 km bicycle path) built by the City of Sydney as part of its expanding bicycle network - *Description of additional intervention components*: NR - *Duration of intervention*: Path completed in June 2014; permanent thereafter - *How was the intervention implemented?*: A new cycling infrastructure of 2.4km length was built by the City of Sydney as part of its expanding bicycle network in the city. - *Co-interventions*: NR - *Where was the intervention implemented?*: In George Street, Sydney (See Fig. 1) - *Resource requirements to replicate the intervention*: NR - *Economic indicators*: NR   No cycling infrastructure   - *Type of intervention (e.g. infrastructure, policy or both)*: No intervention - *Description of main intervention*: No intervention - *Description of additional intervention components*: No intervention - *Duration of intervention*: No intervention - *How was the intervention implemented?*: No intervention - *Co-interventions*: NR - *Where was the intervention implemented?*: No intervention - *Resource requirements to replicate the intervention*: No intervention - *Economic indicators*: No intervention |
| **Identification** | **Sponsorship source:** "This project is funded by an Australian Research Council Linkage Grant LP120200237."  **Country:** Australia  **Setting:** Inner-city Sydney  **Comments:**  **Author’s name:** Chris Rissel  **Institution:**  **Email:** chris.rissel@sydney.edu.au  **Address:**  **Declaration of interests stated?:** "The authors declare that they have no competing interests."  **Specify any documents relevant to this study:** Crane 2017 reports a longer and expanded follow-up; this extraction reflects both studies  **Study or programme name or acronym:** NA  **Type of record:** Journal article |
| **Notes** | Linked studies: Crane 2017(30) |

## Risk-of-bias table

| **Bias** | **Authors' judgement** | **Support for judgement** |
| --- | --- | --- |
| Random sequence generation (selection bias) | High risk | Quote: "Participants were identified as living in either the intervention or comparison area, defined in the intervention area by postcodes."  CBA study with allocation determined by proximity to intervention or control area. |
| Allocation concealment (selection bias) | High risk | CBA study; investigators were in complete control of the intervention and control site definition |
| Baseline characteristics similar | High risk | See Table 1; several potentially relevant differences between groups, including sex, age, having children, education, income. |
| Baseline outcome measurements similar | High risk | See Table 1; some differences that may be relevant for specific outcomes, including weekly cycling, MVPA, QoL and health satisfaction. |
| Blinding of participants and personnel (performance bias) | Low risk | Participants were not blinded, but unlikely that this would influence their behavior with these types of interventions |
| Blinding of outcome assessment (detection bias) | High risk | Investigators not blinded and outcomes were self reported |
| Protection against contamination | High risk | Very likely that the cycling path may have influenced the cycling behavior of those in the comparison area, although this would have led to a bias towards the null. |
| Incomplete outcome data (attrition bias) | Unclear risk | It is not reported how much missing outcome data were present or how authors dealt with this, although it is clear from Table 2 that some data were missing. |
| Selective reporting (reporting bias) | Low risk | No evidence of selective outcome reporting |
| Other bias | High risk | "those participants who were retained in the study, were more likely to be older and earn a high income, and less likely to cycle regularly or cycle to work than the baseline sample..." |

# Skov Petersen 2017

| **Methods** | **Study design:** ITS study  **Study grouping:** Parallel  **How was missing data handled?:** Satisfaction analyses: 88 of 442 were removed due to missing data.  **How were participants recruited and sampled within the study sites?:** Recruited by handing out flyers at the crossing point of the two routes. The respondents were sampled by responding to if they came via the Albertslund or Vestvolden route (study sites).  **Methods for selecting intervention and control sites:** Intervention sites had infrastructure improvements, as part of an ongoing project, while the control site did not.  **Number of control sites:** 1  **Number of intervention sites:** 2  **Post-intervention period (start and end date or year it was conducted):** Vestvolden - October 2011 Albertslund - April 2012 -  **Pre-intervention time period (before intervention start):** Vestvolden - 1858-1918 Albertslund -nr  **Sample size justification and outcome used:** nr  **Study aim or objective:** to investigate the effects of improvements made to two large,interconnected bicycle infrastructure in the western suburbs of Copenhagen, Denmark, on bicycle volumes and mode share, and cyclists' behaviour, perceptions, and experiences  **Time points of data collection:** For questionnaire data, three: baseline (May 2011); May 2012; May 2013. Counts data was running from October 2010 for 35 months  **Unit of allocation or exposure:** Individual |
| --- | --- |
| **Participants** | **Baseline Characteristics**  Intervention:   - *Age*: NR - *Place of residence*: NR - *Sex*: NR - *Ethnicity and language*: NR - *Occupation*: NR - *Education*: NR - *Socioeconomic status*: NR - *Social capital*: NR   Control   - *Age*: NR - *Place of residence*: NR - *Sex*: NR - *Ethnicity and language*: NR - *Occupation*: NR - *Education*: NR - *Socioeconomic status*: NR - *Social capital*: NR   Overall   - *Age*: NR - *Place of residence*: NR - *Sex*: NR - *Ethnicity and language*: NR - *Occupation*: NR - *Education*: NR - *Socioeconomic status*: NR - *Social capital*: NR   **Included criteria:** NR  **Excluded criteria:** NR  **Pretreatment:** Unclear; counts of control area NR  **Attrition per relevant group:** For ITS analysis: n/a. For questionnaire data: NR  **How were baseline differences assessed?:** NR  **Total number completed and analysed per relevant group:** NR  **Total number in each relevant group pre-intervention:** NR  **Total number randomised per relevant group:** N/A |
| **Interventions** | **Intervention Characteristics**  Intervention   - *Type of intervention (e.g. infrastructure, policy or both)*: Infrastructure - *Description of main intervention*: Improvements to two routes: cycle greenway: Vestvolden and a cycle highway: the Albertslund Route. - *Description of additional intervention components*: Vestvolden: "The improvements to the route include new surface and light conditions along a substantial part of the route. Furthermore, but not in focus here, the project also included a number of trial- and playgrounds intended for bicyclists (Schipperijn et al., 2015), roller skate tracks, and a range of information activities including the establishment of an information centre, the installation of signs, and the publication of leaflets, audio guides, etc. to attract leisure bicyclists." Albertslund: "The improved route was opened in April 2012 as the first of the system of ‘Cycle highways of Greater Copenhagen". "Copenhagen. The overall goal of the network is to enhance the conditions for cycle commuters through improved surfacing, connectivity, signage, lighting, etc. The plan is to construct a total of 28 routes (totalling 500 km), most of which extend from the periphery to the centre of the region. Most stretches represent improvements to existing routes and facilities, while only few are newly constructed infrastructure." - *Duration of intervention*: 2 years - *How was the intervention implemented?*: Both projects part of planned government activities. Albertslund: "The improved route was opened in April 2012 as the first of the system of ‘Cycle highways of Greater Copenhagen’ (Cycle Super Highways, 2013), which is a network of 22 municipalities in the Copenhagen region and the regional planning authority of Copenhagen." "The route along Vestvolden is part of the Copenhagen Fortification Project (2013)" - *Co-interventions*: None reported - *Where was the intervention implemented?*: City of Copenhagen: cycle greenway: Vestvolden and a cycle highway: the Albertslund Route. - *Resource requirements to replicate the intervention*: NR - *Economic indicators*: NR   Control   - *Type of intervention (e.g. infrastructure, policy or both)*: No intervention - *Description of main intervention*: N/A - *Description of additional intervention components*: N/A - *Duration of intervention*: N/A - *How was the intervention implemented?*: N/A - *Co-interventions*: None reported - *Where was the intervention implemented?*: control site at Roskildevej - *Resource requirements to replicate the intervention*: N/A - *Economic indicators*: N/A |
| **Identification** | **Sponsorship source:** "Danish Council for Strategic Research (file 09-067197), and the Copenhagen Fortification project, which was funded by the Danish Cancer Society, the Realdania Foundation, the Danish Forest & Nature Agency, and the Heritage Agency of Denmark."  **Country:** Copenhagen-Denmark  **Setting:** City  **Comments:**  **Author’s name:** Skov-Petersen Hans  **Institution:** University of Copenhagen, Dept. of Geoscience and Natural Resource Management. Denmark  **Email:** hsp@ign.ku.dk  **Address:**  **Declaration of interests stated?:** No  **Specify any documents relevant to this study:** Supplementary material: https://doi.org/10.1016/j.jtrangeo.2017.09.011  **Study or programme name or acronym:** N/A  **Type of record:** Journal article |
| **Notes** |  |

## Risk-of-bias table

| **Bias** | **Authors' judgement** | **Support for judgement** |
| --- | --- | --- |
| ITS - Was the intervention independent of other changes? | Low risk | Other changes were related to the intervention |
| ITS - Was the shape of intervention effect prespecified? | Low risk | The point of analysis is the point of the intervention |
| ITS - Was the intervention unlikely to affect data collection? | Low risk | Electronic counters on the route used before and after the changes |
| ITS - Was knowledge of the allocated interventions adequately prevented during the study | Low risk | Automatic counters on the routes used |
| ITS - Were incomplete outcome data addressed? | Low risk | No missing data |
| ITS - Was the study free from selective reporting | Low risk | All relevant outcomes reported |
| ITS - Was the study free from other bias? | Low risk | No other bias identified |

# Slater 2016

| **Methods** | **Study design:** CBA (Quasi-experimental prospective longitudinal design)  **Study grouping:** Parallel group  **How was missing data handled?:** Missing data were not considered in the analysis. "Model 2 analyses used 153 observations due to missing incivilities data at the follow-up for one park."  **How were participants recruited and sampled within the study sites?:** Participants were recruited based on geographical proximity to the control or interventions site.  **Methods for selecting intervention and control sites:** Control and interventions parks were selected based on (a) the level of community support and playground maintenance plan, (b) the age and condition of the existing playground, and (c) equitable geographic distribution of new playgrounds throughout the city (north, central, south). They considered the size and park features, close proximity and underlying neighborhood characteristics. They were then matched by neighborhood median household income and race/ethnicity.  **Number of control sites:** 1  **Number of intervention sites:** 1  **Post-intervention period (start and end date or year it was conducted):** between July and October 2014  **Pre-intervention time period (before intervention start):** between July and October 2013  **Sample size justification and outcome used:** NR  **Study aim or objective:** The aim of this study was to examine whether involvement of community groups in playground design selection, installation, and ongoing maintenance influences park utilization and moderate-to-vigorous physical activity (MVPA) post-playground renovations.  **Time points of data collection:** Baseline - July and October 201312 month follow up - July and October 2014  **Unit of allocation or exposure:** Clusters - parks |
| --- | --- |
| **Participants** | **Baseline Characteristics**  Intervention   - *Age*: NR - *Place of residence*: NR - *Sex*: NR - *Ethnicity and language*: Predominant race n (average %) : 38 (53%) - *Occupation*: NR - *Education*: NR - *Socioeconomic status*: Median household income: n (range) = 38 (18.40-121.54) - *Social capital*: NR   Control   - *Age*: NR - *Place of residence*: NR - *Sex*: NR - *Ethnicity and language*: Predominant race n (average %) : 39 (56%) - *Occupation*: NR - *Education*: NR - *Socioeconomic status*: Median household income: n (range) 39 (12.33-121.54) - *Social capital*: NR   Overall   - *Age*: NR - *Place of residence*: Thirty-one percent, 28%, and 41% of study parks were located on Chicago’s North, Central/West, and South sides, respectively - *Sex*: nr - *Ethnicity and language*: Predominant race n (average %) : 77 (55%) "Fifty-five per-cent, 23%, 16%, and 6% of study parks were located in predominantly African American, White, mixed race, and Latino neighborhoods, respectively." - *Occupation*: NR - *Education*: NR - *Socioeconomic status*: Neighborhood median household income ranged from US$12,333 to US$121,541 - *Social capital*: NR   **Included criteria:** Parks were selected taking into consideration (a) the level of community support and playground maintenance plan, (b) the age and condition of the existing playground, and (c) equitable geographic distribution of new playgrounds throughout the city (north, central, south).  **Excluded criteria:** NR  **Pretreatment:** NR  **Attrition per relevant group:** NR  **How were baseline differences assessed?:** NR  **Total number completed and analysed per relevant group:** Intervention - 48 Control - 30  **Total number in each relevant group pre-intervention:** Intervention - 39 Control - 39  **Total number randomised per relevant group:** NA |
| **Interventions** | **Intervention Characteristics**  Intervention   - *Type of intervention (e.g. infrastructure, policy or both)*: Infrastructure: renovated with community involvement - *Description of main intervention*: "Renovations involved replacing old playground equipment and ground surfacing and community engagement." - *Description of additional intervention components*: Community groups: (a) nominated their local playgrounds to be renovated in Year 1 of the program, (b) were included in the selection process of new playground equipment, (c) were involved in some of the playground installation, and (d) were asked to propose plans for ongoing playground maintenance. Community were also involved to identify how playground renovations will benefit their community and (b) collaborate with FOTP post renovation to successfully implement ongoing care and maintenance of playgrounds with the goal of enhancing playground renovations. - *Duration of intervention*: 12 months - *How was the intervention implemented?*: Parks in intervention groups underwent renovation - *Co-interventions*: NR - *Where was the intervention implemented?*: 39 parks in Chicago's 77 neighbourhoods - *Resource requirements to replicate the intervention*: The Chicago Plays initiative, a local NGO, new playground equipment, community involvement in the installation of some equipment and in making decisions about playground maintenance. - *Economic indicators*: "Chicago Park District (CPD) earmarked capital improvement fund"   Control   - *Type of intervention (e.g. infrastructure, policy or both)*: Infrastructure : not yet renovated - *Description of main intervention*: Not yet renovated and no community engagement - *Description of additional intervention components*: None - *Duration of intervention*: NA - *How was the intervention implemented?*: NA - *Co-interventions*: NR - *Where was the intervention implemented?*: NA - *Resource requirements to replicate the intervention*: NR - *Economic indicators*: NR |
| **Identification** | **Sponsorship source:** The author(s) disclosed receipt of the following financial support for the research, authorship, and/or publication of this article: This work is conducted by the Illinois Prevention Research Center and was supported by grants from UIC’s Institute for Policy and Civic Engagement and the Cooperative Agreement No. U48-DP005010, under the Health Promotion and Disease Prevention Research Centers program, funded by the Centers for Disease Control and Prevention, and administered by the Institute for Health Research and Policy at the University of Illinois at Chicago.  **Country:** USA  **Setting:** Chicago  **Comments:** N/A  **Author’s name:** Sandy Slater  **Institution:**  **Email:** "sslater@uic.edu"  **Address:**  **Declaration of interests stated?:** The author(s) declared no potential conflicts of interest with respect to the research,authorship, and/or publication of this article.  **Specify any documents relevant to this study:** NR  **Study or programme name or acronym:** NA  **Type of record:** Journal article |
| **Notes** |  |

## Risk-of-bias table

| **Bias** | **Authors' judgement** | **Support for judgement** |
| --- | --- | --- |
| Random sequence generation (selection bias) | High risk | CBA study |
| Allocation concealment | High risk | CBA study |
| Baseline characteristics similar | Low risk | Quote: "All models controlled for park size, daily outside temperature, distance between matched parks, neighborhood median household income, and neighborhood predominant race."  Intervention and control groups had similar characteristics, and of those which had imbalances, an adjustment in the analysis stage was done. |
| Baseline outcome measurements similar | Low risk | Outcomes were measured prior to the intervention, and no important differences were present across study groups. |
| Blinding of participants and personnel (performance bias) | Low risk | Blinding was not applicable in this kind of study. |
| Blinding of outcome assessment (detection bias) | Unclear risk | It is not clear if the assessors were blinded. |
| Protection against contamination | Low risk | The allocation was done by the community and there is less chance of contamination. |
| Incomplete outcome data (attrition bias) | Unclear risk | N=77 before and after the intervention. However, the authors do not prove sufficient information to assume 100% follow up. |
| Selective reporting (reporting bias) | Low risk | All relevant outcomes in the methods section are reported in the results section. |
| Other bias | Low risk | Misclassification of exposure - low risk since the tools used were standardised and were obtained from existing databases Measurement bias - low-risk: objective widely accepted tools were used Clustering - low risk: since it was appropriately adjusted for using random effects. |

# Tester 2009

| **Methods** | **Study design:** CBA study  **Study grouping:** Parallel  **How was missing data handled?:** Cross-sectional samples differed but missing/excluded data NR.  **How were participants recruited and sampled within the study sites?:** Participants visiting the intervention/control parks were observed.  **Methods for selecting intervention and control sites:** Purposefully selected. "Parks were selected for renovation based on the following criteria: condition, typical use, ability to increase field capacity with artificial turf, community value of the parks, and existing programming". "Jose Coronado Playground (Park C) was selected as the control because of similar socioeconomic and racial/ethnic demographics of nearby residents and its approximation in features (e.g. presence of playground, and soccer/baseball area) to the two intervention parks."  **Number of control sites:** 1 park/playfield  **Number of intervention sites:** 2 parks/playfields  **Post-intervention period (start and end date or year it was conducted):** Observations were conducted from May 30 to June 5, in 2006 and again in 2007.  **Pre-intervention time period (before intervention start):** Before summer 2006 (during which parks were renovated)  **Sample size justification and outcome used:** NR  **Study aim or objective:** "This study was undertaken to study the impact of a playfield renovation in two urban parks in low-income neighborhoods. Additionally, one park was part of an initiative to increase the quality of family and youth-oriented services and programs. We compare findings at baseline and one year follow-up to those from a control park."  **Time points of data collection:** "Observations were conducted from May 30 to June 5, in 2006 and again in 2007. In each target area of each park, scans were performed 8 times each day (9:00, 9:30, 12:30, 13:00, 15:30, 16:00, 18:30, and 19:00)."  **Unit of allocation or exposure:** Parks/Playfields |
| --- | --- |
| **Participants** | **Baseline Characteristics**  Intervention   - *Age*: 26.4% children/teens observed (138/523) - *Place of residence*: resource-poor neighbourhoods in San Francisco - *Sex*: 24.3 % female (396 males; 127 females observed) - *Ethnicity and language*: One park was in a primarily Latino neighborhood and the other had a mix of Latino, African-American and Asian - *Occupation*: NR - *Education*: NR - *Socioeconomic status*: Median household income of census block group: $43,333 in park A and $54,125 in park B - *Social capital*: NR   Control   - *Age*: 18.4% children/teens observed (89/483) - *Place of residence*: Resource-poor neighbourhoods in San Francisco - *Sex*: 6% female (454 males; 29 females observed) - *Ethnicity and language*: Park in primarily latino neighbourhood - *Occupation*: NR - *Education*: NR - *Socioeconomic status*: Median household income of census block group: $56,000 - *Social capital*: NR   Overall   - *Age*: NR - *Place of residence*: Resource-poor neighbourhoods in San Francisco - *Sex*: NR - *Ethnicity and language*: NR - *Occupation*: NR - *Education*: NR - *Socioeconomic status*: NR - *Social capital*: NR   **Included criteria:** "Parks were selected for renovation based on the following criteria: condition, typical use, ability to increase field capacity with artificial turf, community value of the parks, and existing programming". "Jose Coronado Playground (Park C) was selected as the control because of similar socioeconomic and racial/ethnic demographics of nearby residents and its approximation in features (e.g. presence of playground, and soccer/baseball area) to the two intervention parks."  **Excluded criteria:** NR  **Pretreatment:** NR. Median household income based on census data was higher in the control group.  **Attrition per relevant group:** n/a. Data collected by observing two cross-sectional samples at two different timepoints based on visitors to the parks.  **How were baseline differences assessed?:** NR  **Total number completed and analysed per relevant group:** Numbers differ for baseline and follow-up; two cross-sectional samples observed.Total nr of people observed in intervention parks: 3298; in control park: 585 (children, teens, adult males/females, seniors).  **Total number in each relevant group pre-intervention:** 523 people observed in intervention parks; 483 people observed in control park (children, teens, adult males/females, seniors).  **Total number randomised per relevant group:** n/a |
| **Interventions** | **Intervention Characteristics**  Intervention   - *Type of intervention (e.g. infrastructure, policy or both)*: Infrastructure - *Description of main intervention*: Park renovations - *Description of additional intervention components*: "In both parks, artificial turf replaced uneven dirt fields, and new fencing, landscaping, lighting, and picnic benches were added. In Park A, permanent soccer goals were installed, and in Park B, a walkway around the field was restored." - *Duration of intervention*: No end date; park renovated in summer of 2006 - *How was the intervention implemented?*: "In the summer of 2006, two parks in San Francisco, CA underwent significant renovations of their playfields used primarily for soccer and baseball. This was a public–private venture (approximate total cost of $5.5 million) undertaken by the City Fields Foundation and the City of San Francisco with the goal of increasing access to quality playfields for youth and families in resource-poor neighborhoods." - *Co-interventions*: "Additionally, Park B was one of 5 parks selected to be a part of the RecConnect Initiative, which is a collaboration between the San Francisco Recreation and Parks Department, the Department of Children, Youth, and Families, and neighborhood community based organizations. It is a public/private partnership designed to improve the quality of youth and family programs at public recreation centers. Some specific program components are expanded hours of park operation (e.g. playfield lights kept on during later evening hours), professional training and skills development for park and recreation program staff, and expanded programs driven by community input (e.g. dances organized by teens for teens). These program changes began at the same time as renovation in Park A." - *Where was the intervention implemented?*: Playfields in poor resource neighbourhoods in San Francisco: Garfield Square (Park A) and Silver Terrace (Park B) - *Resource requirements to replicate the intervention*: This was a public–private venture (approximate total cost of$5.5 million) undertaken by the City Fields Foundation and the City of San Francisco with the goal of increasing access to quality playfields for youth and families in resource-poor neighborhoods. - *Economic indicators*: "approximate total cost of $5.5 million"   Control   - *Type of intervention (e.g. infrastructure, policy or both)*: No intervention - *Description of main intervention*: No intervention - *Description of additional intervention components*: No intervention - *Duration of intervention*: n/a - *How was the intervention implemented?*: n/a - *Co-interventions*: None - *Where was the intervention implemented?*: Playfields in poor resource neighbourhoods in San Francisco: Jose CoronadoPlayground - *Resource requirements to replicate the intervention*: n/a - *Economic indicators*: n/a |
| **Identification** | **Sponsorship source:** "This research was made possible with funds from Team Up For Youth and from the Robert Wood Johnson Health and Society Scholars Program at UC San Francisco/UC Berkeley"  **Country:** USA  **Setting:** Urban parks in resource-poor neighbourhoods in San Francisco  **Comments:** n/a  **Author’s name:** June Tester  **Institution:** University of California, Berkeley, School of Public Health  **Email:** jtester@chori.org; junetester@post.harvard.edu  **Address:**  **Declaration of interests stated?:** Yes. "The authors declare that there are no conflicts of interest. This project was initiated and analyzed by the investigators."  **Specify any documents relevant to this study:** n/a  **Study or programme name or acronym:** n/a  **Type of record:** Journal article |
| **Notes** |  |

## Risk-of-bias table

| **Bias** | **Authors' judgement** | **Support for judgement** |
| --- | --- | --- |
| Random sequence generation (selection bias) | High risk | CBA study |
| Allocation concealment (selection bias) | High risk | CBA study the unit of allocation was geographic location. |
| Baseline characteristics similar | Unclear risk | These program changes began at the same time as renovation in Park A. Jose Coronado Playground (Park C) was selected as the control because of similar socioeconomic and racial/ethnic demographics of nearby residents and its approximation in features (e.g. presence of playground, andsoccer/baseball area) to the two intervention parks The parks seem similar but the data was not clearly reported. |
| Baseline outcome measurements similar | Unclear risk | Data NR for all park visitors as a whole. |
| Blinding of participants and personnel (performance bias) | Low risk | Unlikely to influence for this type of intervention. |
| Blinding of outcome assessment (detection bias) | High risk | "observers were not blinded to the purpose of the study, and it is possible that they were biased towards higher levels of physical activity." Furthermore, "The interobserver agreement on PA in the follow-up period was low." |
| Protection against contamination | Low risk | Allocation not at individual level. |
| Incomplete outcome data (attrition bias) | Unclear risk | Data based on observations of two cross-sectional samples at different timepoints. Missing/excluded data NR. |
| Selective reporting (reporting bias) | Unclear risk | Protocol not available. |
| Other bias | High risk | The interobserver agreement on PA in the follow-up period was low. This may have been due to a methodological problem with the reliability data itself. A smaller number of reliability assessments were collected, and they were collected during a time of particularly high park attendance. Higher numbers of individuals on the playfield make it increasingly difficult to achieve exact agreement between observers. |

# Veitch 2018

| **Methods** | **Study design:** Cluster randomized controlled trial  **Study grouping:** NR  **How was missing data handled?:** NR  **How were participants recruited and sampled within the study sites?:** "Recruitment via schools: pre-schools, primary (elementary) and secondary government and Catholic schools located within a 3km buffer surrounding the two parks were emailed and contacted by telephone and invited to participate. Recruitment via postal survey: random selection of 5000 residents (n=2500 from each park area) who lived within a 5 km buffer of the two parks were identified from the two City Councils within which the parks were located."  **Methods for selecting intervention and control sites:** The refurbishment of the intervention site presented a suitable opportunity for the study. Control site was selected based on similarities of infrastructure and amenities to the intervention site prior to refurbishment.  **Number of control sites:** N = 1  **Number of intervention sites:** N = 1  **Post-intervention period (start and end date or year it was conducted):** Follow up 1: April 2014 - May 2014 ( 2 months post intervention); Follow up 2: April 2015 - May 2015 (1 year and 2 months post intervention)  **Pre-intervention time period (before intervention start):** Baseline assessment: April 2013 - May 2013  **Sample size justification and outcome used:** Sample size calculations for the neighbourhood survey were based on ordered logistic regression analyses from a previous pilot natural experiment study in parks by comparing responses for use of control and intervention parks post-refurbishment. Outcome used: Park Use  **Study aim or objective:** 1. Examine whether park improvement increases overall park usage in the intervention park compared with the control park; 2. Examine whether park improvement increases the proportion of local residents engaging in park-based physical activity and active travel to and from the park in the intervention compared with the control park; and 3. Identify the specific aspects of the park refurbishment that attract visitors to the park and encourage park users to be more active.  **Time points of data collection:** Baseline assessment: April-May 2013 (T1); Follow up 1: April-May 2014 (T2); Follow up 2: April-May 2015.  **Unit of allocation or exposure:** Individual |
| --- | --- |
| **Participants** | **Baseline Characteristics**  Intervention   - *Age*: Child (1-12) (n, %) = 434 (18.3); Teen (13-20) (n, %) = 188 (7.9); Adult (21-59) (n, %) = 1325 (55.8); Older adult (60+) (n, %) = 427 (17.9) - *Place of residence*: NR - *Sex*: Female (n, %) = 1177 (49.6) - *Ethnicity and language*: NR - *Occupation*: NR - *Education*: NR - *Socioeconomic status*: Intercept survey only: children attending pre-schools, primary and secondary schools; amongst adults who visited the park in the past 3 months (n=294) employment status (%) full-time=36.8; part-time=27.8; unemployed=22.9 - *Social capital*: NR   Control   - *Age*: Child (1-12) (n, %) = 678 (28.5); Teen (13-20) (n, %) = 165 (6.9); Adult (21-59) (n, %) = 1217 (51.1); Older adult (60+) (n, %) = 322 (13.5) - *Place of residence*: NR - *Sex*: Female (n, %) = 1263 (53.0) - *Ethnicity and language*: NR - *Occupation*: NR - *Education*: NR - *Socioeconomic status*: Children attending pre-schools, primary and secondary schools; amongst adults who visited the park in the past 3 months (n=374) employment status (%) full-time = 33.6; part-time = 29.2 unemployed = 23.9 - *Social capital*: NR   Overall   - *Age*: NR - *Place of residence*: NR - *Sex*: NR - *Ethnicity and language*: NR - *Occupation*: NR - *Education*: NR - *Socioeconomic status*: NR - *Social capital*: NR   **Included criteria:** "As the intervention is a playspace it is important to recruit families with children aged between 2–15 years. To obtain participants with these demographic characteristics, recruitment was via two methods: 1) families with children attending pre-schools, primary and secondary schools located within 3 km of each park; and 2) a postal survey from the local City Council to households located within 5 km of each park."  **Excluded criteria:** NR  **Pretreatment:** "Despite the differences in overall size and SES, at baseline these two parks provided similar infrastructure and settings for being active, such as extensive walking/cycling paths, grassy open space areas and basic playground equipment."  **Attrition per relevant group:** NR  **How were baseline differences assessed?:** "Descriptive statistics for overall observation visitor counts, observation visitor counts in the new play-scapeat the intervention park and playground at the control park, path monitor counts, traffic counts, intercept surveys and resident surveys for the two parks at each time-point were calculated."  **Total number completed and analysed per relevant group:** Total visitor counts, intervention n = 3162 & 3157; control n = 2130 & 1654. Intercept survey, intervention n=1395 & control n=1600; Resident survey, intervention n = 550 & control n= 692  **Total number in each relevant group pre-intervention:** Intervention site: Total visitor count in the park: 2374; Total visitor count in the playscape area: 132; Path monitor counts: 1137; Traffic counts: 2336; Intercept Survey: 313; Resident survey adults: 294; Resident survey children: 180; Control site:Total visitor count in the park: 2382; Total visitor count in the play-scape area: 448: Path monitor counts: 6067; Traffic counts: 2995; Intercept Survey: 481; Resident survey adults: 374; Resident survey children: 228  **Total number randomised per relevant group:** N/A |
| **Interventions** | **Intervention Characteristics**  Intervention   - *Type of intervention (e.g. infrastructure, policy or both)*: Infrastructure - *Description of main intervention*: "...refurbishment of a large regional park (Brimbank Park)... The new equipment included a large 360 degree swing, traditional swing set, maze, rockers, sandpit, nature play area, climbing equipment, landscaping, and various sculptures and was designed to be accessible for children with disabilities. The play-scape was also designed to encourage visitors to connect with both the natural environment and the significant indigenous cultural heritage of the region with references to local flora, fauna, past farming practices and key indigenous stories throughout the play-scape." - *Description of additional intervention components*: NR - *Duration of intervention*: The park refurbishment process was conducted from September 2013 - February 2014. - *How was the intervention implemented?*: "It involved the installation of an innovative play space suitable for children of all ages and abilities." - *Co-interventions*: NR - *Where was the intervention implemented?*: "As well as being accessible by road, Brimbank Park (329 hectares) is accessible via a shared path for walking and cycling that stretches 28 km north-west from Melbourne’s central business district (CBD). Within the park is a further 4.3 km shared path circuit. The City of Brimbank has a total population of almost 190,000 residents." - *Resource requirements to replicate the intervention*: Project staff and traffic counter. - *Economic indicators*: "The refurbishment at the intervention park involved the installation of an innovative AUD$1.1 million play-scape suitable for children of all abilities that was designed by a landscape architect sourced by Parks Victoria."   Control   - *Type of intervention (e.g. infrastructure, policy or both)*: No intervention - *Description of main intervention*: The playground at the control park was an older style adventure playground which included play equipment such as: slides, swings, climbing equipment, fireman’s pole, and swing bridges. There are no planned improvements or changes to the control park during the study period. - *Description of additional intervention components*: No intervention - *Duration of intervention*: No intervention - *How was the intervention implemented?*: No intervention - *Co-interventions*: NR - *Where was the intervention implemented?*: No intervention - *Resource requirements to replicate the intervention*: No intervention - *Economic indicators*: No intervention |
| **Identification** | **Sponsorship source:** "The REVAMP project is funded by an Australian Research Council Linkage Grant (LP120200396) and includes four partners: Parks Victoria, the Victorian Health Promotion Foundation, Brimbank City Council and City West Water. JV is supported by a National Health and Medical Research Council Early Career Fellowship (ID 1053426). JS and BGC are supported by National Health and Medical Research Council Principal Research Fellowships (IDs 1026216 and #1107672 respectively). AT is supported by a Future Leader Fellowship from the National Heart Foundation of Australia (ID 100046). The contents of this manuscript are the responsibility of the authors and do not reflect the views of the funding bodies."  **Country:** Australia  **Setting:** Melbourne  **Comments:** N/A  **Author’s name:** Jenny Veitch  **Institution:** N/A  **Email:** jenny.veitch@deakin.edu.au  **Address:** N/A  **Declaration of interests stated?:** "The authors declare that they have no competing interests."  **Specify any documents relevant to this study:** Veitch 2014  **Study or programme name or acronym:** REVAMP: Recording and EValuating Activity in a Modified Park  **Type of record:** Journal Article |
| **Notes** | *Bey-Marrie Schmidt* on 07/09/2018 19:16 **Population** Unable to add columns to baseline characteristics table from my computer. |

## Risk-of-bias table

| **Bias** | **Authors' judgement** | **Support for judgement** |
| --- | --- | --- |
| Random sequence generation (selection bias) | High risk | Controlled trial; allocation not random |
| Allocation concealment (selection bias) | High risk | Controlled trial; allocation not random |
| Blinding of participants and personnel (performance bias) | Low risk | Blinding of participants and personnel was not possible, but unlikely that lack of blinding could have influenced the outcomes. |
| Blinding of outcome assessment (detection bias) | Low risk | No blinding but outcome not likely to be influenced by lack of blinding. |
| Incomplete outcome data (attrition bias) | Unclear risk | The authors mention that there is missing data, but it is unclear whether the necessary adjustments were made in the analysis. |
| Selective reporting (reporting bias) | Low risk | Judgement Comment: All relevant outcomes in the methods section are reported in the results section. |
| Other bias | Low risk | No misclassification and measurement bias. |
| Protection against contamination | Low risk | Intervention and control park were sufficiently far apart in distance to avoid contamination. |
| Baseline characteristics similar | High risk | It was not possible to find a matching large park in a disadvantaged area with similar features to the intervention park that was not undergoing any refurbishment |
| Baseline outcome measurements similar | Low risk | Similar for all baseline outcome measurements except visitor counts at the play-scape area. |

# Ward Thompson 2019

| **Methods** | **Study design:** CBA study  **Study grouping:**  **How was missing data handled?:** Missing data was input: "As 4410 out of 5460 observations had complete information (81%), imputation techniques were considered to handle missing data. Imputation was used only for data from a particular survey wave that were missing for an individual who had participated in that wave of the survey. In other words, no participant’s data were imputed other than for variables in the survey wave in which they had participated". Some data relating to the mental health outcome was excluded: "to ensure the reliability of the analysis, all data collected by the interviewers whose results for PSS were questionable needed to be excluded. The 857 problematic cases were deleted from the final sample".  **How were participants recruited and sampled within the study sites?:** "Selection used a stratified random sampling approach, stratified in accordance with distance from the WIAT intervention woodlands. This address file lists all deliverable addresses in the UK and can distinguish business and domestic addresses; we focused on domestic addresses only. Each unit postcode has a grid reference and this was used to stratify the sample by distance from the local woodland. We considered stratification by distance necessary because previous research suggests that the use of woodlands for populations living nearby may decline with distance,[30] but there is also evidence that the quality of the natural environment may moderate the effect of distance,[31] so distance was necessary to consider because the WIAT intervention is aimed at improving woodland quality. We stratified the sample in accordance with five distance points from the six WIAT-eligible woodlands. These distance points were in the range of 150m, 300m, 500m, 750m and 1500m. Letters were then sent to the selected households informing them of the research project and that participants were being sought within the communities. The letters also contained the contact details of the research team members and their office, offering participants the opportunity to receive further information about the project or to opt out (see Report Supplementary Materials 2–4). Addresses of those residents who decided to opt out were then removed from the sample. A door-to-door approach and a quadruple call-back system were used to recruit participants. Recruitment was by face-to-face request to the first adult that responded to the door-to-door approach adopted".  **Methods for selecting intervention and control sites:** We identified three pairs of sites (where each site comprised a woodland and its associated community) in central Scotland that were eligible for WIAT and were in areas within the worst 30% of deprivation in Scotland as measured by the Scottish Index of Multiple Deprivation (SIMD) [26]. The sites varied in size between a minimum of 4ha and a maximum of 24 ha. To reduce confounding, each pair of sites was matched as closely as possible by physical characteristics and by the social, economic, demographic and health characteristics of the population resident in a catchment area, defined as 1.5 km around each site [23]. Sites were assigned to the intervention arm of the study based on their readiness for management agreements between the FCS and landowners (predominantly local authorities), to ensure they would proceed and that no other interventions were planned.  **Number of control sites:** 3  **Number of intervention sites:** 3  **Post-intervention period (start and end date or year it was conducted):** Phase 1 start: October 2013 and March 2014. No end date (last cross-sectional survey from April-July 2015)  **Pre-intervention time period (before intervention start):** Baseline: late April to June 2013  **Sample size justification and outcome used:** "To answer the primary research question, our sample size needed to be large enough to (1) detect an effect of the WIAT programme in the intervention group compared with the control group at each post-intervention wave, and (2) allow us to detect a gender difference in that effect. Based on data from Stigsdotter et al.,[27] to detect a male/female difference in means of 1.2 in each group (intervention and control), with a common standard deviation (SD) of 6.2 based on a two-sided, two-sample test with a 5% level of significance and 80% power, would require a minimum of 420 males and 420 females in each arm of the study. Therefore, a total sample size of 1680, comprising 840 intervention group and 840 control group participants, was required, with an equal split of male and female participants in each group. "  **Study aim or objective:** "the study aimed to provide robust and generalisable evidence on the impact on mental health of an intervention designed to enhance, and increase engagement with, natural environments. To provide a complete assessment of the WIAT interventions, the study aimed to evaluate the effects of the interventions, the functioning of the interventions [35] and the interventions’ value for money through linked quantitative, qualitative and economic evaluations".  **Time points of data collection:** "wave 1 (baseline pre-interventions, late April to June 2013), wave 2 (follow-up, minimum of 2 months post-physical environment interventions at each site, May to June 2014) and wave 3 (follow-up, minimum of 2 months post woodland promotion interventions at each site, late April to July 2015)."  **Unit of allocation or exposure:** Communities. "Each site comprised a woodland and its associated community) in central Scotland" "areas within the worst 30% of deprivation in Scotland as measured by the Scottish Index of Multiple Deprivation (SIMD)". |
| --- | --- |
| **Participants** | **Baseline Characteristics**  Intervention   - *Age*: Proportion in age group: 16–24: 9.1%; 25–34:18.4%; 65–74: 18.5;% ≥ 75: 6.5% - *Place of residence*: Deprived urban community - *Sex*: Proportion female: 61.3 - *Ethnicity and language*: NR - *Occupation*: NR - *Education*: % in the highest level of qualification category. none: 41.4; 1: 30.6; 2: 17.4; 3: 7.0; 4: 3.7 - *Socioeconomic status*: % working: 43.4. % not coping with income: 25.0; coping with income: 53.2; living comfortably with income: 21.9. % with access to a car: 55.1. % in social class categories (I-best to V - worst) - I:2.5; II: 18.7; III: 18.7; IV: 25.0; V: 37.3 - *Social capital*: NR - *Health status*: % with disability: 13.4. % with health limited a lot: 8.8   Control   - *Age*: Proportion in age group: 16–24: 7.9%; 25–34:14.5%; 65–74: 20.2;% ≥ 75: 6.2% - *Place of residence*: Deprived urban community - *Sex*: Proportion female: 62.2 - *Ethnicity and language*: NR - *Occupation*: NR - *Education*: % in the highest level of qualification category. none: 32.8; 1: 38.0; 2: 12.5; 3: 9.2; 4: 7.5 - *Socioeconomic status*: % working: 41.5. % not coping with income: 17.8; coping with income: 54.4; living comfortably with income: 27.8. % with access to a car: 67.2. % in social class categories (I-best to V - worst) - I: 4.1; II: 21.9; III: 19.6 IV: 22.3; V: 32.1 - *Social capital*: NR - *Health status*: % with disability: 11.4. % with health limited a lot: 11.3   Overall   - *Age*: Proportion in age group: 16–24: 9.0%; 25–34: 16.4%; 65–74: 19.3;% ≥ 75: 6.4% - *Place of residence*: Deprived urban community - *Sex*: Proportion female: 61.8 - *Ethnicity and language*: NR - *Occupation*: NR - *Education*: % in the highest level of qualification category. none: 37.1; 1: 34.3; 2: 14.9; 3: 8.1; 4: 5.6 - *Socioeconomic status*: % working: 42.7. % not coping with income: 21.4; coping with income: 53.8; living comfortably with income: 24.9. % with access to a car: 61.2. % in social class categories (I-best to V - worst) - I: 3.3; II: 19.2; III: 19.1; IV: 23.7; V: 34.7 - *Social capital*: NR - *Health status*: % with disability: 12.4. % with health limited a lot: 10.0   **Included criteria:** "We identified three pairs of sites (where each site comprised a woodland and its associated community) in central Scotland that were eligible for WIAT and were in areas within the worst 30% of deprivation in Scotland as measured by the Scottish Index of Multiple Deprivation (SIMD)." "Individuals aged 16 or older, living within the intervention and control communities, within 1.5 km of the target woodland site, were eligible for the study."  **Excluded criteria:** Individuals were not eligible to participate if they primarily resided outside the study sites.Individuals recruited to the linked community-led audit and community focus group branches of the study (see Environmental audits and Community focus groups and interviews) were excluded from subsequent waves of survey data collection to avoid contamination of response by their experience of in-depth involvement in the research.  **Pretreatment:** "At baseline, panel A (see Table 8) had differences between intervention and control samples in two of the age groups and more ‘much worse than normal’ life events in the control group. There were more lower social class participants in the intervention group and more higher social class participants in the control group. The difference is more significant with regard to the educational levels of the two groups, with the intervention group having more participants with no qualifications and the control having more with higher and further educational levels. This difference is also reflected in the larger numbers finding it difficult to cope on their income in the intervention group and more in the control group living comfortably on their income. Despite attempts to match sites in accordance with physical characteristics, including woodlands, the intervention group had fewer people living close to their local woodlands and more living at a distance of ≥ 751 m than the control group. The intervention group also had lower numbers of participants with access to a car. In health terms, there were more smokers in the intervention group. The control group had more dog owners than the intervention group. There were no other significant differences between the two groups in the sample." "There were some differences in profile between panel A and panel B. As with panel A at baseline, in the panel B sample (see Table 9) many individual characteristics were not significantly different between the intervention and control groups but there were a number of significant differences. Unlike panel A, there were no differences in the age profile between intervention and control groups and the control group life events ratings included more ‘better than normal’. The pattern of significant differences for social class, educational qualifications, income coping, proximity to the local woodlands, access to a car and smoking status matched that of panel A, with the intervention group showing a disadvantaged profile in these characteristics compared with the control group. Unlike in panel A, there are also significant differences in the proportions of the panel B sample from intervention or control sites in each of the site pairs."  **Attrition per relevant group:** "Based on the random sample of addresses taken up by the survey company from the supplied postcode files, the overall response level achieved for the three surveys was 53%, lower than originally targeted." Samples for cross-sectional sample differ at each wave and are approx 30% and 13% smaller in intervention and control sites, respectively, in wave 2, and 23% and19% smaller in intervention and control sites, respectively, in wave 3. For the longitudinal cohort, attrition is 55% in the intervention group and 32% in the control group in wave 2. Attrition in wave 3 was 40% in the intervention group and 29% in the control group.  **How were baseline differences assessed?:** "To inspect the extent of imbalance in the covariates at baseline, a p-value for test of differences is also reported."  **Total number completed and analysed per relevant group:** "The repeat cross-sectional data set used for analysis from waves 2 and 3 had 1672 and 1671 cases respectively. Likewise, the number of cohort participants was 350 at wave 2 and 402 at wave 3." Intervention group: Wave 2: - 750; wave 3- 816 Control group: wave 2 - 922?; wave 3 -855  **Total number in each relevant group pre-intervention:** Cross-sectional sample of 5460 participants (wave 1, n = 2117; wave 2, n = 1672; wave 3, n = 1671),  **Total number randomised per relevant group:** n/a |
| **Interventions** | **Intervention Characteristics**  Intervention   - *Type of intervention (e.g. infrastructure, policy or both)*: Infrastructure - *Description of main intervention*: Phase 1: environmental interventions consisted of " physical changes to the woodland environment, designed to facilitate better access to, and use of, the woods. The interventions took place simultaneously across the three intervention sites over a period of 6 months, between October 2013 and March 2014. As with all WIAT schemes, the interventions were responsive to local conditions and community needs and, therefore, followed WIATprinciples but varied in the detail of their design and implementation." See specific changes to each site in pg 11 of the report. - *Description of additional intervention components*: "Phase 2 social interventions, implemented at least 4 months after the environmental interventions, not relevant for this review and thus not extracted." - *Duration of intervention*: Phase 1 started between October 2013 and March 2014. No end date (last cross-sectional survey from April-July 2015) - *How was the intervention implemented?*: "Regular progress meetings took place between the researchers and FCS staff managing the interventions to discuss plans for the interventions, progress, any problems or delays, any additional engagement with the local community and current or potential site users and any feedback on the process of intervention implementation." "Implementation of the physical interventions involved both Forest Enterprise staff and contractors to undertake the construction work, as is typical of WIAT projects. The progress meetings record that the phase 1 physical interventions were carried out as planned, without any significant divergence from the agreed programme of activities (minutes of meetings on 24 January 2014). Minor adaptations to physical interventions were responsive to local conditions and community responses; this is typical of the WIATprogramme and would be expected of any such project". - *Co-interventions*: "In the case of intervention site B, wider community developments were identified that had taken place during the course of the study and may have had an influence on outcomes." These included new developments and upgrades of paths to the edge of intervention site. "As the path upgrade by the council took place only as the final wave survey was being undertaken, it is unlikely to have affected community use of the woodlands in the previous year but may have disrupted or enhanced use during the few weeks prior to some respondents completing the survey." - *Where was the intervention implemented?*: Woodlands in deprived urban communities in Scotland - *Resource requirements to replicate the intervention*: Dedicated staff. NR. - *Economic indicators*: Cost evaluation conducted: "The total cost of the interventions was £241,667 across the estimated eligible population (n = 20,472). The average cost for the physical intervention in wave 2 was £7.68 (95% CI £7.67 to £7.69) and £11.80(95% CI £11.79 to £11.82) for both physical and social interventions in wave 3. There was no evidence of a statistically significant association between the interventions and HRQoL. An illustrative CUA in panel A reveals an incremental ICER of £935 (95% CI £399 per QALY to dominated) in wave 2 for the physical intervention (thus higher cost and lower QALY than in the control) and an ICER of £662 (95% CI £206 per QALY to dominated) in wave 3 for both social and physical interventions. The cost per QALY in panel B is £361 (95% CI £160 per QALY to dominated) for wave 2 after the physical intervention and £165 (95% CI£71 per QALY to dominated) for wave 3 after both social and physical interventions. Overall, the CCA suggests significant effects for the secondary outcomes of moderate physical activities, walking activities,connectedness to nature and social cohesion in panel A, and moderate activity and overall PA for panel B". See table 3 in Thompson 2019 journal publication.   Control   - *Type of intervention (e.g. infrastructure, policy or both)*: No intervention - *Description of main intervention*: No intervention - *Description of additional intervention components*: No intervention - *Duration of intervention*: No intervention - *How was the intervention implemented?*: No intervention - *Co-interventions*: No intervention - *Where was the intervention implemented?*: No intervention - *Resource requirements to replicate the intervention*: No intervention - *Economic indicators*: No intervention |
| **Identification** | **Sponsorship source:** "Funding: The National Institute for Health Research Public Health Research programme."  **Country:** Scotland, UK  **Setting:** Six woodland sites and associated communities, located within the Scottish Lowlands Forest District; highly urbanised and contains the majority of the most deprived populations.  **Comments:**  **Author’s name:** Catherine Ward Thompson  **Institution:**  **Email:** c.ward-thompson@ed.ac.uk  **Address:**  **Declaration of interests stated?:** Yes. "Steven Cummins and Alastair H Leyland were members of the National Institute for Health Research (NIHR) Public Health Research Funding Board at the time of application for grant funding; however, they played no role in the discussions or decision on the funding of this project. Steven Cummins is supported by a UK NIHR Senior Research Fellowship. Richard Mitchell, Alastair H Leyland and Aldo Elizalde declare that the Social and Public Health Sciences Unit, University of Glasgow, received core support from the Medical Research Council (reference numbers MC_UU_12017/10 and MC_UU_12017/13) and the Scottish Government Chief Scientist Office (reference numbers SPHSU10 and SPHSU13). Andrew Briggs reports grants from NIHR during the conduct of this study, outside the submitted work. Willings Botha received support from a Forestry Commission Scotland (FCS)-funded studentship during the course of the study, supervised by Andrew Briggs and Richard Mitchell. Catharine Ward Thompson and Richard Mitchell report grants from FCS prior to the commencement of this project and co-supervision of FCS-funded studentships that drew on the project described in the report. Catharine Ward Thompson was lead researcher (2006–11) on commissioned research funded by FCS to undertake an evaluation of some of its Woods In and Around Towns programme. Richard Mitchell is a non-remunerated director of a charity(Paths For All), which delivers, and advocates for, walking for health."  **Specify any documents relevant to this study:** Second Thompson 2019 publication: Sustainability 2019, 11, 3317; doi:10.3390/su11123317  **Study or programme name or acronym:** Woods In and Around Town (WIAT)  **Type of record:** Report |
| **Notes** |  |

## Risk-of-bias table

| **Bias** | **Authors' judgement** | **Support for judgement** |
| --- | --- | --- |
| Random sequence generation (selection bias) | High risk | No randomisation done; CBA study. |
| Allocation concealment (selection bias) | High risk | No allocation concealment done; CBA study. |
| Baseline characteristics similar | Low risk | Some baseline characteristics differed but these were adjusted for in the analyses. |
| Baseline outcome measurements similar | Unclear risk | Outcomes not measured at baseline. |
| Blinding of participants and personnel (performance bias) | Low risk | No blinding done but this is unlikely to influence the delivery of these types of interventions. |
| Blinding of outcome assessment (detection bias) | High risk | Blinding was not possible and Outcomes assessed by self-report. Knowledge of treatment allocation could have influenced self-reported outcomes. |
| Protection against contamination | Low risk | Intervention exposure is by community and contamination unlikely |
| Incomplete outcome data (attrition bias) | High risk | Overall response rate across the 3 waves was very low (aprox 50%) and samples over time were 20% or more smaller than at baseline for both groups. For the Stress outcome implausible data was excluded (14% of all data; 857/6317). |
| Selective reporting (reporting bias) | Low risk | All relevant outcomes reported |
| Other bias | Low risk | Misclassification of exposure: low risk; fieldworkers visited houses of participants; Measurement bias: low risk. validated scales used and fieldworkers trained. Incorrect analysis: n/a |

# West 2011

| **Methods** | **Study design:** CBA study  **How was missing data handled?:** Participants who did not respond to both the pre- and post-development surveys were excluded.  **How were participants recruited and sampled within the study sites?:** Before data collection, the City’s Planning Department provided researchers with a list of property owners who owned a single-family dwelling unit valued at more than $5000 and was located within 1 mile (Euclidian distance) of the greenway. Although it is not yet known what buffer size is the most appropriate for assessing walkability around homes [12] a comparison of those living within 1 mile was selected because this distance has been used previously in other physical activity research [12]. Approximately 600 property owners were randomly selected from households living within .50 miles (n = 597) of the greenway. Another 571 owners were again randomly selected from households within .51 and 1.0 miles of the greenway. Using a modified Dillman’s approach, a cover letter, survey and postage paid business reply envelope were mailed to these 1168 households during the first week of December 2007, immediately before construction on the new greenway section.  **Methods for selecting intervention and control sites:** Intervention and control sites were based on the proximity to the newly developed greenway  **Number of control sites:** n/a.  **Number of intervention sites:** n/a.  **Post-intervention period (start and end date or year it was conducted):** 2008- (11 months post-opening of the new greenway)  **Pre-intervention time period (before intervention start):** December 2007 before construction of greenways  **Sample size justification and outcome used:** NR  **Study aim or objective:** "The purpose of this study was to determine whether the development of a new greenway has the potential to increase activity levels of existing, proximate residents."  **Time points of data collection:** Baseline = 2007 11 post intervention = 2008  **Unit of allocation or exposure:** Households (residents) |
| --- | --- |
| **Participants** | **Baseline Characteristics**  Intervention (Living within .5 miles)   - *Age*: ( n = 91): 30 and under = 14.3%; 31-50 = 41.8%; 51-7- = 32.9%; Over 70 = 11. 0% - *Place of residence*: midsized Southeastern US city - *Sex*: (n=95): Male = 48.9%; Female = 51.1% - *Ethnicity and language*: (n = 91): African American = 9.9%; Caucasian = 85.7% ; Hispanic—nonwhite = 4.4% - *Occupation*: NR - *Education*: NR - *Socioeconomic status (Annual household income (per year))*: (n=87): <$15,000 = 13.8%; $15,000–$45,000 = 31% ; $45,001–$100,000 = 34.5%; >$100,00 = 20.7% ; - *Social capital*: NR   Control (Living .51–1.0 miles)   - *Age*: (n=71)30 and under= 5.6% 31–50 = 49%; 51–70= 35.6%; Over 70= 9.8% - *Place of residence*: midsized Southeastern US city - *Sex*: (n=74): Male = 45.9%; Female = 54.1% - *Ethnicity and language*: (n = 71): African American = 1.4%; Caucasian = 95.8%; Hispanic—nonwhite = 2.8% - *Occupation*: NR - *Education*: NR - *Socioeconomic status (Annual household income (per year))*: (n=67): <$15,000 = 14.9%; $15,000–$45,000 = 29.8%; $45,001–$100,000 = 41.9%; >$100,00 = 13.4%; - *Social capital*: NR   Overall   - *Age*: (n=163) % <30: 10.4, 31-50: 45.4, 51-70: 35 - *Place of residence*: NR - *Sex*: n= 169. % female: 52.4 - *Ethnicity and language*: n=163. % African American: 6.1; % caucasian: 90.2. % hispanic: 3.7 - *Occupation*: NR - *Education*: NR - *Socioeconomic status (Annual household income (per year))*: n=156. % HH income <$15,000: 14.7,; $15,000$45,001–$100,000: 32.1; –$45,000: 30.8; >$100,00: 17.3 - *Social capital*: NR   **Included criteria:** "The City’s Planning Department provided researchers with a list of property owners who owned a single-family dwelling unit valued at more than $5000 and was located within 1 mile (Euclidian distance) of the greenway." Controls: Not stated, however, "Although it is not yet known what buffer size is the most appropriate for assessing walkability around homes12 a comparison of those living within 1 mile was selected because this distance has been used previously in other physical activity research".  **Excluded criteria:** NR  **Pretreatment:** More than double of those aged 30 and under Living within .5 miles. compared to those Living .51–1.0 miles. Annual household income (per year). Imbalances between groups for >$100,00 and $45,001–$100,000.  **Attrition per relevant group:** "The initial pre-development survey was sent to 1168 residents. 368 returned a completed survey for an initial response rate of 31.5%. The post-development survey was sent to those 368 residents, of whom 166 residents returned a completed survey. This resulted in a response rate for the post-development survey of 45.1% and an overall response rate of 14.2%, reflecting those respondents from the initial sample who completed both the pre-development and post-development surveys." A small nr of participants (1 to 2) missing for some outcomes.  **How were baseline differences assessed?:** "Nonparametric statistics indicated no differences between responders and nonresponders with regard to park visitation or physical activity in the 7 days before survey data collection."  **Total number completed and analysed per relevant group:** N (total analyzed)= 169n (.5 mile) =95n (.5-1.0 miles) = 74  **Total number in each relevant group pre-intervention:** Living within .5 miles: n = 95Living .51–1.0 miles: n = 74  **Total number randomised per relevant group:** n/a |
| **Interventions** | **Intervention Characteristics**  Intervention (Living within .5 miles)   - *Type of intervention (e.g. infrastructure, policy or both)*: Infrastructure - *Description of main intervention*: Living closer to a greenway development. In early 2008, 5 miles of greenway were developed and added to an existing greenway along a river in a midsized Southeastern US city (population ~94,000). According to this city’s own website, greenways “are open-space corridors reserved for recreational use or environmental preservation that connect urban centers.” - *Description of additional intervention components*: NR - *Duration of intervention*: 11 months - *How was the intervention implemented?*: NR - *Co-interventions*: NR - *Where was the intervention implemented?*: New greenway was added along a river in a midsized Southeastern US city - *Resource requirements to replicate the intervention*: NR - *Economic indicators*: NR   Control (Living .51–1.0 miles)   - *Type of intervention (e.g. infrastructure, policy or both)*: Infrastructure - *Description of main intervention*: Living farther away from a greenway development - *Description of additional intervention components*: NR - *Duration of intervention*: 11 months - *How was the intervention implemented?*: NR - *Co-interventions*: NR - *Where was the intervention implemented?*: New greenway was added along a river in a midsized Southeastern US city - *Resource requirements to replicate the intervention*: NR - *Economic indicators*: NR |
| **Identification** | **Sponsorship source:** NR  **Country:** USA  **Setting:** Urban area  **Comments:**  **Author’s name:** Stephanie T. West  **Institution:**  **Email:** NR  **Address:**  **Declaration of interests stated?:** NR  **Specify any documents relevant to this study:** n/a  **Study or programme name or acronym:** n/a  **Type of record:** Journal Article |
| **Notes** |  |

## Risk-of-bias table

| **Bias** | **Authors' judgement** | **Support for judgement** |
| --- | --- | --- |
| Random sequence generation (selection bias) | High risk | CBA study |
| Allocation concealment (selection bias) | High risk | CBA study |
| Baseline characteristics similar | Low risk | Baseline characteristics per arm are more or less similar. |
| Baseline outcome measurements similar | Low risk | Walking, Moderate and Vigorous activities baselines measurements were similar in all three categories of activity. |
| Blinding of participants and personnel (performance bias) | Low risk |  |
| Blinding of outcome assessment (detection bias) | High risk |  |
| Protection against contamination | Low risk | Intervention contamination in these types of interventions is very unlikely. |
| Incomplete outcome data (attrition bias) | Unclear risk | The authors mention how non-response was handled, however, they don't account for the missing outcomes in these missing household/individuals. |
| Selective reporting (reporting bias) | Low risk | All relevant outcomes in the methods section are reported in the results section. |
| Other bias | Low risk | Non-respondent bias was minimized. Other biases due to clustering or measurement seem unlikely. |
