# Supplementary material for: Infrastructure, policy and regulatory interventions to increase physical activity to prevent cardiovascular diseases and diabetes: a systematic review
Source: BMC Public Health. 2023 Jan 16;23:112. doi: 10.1186/s12889-022-14841-y (PMC9841711; doi:10.1186/s12889-022-14841-y)
Supplement: Supplementary file 4 — Additional file 4. Table of studies not included in the synthesis. [file 12889_2022_14841_MOESM4_ESM.docx]

Additional file 4: Studies included in the review, but not in the evidence synthesis (n = 8)

| **Study ID** | **Study design** | **Participants (Country)** | **Intervention** | **Comparison** | **Outcomes** |
| --- | --- | --- | --- | --- | --- |
| Sarrafzadegan 2012  (linked studies: Rabiei 2010; Sarrafzadegan 2009) | Cluster RCT | Adults: Isfahan and Najaf-Abad (intervention areas) and Arak (control area), Iran | *Technology and infrastructure interventions:* modification and improvement of urban environments  *Policy and regulatory interventions:* directives on allowing time for exercise at workplace  *Co-interventions:* educating all people through campaigns and public media, as well as education of specific target groups | No intervention | Secondary outcomes:   - Prevalence of daily smoking, by gender - Percentage of individuals with different lifestyle scores before and after the intervention - Mean lifestyle score |
| Phillips 2014  (Phillips 2012) | Cluster RCT | Adults: London, United Kingdom | *Technology and infrastructure interventions:*  changes to the food supply and the neighborhood environment  *Co-interventions:* Well London project was a community engagement programme promoting healthy eating (e.g. healthy cooking classes, community food growing/feasts, food co-ops), physical activity sessions, and mental well-being in deprived neighbourhoods. | No intervention | Secondary outcome:   - Exposure to the Well London intervention   Associations between measures of exposure and trial outcomes |
| Liu 2019 | Cluster RCT | Children (7 – 11 years); Beijing, China | *Technology and infrastructure interventions:* Changes to physical environment (equipment, sports club, campaign)  *Co-interventions:*  political environment (sugar sweetened beverage ban, promotion of water consumption, improvement of school lunches, encouragement for PA), socio-cultural environment | No intervention | Primary outcomes:   - BMI - Physical activity Median (IQR) no. of days doing ± 1 hour MVPA/week |
| Bailly 2018 | CBA study | Adults above 65 years (France) | *Technology and infrastructure interventions:* improved urban area where an organized urban pedestrian circuit is present  *Co-intervention:* coaching or no coaching by a professional physical training coach | No intervention | Primary outcomes:   - Physical activity score   Secondary outcomes   - Ageing Stereotypes and Exercise - Quality of life - Physical self-esteem - Perceived health - Functional health |
| Christiansen 2014 | CBA study | Adolescents, Denmark | *Technology and infrastructure interventions:*  Physical changes to improve safety for active transport, improve cycling abilities and raise awareness of benefits  *Co-interventions:* include educating and training students in safe cycling + traffic patrol helping students cross the street | No intervention | Primary outcome:  Active school transport (x active trips out of n reported trips) |
| Gao 2013 | CBA study | Adults: Hangzhou, China | *Technology and infrastructure interventions:* structural change  *Co-interventions:*   - community mobilisation - structural change - health education   social marketing | No intervention | Secondary outcomes   - Knowledge and beliefs - Benefits of regular physical activity - Lifestyle behaviours   Potential environmental tobacco smoke |
| Kramer 2014  (Linked study: Droomers 2016) | ITS study | Adults, Netherlands | *Technology and infrastructure interventions:* green space, footpaths and cycle tracks, play grounds, sports facilities and activities  *Co-interventions*: The URBAN40 study included investments to ameliorate problems with employment, education, housing and the residential environment | No intervention | Primary outcome:   - Frequency (days per week)and duration (minutes per day) of leisure time used for physical activity (walking, cycling, sports) |
| Ickovicks 2019 | Cluster RCT | Middle school students in urban districts, USA | Support school wellness policy implementation: Support provided for implementation of nutrition policies and physical activity policies (e.g., opportunities for PA during and after school) | No intervention | Primary outcome:   - Differential changes in BMI |
