# Supplementary material for: Infrastructure, policy and regulatory interventions to increase physical activity to prevent cardiovascular diseases and diabetes: a systematic review
Source: BMC Public Health. 2023 Jan 16;23:112. doi: 10.1186/s12889-022-14841-y (PMC9841711; doi:10.1186/s12889-022-14841-y)
Supplement: Supplementary file 5 — Additional file 5. Characteristics of ongoing studies. [file 12889_2022_14841_MOESM5_ESM.docx]

Characteristics of ongoing studies

# Macmillan 2018(1)

| **Study name** | Controlled before-after intervention study of suburb-wide street changes to increase walking and cycling: Te Ara Mua-Future Streets study design |
| --- | --- |
| **Methods** | Controlled before-after intervention study |
| **Participants** | Residents aged 7 and over who live in the study areas - neighbourhoods in Auckland, New Zealand - during the study period |
| **Interventions** | Improving and widening footpaths, new pedestrian crossings, pedestrian priority across side streets, improved routes through linear parks, a fitness trail, improvements to lighting and informal surveillance, cycle lanes, way-finding, and landscaping |
| **Outcomes** | Road-user behaviour, changes to travel mode for short trips, physical activity, air quality, road traffic injuries, greenhouse gas emissions, and perceptions of neighbourhood social connection, safety, and walking and cycling infrastructure |
| **Starting date** | October 2012 to December 2021 |
| **Contact information** | Dr Alexandra Macmillan  alex.macmillan@otago.ac.nz |
| **Notes** | ISRCTN89845334 |

# Aldred 2019(2)

| Study name | Impacts of an active travel intervention with a cycling focus in a suburban context: One-year findings from an evaluation of London’s in-progress mini-Hollands programme |
| --- | --- |
| Methods | Longitudinal study t |
| Participants | Individuals sampled from households in mini-Holland boroughs (intervention sample) and from non mini-Holland Outer London boroughs (control sample). |
| Interventions | Supporting a shift from private car use to active transport modes, through creating pedestrian and cycling-friendly street environments Two subgrous: “high dose – substational changes already implemented” and “low dose neighbourhoods” – changes not yet made |
| Outcomes | Travel behaviour and attitudes to transport and the local environment |
| Starting date | 2016 |
| Contact information | Rachel Aldred ([r.aldred@westminster.ac.uk](mailto:r.aldred@westminster.ac.uk)) |
| Notes | n/a |

# Tully 2013(3)

| Study name | Physical activity and the rejuvenation of Connswater (PARC study): protocol for a natural experiment investigating the impact of urban regeneration on public health |
| --- | --- |
| Methods | Natural experiment (quasi-experimental before-and-after survey of the Greenway population; repeated cross-sectional design) |
| Participants | Households in the electoral wards part of the Greenway political constituency |
| Interventions | Connswater Community Greenway in Belfast: major urban regeneration project involving the development of a 9 km linear park, including the provision of new cycle paths and walkways. In addition to the environmental improvements, this complex intervention involves a number of programmes to promote physical activity in the regenerated area |
| Outcomes | Primary outcomes: change in proportion of individuals identified as being regularly physically active, according to the current UK recommendations |
| Starting date | unclear |
| Contact information | Frank Kee ([F.Kee@qub.ac.uk](mailto:F.Kee@qub.ac.uk)) |
| Notes | Linked to Hunter 2021 |

# Frank 2019(4)

| Study name | The Health and economic effects of light rail lines: design, methods, and protocol for a natural experiment |
| --- | --- |
| Methods | natural experiment prospectively following 3036 adults exposed to the new LRT line and a similar cohort of 4386 adults who do not live close to the new line |
| Participants | Kaiser Permanente Northwest (KPNW) members |
| Interventions | new light rail transit (LRT) line in Portland, Oregon |
| Outcomes | Blood pressure, body mass index, lipids, glycosylated hemoglobin, and health care utilization and costs, travel patterns, physical activity (PA), and perceived neighborhood walkability. |
| Starting date | 2015 |
| Contact information | Stephen P. Fortmann ([Stephen.P.Fortmann@kpchr.org](mailto:Stephen.P.Fortmann@kpchr.org)) |
| Notes | n/a |

# Winters 2018(5)

| Study name | Impacts of Bicycle Infrastructure in Mid-Sized Cities (IBIMS): protocol for a natural experiment study in three Canadian cities |
| --- | --- |
| Methods | Natural experiment (one intervention city and two comparison cities) |
| Participants | Adults (>18 years) living in Victoria, Bc, Kelowa, BC; and Halifax, Nova Scotia in Canada |
| Interventions | City of Victoria’s AAA bicycle network investment; buidling of a connected network of infrastructure that separates bicycles from motor vehicles, designed to attract people of ‘all ages and abilities’ to bicycling |
| Outcomes | The primary outcome is the proportion of people reporting bicycling. Secondary outcomes are perceived safety and bicycle safety incidents. Spatial analyses will compare the distribution of bicycle infrastructure and bicycle safety incidents across neighbourhoods and across time. We will also calculate the economic benefits of bicycling using WHO’s Health Economic Assessment Tool. |
| Starting date | 2016 |
| Contact information | Dr Meghan Winters; [mwinters@sfu.ca](mailto:mwinters@sfu.ca) |
| Notes | n/a |

# Hobin 2020(6)

| Study name | Physical activity trails in an urban setting and cardiovascular disease morbidity and mortality in Winnipeg, Manitoba, Canada: a study protocol for a natural experiment |
| --- | --- |
| Methods | Interrupted time series analysis using population-based health data with census and environmental data |
| Participants | Individuals 30–65 years of age residing within 400–1200 m of the trail |
| Interventions | Newly built urban trail that was part of a large policy/infrastructure investment from the city/province to enhance the built environment for active transport and recreational physical activity in the city of Winnipeg, Manitoba Canada between 2010 and 2012. Four, paved, multiuse (eg, cycling, walking and running), two-lane trails that are 5–8km long built, which span ~60 neighbourhoods. |
| Outcomes | Primary outcome: composite measure of incident major adverse CVD events (ie, CVD-related mortality, ischaemic heart disease, stroke and congestive heart failure). Secondary outcome: composite measure of incident CVD-related risk factors (ie, diabetes, hypertension and dyslipidaemia). |
| Starting date | 2018 |
| Contact information | Dr Jonathan McGavock; [jmcgavock@chrim.ca](mailto:jmcgavock@chrim.ca) |
| Notes | ClinicalTrials.gov Identifier: NCT04057417 |

# Kestens 2019(7)

| Study name | INTERACT: A comprehensive approach to assess urban form interventions through natural experiments |
| --- | --- |
| Methods | Natural experiment |
| Participants | Adults 18 years or older living in each city |
| Interventions | INTERACT will evaluate natural experiments in four Canadian cities: the Arbutus Greenway in Vancouver, British Columbia; the All Ages and Abilities Cycling Network in Victoria, BC; a new Bus Rapid Transit system in Saskatoon, Saskatchewan; and components of the Sustainable Development Plan 2016–2020 in Montreal, Quebec |
| Outcomes | Transportation mode use and physical activity |
| Starting date | 2017 |
| Contact information | Yan Kestens; [yan.kestens@umontreal.ca](mailto:yan.kestens@umontreal.ca) |
| Notes | n/a |

# Astell-Burt 2016(8)

| **Study name** | Large-scale investment in green space as an intervention for physical activity, mental and cardiometabolic health: study protocol for a quasi-experimental evaluation of a natural experiment |
| --- | --- |
| **Methods** | Quasi-experimental design |
| **Participants** | participants in the 45 and Up Study who resided up to 5 km Euclidean distance (as the crow flies) from any point of the Western Sydney Parklands |
| **Interventions** | Largescale investment in approximately 5280 hectares of green space stretching 27 km north to south in Western Sydney, Australia including public access points, advertising billboards, walking and cycle tracks, BBQ stations, and children’s playgrounds |
| **Outcomes** | Psychological wellbeing, physical functioning, BMI and overweight/obesity, incidence of doctor-diagnosed cardiometabolic diseases such as hypertension, cardiovascular disease and diabetes |
| **Starting date** | 2006 |
| **Contact information** | Thomas Astell-Burt; thomasab@uow.edu.au |
| **Notes** | n/a |

# Benton 2018(9)

| **Study name** | Evaluating the Impact of Improvements in Urban Green Space on Older Adults' Physical Activity and Wellbeing: a Natural Experimental Study (GHIA) |
| --- | --- |
| **Methods** | Observational prospective study |
| **Participants** | Participants visiting intervention sites |
| **Interventions** | Urban street greening |
| **Outcomes** | Take notice behavior, overall count of older adults, sedentary behavior, walking behavior, vigorous physical activity, connect behavior |
| **Starting date** | September 4, 2017 |
| **Contact information** | Jack Benton, University of Manchester |
| **Notes** | [https://ClinicalTrials.gov/show/NCT03575923](https://clinicaltrials.gov/show/NCT03575923) |

# NCT01711138(10)

| **Study name** | An Intervention Study to Assess the Role of the Built Environment on Physical Activity and Active Transportation to School in Children and Youth (ASAP-Jr) |
| --- | --- |
| **Methods** | Case-control study |
| **Participants** | Student is in grade 4 - 10 at start of study (fall 2012), or grade 4 at any of the subsequent study years (2013-2015). |
| **Interventions** | Neighborhood development |
| **Outcomes** | Objectively measured physical activity overall (minutes of moderate-to-vigorous physical activity per day), Objectively measured physical activity during commuting to and from school (minutes of moderate-to-vigorous physical activity during commuting) |
| **Starting date** | October 2012 |
| **Contact information** | Christine Voss; [christine.voss@hiphealth.ca](mailto:christine.voss@hiphealth.ca) |
| **Notes** | [https://ClinicalTrials.gov/show/NCT01711138](https://clinicaltrials.gov/show/NCT01711138) |

# NCT03946241(11)

| **Study name** | Physical Activity in Schools After the Reform (PHASAR) |
| --- | --- |
| **Methods** | Ecologic study |
| **Participants** | Pre-reform studies: School children in 1st to 9th grade (ages between 6 and 17 years). The participating children's parents or legal guardians.  Post-reform studies: School children in 1st to 9th grade (ages between 6 and 17 years). The participating children's parents or legal guardians. Teachers, pedagogues, and headmasters from the same schools |
| **Interventions** | Wide-ranging school reform for all public schools in Denmark. The reform involved changes in several aspects of the school structure and content. In a physical activity promotion perspective, a distinctive feature of the school reform is that it has become mandatory to integrate an average of 45 minutes of daily physical activity during the regular school day. |
| **Outcomes** | Change in levels of school time physical activity, Change in school children's BMI from the majority of all Danish public schools, Changes in waist-to-height ratio among school children, Change in time spent in moderate to vigorous physical activity in school children during school time, leisure time and total time, total amount of physical activity in school children during school time, leisure time and total time |
| **Starting date** | August 22, 2017 |
| **Contact information** | Anders Grøntved, University of Southern Denmark |
| **Notes** | [https://ClinicalTrials.gov/show/NCT03946241](https://clinicaltrials.gov/show/NCT03946241) |

# NCT04569578(12)

| **Study name** | Increasing Children's Physical Activity by Policy (CAP) |
| --- | --- |
| **Methods** | Randomised Controlled Trials |
| **Participants** | Children and teachers at selected schools |
| **Interventions** | Formalized physical activity policies implemented in preschools, and a study website with an activity bank also serving as a platform for inter-professional education that preschool teachers can share and communicate about engaging activities and experience |
| **Outcomes** | For children and teachers: Change in physical activity levels, Change in sedentary time, Change in musculoskeletal fitness, Change in anthropometry (weight in kg, height in cm, waist circumference in cm, BMI), Change in psychosocial functioning, Change in sleep, Sick leave duration and frequency, Change in compliance with the World Health Organization (WHO) guidelines on physical activity, sedentary behaviour and sleep |
| **Starting date** | September 23, 2020 |
| **Contact information** | Daniel Berglind, Karolinska Institutet |
| **Notes** | [https://ClinicalTrials.gov/show/NCT04569578](https://clinicaltrials.gov/show/NCT04569578) |

# NCT04507100(13)

| **Study name** | Comprehensive Analysis of the Program:Salud Escolar |
| --- | --- |
| **Methods** | Clinical Trial |
| **Participants** | School children 6-12 years old in Mexico city |
| **Interventions** | World Health Organization (WHO) health promoting framework to foster a healthy environment and enhance children's physical activity, healthy eating and hydration. Salud Escolar consists on several activities aligned in the following components: healthy eating, healthy hydration, physical activity and additional cross sectional strategies |
| **Outcomes** | Physical activity, water consumption, vegetable intake, fruit intake, self-reported knowledge and attitudes, perceived behavioral control, junk food intake |
| **Starting date** | February 17, 2020 |
| **Contact information** | Alejandra Jáuregui, National Institute of public Health |
| **Notes** | [https://ClinicalTrials.gov/show/NCT04507100](https://clinicaltrials.gov/show/NCT04507100) |

# REFERENCES

1. Macmillan AK, Mackie H, Hosking JE, Witten K, Smith M, Field A, et al. Controlled before-after intervention study of suburb-wide street changes to increase walking and cycling: Te Ara Mua-Future Streets study design. Bmc Public Health. 2018;18.

2. Aldred R, Croft J, Goodman A. Impacts of an active travel intervention with a cycling focus in a suburban context: One-year findings from an evaluation of London's in-progress mini-Hollands programme. Transportation Research Part a-Policy and Practice. 2019;123:147-69.

3. Tully MA, Hunter RF, McAneney H, Cupples ME, Donnelly M, Ellis G, et al. Physical activity and the rejuvenation of Connswater (PARC study): protocol for a natural experiment investigating the impact of urban regeneration on public health. BMC public health. 2013;13:774.

4. Frank LD, Kuntz JL, Chapman JE, Fox EH, Dickerson JF, Meenan RT, et al. The Health and economic effects of light rail lines: design, methods, and protocol for a natural experiment. BMC public health. 2019;19(1):200.

5. Winters M, Branion-Calles M, Therrien S, Fuller D, Gauvin L, Whitehurst DGT, et al. Impacts of Bicycle Infrastructure in Mid-Sized Cities (IBIMS): protocol for a natural experiment study in three Canadian cities. BMJ open. 2018;8(1):e019130.

6. Hobin E, Swanson A, Booth G, Russell K, Rosella LC, Smith BT, et al. Physical activity trails in an urban setting and cardiovascular disease morbidity and mortality in Winnipeg, Manitoba, Canada: a study protocol for a natural experiment. BMJ open. 2020;10(2):e036602.

7. Kestens Y, Winters M, Fuller D, Bell S, Berscheid J, Brondeel R, et al. INTERACT: A comprehensive approach to assess urban form interventions through natural experiments. BMC public health. 2019;19(1):51.

8. Astell-Burt T, Feng XQ, Kolt GS. Large-scale investment in green space as an intervention for physical activity, mental and cardiometabolic health: study protocol for a quasi-experimental evaluation of a natural experiment. Bmj Open. 2016;6(4):9.

9. Benton JS, Anderson J, Cotterill S, Dennis M, Lindley SJ, French DP. Evaluating the impact of improvements in urban green space on older adults' physical activity and wellbeing: protocol for a natural experimental study. BMC public health. 2018;18(1):923.

10. Columbia UoB, Vancouver Co, Board VS, Sporta J. An Intervention Study to Assess the Role of the Built Environment on Physical Activity and Active Transportation to School in Children and Youth. <https://ClinicalTrials.gov/show/NCT01711138>; 2012.

11. Denmark UoS, TrygFonden D, Cambridge Uo. Physical Activity in Schools After the Reform. <https://ClinicalTrials.gov/show/NCT03946241>; 2017.

12. Institutet K, Council SC. Increasing Children's Physical Activity by Policy (CAP). <https://ClinicalTrials.gov/show/NCT04569578>; 2020.

13. Instituto Nacional de Salud Publica M, UNICEF, Ministry of Education M, Ministry of Health M. Comprehensive Analysis of the Program:Salud Escolar. <https://ClinicalTrials.gov/show/NCT04507100>; 2020.
