# Supplementary material for: Infrastructure, policy and regulatory interventions to increase physical activity to prevent cardiovascular diseases and diabetes: a systematic review
Source: BMC Public Health. 2023 Jan 16;23:112. doi: 10.1186/s12889-022-14841-y (PMC9841711; doi:10.1186/s12889-022-14841-y)
Supplement: Supplementary file 6 — Additional file 6. Characteristics of studies awaiting classification. [file 12889_2022_14841_MOESM6_ESM.docx]

Characteristics of studies awaiting classification

# Thompson 2014(1)

| **Methods** | Pre-post cross sectional survey + cohort study post intervention |
| --- | --- |
| **Participants** | Residents aged 65 or older living on the intervention or comparison streets |
| **Interventions** | Sustrans ‘DIY streets’ |
| **Outcomes** | Primary : general health(EQ-5D), quality of life ( CASP-19)  Secondary : frequency of outdoor visits, typical time spent outdoors |
| **Notes** | n/a |

# NSW Department of Health 2002(2)

| **Methods** | n/a |
| --- | --- |
| **Participants** | n/a |
| **Interventions** | Park modifications |
| **Outcomes** | Physical activity |
| **Notes** | This publication could not be accessed |

# Reger-Nash 2005(3)

| **Methods** | n/a |
| --- | --- |
| **Participants** | n/a |
| **Interventions** | Marketing to promote walking |
| **Outcomes** | n/a |
| **Notes** | No record of this publication was identified |

# Simon 2011(4)

| **Methods** | Multilevel randomized study |
| --- | --- |
| **Participants** | Adolescents |
| **Interventions** | Intervention Centred on Adolescents’ Physical Activity and Sedentary Behaviour (ICAPS) - multilevel program to enhance physical activity integrating environmental changes |
| **Outcomes** | Measured body mass index (BMI) and waist circumference (W), reported physical activity (PA) and sedentary behaviours (MAQ questionnaire) |
| **Notes** | From conference abstract |

# Giles-corti 2019(5)

| **Methods** | Natural experiment + longitudinal data collection at four time points |
| --- | --- |
| **Participants** | Residents in the Perth metropolitan region |
| **Interventions** | Greenfield developments |
| **Outcomes** | Built-environment measures including access to public open space,shops and public transport; provision of footpaths, street connectivity, land-use mix, residential density and (standardised) neighbourhood walkability measures |
| **Notes** | Article within a book. The article is a literature review which includes a summary of the RESIDE study. |

# Curtis 2010(6)

| **Methods** | Quasi-longitudinal study |
| --- | --- |
| **Participants** | Residents in Southern Perth, Western Australia |
| **Interventions** | Transit oriented development ( new railway stations and associated configurations e.g shops, services ) |
| **Outcomes** | Change in travel behavior ( reduction in motorized travel, substitution to public transport, walking, cycling) |
| **Notes** | Conference abstract |

# Greiser 2013(7)

| **Methods** | community-based interventional study with longitudinal data collection |
| --- | --- |
| **Participants** | Men and women 25 - 69 years |
| **Interventions** | Education program including print media, screenings, events and environmental programs |
| **Outcomes** | cardiovascular mortality |
| **Notes** | Abstract |

# REFERENCES

1. Thompson CW, Curl A, Aspinall P, Alves S, Zuin A. Do changes to the local street environment alter behaviour and quality of life of older adults? the 'DIY Streets' intervention. British Journal of Sports Medicine. 2014;48(13):1059-65.

2. Health NSWDo. The Effect of Park Modifications and Promotion on Physical Activity Participation. Summary Report. 2002;vol. vi.

3. Reger-Nash B, Cooper L, Orren J, Cook D. Marketing used to promote walking in McDowell County. The West Virginia medical journal. 2005;101(3):106-.

4. Simon C, Schweitzer B, Drai J, Kellou N, Sandalinas F, Reitzer C, et al. Overweight prevention in adolescents by increasing physical activity: 6-year results of the randomized ICAPS study. Obesity Reviews. 2011;12(SUPPL. 1):26.

5. Giles-Corti B, Gunn L, Hooper P. Built environment and physical activity. Integrating Human Health into Urban and Transport Planning. 2019.

6. Curtis C, Olaru D. The impacts of a new railway: Travel behaviour of residents in new station precincts. 12th World Transport Research Conference. 2010:25.

7. Greiser EM. Risk factor trends and cardiovascular mortality risk after 3.5 years of community-based intervention in the German Cardiovascular Prevention Study. Annals of Epidemiology. 1993;3(5 SUPPL.):S13-S27.
