# Supplementary material for: Infrastructure, policy and regulatory interventions to increase physical activity to prevent cardiovascular diseases and diabetes: a systematic review
Source: BMC Public Health. 2023 Jan 16;23:112. doi: 10.1186/s12889-022-14841-y (PMC9841711; doi:10.1186/s12889-022-14841-y)
Supplement: Supplementary file 7 — Additional file 7. Detailed Risk of bias assessment. [file 12889_2022_14841_MOESM7_ESM.docx]

**Risk of bias in included studies**

Risk of bias assessments of all trials and CBA studies included are depicted in Figure 3, and of all ITS studies in Figure 4.

Risk of bias in included studies with a comparison group (n=29)

*Sequence generation and allocation concealment (selection bias):* The one included cluster trial (Veitch et al., 2018) was classified at high risk of selection bias as it was a controlled trial and the allocation to study arms was not random. All CBA studies (n=28) were classified at high risk of selection bias due to the non-random nature of the study design.

**Figure 3. Risk of bias of included trials and CBA studies (n=29)**

*Similarity in baseline characteristics (selection bias):* Four studies (Veitch et al., 2018, Cortinez-O'Ryan et al., 2017, Rissel et al., 2015, Richardson et al., 2020) were at high risk of selection bias due to differences in park or participant characteristics at baseline that were not fully adjusted for in the analysis. Fifteen studies were at low risk of bias as they reported no imbalance of baseline characteristic, or adequately adjusted for any imbalance in the analysis. The remaining 10 studies were at unclear risk of bias; for these the baseline characteristics were not reported at all or were not reported in sufficient detail to enable a judgment.

*Similarity of outcomes at baseline (selection bias):* Six studies (Rissel et al., 2015, Prins et al., 2017, Richardson et al., 2020, Kubota et al., 2019, Dill et al., 2014, Bohn-Goldbaum et al., 2013) were at high risk of selection bias due to an imbalance of outcomes at baseline that were not adjusted for in the analysis.  Fourteen studies were at low risk of bias as they did not report any differences at baseline or, if there were any, these were adjusted for in the analyses. Nine studies were at unclear risk of bias; these either did not report the outcomes at baseline or the information was not clearly reported.

*Blinding of participants and personnel (performance bias):* All studies were judged at low risk of performance bias (n=29). In general blinding in these studies is not possible however, due to the ecological nature of the interventions, performance bias is unlikely.

*Blinding of outcome assessors (detection bias):* Eighteen studies were at high risk of detection bias; blinding was not possible or not reported and the outcomes were self-reported and thus more prone to influence from lack of blinding. Eight studies were at low risk of bias as outcomes were collected through routinely collected data and using  objective measures (e.g., actigraph) and thus were unlikely to be influenced by lack of blinding. Three studies were at unclear risk of detection bias(McDonald et al., 2013, D'Haese et al., 2015, Slater et al., 2016).

*Protection against contamination:* Six studies were at high risk of bias; these studies either reported contamination or the study groups were determined by distance from  the intervention site, which made contamination likely. Seventeen studies were at low risk of bias as sites were different geographic areas and thus contamination was unlikely. Six studies were at unclear risk of bias due to insufficient information reported.

*Incomplete outcome data (attrition bias):* 10 studies were at high risk of bias as they reported high levels of attrition (>10%) or very low response rates which differed between study groups. Five studies were at low risk of attrition bias; no missing data was reported or it was similar between the groups (Quigg et al., 2012, D'Haese et al., 2015, Pazin et al., 2016, Hirsch et al., 2017, Green et al., 2014a). Fourteen studies were at unclear risk of attrition bias.

*Selective reporting (reporting bias):* Twenty-four studies were at low risk of bias; these studies reported, in the results sections, the same outcomes and analysis reported in the methods section or the protocol. Five studies were at unclear risk of reporting bias as the methods were not clearly reported and no protocol was available.

*Other potential sources of bias:* Eight studies were at high risk due to measurement bias and 16 were at low risk of bias as no other potential bias was identified. Five studies were at unclear risk of other bias due to potential misclassification and measurement bias.

Risk of bias in ITS studies (n=4)

*Intervention independent of other changes*: One study was at high risk of bias due to the high likelihood of many other changes influencing the outcome during the study period (Branas et al., 2011). One study (Skov-Petersen et al., 2017) was at low risk of bias and two studies (Higgerson et al., 2018, Grunseit et al., 2019) were at unclear risk of bias due to insufficient information being reported.

**Figure 4. Risk of bias of included ITS studies (n=4)**

*Shape of intervention effect prespecified*:  Two studies (Higgerson et al., 2018, Skov-Petersen et al., 2017) were at low risk of bias as the point of analysis was the point of the intervention. Two studies (Grunseit et al., 2019, Branas et al., 2011) were at unclear risk of bias.

*Intervention unlikely to affect data collection*: All four studies were at low risk of bias as data collection was not influenced by the intervention and was collected in the same way before and after the intervention.

*Knowledge of the allocated interventions*: All four studies were at low risk of bias; the outcomes assessed were objective and collected using routinely collected data or automatic counters.

*Incomplete outcome data addressed*: Two studies (Grunseit et al., 2019, Skov-Petersen et al., 2017) were at low risk of attrition bias; the data was collected using automatic counters and thus missing data was unlikely or no missing data was reported. Two studies (Higgerson et al., 2018, Branas et al., 2011) were at unclear risk of bias.

*Selective reporting and other bias:* All studies were judged at low risk of bias from selective reporting and from other bias; all relevant methods and outcomes were reported and no other bias was identified.
