# Supplementary material for: Infrastructure, policy and regulatory interventions to increase physical activity to prevent cardiovascular diseases and diabetes: a systematic review
Source: BMC Public Health. 2023 Jan 16;23:112. doi: 10.1186/s12889-022-14841-y (PMC9841711; doi:10.1186/s12889-022-14841-y)
Supplement: Supplementary file 8 — Additional file 8. Individual study results table. [file 12889_2022_14841_MOESM8_ESM.docx]

# 1. Interventions addressing technology and infrastructure (n = 18)

## 1.1 Green or other spaces

| **Study ID**  **Study design**  **(Country)** | **Comparison** | **Participants at baseline (n)*** | **Outcome measure reported** | **Intervention** | | **Control** | | **Effect measure reported** | **Effect direction** | **Time of outcome measure** |  |
| --- | --- | --- | --- | --- | --- | --- | --- | --- | --- | --- | --- |
|  |  |  |  | **Baseline value** | **Follow-up** | **Baseline value** | **Follow-up** |  |  |  |  |
| **Outcome: Physical activity** | | | | | | | | | | | |
| 1. Veitch 2018 (cRCT)  (Australia)  *also reports vigorous, sedentary, etc levels of PA. outcomes not extracted | Park refurbishment vs no infrastructure changes | Total visitor counts: 4756 ( intervention park: 2374; control park: 2382 | Proportion engaging in MVPA at the park | n (%)789 (33.2) | n (%)1057 (33.4) | n (%) 1028 (43.2) | n (%) 742 (34.8) | IRR 2.19 (95% CI 1.14 to 4.20, p = 0.019) | ▲ | 1 year |  |
|  |  |  | Proportion engaging in MVPA at the park | n (%) 789 (33.2) | n (%) 907 (28.7) | n (%) 1028 (43.2) | n (%) 583 (35.2) | IRR 2.28 (95% CI 1.19 to 4.38, p = 0.013) | ▲ | 2 years |  |
|  |  |  | Park visits | mean (SE) 32.5 ± 5.1 | mean (SE) 41.6 ± 6.3 | mean (SE) 32.6 ± 3.9 | mean (SE) 28.0 ± 4.8 | NR |  | 1 year |  |
|  |  |  | Park visits | mean (SE) 32.5 ± 5.1 | mean (SE) 41.5 ± 6.4 | mean (SE) 32.6 ± 3.9 | mean (SE) 21.8 ± 2.9 | NR |  | 2 years |  |
| 2. Cortinez O'Ryan 2017 (CBA)  (Chile) | Neighbourhood with street closed for play vs control neighbourhood | 100 children (intervention neighbourhood: 51, control neighbourhoods: 49) | Meeting pedometer-derived physical activity guidelines | 27.5% | 52.8%; | 49% | 53% | Change in IG: 25.3%; Change in CG: 4.0%; Between group comparison: p>0.05  *Significant increase in intervention sites and non-significant increase in control sites* | △ | 3 months |  |
|  |  |  | Number of weekdays with outside play (median) | Median (IQR): 2 (5) | Median (IQR): 3 (3)" | Median (IQR): 3 (5) | Median (IQR): 5 (5)" | NR (p>0.05) |  | 3 months |  |
|  |  |  | After-school outdoor playtime median (min/day) | Median (IQR): 60 (120) | Median (IQR): 90 (60) | Median (IQR): 60 (120) | Median (IQR): 60 (90) | NR (p>0.05) |  | 3 months |  |
|  |  |  | Weekly after-school outdoor playtime median (min/weekday) | Median (IQR): 120 (480) | Median (IQR): 300 (480) | Median (IQR): 150 (600) | Median (IQR): 300 (600) | NR (p>0.05) |  | 3 months |  |
|  |  |  | General weekdays median (steps/day Monday to Sunday) | Median (IQR): 10168 (3797) | Median (IQR): 12824 (8561) | Median (IQR): 12107 (5405) | Median (IQR): 13196 (6071) | NR (p>0.05) |  | 3 months |  |
|  |  |  | Intervention days median (steps/day on Wednesday and Friday) | Median (IQR): 13215 (6836) | Median (IQR): 14124 (12772) | Median (IQR): 12613 (6301) | Median (IQR): 12165 (7388) | NR (p>0.05) |  | 3 months |  |
|  |  |  | During intervention hours (s steps/day on Wednesday and Friday from 18h00 to 21h00) | Median (IQR): 2090 (2262) | Median (IQR): 4249 (4942) | Median (IQR): 2347 (1746) | Median (IQR): 2911 (2176) | NR (p>0.05) |  | 3 months |  |
| 3. D’Haese 2015  CBA study  (Belgium) | Play streets vs no intervention | 167 children (Playstreet: 71; control: 96) | MVPA (minutes/day) | 54.92 (24.94) | 67.05 (38.00) | 57.41 (33.68) | " 52.87 (27.98) | Regression coefficient, 0.854, 95% CI: 0.204 to 1.504, SE = 0.332, p-value = 0.01. | ▲ | 1 week |  |
|  |  |  | Sedentary time (minutes/day) | 367.46Min/day (110.72) | 336.69 (72.92) | 381.50 (92.39) | 400.33 (94.43) | Regression coefficient = -0.616, SE = 0.246, p-value = 0.012 |  | 1 week |  |
| 4. Quigg 2012  CBA study  (New Zealand) | Playground upgrade vs no intervention | 184 children (intervention: 96; control: 88) | Total daily PA (total daily accelerometer counts/child day) | NR | NR | NR | NR | Ratio of geometric means = 1.11; 95% CI 0.85 to 1.44, p-value = 0.456 | △ | 12 months |  |
| 5. Ward Thompson 2019  CBA study  (Scotland) | Physical changes to the woodland environment to facilitate access to and use of the woods vs no intervention | Cross-sectional sample of 5460 participants (wave 1, n = 2117; wave 2, n = 1672) | vigorous activity (MET-minutes per week) | NR | NR | NR | NR | b=–152.9  95% CI (–422.6 to 116.8) | ▽ | 2 months |  |
|  |  |  | moderate activity (MET-minutes per week) | NR | NR | NR | NR | b=–215.40  95% CI (–409.40 to –21.39)  p<0.01 | ▼ | 2 months |  |
|  |  |  | walking activity (MET-minutes per week) | NR | NR | NR | NR | b=203.3  95% CI 36.81 to 369.8  p<0.01 | ▲ | 2 months |  |
|  |  |  | Overall PA (MET-minutes per week) | NR | NR | NR | NR | b=–282.4  95% CI –732.1 to 167.3 | ▽ | 2 months |  |
| 6. Richardson 2020  CBA study  (USA) | Public housing development and greenspace landscaping, including changing the streetscape surrounding the developments, providing improved aesthetics (e.g. trees, grass) and walkability (e.g. sidewalks, street crossings). Renovation of current greenspace, including multiple parks, six outdoor stairwells, and three trails connecting parks **vs.**  Fewer investments, exclusively related to housing | 17 parks (8 intervention, 9 control)  Participants: 673 in intervention, 330 in control | Number of park users/hour (Richardson 2020) | 8.8 (4.9) | 6.3 (2.2) | 7.8 (3.0) | 3.2 (0.6) | DID=2.4  p=0.3 | △ | 3 years |  |
|  |  |  | Number of park users in Sedentary activity (e.g. sitting)/hour (Richardson 2020) | 5.4 (4.2) | 3.9 (1.8) | 4.9 (2.0) | 1.7 (0.3) | DID=2.1  p=0.22 | ▽ | 3 years |  |
|  |  |  | Number of park users in moderate to vigourous activity/hour (Richardson 2020) | 3.3 (1.0) | 1.6 (0.4) | 3.5 (0.9) | 2.2 (0.5) | DID=− 0.5  p=0.54 | ▽ | 3 years |  |
|  |  |  | MVPA (minutes /day) (Dubowitz 2019) [accelerometer data] | Mean (SE) 6.89 (0.90) | Mean 6.06 | Mean (SE) 6.18 (1.22) | Mean 5.12 | DID: 0.24  p = 0.813 | △ | 3 years |  |
|  |  |  | Active transport – average time spent in active transport in the past 7 days (min/week) (Dubowitz 2019) [self-reported data] | Mean (SE) 197.74 (15.02) | Mean 177.52 | Mean (SE) 201.93 (25.71) | Mean 85.75 | DID: 36.40  p = 0.270 | △ | 3 years |  |
| 7. Bohn-Goldbaum 2013  CBA study  (Australia) | Upgrade of playgrounds in a park **vs.**  Parks not renovated/with similar pre-renovation playgrounds as intervention park | NR | Children playground usage ("mean number of children per 2-hour observation period." | mean (SD?) 4.50 (5.03) | mean (SD?) 4.98 (6.05) | mean (SD?) 8.52 (9.99) | mean (SD?) 6.69 (6.64) | “there was no detectable difference between the parks at follow-up (interaction between park and time: 𝑃 = 0.42), when the mean number of children in the playground in Park A increased by approximately 10% (p= 0.74) and decreased by 22% at Park B (p= 0.42)" | △ | 9 months |  |
|  |  |  | MVPA (mean number of children engaged in MVPA per 2-hour observation period) | mean (SD?) 1.17 (2.21) | mean (SD?) 0.67 (1.18) | mean (SD?) 2.86 (3.95) | mean (SD?) 1.98 (3.03) | *“After the park upgrade, there was no detectable difference between parks in the number of children engaged in MVPA (interaction between park and time: 𝑃 = 0.73); the proportion of physically active children had decreased by 41% at the intervention playground and by 32% at the comparison playground”* | ▽ | 9 months |  |
| 8. Tester 2009  CBA study  (USA) | Park renovations (artificial turf, new fencing, landscaping, lighting, and picnic benches were added. In Park A, permanent soccer goals were installed, and in Park B, a walkway around the field was restored) **vs.**  no intervention | 523 people observed in intervention parks; 483 people observed in control park (children, teens, adult males/females, seniors) | - Mean number of park visitors engaging in sedentary PA per observation period - Mean number of park visitors engaging in moderate PA - mean number of park visitors engaging in vigorous PA   Note: Data only presented for each park and per gender separately. | NR | NR | NR | NR | "*There were statistically significant increases among males and females who were observed at each respective PA level in the intervention parks. Sedentary visitors increased 5+ fold, moderately active visitors increased 3+ fold, and vigorously active visitors increased 2+fold (Table 3). On the control playfield, only moderately active males increased*" | △ | 1 year |  |
| 9. Cohen 2009  CBA study  (USA) | Park improvements ( e.g. new or refurbished gymnasiums, field improvements in watering and landscaping; improvements to picnic areas, upgrades to a walking path, and enhancements to a play-ground area..” **vs.**  No intervention | 10 parks: 5 intervention and 5 comparison parks.  Two cross-sectional samples of park users interviewed: 768 at baseline and 712 at follow-up. | Proportion exercising regularly [reporting exercising at least three times per Week] | 0.616 | 0.419 | 0.667 | 0.482 | Ratio of OR: 0.99  p = 0.812 | ▽ | Approximately 1 year |  |
|  |  |  | Reported park use (proportion) | 0.587 | 0.488 | 0.692 | 0.582 | Ration of OR: 1.01  p = 0.850 | € | Approximately 1 year |  |
| 10. Slater 2016 CBA study  (USA) | Park renovation (which involved replacing old playground equipment and ground surfacing and community engagement) **vs.**  no renovations and no community engagement | Intervention – 39 parks; Control – 39 parks | Sedentary behaviour (proportion) | Mean (SD) 18.87 (21.02) | Mean (SD) 17.62 (18.32) | Mean (SD) 17.21 (31.27) | Mean (SD) 12.60 (18.15) | beta=0.173  SE=0.089  p<0.05  95% CI [calculated]  -0.00144 to 0.34744 | ▽ | 1 year |  |
|  |  |  | Park utilization (mean number of people observed per day across parks) | Mean (SD) 35.1 (39.97) | Mean (SD) 42.26 (40.09) | Mean (SD) 29.38 (48.82)" | Mean (SD) 27.33 (38.01)" | beta = 0.211  SE = 0.063  p <0.10  95% CI [calculated]  0.08752 to 0.33448 | ▲ | 1 year |  |
|  |  |  | Park-based MVPA (mean number of people observed per day) | Mean (SD) 17.07 (21.87) | Mean (SD) 24.95 (23.93)" | Mean (SD) 12.33 (19.59)" | Mean (SD) 15.33 (20.44)" | beta = 0.199  SE = 0.089  p<0.10  95% CI [calculated]  0.02456 to 0.37344 | ▲ | 1 year |  |
| 11. Kubota 2019 CBA study  (Japan) | Construction of a new multipurpose exercise facility including indoor facilities (25 m pool, 170 m walking trail, multi-purpose gym, and group exercise rooms) and outdoor facilities (multi-purpose athletic field, 875 m walking trail, and park), accessible to all residents for a small fee + PA promotion **vs.**  No new exercise facility or PA promotion but with routine health promotion program | Intervention site: 1107 adults  Control site: 1125 adults | Percentage meeting the PA guideline (i.e. total energy expenditure ≥23 MET·hour/week) | N (%) 821 (20.2) | N (%)1018(18.5) | N (%) 845(23.2) | N (%)924(21.1) | OR 1.01  95% CI (0.86, 1.19)  p=0.91 | △ | 2 years |  |
|  |  |  | Percentage engaging in MVPA | N (%) 821 (42.6) | N (% )1018 (39.6) | N (%) 845 (44.5) | N (% )924 (43.3) | OR 0.96  95% CI (0.84, 1.09)  p=0.51 | ▽ | 2 years |  |
| 12. Branas 2011 CBA study  (USA) | Greening of abandoned vacant lots (involved removing trash and debris, grading the land, planting grass and trees to create a park-like setting, and installing low wooden post-and-rail fences around each lot’s perimeter **vs.**  No greening of vacant lots | "Greened vacant lots (intervention) - n=4,436  Control vacant lots - n=13,308" | Low Exercise (proportion responding <2 times/week) | NR | NR | NR | NR | Beta = 0.25  SE = 0.12  95% CI [calculated]: 0.0148 to 0.4852 | ▼ | 10 years |  |
| **Outcome: Anthropometry** | | | | | | | | | | | |
| 1. Goldsby 2016 CBA  (USA) | living in close proximity (near) to new inner-city park (within 1.5 miles) vs living farther away from the park (further than 5 miles) | 1443 children 2 to 17.9 years old (intervention – “near”: 45, control – “far”: 935) | BMI z-score change (for all children and subgroups: overweight/obese vs normal weight at baseline) | 0.61(1.00) | 0.66(1.09) | 0.83(1.09) | 0.87(1.11) | Regression coefficient = -0.0033, 95% CI: -0.115 to 0.109, SE = 0.0572,  p-value = 0.4804 | △ | 16 months |  |
| 2. Richardson 2020  (in Dubowitz 2019)  CBA  (USA) | Public housing development and greenspace landscaping, including changing the streetscape surrounding the developments, providing improved aesthetics (e.g. trees, grass) and walkability (e.g. sidewalks, street crossings). Renovation of current greenspace, including multiple parks, six outdoor stairwells, and three trails connecting parks **vs.**  Fewer investments, exclusively related to housing | 17 parks (8 intervention, 9 control)  Participants: 673 in intervention, 330 in control | BMI (kg/m^2^) | 30.73 (0.32) | 30.3 | 31.68 (0.67) | 31.04 | DiD=: 0.22  p=0.487 |  | 3 years |  |
|  |  |  | Proportion overweight or obese (BMI>25Kg/m^2^) | 79.46% | 77.11% | 79.30% | 75.52% | DiD= 1.43  p = 0.568 | ▽ |  |  |
|  |  |  | Proportion obese (BMI>30Kg/m^2^) | 49.17% | 46.28% | 53.57% | 50.62% | DiD =0.06  p = 0.983 |  |  |  |
| **Outcome: BP** | | | | | | | | | | |  |
| Branas 2011  ITS study  (USA) | Greening of abandoned vacant lots (involved removing trash and debris, grading the land, planting grass and trees to create a park-like setting, and installing low wooden post-and-rail fences around each lot’s perimeter vs.  No greening of vacant lots | "Greened vacant lots (intervention) - n=4,436  Control vacant lots - n=13,308" | Proportion self-reporting high BP | NR | NR | NR | NR | Beta = 0.63  SE = 0.16  95% CI 0.32 to 0.94 [calculated] | ▼ | 10 years |  |
| **Outcome: Satisfaction** | | | | | | | | | | |  |
| 1. Richardson 2020  (Dubowitz 2019 )  CBA  (USA) | Public housing development and greenspace landscaping, including changing the streetscape surrounding the developments, providing improved aesthetics (e.g. trees, grass) and walkability (e.g. sidewalks, street crossings). Renovation of current greenspace, including multiple parks, six outdoor stairwells, and three trails connecting parks **vs.**  Fewer investments, exclusively related to housing | 17 parks (8 intervention, 9 control)  Participants: 673 in intervention, 330 in control | Neighborhood satisfaction | 69.49% | 73.38% | 42.64% | 52.42% | DiD estimator: -5.89%  p-value: 0.342 | ▽ |  |  |
| **Outcome: Safety issues** | | | | | | | | | | |  |
| 1. Richardon 2020  (Dubowitz 2019) CBA  (USA) | Public housing development and greenspace landscaping, including changing the streetscape surrounding the developments, providing improved aesthetics (e.g. trees, grass) and walkability (e.g. sidewalks, street crossings). Renovation of current greenspace, including multiple parks, six outdoor stairwells, and three trails connecting parks **vs.**  Fewer investments, exclusively related to housing | 17 parks (8 intervention, 9 control)  Participants: 673 in intervention, 330 in control | Perceived neighbourhood safety | 3.03 % (SE 0.03) | 3.18% | 2.55% (SE 0.06) | 2.78% | DiD estimator = -0.08  p-value = 0.280 | ▽ |  |  |
| 2. Cohen 2009  CBA  (USA) | Park improvements ( e.g. new or refurbished gymnasiums, field improvements in watering and landscaping; improvements to picnic areas, upgrades to a walking path, and enhancements to a play-ground area..” **vs.**  No intervention | 10 parks: 5 intervention and 5 comparison parks.  Two cross-sectional samples of park users interviewed: 768 at baseline and 712 at follow-up. | Proportion reporting Perceived park safety | 0.696 | 0.913 | 0.860 | 0.774 | Ratio of ORs = 1.35  p <0.001 | ▲ | 3-14 months |  |
| 3. Slater 2016  CBA  (USA) | Park renovation (which involved replacing old playground equipment and ground surfacing and community engagement) **vs.**  no renovations and no community engagement | Intervention – 39 parks; Control – 39 parks | Neighborhood safety (crime count) | mean (sd) 747.89 (904.68) | Mean (sd) 622.58 (721.28) | Mean (sd) = 579.41 (385.11) | mean (sd) 498.90 (297.18) | NR  Crime count reduced in both groups  “Neighborhood crime was also associated with increased park utilization” | ? | 1 year |  |

*Where provided, we report the number of participants in the intervention group and control group, separately. Where this is not provided, we report the total sample. Where the number of participants is not reported in the study, we could not provide it here.

## 1.2 Active transport interventions

| **Study ID**  **(country)** | **Comparison** | **Participants at baseline (n)*** | **Outcome measure reported** | **Intervention** | | **Control** | | **Effect measure reported** | **Effect direction** | **Time of outcome measure** |
| --- | --- | --- | --- | --- | --- | --- | --- | --- | --- | --- |
|  |  |  |  | **Baseline value** | **Follow-up** | **Baseline value** | **Follow-up** |  |  |  |
| **Outcome: Physical activity** | | | | | | | | | | |
| 1. Fitzhugh 2010  CBA  (USA) | Building greenway/trail vs no intervention | Intervention – 1 neighbourhood; control – 2 neighbourhoods | 2-hr counts of total PA | Median: 4.5 (IQR: 3.0-6.0) | median: 13.0 (IQR: 11.0-15.0) | median: 3.0 (IQR: 0.0-8.0) | median: 1.0 (IQR: 0.0 - 6.0) | NR, p = 0.001  “… the experimental neighborhoods’ change in physical activity was found to be significantly different from the control neighborhoods’ for pedestrian (p=0.001); cycling (p=0.038); and total physical activity (p=0.001)”. | **▲** | 2 years |
|  |  |  | 2-hr observation counts for active transport to school (ATS) | median: 8.5 | Median: 9.0 | Median: 30.0 | median: 19.0 | NR, p = 0.2061  “… Wilcoxon rank sums test detected no signifıcant difference in the pre–post intervention change in ATS for the experimental compared to the control schools (p=0.2061)." |  | 2 years |
| 2. Østergaard 2015 CBA  (Denmark) | Physical environment changes plus 'soft' interventions (motivation and safety encouragement) vs. no intervention  Interventions to increase cycling: structural changes near the school in e.g. road surface, signposting and traffic regulation such as one-way streets and regulation of car drop off zones" |  | PA from cycling (number of trips to and from school in previous week) | 5.8 (4.4) | NR | 6.4 (4.3) | NR | Change beta coefficient: 0.15; 95% CI: -0.25 to 0.54; p-value = 0.463 | △ | 1 year |
|  |  |  | Leisure time physical activity | "Least active: 11.0%; Most active: 46.2%" | NR | "Least active: 9.2%; Most active: 48.7%" | NR | Change beta coefficient: -0.09, 95% CI: (-0.21; 0.03); p-value = 0.124 |  | 1 year |
|  |  |  | PA from cycling– long term school cycling | Always/ almost always: 54.8%;  Sometimes: 25.8%;  Never/ hardly ever: 19.4%" | NR | Always/ almost always: 60.6%; Sometimes: 21.2%;  Never/ hardly ever: 18.2%" | NR | Change beta coefficient:  -0.02; 95% CI: -0.10 to 0.05; p-value: 0.485 |  | 1 year |
|  |  |  | PA from cycling - cycling last week beyond school cycling | Often or very often: 31.7%;  Sometimes: 43.1%;  Seldom or not at all: 25.3%" | NR | Often or very often: 37.7%;  Sometimes: 43.0%;  Seldom or not at all: 19.3%" | NR | Change beta coefficient: -0.04; 95% CI: -0.14 to 0.05; p-value = 0.355 |  | 1 year |
| 3. Goodman 2013 CBA  (UK) | Town-level cycling initiative (infrastructure and health promotion) vs Matched comparison | Intervention – 37 urban census areas; Control – 27 urban census areas | Proportion of commuters cycling to work | 5.81%; (5.77; 5.86) | 6.78%; (6.74; 6.83) | 4.03% (3.99; 4.08) | 4.32%; (4.28; 4.36)" | coefficient: 0.69; 95% CI: 0.60; 0.77. | ▲ | 10 years |
| 4. Rissel 2015 CBA  (Australia)  Crane 2017 | building cycling infrastructure vs no intervention | Intervention – 398 adult residents; Control – 448 adult residents | MVPA (min/week) | "mean (sd) 239.5  (274.5); n = 398" | "mean (sd)184.1 (209.8)  ; n =240" | mean (sd) 211.1 (229.6);  n = 448" | "mean (sd) 198.1 (219.6);  n = 272 | DID = -42.4 [calculated] | ▽ | 4 months |
|  |  |  | MVPA (min/week) | "mean (sd) 239.5  (274.5); n = 398 | mean (sd) 204.0 (252.9);  n =189 )" | mean (sd) 211.1(229.6);  n = 448" | "mean (sd) 180.5(197.6);  n = 229" | DID =-4.9 [calculated] | ▽ | 16 months |
|  |  |  | Proportion cycling last week | 29.2% | 25.8% | 22.4% | 23.2% | Adjusted OR: 1.07; 95% CI: 0.67 to 1.69; p-value: 0.767 | △ | 6 months |
|  |  |  | Cycling frequency (odds of cycling at least weekly) | n (%)127 (31,9) | n (%) 62 (25.8); | n (%) 110 (24.6); | n (%) 63 (23.2); | AOR: 1.59; 95%CI: 0.51 to 4.98 | △ | 4 months |
|  |  |  |  | n (%) 127 (31,9); | n (%) 49 (25.9); | n (%) 110 (24.6) | n (%) 48 (21.0); | AOR: 2.79  95%CI: 0.81 to 9.66 | △ | 16 months |
|  |  |  | Proportion usually cycling to and from work/study | 14.1% | 11.3% | 12.7% | 7.7% | NR; p=0.4  “There was a reduction in travel to work or study by bicycle (p = 0.001) between baseline and follow-up, observed across both intervention (14.1–11.3 % at follow-up) and comparison areas (12.7–7.7 % at follow-up) (p = 0.40). | △ | 12 months |
| 5. Brown 2016 CBA (USA) | Participants living near (within 800 m) of the intervention street vs those living far from intervention street  Street improvements included new bike lanes, wider and better lit sidewalks | 910 residents | Transit-related active transportation trips | prop=0.21, SD=0.41. | prop=0.39 | prop=0.15, SD=0.35 | 0.25 | OR = 1.48; 95% CI: 1.14 to 1.68, p=0.01 | ▲ | 12 months |
|  |  |  | Non-transit walking | prop=0.35, SD=0.48. | prop=0.56 (fig 1) | prop=0.13, SD=0.34. | prop=0.25 (fig 1) | OR=0.26; 95% CI: 0.17 to 0.4, p=0.00 | ▼ | 12 months |
|  |  |  | Bicycling | prop=0.07, SD=0.26. | prop=0.08 | prop=0.04, SD=0.18 | prop=0.09 | OR=0.85; 95% CI: 0.43 to 1.68, p=0.63 | ▽ | 12 months |
| 6. Benjamin Neelon 2015 CBA  (USA) | Built environment changes including new sidewalks and crosswalks vs no intervention | Intervention – 64 children; Control – 40 children | MVPA (min/hr) | mean=4.0 SD=1.7 | mean=4.2 SD=1.9 | mean=3.8 SD=2.0 | mean=3.4 SD=1.5 | Regression coefficient: 1.3; 95% CI: 0.2 to 2.3; p-value=0.03 | ▲ | 12 months |
| 7. Prins 2017_CBA analysis | New motorway vs no motorway | 1412 adults from two urban areas | Participation in MVPA (proportion) | 65.5 (n=220) | 71.9 (n=231) | 62 (n=234) | 68.5 (n=254) | OR: 0.95 ( 95% CI: 0.53 to 1.72) | ▽ | 8 years |
|  |  |  | MVPA time (min/week) | mean: 574.9, sd: 542.8 | mean: 492.9, sd: 440.1 | mean: 573.1, sd: 534.2 | mean: 531.1, sd: 448.7 | IRR: 0.94; 95% CI: 0.71 to 1.25 | ▽ |  |
|  |  | 1499 adults from two urban areas | Participation in walking (proportion) | 86.3 | 85.6 | 81.3 | 81.7 | OR: 0.95; 95% CI: 0.47 to 1.93 | ▽ | 8 years |
|  |  |  | Walking time (min/week) | mean: 391.4, sd: 404.7 | mean: 357.4, sd: 338.0 | mean: 419.1, sd: 372.6 | mean: 352.1, sd: 352.2 | IRR: 1.08 95% CI: 0.83 to 1.40 | △ | 8 years |
|  |  | 1318 adults from two urban areas | Sedentary behaviour (min/day) | mean: 398.5, sd: 253.0 | mean: 402.1, sd: 247.2 | mean: 382.6, sd: 245.2 | mean: 367.1, sd: 226.9 | Regression coefficient: 20.72; 95% CI: -42.95 to 84.03 | △ | 8 years |
| Prins 2017_Cohort analysis | new motorway vs no motorway | 248 adults from two urban areas | Participation in walking (proportion) | 88.8 | 86.5 | 89.8 | 88.6 | OR: 0.68; 95% CI: 0.24 to 1.89 | ▽ | 8 years |
|  |  |  | Walking time (min/week) | mean=401.7, SD: 366.9 | mean=346.3, SD: 328.7 | mean:380.6, SD: 361.5 | mean: 384.7, SD: 381.9 | IRR: 0.82; 95% CI: 0.62 to 1.10 | ▽ | 8 years |
|  |  | 214 adults from two urban areas | Participation in MVPA (proportion) | 77.8 | 75 | 75.3 | 84 | OR: 0.60; 95% CI: 0.25 to 1.43 | ▽ | 8 years |
|  |  |  | MVPA time (min/week) | 568.8, SD: 508.9 | mean: 504.1, SD: 401.8 (n=49) | mean: 436.5, SD: 466.7 (n=55) | mean: 563.5, SD: 634.4) (n=55 | IRR: 0.94; 95% CI: 0.68 to 1.31 | ▽ | 8 years |
|  |  | 215 adults from two urban areas | Sedentary behaviour (min/day) | mean: 391.7, SD: 213.9 | mean: 405.3, sd: 247.3 | mean: 428.8, SD: 227.8 | mean: 367.2, SD: 210.9 | Regression coefficient: 52.46; 95% CI: -15.70 to 120.62) | ▽ | 8 years |
| 8. McDonald 2013  CBA  USA | Schools with SRTS* programme (education + covered bike parking) vs schools with no SRTS programs  [Policy comprised many different components from walking and cycling lanes to education etc] | Intervention Schools - 9; control schools - 5 | proportion biking | NR | NR | NR | NR | marginal effect: 0.106; 95% CI: 0.018 to 0.195 | ▲ | 5 years |
|  | Schools with SRTS programme (education + Sidewalks/crosswalks) vs schools with no SRTS programs |  | proportion walking | NR | NR | NR | NR | Marginal effect: 0.064; 95% CI: -0.002 to 0.130 | △ | 5 years |
| 9. Grunseit 2019  ITS  (Australia) | Trends before and after the construction of multi-use recreational walking and cycling loop trail | All cyclists riding on two trails | Trail use (immediate effect): Counts of bike passes (at Jamieson park) | NR | NR | NR | NR | adj beta 1899 95% CI 1672, 2126 | ▲ | 120 time points "19 weeks February 25th to July 14th  (weeks 9 to 28) for each year 2013, 2014 and 2015" |
|  |  |  | Trail use: Counts of pedestrian passes (at Jamieson park) | NR | NR | NR | NR | adj beta 812 95% CI 161, 1462 | ▲ |  |
|  |  |  | Trend in trail use: for bikes (Jamieson park) effect over time | NR | NR | NR | NR | adj beta -62 95% CI -80 to -44 | ▼ |  |
|  |  |  | Trend in trail use for pedestrians (Jamieson park) | NR | NR | NR | NR | adj beta -8 95% CI -42 to 27 | ▽ |  |
| 10. Dill 2014  CBA  (USA) | Installation of bicycle boulevards vs no installation | 255 parents living at 8 intervention and 11 control street segments | Minutes of MVPA per day | mean (SD) 39.5 (21.9) | mean (SD) 35.6 (19.0) | mean (SD) 35.4 (20.8) | mean (SD) 34.8 (19.4) | beta-coefficient: −3.44  p-value=0.33 | ▽ | 1 year |
|  |  | 101 parents living at 8 intervention and 11 control street segments | Min of biking per day | Mean (SD) 103.9 (73.0) | Mean (SD) 65.9 (74.7) | Mean (SD) 76.8 (69.4) | Mean (SD) 72.7 (55.3) | beta-coefficient: −1.09  p=0.00 | ? |  |
|  |  | 195 parents living at 8 intervention and 11 control street segments | Min walking per day | 107.2 (79.1) | 89.4 (66.8) | 92.0 (86.9) | 75.4 (66.5) | Beta coefficient: −0.096 (p=0.54) | ? |  |
| 11. Frank 2019  CBA  (Canada) | Proximity to infrastructure changes (greenway development); close vs. further away | Intervention – 219 residents; Control – 265 residents | MVPA (average minutes/day) | 51.9 (95% CI 43.2, 60.5) | 62.9 (95% CI 47.6, 78.2) | 58.7 (95% CI 48.1, 69.3) | 52.8 (95% CI 43.6, 62.0) | NR  Calculated: MD 10.10; 95% CI -7.51 to 27.71 | △ | 1 year? |
|  |  |  | MVPA (proportion engaging in >20min/day) | 67.6% (95% CI 61.3, 73.8) | 69.4% (95% CI = 63.3, 75.6 | 68.7% (95% CI 63.1, 74.3) | 60.8% (95% CI = 54.8, 66.7) | OR 2.00  95% CI:  1.00 to 3.98 | △ |  |
|  |  | Intervention – 222 residents; Control – 264 residents | Sedentary behaviour (proportion engaging in >9 hrs/d) | 487.7 (449.4, 526.0) | NR | 473.8 (444.7, 502.8) | NR | OR 0.46  95% CI 0.25 to 0.85 | ▲ |  |
| 12. Hong 2016  CBA  (USA) | New light rail line; treatment group (residing <½ mile) vs control group (>½ mile) | Intervention – 101 residents; Control – 103 residents of an urban area | Average daily walk trips | Mean (SD) 1.00 (1.12) | Mean (SD) 1.29 (1.63) | Mean (SD) 0.79 (0.97) | Mean (SD) 0.79 (1.07) | beta=0.52  p= 0.021 | ▲ | 1 year |
|  |  | Intervention – 32 residents; Control – 41 residents of an urban area | Average minutes of daily MVPA | Mean (SD) 23.09 (17.49) | Mean (SD) 21.52 (16.24) | Mean (SD) 19.81 (18.01) | Mean (SD) 18.56 (17.02) | Coefficient = -0.34  P= 0.063* | △ |  |
| 13. Hirsch 2017  CBA  (USA) | Before vs after infrastructure changes for those near (25^th^ percentile/1.08km) the infrastructure changes (construction of an off-road trail system) | 116 census tracts (population differed at different timepoints) | Proportion commuting to work by bicycle | mean (sd)1.76% (1.96%) | mean (sd)4.04% (3.48%) | NR | NR | coefficient 2.03; 95% CI (0.13; 3.93) | ▲ | 10 years |
| 14. West 2011  CBA  (USA) | Living near (within .5 miles) vs far (within .51–1.0 miles) to new greenway construction | Intervention – 93 residents; Control – 73 residents | Walking (number of days/week) | mean = 3.00; SD = 2.47 | mean = 3.48; SD =2.39 | mean = 2.84; SD = 2.25 | mean = 3.10; SD = 2.27 | Wilks’s Lambda = .995, F(1, 164) = .832, P < 0.363 | △ | 1 year |
|  |  |  | moderate PA (number of days/week) | Mean (SD) 1.76 (1.99) | Mean (SD) 2.39 (1.93)  0.63 | mean = 1.63; SD = 1.81 | mean = 2.11; SD = 1.91 | "Wilks’s Lambda = .997, F(1, 165) = . 509", P < 0.476 | △ | 1 year |
|  |  |  | Vigorous PA (nr of days/week) | mean = 1.41; SD = 1.69 | mean = 1.87; SD = 1.71 | mean = 1.25; SD = 1.79 | mean = 1.71; SD = 1.78 | Wilks’s Lambda = 1.000, F(1, 165) = .002"P <.962" | △ | 1 year |
| 15. Pazin 2016  CBA  (Brazil) | living nearer (0-500m) vs farther away (501-1000) from new walking and cycling route | Intervention – 192 adults; Control – 137 adults from 6 urban neighborhoods | Walking in previous week (min/week) | mean (95% CI): 57 (48 – 67) | mean (95% CI): 89 (72 – 106) | mean (95% CI):  65 (48 – 82) | mean (95% CI): 58 (49 – 67) | NR  Naïve DID = 39 | △ | 3 years |
|  |  |  | MVPA in previous week (min/week) | mean (95% CI)  65 (49 to 81) | mean (95% CI)  82 (64 to 101) | mean (95% CI)  80 (53 – 108) | mean (95% CI)  77 (55 – 98) | NR | △ | 3 years |
|  |  |  | MVPA + walking in previous week (min/week) | mean (95% CI): 107 (90 to 124) | mean (95% CI): 158 (130 to 187) | mean (95% CI): 149 (105 to 193) | Mean (95% CI): 128 (99 to 156) | NR  Naïve DID=72 | △ | 3 years |
| 16. Chapman 2014 (Keall 2015)  CBA  New Zealand | "the introduction of cycle and walkway infrastructure, along with measures to encourage active travel" vs no intervention | 4861 trips | Proportion engaged in active travel | 19.7% (n=111) (unadjusted) | 17.8% (n=151) (unadjusted) | 19.4% (n=131) (unadjusted) | 15.0% (n=132) (unadjusted) | OR 1.37 (1.08 to 1.73) | ▲ | 1 year |
| 17. Skov-Petersen 2017  ITS  (Denmark) | cycle highways (Albertslund) upgrade vs no upgrade | 50,954 counts | Bike volume (cyclists/hr) | NR | NR | NR | NR | Beta: 0.95  error: 0.89  p=0.2858 | △ | 35 months |
|  | cycle greenway upgrade (Vestvolden) vs no upgrade |  |  | NR | NR | NR | NR | Beta: 3.15,  error: 1.11,  p=0,0046 | ▲ |  |
| **Anthropometry** | | | | | | | | | | |
| 1. Østergaard 2015 CBA  (Denmark) | Physical environment change plus 'soft' interventions (motivation and safety encouragement) vs. no intervention | 1390 children | Change in BMI | Mean (SD) 18.24 (2.93) | NR | Mean (SD) 18.23 (2.84) | NR | beta coefficient: 0.01;  95% CI: (-0.13; 0.15);  p-value: 0.887 | ▽ | 1 year |
| 2. Benjamin Neelon 2015 CBA  (USA) | Active transport vs. no intervention | 104 children; Intervention –64; Control – 40 | BMI Z-score | Mean (SD) 0.6 (1.2) | NR | Mean (SD) 1.2 (1.2) | NR | Regression Coefficient: -0.5; 95% CI = -0.9 to -0.02; p-value = 0.045 | ▲ | 1 year |
| **Adverse event - injuries** | | | | | | | | | | |
| 1. Østergaard 2015 CBA | Physical environment change plus 'soft' interventions (motivation and safety encouragement) vs. no intervention | 1684 children; Intervention –897; Control – 641) | Cycling injuries frequency | 193 | 184 | 147 | 137 | NR  Naïve DID = 1 | ▽ | 1 year |
| **Outcome: Satisfaction** | | | | | | | | | | |
| Jung 2017 CBA | Design street project (including the improvement of sidewalks, public spaces, signs, fences, and other physical elements of the streets) **vs** typical street | Intervention – 2016 pedestrians; Control – 15,686 pedestrians | Pedestrian satisfaction score | 3.213 | 3.355 | 3.256 | 3.092 | coefficient=0.291; (SE = 1.31), p<0.05 | ▲ | 3 years |
| **Outcome: Mental health** | | | | | | | | | | |
| Prins 2017_CBA analysis | Construction of a new motorway (also hypothesized to remove traffic from local streets and create a more pedestrian- and cycle-friendly environment) **vs** no motorway | n=1778 | Mental well-being (MCS-8 score) | NR | NR | NR | NR | Regression coefficient: -0.8; 95% CI: –3.1 to 1.5 | ▽ | 8 years |
| Prins 2017_Cohort analysis |  | n=336 | Mental well-being (MCS-8 score) | NR | NR | NR | NR | Regression coefficient: 0.7; 95% CI: -1.6 to 3.0 | △ | 8 years |

*Where provided, we report the number of participants in the intervention group and control group, separately. Where this is not provided, we report the total sample. Where the number of participants is not reported in the study, we could not provide it here; SRTS: Safe Routes to School

# 2. Interventions addressing policy and regulations

## 2.1 Access to PA facilities

| **Study ID**  **(country)** | **Comparison** | **Participants at baseline (n)** | **Outcome measure reported** | **Intervention** | | | **Control** | | **Effect measure reported** | **Effect direction** | **Time of outcome measure** |
| --- | --- | --- | --- | --- | --- | --- | --- | --- | --- | --- | --- |
|  |  |  |  | **Baseline value** | **Follow-up** | **Baseline value** | | **Follow-up** |  |  |  |
| **Outcome: Physical activity** | | | | | | | | | | | |
| 1a. Higgerson 2018_ITS  UK | Re re:fresh scheme: provision of free access to local government leisure facilities at most times of the day (some including swimming pools or gyms) vs.  No intervention | Gym and swim attendees of a leisure center | Increase in activity (gym or swimming) (based on logged attendances) | NR | NR | NR | | NR | RR = 1.64,  95% CI 1.43 to 1.89,  p<0.001 | **▲** | 7 years |
| 1b. Higgerson 2018_CBA  UK | Re re:fresh scheme vs rest of England | Adult residents of two urban areas | Proportion of individuals undertaking at least 30min of moderately intensive gym or swimming activity in previous 4 weeks | NR | NR | NR | | NR | 3.9%  95% CI: 3.6 to 4.1 | **▲** | 7 years |
|  |  |  | Proportion participating in any sport or active recreation for a least 30 min on at least 12 days over the last four weeks | NR | NR | NR | | NR | 1.9%  95% CI: 1.7 to 2.1) | **▲** | 7 years |

*Where provided, we report the number of participants in the intervention group and control group, separately. Where this is not provided, we report the total sample. Where the number of participants is not reported in the study, we could not provide it here.

## 2.1 Free bus travel

| **Study ID**  **(country)** | **Comparison** | **Participants at baseline (n)** | **Outcome measure reported** | **Intervention** | | **Control** | | **Effect measure reported** | **Effect direction** | **Time of outcome measure** |
| --- | --- | --- | --- | --- | --- | --- | --- | --- | --- | --- |
|  |  |  |  | **Baseline value** | **Follow-up** | **Baseline value** | **Follow-up** |  |  |  |
| **Outcome: Physical activity** | | | | | | | | | | |
| Green 2014  CBA  London | Free bus travel in 12-17 year old’s vs no free bus travel in 25-59 year old’s | Intervention group -4206; Control group - 31169 | Number of bus trips | Proportion: 0.64 | Proportion: 0.87 | Proportion: 0.25 | Proportion: 0.34 | Ratio of ratios: 1.0 (95% CI: 0.89 to 1.10) | **€** | 3 years |
|  |  |  | Number of walking trips | Proportion: 0.99 | Proportion: 0.83 | Proportion: 0.83 | Proportion: 0.91 | Ratio of ratios: 0.76 (95% CI 0.70 to 0.85) | **▼** | 3 years |
|  |  |  | Number of cycling trips | Proportion: 0.06 | Proportion: 0.04 | Proportion: 0.05 | Proportion: 0.07 | Ratio of ratios: 0.53 (95% CI 0.35 to 0.87) | **▼** | 3 years |
|  |  |  | Distance travelled by walking | Proportion: 1.42 | Proportion: 1.41 | Proportion: 1.32 | Proportion: 1.33 | Ratio of ratios: 0.99 (95% CI 0.92 to 1.07) | ▽ | 3 years |
|  |  |  | short distance trips by walking as main mode | %: 82 | %: 81 | %: 70 | %: 76 | Ratio of ratios: 0.91 (95% CI 0.87 to 0.97) | **▼** | 3 years |
|  |  |  | Distance travelled by cycling | Proportion: 1.1 | Proportion: 0.06 | Proportion: 0.2 | Proportion: 0.31 | Ratio of ratios: 0.37 (95% CI 0.18 to 0.65) | **▼** | 3 years |
|  |  |  | short distance trips by cycling as main mode | %: 3 | %: 1 | %: 1 | %: 1 | Ratio of ratios: 0.54 (95% CI 0.22 to 1.17) | ▽ | 3 years |
| **Outcome: Adverse events** | | | | | | | | | | |
| Green 2014  CBA  London | Free bus travel in 12-17 year olds vs no free bus travel in 25-59 year olds | NR | Incidence of Road Traffic Injuries for all transport modes | 5.46 per 1000 person years | 3.23 per 1000 person years | 5.81 per 1000 person years | 4.08 per 1000 person years | Ratio of ratios 0.84  95% CI 0.82 to 0.87 | **▲** | 3 years |
| **Outcome: Safety** | | | | | | | | | | |
| Green 2014  CBA  London | Free bus travel in 12-17 year olds vs no free bus travel in 25-59 year olds | NR | Rate of hospitalisation for injuries inflicted by assaults | 1.13 admissions per 1000 person-years | 1.61 admissions per 1000 person-years | 0.77 admissions per 1000 person-years | 0.91 admissions per  1000 person-years | Relative effect 19% 95% CI 16% to 22% | **▼** | 3 years |

*Where provided, we report the number of participants in the intervention group and control group, separately. Where this is not provided, we report the total sample. Where the number of participants is not reported in the study, we could not provide it here.
